# SAR and molecular mechanism studies of monoamine oxidase inhibition by selected chalcone analogs

**DOI:** 10.1080/14756366.2019.1593158

**Published:** 2019-03-27

**Authors:** Raed Shalaby, Jacobus P. Petzer, Anél Petzer, Usman M. Ashraf, Ealla Atari, Fawaz Alasmari, Sivarajan Kumarasamy, Youssef Sari, Ashraf Khalil

**Affiliations:** a Department of Pharmaceutical Sciences, College of Pharmacy, Qatar University, Doha, Qatar;; b Department of Pharmaceutical Chemistry, School of Pharmacy and Centre of Excellence for Pharmaceutical Sciences, North-West University, Potchefstroom, South Africa;; c Department of Physiology and Pharmacology, Centre for Hypertension and Personalized Medicine, University of Toledo College of Medicine, Toledo, OH, USA;; d Department of Pharmacology and Experimental Therapeutics, College of Pharmacy and Pharmaceutical Sciences, The University of Toledo, Toledo, OH, USA

**Keywords:** Monoamine oxidase, chalcone, reversibility, dopamine, mRNA

## Abstract

The present study describes the synthesis of a series of 22 chalcone analogs. These compounds were evaluated as potential human MAO-A and MAO-B inhibitors. The compounds showed varied selectivity against the two isoforms. The IC_50_ values were found to be in the micromolar to submicromolar range. The *K_i_* values of compound 16 were determined to be 0.047 and 0.020 μM for the inhibition of MAO-A and MAO-B, respectively. Dialysis of enzyme-inhibitor mixtures indicated a reversible competitive mode of inhibition. Most of the synthesized chalcone analogs showed a better selectivity toward MAO-B. However, introducing of 2,4,6-trimethoxy substituents on ring B shifted the selectivity toward MAO-A. In addition, we investigated the molecular mechanism of MAO-B inhibition by selected chalcone analogs. Our results revealed that these selected chalcone analogs increased dopamine levels in the rat hepatoma (H4IIE) cells and decreased the relative mRNA expression of the MAO-B enzyme.

## Introduction

Neuronal signaling is mediated by several neurotransmitters, including monoamine neurotransmitters such as norepinephrine, dopamine, and serotonin. The levels of monoamines in the brain are controlled by a balance between their enzyme-catalyzed synthesis and breakdown, while both reuptake carriers and vesicular transporters regulate the concentrations of these neurotransmitters in the synapse. Alterations of the monoamines homeostasis are associated with many neurological conditions such as depression, autism, drug addiction, and neurodegenerative diseases [Alzheimer’s disease (AD) and Parkinson’s disease (PD)]^1^. Monoamine oxidases (MAOs, EC 1.4.3.4) are among a family of flavin adenine dinucleotide (FAD)-dependent enzymes that play a key role in the breakdown of endogenous and exogenous amines^2^. These enzymes are located on the mitochondrial outer membrane, mostly in the brain, and are also present in the liver, gut, intestine, skin, placenta, lymphocytes, and platelets^3^.

MAOs are responsible for catalyzing the oxidative deamination of various biogenic amine neurotransmitters and a variety of xenobiotic amines, and modulate their concentrations in the brain and peripheral tissues^4^. The MAO enzymes exist as two isoforms, MAO-A and MAO-B, with a sequence similarity of 73% but with different inhibitor selectivity, substrate specificity, and tissue distribution^5^. The MAO-A isoform predominantly deaminates serotonin, norepinephrine, and epinephrine, whereas MAO-B has substrate specificity for benzylamine and β-phenylethylamine. Tyramine and dopamine are common substrates for both isoforms^6^. The amine in the deprotonated form binds to the active site of the enzyme and is oxidized to the corresponding imine with the reduction of the FAD cofactor to its hydroquinone form.

The reduced FAD cofactor reacts with oxygen (O_2_) to regenerate FAD to its oxidized form. During this process, hydrogen peroxide is produced, which may generate highly reactive hydroxyl radicals that may lead to the development of neurodegenerative processes and neuronal death^7^^,^^8^. As mentioned, the imbalance in the brain neurotransmitters concentration is linked with various neurodegenerative diseases and psychiatric disorders, including depression, PD, AD, and Huntington’s disease^9^^,^^10^. Several studies have confirmed that MAO-B is overexpressed in the brains of AD patients and is attributed to the loss of cognitive functions^11^. Other reports revealed positive correlation between the low level of neurotransmitters in patients with severe depression and the increased concentrations of MAO-A.

MAO inhibitors block the enzyme catalytic activities and halt the oxidative deamination of these neurotransmitters. Inhibition of MAOs can lead to increase in the concentrations of neurotransmitters stored in the nerve terminals (e.g. serotonin and dopamine). Thus, MAO inhibitors can be developed as therapeutic agents, particularly for disease states where MAO enzyme is overexpressed. As aforementioned, MAO catalyzes metabolism of monoamine neurotransmitters, which may generate byproducts potentially injurious species (e.g. hydrogen peroxide and aldehydes), and lead to neuronal damage. MAO inhibitors can reduce the production of the reactive oxygen species (ROS) by halting the MAO-catalyzed oxidation process and hence prevent their neurotoxic effect. This is in agreement with a prior study demonstrating that MAO inhibitors are potential neuroprotective agents^12^.

The X-ray crystal structures of human MAO-A and MAO-B revealed that human MAO-A has a single substrate cavity with a volume of ∼500Å^3^, while human MAO-B has a bipartite cavity structure with an entrance cavity (∼300Å^3^) and a substrate cavity (∼400Å^3^). These two cavities are separated by the side chains of Ile199 and Tyr326^13^. Mutagenesis experiments with the human MAO-B mutant, Ile199Phe, indicated that the bulky Phe side chain reduces conformational flexibility of this residue in MAO-B, and as a result impedes the binding of larger cavity-spanning MAO-B selective inhibitors to both the active and entrance cavities^14^. The smaller side chain of the Ile199 residue, in turn, may rotate from the active site cavity to permit the binding of cavity-spanning inhibitors. Thus, Ile199 serves as gating residue and a structural determinant for substrate and inhibitor recognition by MAO-B^15^. A single Ile199Ala mutation to MAO-B permanently opens the “gate.” Tyr326, another key residue that determines substrate and inhibitor specificity, also exhibits conformational changes on the inhibitor binding and restricts the binding of certain inhibitors (e.g. harmine) to human MAO-B. In MAO-A, the corresponding residue is Ile335, which is much smaller and this allows for the binding of inhibitors such as harmine. The double mutations Ile199Ala-Tyr326Ala in MAO-B exhibit inhibitor binding properties similar to those of MAO-A rather than to MAO-B.

**Figure 1.**
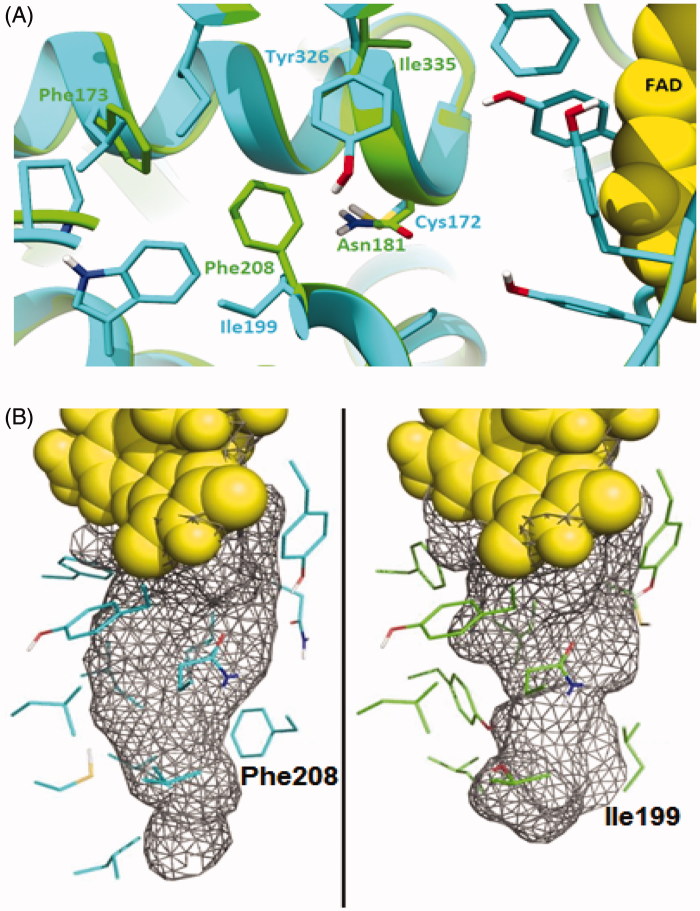
(A) Superimposition of the binding sites of both hMAO-A (green) and hMAO-B (cyan), showing the different residues. (B) Binding site of both hMAO-A and hMAO-B in mesh representation. Phe208 in hMAO-A is bulkier than Ile199 in hMAO-B and creates a “bottleneck” in the middle of the pocket making it smaller in size.

This analysis indicates the importance of the bipartite cavity structure of MAO-B to substrate and inhibitor recognition, and in distinguishing its specificities from those of MAO-A. It has been reported that Ile199 and Tyr326 can act as gating residues in human MAO-B. With long inhibitors, the side chain of Ile 199 rotates into an open conformation leading to the merging of the two cavities to form one large cavity of about 700Å^3^ to accommodate the long inhibitor. This information would be helpful in designing selective MAO-A and MAO-B inhibitors (Figure 1).

Most of the marketed selective MAO-B inhibitors are irreversible and can cause potential adverse side effects due to long-lasting enzyme inhibition. However, safinamide was recently approved as the first reversible selective MAO-B inhibitor for the treatment of PD. There is an increase interest in chalcone as a new promising scaffold for the development of novel reversible MAO-B inhibitors. In our previous work, we explored the effect of introducing a benzodioxol ring in the chalcone molecule in combination with different substituents^13^. Computer-aided docking studies confirmed the role of the carbonyl group in forming a hydrogen bond with the SH group of Cys172. Based on our previous findings, we designed and prepared a series of chalcone analogs that showed different MAO inhibitory activities and selectivities. In addition, the molecular mechanism of MAO inhibition by chalcone analogs remains unclear with respect to their effects of protein expression. The main question to be answered is whether chalcone analogs inhibit the enzyme expression in addition to the inhibition of MAO activity.

## Experimental section

### General methods

All solvents and fine chemicals were purchased from Sigma–Aldrich (unless specified otherwise) and were used without further purification. For the MAO inhibition studies, recombinant human MAO-A and human MAO-B (5 mg protein/mL) from Sigma–Aldrich were used. Analytical thin layer chromatography was carried out on precoated silica gel 60 F254 aluminum plates purchased from Merck and visualized by UV. Melting points were measured on a Stuart SMP40 automatic melting point apparatus and are uncorrected. For all of the prepared compounds, infrared (IR) spectra were recorded with a Perkin Elmer Spotlight 400 Fourier transform infrared (FTIR) imaging system. GC-MS was performed on a Thermo instrument (ISQELTL). Proton (^1^H) and carbon (^13^C) NMR spectra were recorded on JEOL 600 MHz using CDCl_3_ as a solvent at the Central Lab Unit, Qatar University and a Bruker Avance III spectrometer (700 MHz) at the King Abdullah Institute for Research & Consulting Studies, King Saud University, Riyadh, Saudi Arabia. The chemical shifts were reported as parts per million (δ) relative to the solvent peak and coupling constants are given in hertz (Hz). The original spectra are provided in the Supporting information. Purity of compounds were confirmed with C, H and S analysis performed on Thermo Scientific FLASH 2000 CHNS/O analyzer.

### General synthetic procedure

The proposed Chalcone analogs were synthesized using the Claisen–Schmidt condensation reaction. To a stirred solution of the appropriate acetophenone (1 mmol) and the substituted benzaldehyde (1 mmol) in ethanol at 0 °C, a sodium hydroxide solution (0.12 g; 3 mmol in minimum amount of water) was added dropwise. The reaction mixture was stirred at room temperature overnight. The obtained precipitate was filtered, washed with cold water and recrystallized from ethanol to produce chalcones **1**–**22**.

#### 1. (E)-3–(4-(dimethylamino)phenyl)-1–(3-(trifluoromethyl)phenyl)prop-2-en-1-one

Orange crystals, yield = 50%, m.p. = 96–99 °C. ^1^H NMR (600 MHz, Chloroform-*d*) δ 8.25 (s, 1H), 8.18 (d, *J* = 8.4 Hz, 1H), 7.84 (d, *J* = 15 Hz, 1H), 7.8 (d, *J* = 8.4 Hz, 1H), 7.63 (dd, *J* = 7.8 Hz, 7.8, 1H), 7.57 (d, *J* = 9 Hz, 2H), 7.31 (d, *J* = 16 Hz, 1H), 6.71 (d, J = 9 Hz, 2H), 3.10 (s, 6H).

EI-MS (*m*/*z*): calculated 319.1, observed 319.1 (M^+^).

#### 2. (E)-3–(4-(dimethylamino)phenyl)-1–(4-(trifluoromethyl)phenyl)prop-2-en-1-one

Yellow crystals, yield = 62%, m.p. = 141–145 °C. The obtained analytical data matched with the previously published finding^16^.

#### 3. (E)-1–(4-bromophenyl)-3–(4-(dimethylamino)phenyl)prop-2-en-1-one

Yellow crystals, yield = 54%, m.p. = 139–142 °C. ^1^H NMR (600 MHz, Chloroform-*d*) δ 7.87 (dd, *J* = 6.6, 1.8 Hz, 2H), 7.80 (d, *J* = 15.6 Hz, 1H), 7.62 (dd, *J* = 6.0, 1.2 Hz, 2H), 7.55 (d, *J* = 9 Hz, 2H), 7.27 (d, *J* = 15.0 Hz, 1H), 6.70 (d, *J* = 9 Hz, 2H), 3.10 (s, 6H). ^13 ^C NMR (151 MHz, CDCl_3_) δ 189.4, 152.2, 146.4, 137.8, 131.7, 130.6, 130.0, 127.1, 122.4, 116.2, 111.8, 40.1 EI-MS (*m*/*z*): calculated 329.0, observed 329.0 (M^+^).

#### 4. (E)-1–(4-chlorophenyl)-3–(4-(dimethylamino)phenyl)prop-2-en-1-one

Yellow crystals, yield = 53%,

The obtained analytical data matched with the previously published^17^.

#### 5. (E)-3-(benzo[d][1,3]dioxol-4-yl)-1–(4-bromophenyl)prop-2-en-1-one

Yellow crystals, yield = 55%, m.p. = 96–100 °C. ^1^H NMR (500 MHz, Chloroform-*d*) δ 7.92 (d, *J* = 8.3 Hz, 2H), 7.77 (d, *J* = 7.7 Hz, 2H), 7.67 (d, *J* = 8.5 Hz, 2H), 7.10–6.99 (m, 1H), 6.90 (d, *J* = 3.6 Hz, 2H), 6.15 (s, 2H). ^13 ^C NMR (176 MHz, CDCl3) δ 189.6, 148.1, 146.8, 139.9, 136.9, 131.9, 130.1, 127.9, 124.0, 123.6, 122.0, 117.9, 110.2, 101.6 EI-MS (*m*/*z*): calculated 331.9, observed 331.9 (M^+^).

#### 6. (E)-3-(benzo[d][1,3]dioxol-4-yl)-1–(4-chlorophenyl)prop-2-en-1-one

Yellow crystals, yield = 50%, m.p. = 83–87 °C. ^1^H NMR (500 MHz, Chloroform-*d*) δ 8.00 (d, *J* = 8.5 Hz, 2H), 7.75 (d, *J* = 4.3 Hz, 2H), 7.50 (d, *J* = 8.5 Hz, 2H), 7.04 (dq, *J* = 7.9, 4.0 Hz, 1H), 6.89 (d, *J* = 3.6 Hz, 2H), 6.15 (s, 2H). ^13 ^C NMR (176 MHz, CDCl_3_) δ 189.3, 148.0, 146.8, 139.8, 139.2, 136.5, 130.0, 128.4, 124.0, 123.6, 122.0, 117.9, 110.2, 101.6. EI-MS (*m*/*z*): calculated 286.0, observed 286.0 (M^+^).

#### 7. (E)-3-(benzo[d][1,3]dioxol-4-yl)-1–(3-bromophenyl)prop-2-en-1-one

Yellow crystals, yield = 67%, m.p. = 107–111 °C. ^1^H NMR (600 MHz, Chloroform-*d*) δ 8.17 (s, 1H), 7.98 (d, *J* = 7.0 Hz, 1H), 7.84–7.65 (m, 3H), 7.47–7.35 (m, 1H), 7.04 (s, 1H), 6.90 (s, 2H), 6.16 (d, *J* = 5.5 Hz, 2H). ^13 ^C NMR (150 MHz, CDCl_3_) δ 189.1, 148.0, 146.8, 140.1, 140.0, 135.6, 131.5, 130.2, 127.0, 123.9, 123.5, 122.9, 122.0, 117.7, 110.2, 101.6. EI-MS (*m*/*z*): calculated 331.9, observed 331.9 (M^+^).

#### 8. (E)-3-(benzo[d][1,3]dioxol-4-yl)-1–(3-chlorophenyl)prop-2-en-1-one

Yellow crystals, yield = 72%, m.p. = 79–83 °C. ^1^H NMR (700 MHz, Chloroform-*d*) δ 8.08–7.98 (m, 1H), 7.96–7.87 (m, 1H), 7.85–7.65 (m, 2H), 7.63–7.53 (m, 1H), 7.52–7.40 (m, 1H), 7.04 (q, *J* = 4.9 Hz, 1H), 6.91 (s, 2H), 6.16 (s, 2H). ^13 ^C NMR (176 MHz, CDCl_3_) δ 189.3, 148.1, 146.9, 140.2, 139.8, 134.9, 132.7, 130.0, 128.6, 126.6, 124.0, 123.6, 122.0, 117.8, 110.3, 101.6. EI-MS (*m*/*z*): calculated 286.0, observed 286.0 (M^+^).

#### 9. (E)-3–(4-bromophenyl)-1–(4-(methylsulfonyl)phenyl)prop-2-en-1-one

White crystals, yield = 71%, m.p. = 206–210 °C. ^1^H NMR (700 MHz, Chloroform-*d*) δ 8.18 (d, *J* = 7.7 Hz, 2H), 8.12 (d, *J* = 8.0 Hz, 2H), 7.80 (d, *J* = 15.9 Hz, 1H), 7.60 (d, *J* = 7.7 Hz, 2H), 7.55 (d, *J* = 8.1 Hz, 2H), 7.49 (d, *J* = 15.9 Hz, 1H), 3.13 (s, 3H). ^13 ^C NMR (176 MHz, CDCl_3_) δ 189.2, 145.3, 143.9, 142.3, 133.2, 132.4, 130.0, 129.3, 127.9, 125.6, 121.8, 44.4. EI-MS (*m*/*z*): calculated 364.9, observed 364.9 (M^+^).

#### 10. (E)-3–(4-bromo-2-methoxyphenyl)-1–(4-(methylsulfonyl)phenyl)prop-2-en-1-one

Yellow crystals, yield = 67%, m.p. = 199–203 °C. ^1^H NMR (700 MHz, Chloroform-*d*) δ 8.17 (d, *J* = 8.6 Hz, 2H), 8.11 (d, *J* = 7.2 Hz, 2H), 8.05 (d, *J* = 16.3 Hz, 1H), 7.57 (d, *J* = 15.6 Hz, 1H), 7.51 (d, *J* = 8.3 Hz, 1H), 7.19 (d, *J* = 8.1 Hz, 1H), 7.15–7.08 (m, 1H), 3.96 (s, 3H), 3.13 (s, 3H). ^13 ^C NMR (176 MHz, CDCl_3_) δ 189.9, 159.3, 143.6, 142.7, 141.1, 130.4, 129.3, 127.8, 126.3, 124.2, 122.5, 122.4, 115.1, 56.0, 44.4. EI-MS (*m*/*z*): calculated 395.9, observed 395.9 (M^+^).

#### 11. (E)-3–(3-bromophenyl)-1–(4-(methylthio)phenyl)prop-2-en-1-one

Yellow crystals, yield = 67%, m.p. = 139–143 °C. ^1^H NMR (700 MHz, Chloroform-*d*) δ 8.06–7.94 (m, 2H), 7.82 (s, 1H), 7.75 (d, *J* = 15.3 Hz, 1H), 7.64–7.47 (m, 3H), 7.41–7.23 (m, 3H), 2.57 (s, 3H). ^13 ^C NMR (176 MHz, CDCl_3_) δ 188.7, 146.1, 142.6, 137.1, 134.1, 133.2, 130.8, 130.5, 129.0, 127.3, 125.1, 123.1, 122.9, 14.8. EI-MS (*m*/*z*): calculated 333.9, observed 333.9 (M^+^).

#### 12. (E)-1–(4-fluorophenyl)-3–(4-nitrophenyl)prop-2-en-1-one

Yellow crystals, yield = 70%, m.p. = 165–169 °C. ^1^H NMR 400 MHz; CDCl_3_) δ (ppm): 8.27 (d, *J* = 8.7, 2H), 8.10 (dd, *J* = 8.7, 5.4 Hz, 2H), 7.81 (d, *J* = 15.8 Hz, 1H), 7.77 (d, *J* = 8.8 Hz, 2H),) , 7.60 (d, *J* = 15.7 Hz, 1H), 7.19 (d, *J* = 8.5 Hz 2H., ^13 ^C NMR (176 MHz, CDCl_3_) δ 188.0, 166.7, 165.2, 148.6, 141.8, 140.9, 133.9, 133.9, 131.3, 131.3, 129.0, 128.6, 125.2, 124.3, 123.9, 116.1, 116.0. EI-MS (*m*/*z*): calculated 271.0, observed 271.0 (M^+^).

#### 13. (E)-1–(4-chlorophenyl)-3–(4-nitrophenyl)prop-2-en-1-one

White crystals, yield = 61%, m.p. = 162–166 °C. ^1^H NMR (700 MHz, Chloroform-*d*) δ 8.37–8.28 (m, 2H), 8.15–7.96 (m, 2H), 7.96–7.78 (m, 3H), 7.64 (d, *J* = 15.8 Hz, 1H), 7.59–7.45 (m, 2H). ^13 ^C NMR (176 MHz, CDCl_3_) δ 188.3, 148.7, 142.0, 140.8, 139.9, 135.8, 130.0, 129.2, 129.0, 125.1, 124.3. EI-MS (*m*/*z*): calculated 287.0, observed 287.0 (M^+^).

#### 14. (E)-1-(benzo[d][1,3]dioxol-5-yl)-3–(4-fluorophenyl)prop-2-en-1-one

White crystals, yield = 58%, m.p. = 126–130 °C. ^1^H NMR (700 MHz, Chloroform-*d*) δ 7.80 (d, *J* = 15.4 Hz, 1H), 7.75–7.61 (m, 3H), 7.61–7.51 (m, 1H), 7.43 (d, *J* = 15.6 Hz, 1H), 7.20–7.07 (m, 2H), 6.93 (d, *J* = 8.4 Hz, 1H), 6.10 (s, 2H). ^13 ^C NMR (176 MHz, CDCl_3_) δ 188.0, 164.7, 163.3, 151.8, 148.3, 143.0, 132.9, 131.2, 131.2, 130.3, 130.3, 124.7, 121.4, 121.4, 116.2, 116.1, 108.4, 107.9, 101.9. EI-MS (*m*/*z*): calculated 270.0, observed 270.0 (M^+^).

#### 15. (E)-1-(benzo[d][1,3]dioxol-5-yl)-3–(4-bromophenyl)prop-2-en-1-one

White crystals, yield = 45%, m.p. = 150–153 °C. ^1^H NMR (500 MHz, Chloroform-*d*) δ 7.74 (d, *J* = 15.6 Hz, 1H), 7.66 (dd, *J* = 8.3, 2.1 Hz, 1H), 7.62–7.46 (m, 6H), 6.92 (d, *J* = 8.1 Hz, 1H), 6.09 (s, 2H). ^13 ^C NMR (176 MHz, CDCl_3_) δ 187.9, 151.9, 148.4, 142.8, 133.9, 132.8, 132.2, 129.7, 124.8, 124.7, 122.2, 108.4, 108.0, 102.0. EI-MS (*m*/*z*): calculated 331.9, observed 331.9 (M^+^).

#### 16. (E)-1-(benzo[d][1,3]dioxol-5-yl)-3–(4-chlorophenyl)prop-2-en-1-one

White crystals, yield = 67%, m.p. = 167–171 °C. ^1^H NMR (700 MHz, Chloroform-*d*) δ 7.76 (dd, *J* = 15.7, 3.6 Hz, 1H), 7.67 (d, *J* = 7.8 Hz, 1H), 7.62–7.53 (m, 3H), 7.48 (d, *J* = 14.9 Hz, 1H), 7.45–7.37 (m, 2H), 7.03–6.84 (m, 1H), 6.10 (s, 2H). ^13 ^C NMR (176 MHz, CDCl_3_) δ 187.9, 151.9, 148.4, 142.8, 136.3, 133.5, 132.8, 129.5, 129.2, 124.8, 122.1, 108.4, 108.0, 101.9.

EI-MS (*m*/*z*): calculated 286.0, observed 286.0 (M^+^).

#### 17. (E)-1-(benzo[d][1,3]dioxol-5-yl)-3–(4-nitrophenyl)prop-2-en-1-one

Yellow crystals, yield = 63%, m.p. = 172–175 °C. ^1^H NMR (700 MHz, Chloroform-*d*) δ 8.36–8.27 (m, 2H), 7.88–7.75 (m, 3H), 7.69 (d, *J* = 7.8 Hz, 1H), 7.66–7.59 (m, 1H), 7.56 (s, 1H), 6.94 (d, *J* = 8.4 Hz, 1H), 6.11 (d, *J* = 3.8 Hz, 2H). ^13 ^C NMR (176 MHz, CDCl_3_) δ 187.4, 152.3, 148.5, 148.5, 141.2, 141.0, 132.3, 128.9, 126.6, 125.5, 125.1, 124.2, 123.8, 108.4, 108.1, 102.1. EI-MS (*m*/*z*): calculated 297.0, observed 297.0 (M^+^).

#### 18. (E)-1–(4-bromophenyl)-3–(2,4,6-trimethoxyphenyl)prop-2-en-1-one

Yellow crystals, yield = 64%, m.p. = 148–152 °C. ^1^H NMR 400 MHz; CDCl_3_) δ (ppm): 8.25 (d, *J* = 15.8 Hz, 1H), 7.87 (d, *J* = 8.4 Hz, 2H), 7.80 (d, *J* = 15.9 Hz, 1H), 7.61 (d, *J* = 8.4 Hz, 2H), 6.14 (s, 2H), 3.91 (s, 6H, 2 OCH_3_), 3.87 (s, 3H, OCH_3_). ^13 ^C NMR (100 MHz; DCl_3_)δ: 191.1, 163.4, 161.9, 138.1, 136.7, 131.6, 130.1, 121.4, 106.5, 90.6, 55.9, 55.4. EI-MS (*m*/*z*): calculated 378.0, observed 378.0 (M^+^).

#### 19. (E)-1–(4-fluorophenyl)-3–(2,4,6-trimethoxyphenyl)prop-2-en-1-one

Yellow crystals, yield = 66%, m.p. = 146–150 °C. ^1^H NMR (700 MHz, Chloroform-*d*) δ 8.30 (d, *J* = 15.8 Hz, 1H), 8.14–8.00 (m, 2H), 7.88 (d, *J* = 15.7 Hz, 1H), 7.17 (d, *J* = 8.7 Hz, 2H), 6.16 (s, 2H), 3.93 (s, 6H), 3.89 (s, 3H). ^13 ^C NMR (176 MHz, CDCl_3_) δ 190.6, 165.9, 164.5, 163.2, 161.8, 136.3, 135.6, 135.6, 131.0, 130.9, 121.4, 115.4, 115.3, 106.4, 90.5, 55.4, 55.4. EI-MS (*m*/*z*): calculated 316.1, observed 316.1 (M^+^).

#### 20. (E)-1–(4-bromophenyl)-3–(2-methoxyphenyl)prop-2-en-1-one

White crystals, yield = 60%, m.p. = 73–77 °C. ^1^H NMR (700 MHz, Chloroform-*d*) δ 8.14 (d, *J* = 15.8 Hz, 1H), 7.95–7.86 (m, 2H), 7.70–7.62 (m, 3H), 7.58 (d, *J* = 16.0 Hz, 1H), 7.48–7.36 (m, 1H), 7.06–7.00 (m, 1H), 7.00–6.94 (m, 1H), 3.95 (s, 3H). ^13 ^C NMR (176 MHz, CDCl_3_) δ 190.1, 158.9, 141.1, 137.2, 132.0, 131.8, 130.1, 129.4, 127.6, 123.7, 122.3, 120.8, 111.3. EI-MS (*m*/*z*): calculated 318.0, observed 318.0 (M^+^).

#### 21. (E)-1–(4-bromophenyl)-3–(2,3-dimethoxyphenyl)prop-2-en-1-one

White crystals, yield = 64%, m.p. = 120–124 °C. ^1^H NMR (700 MHz, Chloroform-*d*) δ 8.13 (d, *J* = 16.0 Hz, 1H), 7.95–7.87 (m, 2H), 7.66 (d, *J* = 6.9 Hz, 2H), 7.57 (d, *J* = 16.1 Hz, 1H), 7.34–7.25 (m, 1H), 7.17–7.08 (m, 1H), 7.01 (d, *J* = 7.5 Hz, 1H), 3.97–3.86 (m, 6H). ^13 ^C NMR (176 MHz, CDCl_3_) δ 189.8, 153.2, 149.0, 140.3, 137.0, 131.9, 130.1, 128.9, 127.8, 124.3, 123.0, 119.6, 114.4, 61.4, 55.9. EI-MS (*m*/*z*): calculated 348.0, observed 348.0 (M^+^).

#### 22. (E)-1–(4-bromophenyl)-3–(2,3-diethoxyphenyl)prop-2-en-1-one

White crystals, yield = 50%, m.p. = 75–79 °C. ^1^H NMR (700 MHz, Chloroform-*d*) δ 8.15 (d, *J* = 16.1 Hz, 1H), 7.91 (d, *J* = 6.8 Hz, 2H), 7.72–7.62 (m, 2H), 7.56 (d, *J* = 15.6 Hz, 1H), 7.28 (d, *J* = 7.9 Hz, 1H), 7.14–7.03 (m, 1H), 6.98 (d, *J* = 7.6 Hz, 1H), 4.20–4.06 (m, 4H), 1.50 (d, *J* = 6.5 Hz, 3H), 1.42 (t, *J* = 6.7 Hz, 3H). ^13 ^C NMR (176 MHz, CDCl_3_) δ 189.9, 152.6, 148.3, 141.0, 137.1, 131.9, 130.1, 129.2, 127.7, 124.0, 122.8, 119.5, 115.4, 69.6, 64.4, 15.8, 14.91. EI-MS (*m*/*z*): calculated 376.0, observed 376.0 (M^+^). The detailed MS and NMR spectra are provided as Supporting information data.

### Enzymatic screening

#### Screening for the inhibition of MAO

All prepared compounds were screened against both human MAO-A and MAO-B to assess their inhibitory activities. This was done spectrophotometrically using MMTP as a nonselective substrate. MMTP is oxidized by MAO to yield the corresponding MMDP^+^ species, which absorbs maximally at 420 nm. DMSO was used as a co-solvent at 3.3% (v/v) final concentration, which does not affect MAO activity. DMSO was included since the test compounds exhibit limited aqueous solubility.

Each assay was performed in a quartz cuvette (150 µL final volume). A mixture composed of 5 µL enzyme (25 µg of MAO-A or MAO-B), 95 µL buffer (0.1 M potassium phosphate buffer), and 5 µL test compound (10 µM final concentration) was incubated at 37 °C for 5 min. The reactions were initiated by adding 45 µL MMTP at a final concentration of 0.15 mM. The increase in absorbance was monitored at 37 °C and recorded at 420 nm. The enzyme initial velocity (*v*_0_) was determined by using the extinction coefficient of 25000 M^−1 ^cm^−1^ for the dihydropyridinium species. The inhibitory activity of the prepared compounds was determined as the ratio between the obtained v_0_ value for each chalcone analog and that of the control experiment containing no inhibitor (100% activity). Control experiments were prepared by following the same procedure except for the addition of 5 µL DMSO instead of the test compounds. Compounds that showed more than 60% enzyme inhibition were subjected to IC_50_ determination.

#### Determination of IC_50_ values for MAO inhibition

IC_50_ values for the inhibition of MAO were determined by using the recombinant human MAO-A and MAO-B enzymes (Sigma-Aldrich). Enzyme reactions were performed at a final volume of 200 µL in white 96-well microplates (Eppendorf), with the reaction solvent consisting of potassium phosphate buffer (100 mM, pH 7.4, adjusted to isotonicity with KCl). The reactions contained the nonselective MAO substrate, kynuramine (50 μM), and the test inhibitors at 0.003–100 µM. The test compounds were initially dissolved in DMSO (100%), and after addition to the reactions yielded a concentration of 4% DMSO. Control reactions were included, which contained substrate and 4% DMSO but no test inhibitor. The MAO reactions were started by adding MAO-A (0.0075 mg protein/mL) or MAO-B (0.015 mg protein/mL), and were subsequently incubated for a period of 20 min (37 °C) in a convection oven. A volume of 80 µL 2 N sodium hydroxide was added to the reactions, and the fluorescence intensity of 4-hydroxyquinoline (the end-product of the MAO-catalyzed metabolism of kynuramine) in the reactions was measured at *λ*_ex_ 310 and *λ*_em_ 400 nm. To quantify 4-hydroxyquinoline, a calibration curve (linear) ranging from 0.047 to 1.56 μM, was employed. From these data, the rates of 4-hydroxyquinoline formation were determined, sigmoidal plots of activity versus inhibitor concentration [Log(I)] were prepared, and the IC_50_ values were recorded (Prism 5, GraphPad). These measurements were carried out in triplicate and the IC_50_ values are presented as the mean ± standard deviation (*SD*)^18^.

#### Reversibility of MAO-B inhibition

The reversibility of inhibition of MAO by compound **16** was investigated by dialysis. Dialysis cassettes (Slide-A-Lyzer^®^; Thermo Scientific) with a volume of 0.5–3 mL and a molecular weight cutoff of 10,000 were used for the dialysis. Mixtures of MAO-A or MAO-B (0.03 mg protein/mL) and the test inhibitor (concentration = 4 × IC_50_), were prepared in potassium phosphate buffer (100 mM, pH 7.4) with 5% sucrose added. The volume of these mixtures was 0.8 mL, and contained 4% DMSO. The mixtures were pre-incubated for 15 min at 37 °C and then dialyzed at 4 °C in buffer (80 mL), using the above buffer as dialysis buffer. After 3 and 7 h of dialysis, the dialysis buffer was changed with fresh buffer. Controls were prepared in a similar way, MAO-A and MAO-B were pre-incubated without inhibitor present (negative control) and with irreversible MAO inhibitors [pargyline (MAO-A) and (R)-deprenyl (MAO-B)] as positive controls. Pargyline [IC_50_ =13 μM for MAO-A]^19^ and (R)-deprenyl [IC_50_ = 0.079 μM for MAO-B]^20^ were used at concentrations equal to fourfold their IC_50_ values. After 24 h of dialysis, samples of the dialysis mixtures (250 μL) were diluted twofold by adding kynuramine (250 μL) to yield a concentration of 2 × IC_50_ for the inhibitor and 50 µM for kynuramine. After incubation of these enzyme reactions for 20 min at 37 °C, the remaining MAO activities were subsequently recorded by fluorescence spectrophotometry as described above for the IC_50_ value determination. For comparison, incubations of the MAOs and the test inhibitor were prepared as above but were not dialyzed and kept at 4 °C over the same time period. These experiments were carried out in triplicate, and the remaining enzyme catalytic rates are reported as the mean (± *SD*).

#### Lineweaver–Burk plots and *K_i_* value calculations

Sets of six Lineweaver–Burk plots each were prepared to determine if the inhibition of MAO-A and MAO-B by compound **16** is competitive. For the first plot, MAO activity was recorded without inhibitor present; while for the other five plots, MAO activity was recorded at inhibitor concentration of ^1^/_4_ × IC_50_, ^1^/_2_ × IC_50_, ^3^/_4_ × IC_50_, 1 × IC_50_, and 1^1^_/4_ × IC_50_. The substrate, kynuramine, was used at concentrations ranging from 15 to 250 μM, whereas the final enzyme concentration was 0.015 mg protein/mL. All incubations were performed at a volume of 500 μL and the MAO activities were determined by fluorescence spectrophotometry as described for the IC_50_ value determination. *K_i_* values were estimated by graphing the slopes of the Lineweaver–Burk plots against inhibitor concentration. The intercept of the *x*-axis is equal to –*K_i_*.

### Molecular docking studies

To gain a better understanding of inhibitor–enzyme interactions at the molecular level, and to explain the structural requirements for MAO inhibition activity of the synthesized compounds, molecular docking was carried out using Discovery Studio’s CDOCKER^21^. The module uses a CHARMm-based molecular dynamics algorithm to generate random ligand conformations. The conformations are then docked into the binding site using random rigid-body rotations and simulated annealing, followed by a final minimization step to refine the ligand poses. The X-ray crystal structure of human MAO-B in complex with safinamide (code: 2V5Z)^22^ and human MAO-A in complex with harmine (code: 2Z5X)^23^ were downloaded from the PDB. Both structures were prepared by removing the co-crystalized ligand and water molecules, fixing the missing residues and adding hydrogen atoms and partial charges. As in previous docking protocols reported by our group, we performed molecular docking in the presence of preserved water molecules in proximity of the FAD, which seem important for mediating interactions with the inhibitors^11^. Furthermore, to determine the binding site, a sphere with a radius of 13Å was centered on the co-crystalized safinamide in human MAO-B or harmine in human MAO-A. Default CDOCKER parameters were used and each docking run returned ten poses, which were clustered and visually inspected and the top-ranked pose in the largest cluster was chosen. To assess the ability of CDOCKER and to predict the native binding pose, co-crystalized safinamide and harmine were re-docked in the binding site of human MAO-B (2V5Z) and MAO-A (2Z5X), respectively, starting from a random conformation. The docked poses were compared with the orientation of co-crystalized ligand. In addition, crossdocking was performed: various co-crystalized MAO-B ligands were docked into the binding pocket of human MAO-B (2V5Z) and *RMSD* (Root Mean Square Deviation) values against their original crystalized poses were measured after superimposing the different crystal structures. All synthesized compounds were drawn and prepared by adding hydrogen atoms and partial charges using the Discovery Studio software. All single bonds of the compounds were considered rotatable during the docking runs where residues of the binding site were considered rigid. Visualization of key binding pocket residues and docked poses were performed using UCSF Chimera^24^.

### Molecular mechanism investigation

#### H4IIE cell culture and dopamine assay

Rat hepatoma (H4IIE) cells were purchased from American Type Culture Collection (ATCC). H4IIE cells are from rat liver, and contain both MAO-A and MAO-B in approximately equal levels. Cells were cultured in Eagle’s minimum essential medium (EMEM) supplemented with 10% fetal bovine serum (FBS) and 1% streptomycin/penicillin at 37 °C in a 5% CO_2_ humidified incubator. Cells were plated at a cell density of 3.0 × 10^5^ cells in each well of six well plates. Upon 80% confluence, the cells were washed with phosphate buffered saline (PBS) and the media were changed to HBSS supplemented with 10 mM HEPES and 5 mM d-glucose, pH 7.4. These media also contained calcium chloride (1.26 mM) and magnesium chloride (0.5 mM). Cells were then treated with tolcapone (1 0^−6 ^M), a catechol-O-methyltransferase (COMT) inhibitor to protect dopamine from enzymatic degradation, 20 min prior to starting the experiment. The cells were treated with the negative vehicle control (1% DMSO) or 70 µM of the MAO-B inhibitors (compounds **12**, **14**, **16,** and **17**) with the DMSO concentration also being 1%. The cells were subsequently treated with L-DOPA (50 µM). To measure dopamine release and MAO-B gene expression, both the cells and media were collected at 0, 30, 60, and 120 min after treatment. Dopamine level was measured in the supernatant aspirated from the cells by using high performance liquid chromatography (HPLC), while the cells were lysed with trizol for RNA extraction and subsequent gene expression studies.

#### Quantitative real-time polymerase chain reaction

Total RNA was isolated using the Trizol agent (Invitrogen). One μg of RNA from each sample was reverse transcribed to cDNA using the M-MLV reverse transcriptase kit, according to the manufacturer’s instruction (Promega). PowerUp SYBR green master mix (Life technologies) was used for quantitative real-time polymerase chain reaction (qRT-PCR) for analysis of gene expression. The following primers were used (IDTs) MAO-B forward, ACCAGTGTGGAATCCAATCACTTA; MAO-B reverse, TTGTCATGTAGTCCCACTCTTCAG; *β-actin* forward, GCGAGTACAACCTTCTTGCAGCTC and *β-actin* reverse, AGCGCAGCGATATCGTCATCCAT. qRT-PCR was performed in a 384 well plates with the Quant Studio-5 instrument (Applied Biosystems). Samples were carried out in triplicate in a 10 µL reaction volume using the following cycling conditions: UDG activation for one cycle at 50 °C for 2 min, followed by a single cycle at 95 °C for 2 min, then 40 cycles of denaturation at 95 °C for 15 s, and annealing at 60 °C for 1 min. *β-actin* was used as a loading control and gene expression was calculated using the 2^-ΔΔCT^ method as described previously^25^.

#### High performance liquid chromatography with electrochemical detection

Rat hepatoma cells (H4IIE) were treated with 1% DMSO (vehicle-control), compounds **12**, **14**, **16**, or **17** for different time points at a concentration of 70 µM of each compound along with 50uM L-DOPA.

The concentrations of dopamine were detected using a CoulArray electrochemical array detector system as described previously^24^. Briefly, supernatants obtained from cell culture were filtered using 0.22 μm filter. The filtrates were subsequently injected onto a C18 column (3.2 × 150 mm, 3 μm particle size, Thermo Scientific). The mobile phase consisted of a mixture of 54.3 mM sodium phosphate, 0.215 mM octyl sodium sulfate, 0.32 mM citric acid and 11% methanol. Dopamine concentrations were measured with the CoulArray Electrochemical Array Detector. The chromatograms were analyzed using the CoulArray software. A standard calibration curve was determined using different concentrations of an external standard dopamine (SigmaAldrich).

#### Statistical analysis

One-way ANOVA followed by Dunnett’s test (compared to the vehicle-control) was used to analyze the data obtained for the dopamine concentrations for compounds **12**, **14**, **16,** and **17**targeting MAO-B at 0, 30, 60, and 120 min. One-way ANOVA followed by Newman–Keuls multiple comparison tests was used to analyze time-dependent effects of compounds **14** and **17** on the relative mRNA expression (triplicate) of MAO-B enzyme.

## Results and discussion

### Chemistry

**Scheme 1.**
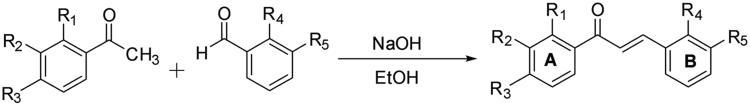
Synthetic route to the proposed chalcone analogs.

**Figure 2.**
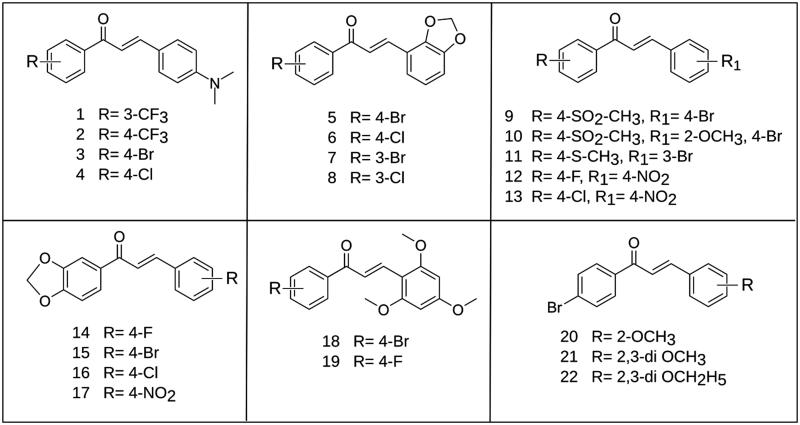
Structures of the synthesized compounds **1–22**.

The 22 chalcone analogs (Figure 2) were designed and prepared in moderate to good yields by Claisen–Schmidt condensation reaction. The synthesis was achieved by the reaction of equimolar amounts of the substituted acetophenones and appropriate benzaldehydes in ethanol and in presence of three equivalent of NaOH (in the minimum amount of water) as shown in Scheme 1.

**Figure 3.**
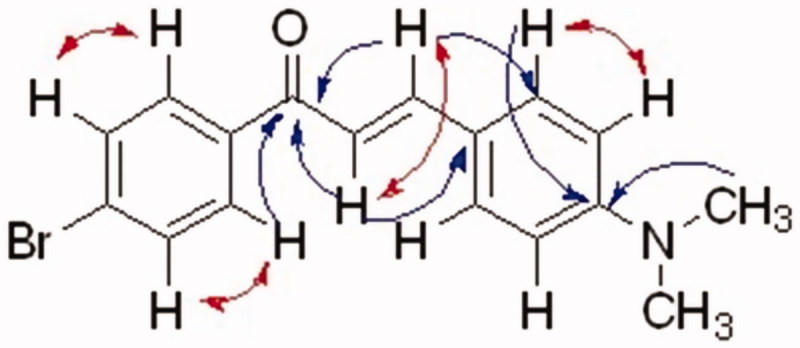
Key COSY (in red) and HMBC (in blue) correlations of compound **4**.

The crude products were collected by filtration and crystallization from ethanol. The structures of the prepared compounds were elucidated by various spectroscopic techniques (e.g. FT-IR, GC-MS, and NMR). The chemical shifts of all protons and carbons of compound **4** were fully determined and assigned by 2D-NMR (COSY, HSQC, and HMBC; Figure 3). The *trans* configuration of the olefinic protons was confirmed by the large coupling constant (*J* = 16 Hz).

### Structure–activity relationships of the prepared analogs

The synthesized compounds were initially screened at 10 μM final concentration against recombinant human MAO-A and MAO-B by using 1-methyl-4–(1-methylpyrrol-2-yl)-1,2,3,6-tetrahydropyridine (MMTP) as a nonselective substrate for both MAO-A and B. It is important to note that the *K*_m_ values for human MAO-A and MAO-B were found to be 88 μM and 101 μM, respectively, and this is in accordance with previously reported values^26^^,^^27^. The formation of the dihydropyridinium species (MMDP^+^) as a MAO-catalyzed oxidation product of MMTP was monitored at 420 nm. The results showed that most of the chalcones, indeed, have a marked inhibitory effect on the MAO enzymes at this concentration. Based on this, the IC_50_ values were determined using kynuramine as a nonselective substrate. The rate of oxidation of kynuramine by MAO was monitored by measuring the fluorescence of the oxidation product, 4-hydroxyquinoline (*λ*_ex_= 310 nm; *λ*_em_= 400 nm). Kynuramine and the chalcone analogs did not interfere with the measurement, as these drugs did not show any fluorescence.

**Figure 4.**
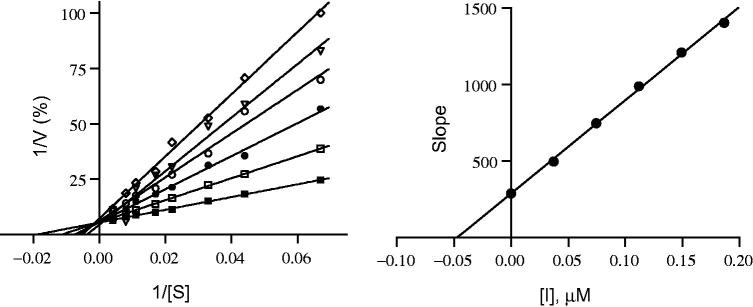
Lineweaver–Burk plots for hMAO-A inhibition by compound **16** (left). The slopes of the Lineweaver–Burk plots were graphed versus inhibitor concentration (right).

**Figure 5.**
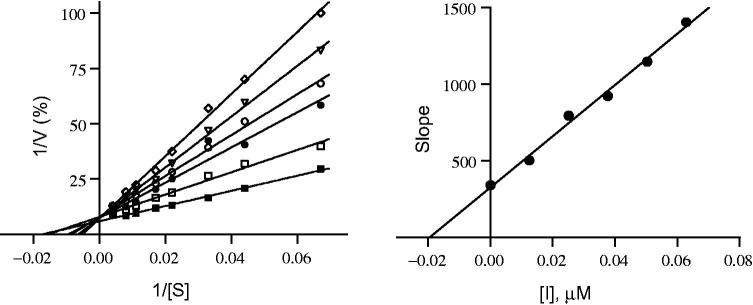
Lineweaver–Burk plots for hMAO-B inhibition by compound **16** (left). The slopes of the Lineweaver–Burk plots were graphed versus inhibitor concentration (right).

The IC_50_ values were determined for all the synthesized compounds against recombinant human MAO-A and B to determine their activities and selectivity (Table 1). Analogs that bear the –N(CH_3_)_2_ group at position 4 of the B aromatic ring and halogens at positions 3 or 4 on the A aromatic ring showed high inhibitory activity and significant selectivity for MAO-B (compounds **1–4**) with IC_50_ values ranged from 0.123 to 0.539 μM. Analogs with the methylenedioxy ring on the B aromatic ring and a halogen at position 3 or 4 on the A ring (compounds **5–8**) showed even higher activity against human MAO-B with IC_50_ values ranged from 0.058 to 0.139 μΜ. Compound **8**, with a Cl substituent at position 3 of the A ring, was most noteworthy with respect to both activity and selectivity in this group with an IC_50_ ranged of 58 nM for human MAO-B and >100 μM for MAO-A. Several other analogs were prepared with –SO_2_-CH_3_, -S-CH_3_, F, or Cl substituents on position 4 of the A ring and Br or NO_2_ on positions 3 or 4 of the B ring (compounds **9–13**). Among these, compound **13** showed the highest activity with an IC_50_ value of 34 nM. Interestingly, the introduction of 2,4,6-trimethoxy substituents on the B aromatic ring shifted the selectivity toward MAO-A. The IC_50_ values for compounds **18** and **19** were found to be >100 (MAO-B) and 0.56 and 0.444 μM (MAO-A), respectively. Compound **16** was selected for the determination of the inhibitor constants (*K_i_*) for human MAO-A and MAO-B inhibition. For these measurements, Lineweaver–Burk plots were prepared for the inhibition of human MAO-A and MAO-B by compound **16**. The Lineweaver–Burk plots for both human MAO isoforms yielded linear lines that cross on the *y*-axis as shown in Figures 4 and 5, respectively. This suggests that the inhibition of both human MAO-A and MAO-B by compound **16** is competitive. By graphing the slopes of the Lineweaver–Burk plots versus the concentration of the inhibitor, *K_i_* values of 0.047 and 0.020 µM for human MAO-A and MAO-B inhibition, respectively, were estimated.

**Table 1.** The IC_50_ values and selectivity indices for recombinant hMAO-A and B inhibition by the chalcones.

Number	Structure	MAO-B IC_50_ (µM ± *SD*)^a^	MAO-A IC_50_ (µM ± *SD*)^a^	Selectivity index^b^
1		0.123 ± 0.003	14.3 ± 1.81	116
2		0.383 ± 0.061	> 100 µM	>260
3		0.539 ± 0.058	> 100 µM	>185
4		0.321 ± 0.023	> 100 µM	>310
5		0.106 ± 0.012	7.18 ± 0.755	67
6		0.139 ± 0.019	18.1 ± 3.49	130
7		0.081 ± 0.012	> 100 µM	>1230
8		0.058 ± 0.009	> 100 µM	>1720
9		0.404 ± 0.064	> 100 µM	>240
10		0.582 ± 0.030	87.3 ± 9.34	150
11		0.198 ± 0.012	96.6 ± 5.21	487
12		0.069 ± 0.006	11.9 ± 0.822	172
13		0.034 ± 0.006	45.5 ± 2.93	>1338
14		0.043 ± 0.004	0.506 ± 0.052	11
15		0.102 ± 0.009	0.173 ± 0.003	1.7
16		0.050 ± 0.002	0.149 ± 0.011	3.0
17		0.069 ± 0.004	0.927 ± 0.121	13
18		> 100 µM	0.560 ± 0.076	<0.005
19		> 100 µM	0.444 ± 0.039	<0.004
20		0.999 ± 0.276	5.81 ± 0.878	5.8
21		0.174 ± 0.015	6.59 ± 0.422	37
22		15.6 ± 0.803	> 100	6.4

a The inhibition data are given as the mean ± *SD* (triplicate).

b Selectivity index (SI) = IC_50_(MAO-A)/IC_50_(MAO-B).

### Reversibility of inhibition of human MAO-A and MAO-B

**Figure 6.**
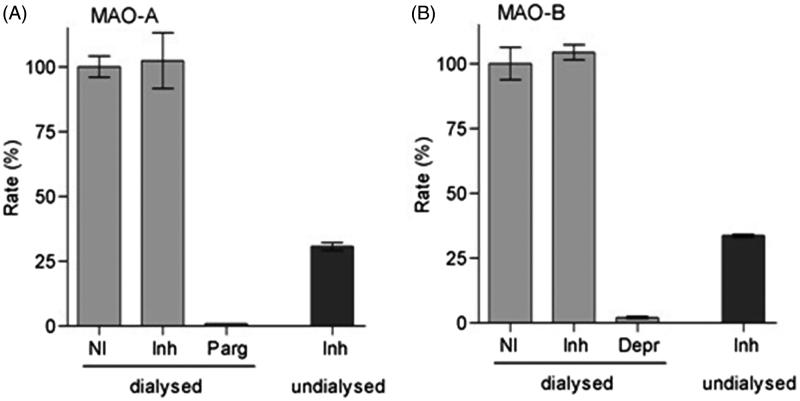
Reversibility of inhibition of hMAO-A (panel A) and hMAO-B (panel B) by compound **16**. A mixture of the enzyme and compound **16** (at a concentration of 4 × IC_50_) was incubated (15 min) and dialyzed (24 h), and the remaining enzyme activity was determined (Inh dialyzed). The enzyme was similarly incubated and dialyzed with no inhibitor present (NI dialyzed), as well as in the presence of pargyline (parg dialyzed) or (R)-deprenyl (Depr dialyzed), both irreversible MAO inhibitors. The residual activity of mixtures of the enzyme and compound **16** that were not subjected to dialysis was also recorded (Inh undialyzed).

Compound **16** was selected as a representative compound for determining the reversibility of MAO inhibition since it showed potent nonselective inhibitory activity against both human MAO-A and MAO-B with IC_50_ values equal to 0.149 and 0.050 μM, respectively. This experiment aims to determine the recovery of enzyme activity after incubation of dialyzed mixtures containing the enzyme and inhibitor. To evaluate reversibility, compound **16** (at a concentration of 4 × IC_50_) was incubated with human MAO-A and MAO-B (15 min) and was then dialyzed (24 h). The MAO activities were determined and compared with the activities of the control samples which constituted of a negative control with no inhibitor present, and a positive control where irreversible MAO inhibitors, pargyline and (R)-deprenyl, are present. As indicated in Figure 6, compound **16** reversibly inhibits MAO-A and MAO-B as catalytic activity of both isoforms is recovered by dialysis, with the activities at 103 and 105%, respectively, compared to the negative control value (100%). In contrast, dialysis failed to restore catalytic activity when MAO-A and MAO-B were incubated in the presence of pargyline and (R)-deprenyl, respectively. For comparison, inhibition of MAO-A and MAO-B by compound **16** persists in undialyzed samples with the activities at 31% and 34%, respectively.

### Molecular docking studies

In general, CDOCKER was able to predict the native binding pose of different MAO-B ligands with a high degree of accuracy. Table 2 shows the calculated *RMSD* values between the docked and the co-crystalized ligands in absence and presence of water molecules in the binding pocket. Harmine was redocked in the active site of human MAO-A with *RMSD* of 0.848Å.

**Table 2.** Calculated *RMSD* values between the docked and the co-crystalized MAO-B ligands in absence and presence of water molecules in the binding pocket.

Inhibitor	PDB code	*RMSD* (Å)	
		water	no water
Safinamide	2V5Z^2^	0.1925	0.1628
Farnesol	2BK3^5^	1.6910	2.0833
7-[(3-Chlorobenzyl)oxy]-2-oxo-2*H*-chromene-4-carbaldehyde	2V60^2^	0.4694	0.7538
7-[(3-Chlorobenzyl)oxy]-4-[(methylamino)methyl]-2*H*-chromen-2-one	2V61^2^	1.2469	8.5715
Zonisamide	3PO7^6^	1.2238	2.4251
Rosiglitazone	4A7A^7^	1.8739	2.6105
Pioglitazone	4A79^7^	3.8394	4.0468

The PDB codes of the crystal structures from which ligands were extracted are indicated. Docking was performed with the protein structure 2V5Z.

**Figure 7.**
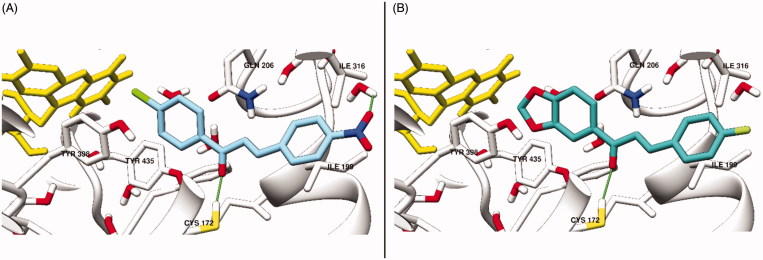
The active site of hMAO-B showing the docked poses of compound **13** (A) and compound **16** (B) with Ile199 rotated into the open conformation. A hydrogen bond between the carbonyl oxygen and the SH of Cys172 is shown as a green line. In addition, the nitro group forms a hydrogen bond with one of the binding site water molecules. Key binding site residues are shown in stick representation.

It is observed that retaining the conserved water molecules leads to lower *RMSD* values for the co-crystalized ligands that were re-docked, indicating a higher capability of predicting the native binding mode. This could be attributed to the reduced binding site size and hence the space available for possible docked poses, however, the persistence of these water molecules in all MAO-B crystal structures indicates that their displacement is unfavorable, and this case can be generalized to our synthesized compounds^8^^,^^9^. In the human MAO-B binding pocket, residues Tyr398 and Tyr435, together with FAD, form an aromatic cage, while Ile199 plays a key role as a gating residue between the entrance and substrate-binding cavities by occupying either a closed or an open conformation based on the length of the inhibitors. The docking study showed that the aromatic amino acids (Tyr398 and Tyr435) of the binding site accommodate the electron-deficient aromatic rings of chalcones. In all poses, the carbonyl oxygen anchors the inhibitors to the bottom of the binding site by forming a hydrogen bond with Cys172 as shown in Figure 7.

### Molecular mechanism investigations

#### Effects of compounds 12, 14, 16, and 17 on dopamine concentrations (ng/mL) at 0, 30, 60, and 120 min

**Figure 8.**
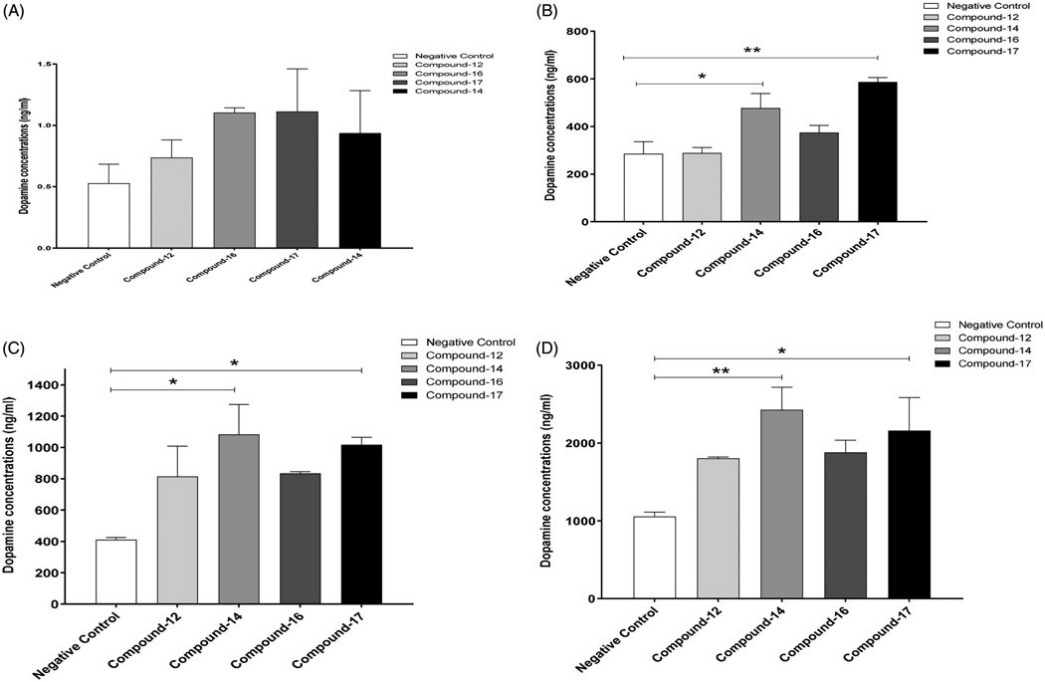
Effects of compounds **12, 14, 16**, and **17** on dopamine concentrations in rat hepatocytes. (A) One way ANOVA followed by Dunnett’s test showed no significant changes in dopamine concentrations between negative control and the selected compounds at 0 min. (B) One way ANOVA followed by Dunnett’s test showed a significant increase in dopamine concentrations with compounds **14** and **17** compared to negative control at 30 min. (C) One way ANOVA followed by Dunnett’s test showed a significant increase in dopamine concentrations with compounds **14** and **17** as compared to negative control at 60 min. D. One way ANOVA followed by Dunnett’s test showed a significant increase in dopamine concentrations with compounds **14** and **17** compared to negative control at 120 min. Data are shown as mean ± *SEM* (*p < .05, **p < .01).

We assessed the concentrations of dopamine in rat hepatoma (H4IIE) cells treated with 1% DMSO (vehicle-control), and compounds **12, 14, 16,** and **17** at 0, 30, 60, and 120 min. One way ANOVA followed by Dunnett’s test did not show any significant changes in dopamine concentrations between the compounds and vehicle-control groups at 0 min (Figure 8(A)). However, One-way ANOVA showed significant differences in the dopamine concentrations among the groups at 30 min [*F*(4, 10) = 10.26863, *p* < .01, (Figure 8(B))]. One-way ANOVA followed by Dunnett’s test revealed that treatment with compounds **14** and **17** increased the concentrations of dopamine compared to the vehicle control group at 30 min. We further investigated the effects of the selected compounds on the dopamine concentrations at 60 min. One-way ANOVA revealed significant changes in the dopamine concentrations among the groups at 60 min [*F*(4, 10) = 4.477, *p* < .05, (Figure 8(C))]. One-way ANOVA followed by Dunnett’s test showed that compounds **14** and **17** caused elevation in the dopamine concentration when compared to the vehicle-control group. Moreover, one-way ANOVA revealed a significant difference at 120 min among groups [*F*(4, 10) = 4.544, *p* < .05, (Figure 8(D))]. One way ANOVA followed by Dunnett’s test indicates that both compounds **14** and **17** were able to increase the dopamine levels at 120 min. These data indicate that compounds **14** and **17** have rapid onset on the elevation of dopamine levels using H4IIE cells. Other compounds showed a positive trend (but not significant) on dopamine levels, and these compounds may be effective to increase the dopamine concentrations when exposed for a longer duration.

#### Effects of compounds 14 and 17 on the relative mRNA expression of the MAO-B enzyme at 0, 30, 60 and 120 min

**Figure 9.**
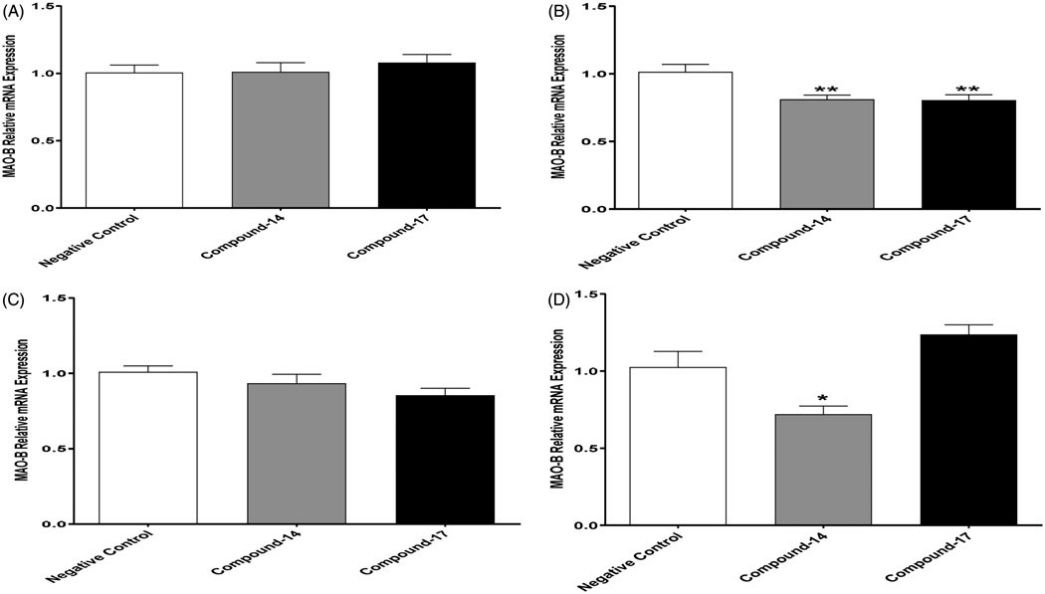
Effects of compounds **14** and **17** on MAO-B mRNA expression in rat hepatocytes. (A) One way ANOVA followed by Dunnett’s test showed no significant changes in MAO-B mRNA expression between negative control and compounds **14** and **17** at 0 min. (B) One way ANOVA followed by Dunnett’s test showed a significant decrease in MAO-B mRNA expression with compound **14** and **17** compared to negative control at 30 min. (C) One way ANOVA followed by Dunnett’s test showed no significant changes in MAO-B mRNA expression with compounds **14** and **17** as compared to negative control at 60 min. (D) One way ANOVA followed by Dunnett’s test showed a significant decrease in MAO-B mRNA expression with compound **14** compared to negative control at 120 min. Data are shown as mean ± *SEM* (*p < .05, **p < .01).

We further investigated the effects of compounds **14** and **17**, which are shown above to increase dopamine concentrations, on the relative mRNA expression of MAO-B at different time points. One way ANOVA followed by Dunnett’s test did not show any differences in the mRNA expression of MAO-B between the test compounds and negative control groups at 0 min (Figure 9(A)). However, the statistical analysis showed a significant change in the relative mRNA expression of MAO-B among groups at 30 min [*F*(2, 24) = 7.210870, *p* < .01, (Figure 9(B))]. One-way ANOVA followed by Dunnett’s test revealed that both compounds, **14** and **17**, reduced the relative expression of MAO-B mRNA at 30 min compared to the negative control. Surprisingly, one-way ANOVA followed by Dunnett’s test did not show any significant changes in the MAO-B mRNA expression between the compounds and negative control at 60 min (Figure 9(C)). However, one-way ANOVA showed significant differences in MAO-B mRNA expression among groups at 120 min [*F*(2, 21) = 15.62921, *p* < .0001, (Figure 9(D))]. One-way ANOVA followed by Dunnett’s test showed that only compound **14** decreased the relative mRNA expression compared to the negative control at 120 min. The data confirm that compound **17** has a short duration on reducing MAO-B mRNA expression since this compound only reduced mRNA expression of the enzyme at 30 min as compared to negative control. This may suggest a compensatory mechanism for the reduction of mRNA expression of MAO-B enzyme that has been occurred after 30 min from the compound exposure. Interestingly, compound **14** was able to reduce MAO-B mRNA expression in H4IIE cells at 30 and 120 min time points but not 60 min. We suggest here that higher concentrations might be required for the compound to exhibit stable reduction effects on the MAO-B mRNA expression in H4IIE cells.

#### Time-dependent effects of compounds 14 and 17 on the relative mRNA expression of MAO-B enzyme

**Figure 10.**
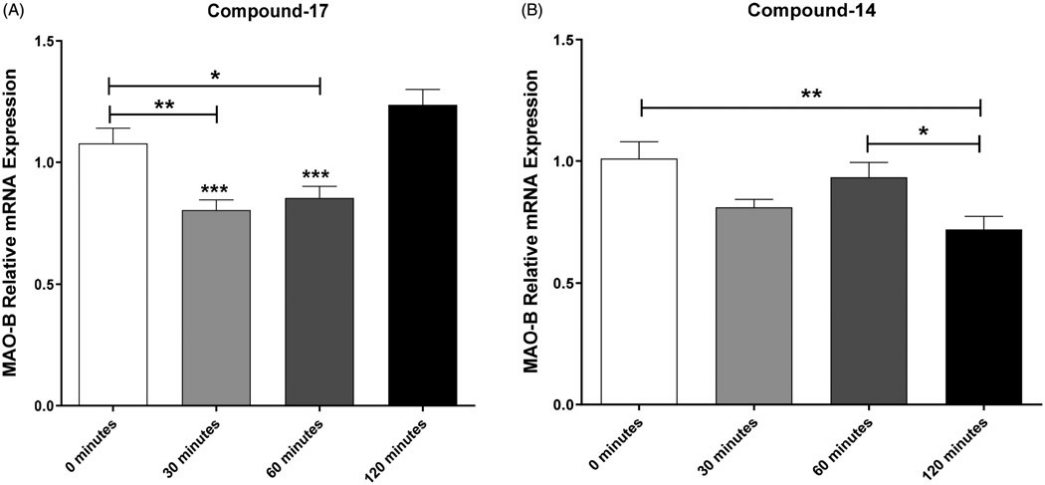
Time-dependent effects of compounds **14** and **17** on the relative mRNA expression of MAO-B. (A) One way ANOVA followed by Newman–Keuls multiple comparison test showed a significant decrease in MAO-B mRNA expression after 30 and 60 min exposure to compound **17** as compared to 0 min exposure (*p < .05,**p < .01). The analysis also showed a significant increase in MAO-B mRNA expression after 120 min exposure to compound **17** as compared to 30 and 60 min exposure (*** *p* < .001). (B) One way ANOVA followed by Newman–Keuls multiple comparison tests showed a significant decrease in MAO-B mRNA expression after 120 min exposure to compound **14** as compared to 0 (**p < .01) and 60 (*p < .05) min exposure time points. Data are shown as mean ± *SEM* (*p < .05,**p < .01, *** *p* < .001).

We further determined the time-dependent effects of the negative control, and compounds **14** and **17** on the relative mRNA expression of MAO-B at 0, 30, 60, and 120 min. However, using one way ANOVA, there was a significant change in the MAO-B mRNA expression among different time points following exposure to compound **17** [*F*(3, 26) = 14.69913, *p* < .0001, (Figure 10(A))]. One way ANOVA followed by Newman–Keuls analysis revealed that compound **17** treatment reduced the relative mRNA levels of MAO-B at 30 and 60 min but not at 120 min compared to the 0 min time point. Moreover, we further analyzed the effects of compound **14** on the relative expression of mRNA at different time points. One-way ANOVA revealed a significant difference in MAO-B mRNA expression among different times of exposure to compound **14** [*F*(3, 29) = 5.308331, *p* < .01, (Figure 10(B))]. One-way ANOVA followed by Newman–Keuls analysis showed that compound **14** decreased mRNA expression of MAO-B only at 120 min compared to the 0 min time point. These findings further support the previous data obtained in Figure 9, which indicate that compound **14** reduced the mRNA expression of MAO-B enzyme at 120 min. However, our work showed that the effects of compound **17** on reducing MAO-B mRNA expression started at 30 min. This indicates that the onset of inhibitory effects of compound **14** is slower than compound **17**.

## Conclusions

Chalcones are the natural precursors for the biosynthesis of flavonoids. These drugs have interesting chemical structures that include two aromatic rings linked with an enone linker (C_6_-C_3_-C_6_). In this study, 22 new chalcone analogs were prepared via Claisen–Schmidt condensation. Their inhibitory activities were evaluated against human MAO-A and MAO-B. The inhibition data showed differing activities and selectivities for MAO-A and MAO-B. Compound **13** showed a high degree of selectivity and activity against MAO-B with an IC_50_ value of 34 nM and selectivity index >1338. Interestingly, the introduction of 2,4,6-trimethoxy substituents on the B aromatic ring shifted the selectivity toward MAO-A as exemplified by compounds **18** and **19**. These compounds were potent and selective MAO-A inhibitors with IC_50_ values of 0.56 μM (selectivity index <0.005) and 0.444 μM (selectivity index <0.004), respectively. As shown with compound **16**, the chalcones are reversible MAO inhibitors as inhibitory activity is reversed by dialysis. Four compounds (**12**, **1**4, **16**, and **17**) were selected to assess their effects on cellular dopamine concentrations using H4IIE cells, with compounds **14** and **17** showing a significant increase in intracellular dopamine concentrations. Furthermore, these two compounds were found to inhibit the relative mRNA expression of MAO-B.

## Supplementary Material

Supplemental Material
