# Supplementary material for: SAR and molecular mechanism studies of monoamine oxidase inhibition by selected chalcone analogs
Source: J Enzyme Inhib Med Chem. 2019 Mar 27;34(1):863–76. doi: 10.1080/14756366.2019.1593158 (PMC6442233; doi:10.1080/14756366.2019.1593158)
Supplement: Supplemental Material [file IENZ_A_1593158_SM9500.zip › IENZ_1593158_Supplementary Material/Compounds_1_22_Total.pdf]

D12

^{1}H NMR (400 MHz, $CDCl_3$) δ 7.93 (d, J = 8.5 Hz, 2H), 7.77 (d, J = 15.4 Hz, 1H), 7.53 (d, J = 8.8 Hz, 2H), 7.43 (d, J = 8.5 Hz, 2H), 7.26 (d, J = 15.4 Hz, 1H), 6.67 (d, J = 8.8 Hz, 2H), 3.03 (s, 6H).

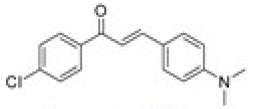

Compound 4

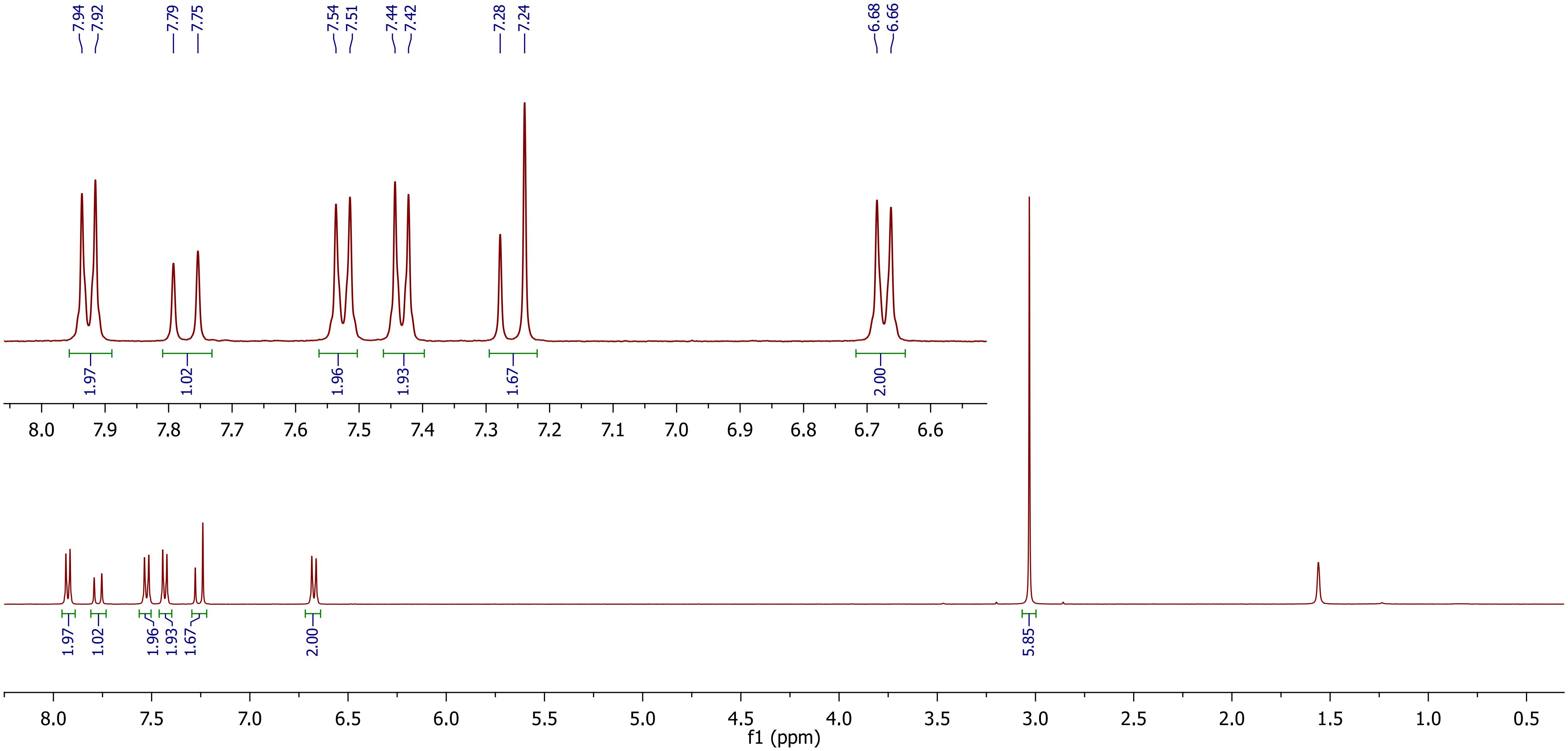

D12

^{13}C NMR (101 MHz, $CDCl_3$) δ 189.29 (s), 152.18 (s), 146.38 (s), 138.45 (s), 137.42 (s), 130.56 (s), 129.74 (s), 128.74 (s), 122.47 (s), 116.26 (s), 111.83 (s), 40.14 (s).

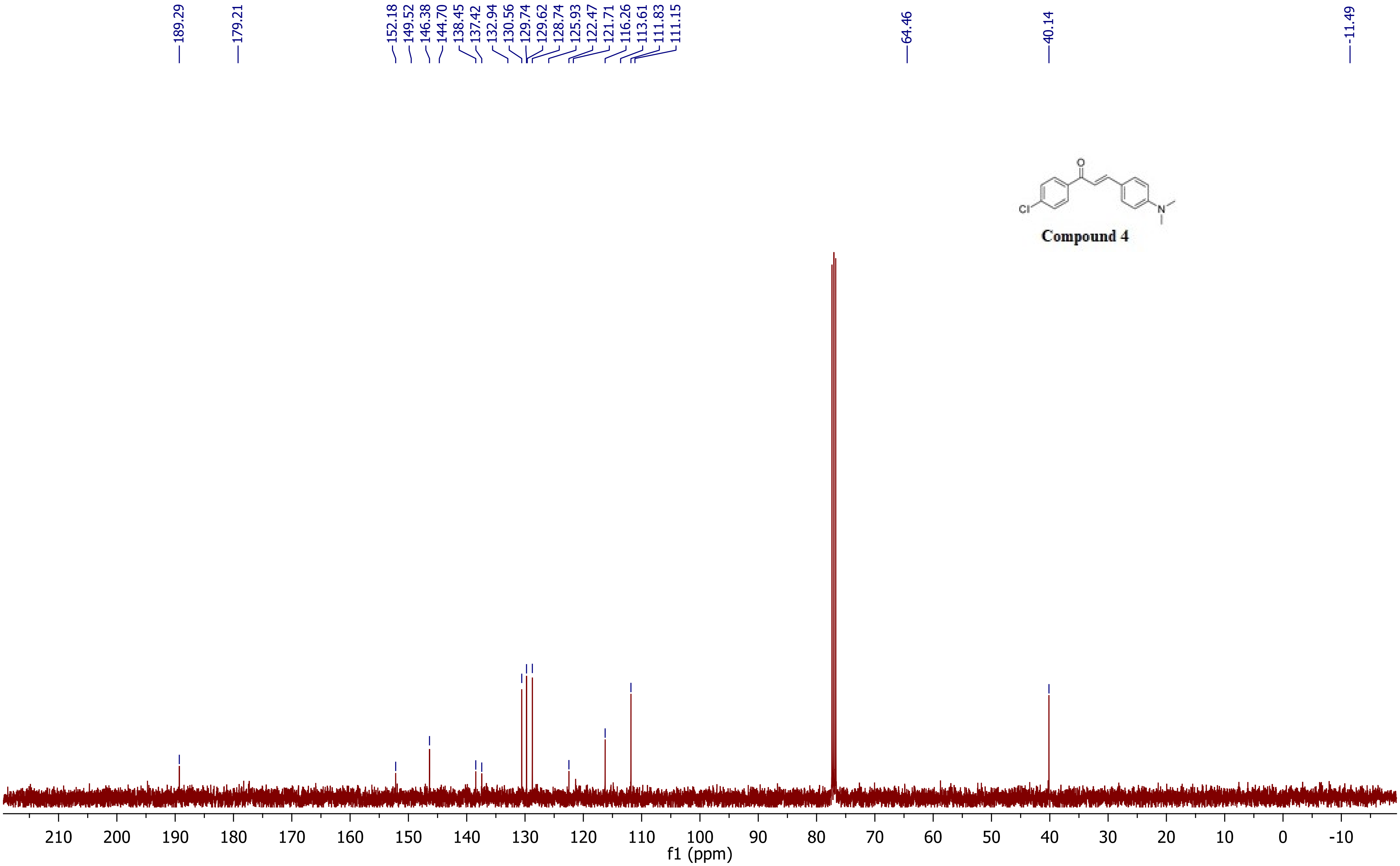

D12

Title	ASHRAF-D12
Solvent	CDCl3
Pulse Sequence	hsqc
Spectrometer Frequency	(400.15, 100.62)
Nucleus	(1H, 13C)

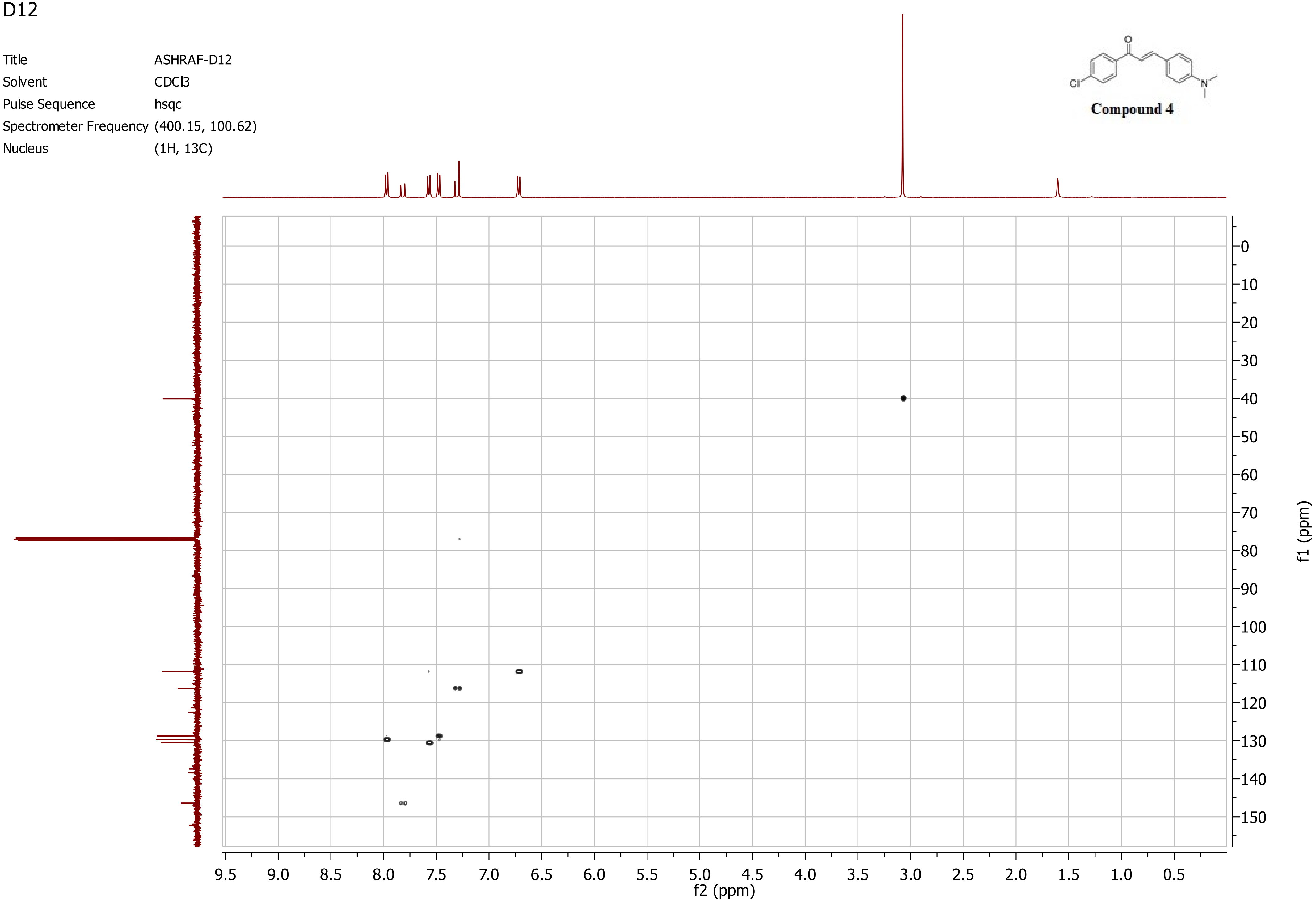

Compound 4

D12

Title	ASHRAF-D12
Solvent	CDCl3
Pulse Sequence	hsqc
Spectrometer Frequency	(400.15, 100.62)
Nucleus	(1H, 13C)

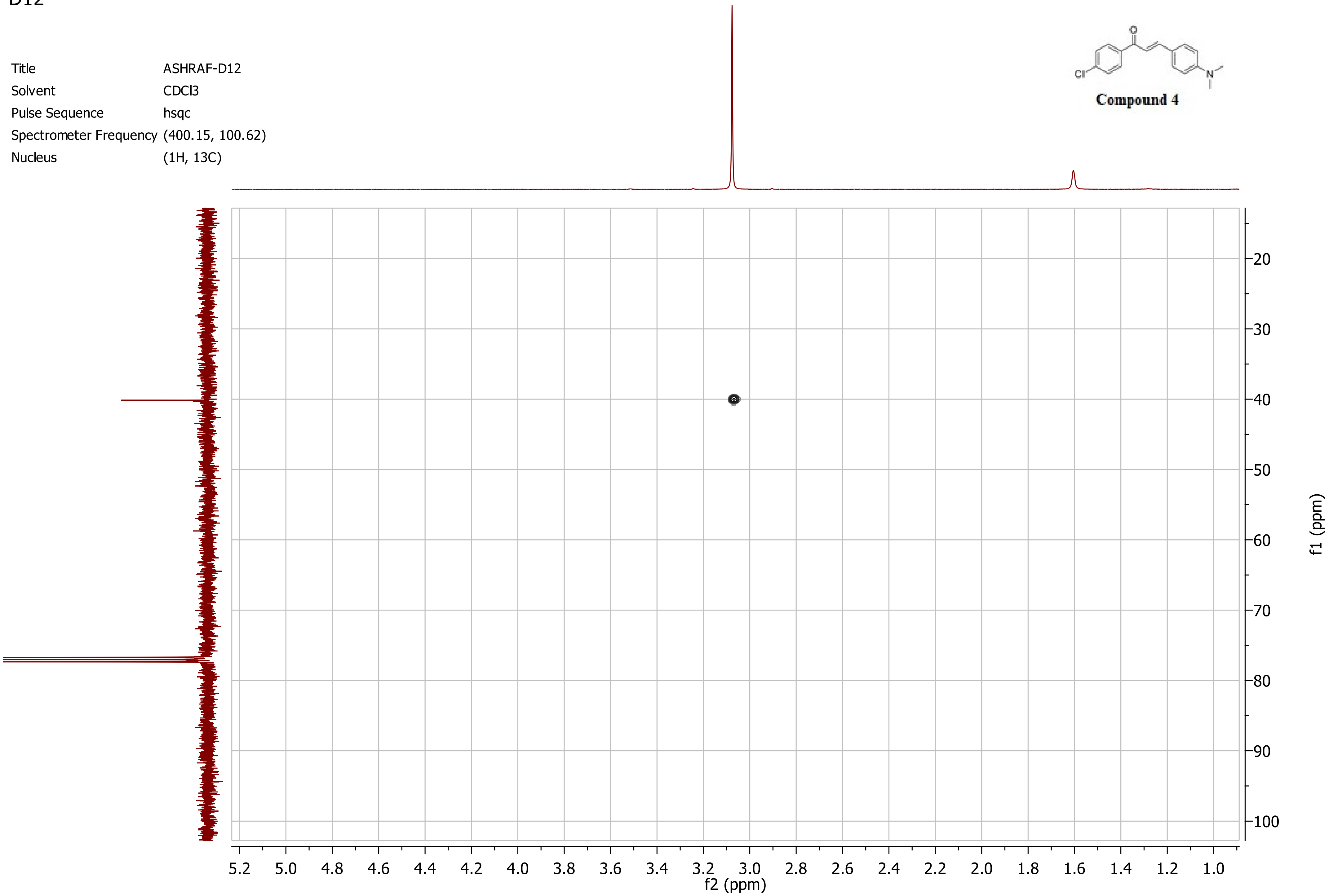

D12

Title	ASHRAF-D12
Solvent	CDCl3
Pulse Sequence	hsqc
Spectrometer Frequency	(400.15, 100.62)
Nucleus	(1H, 13C)

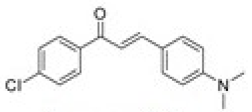

Compound 4

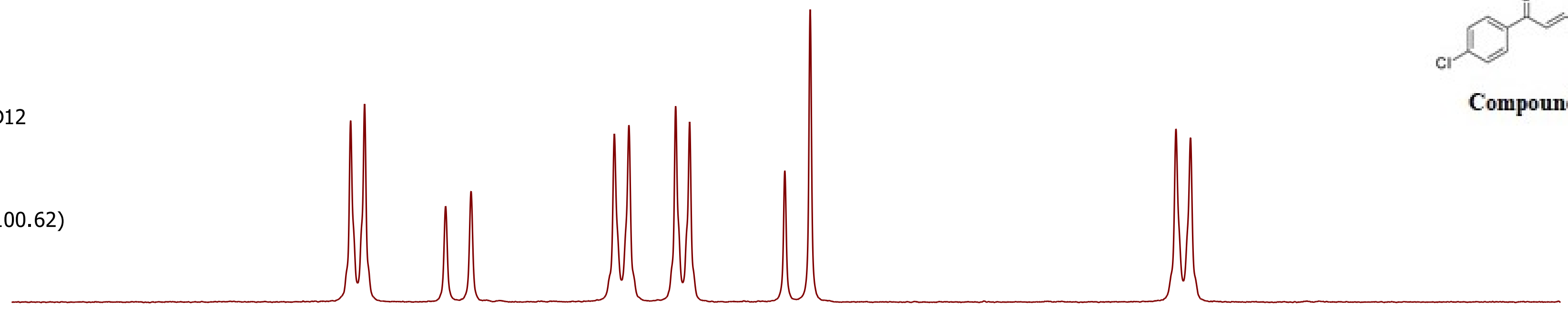
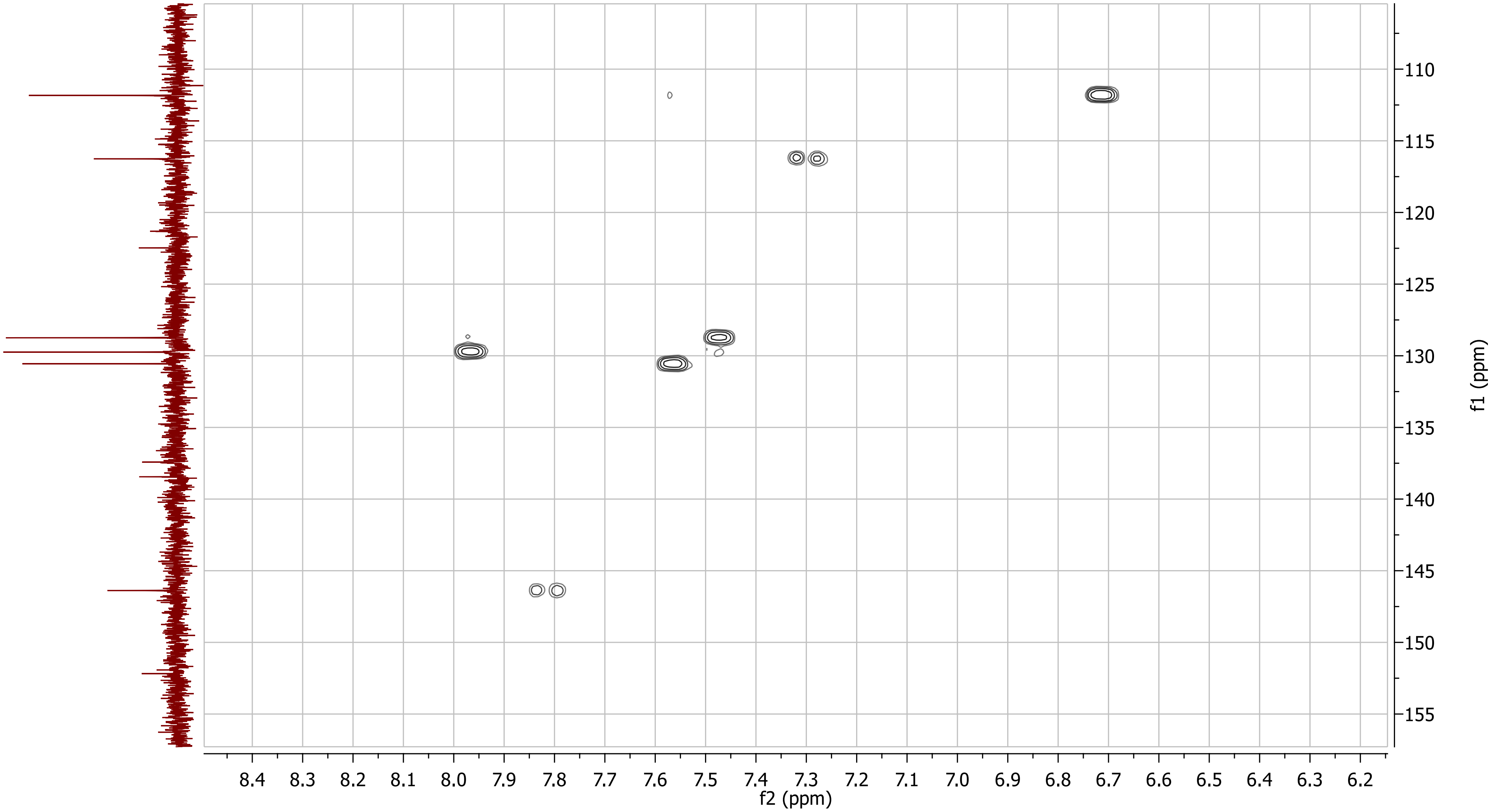

D12

Title	ASHRAF-D12
Solvent	CDCl3
Pulse Sequence	hsqc
Spectrometer Frequency	(400.15, 100.62)
Nucleus	(1H, 13C)

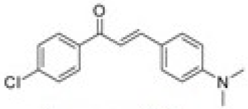

Compound 4

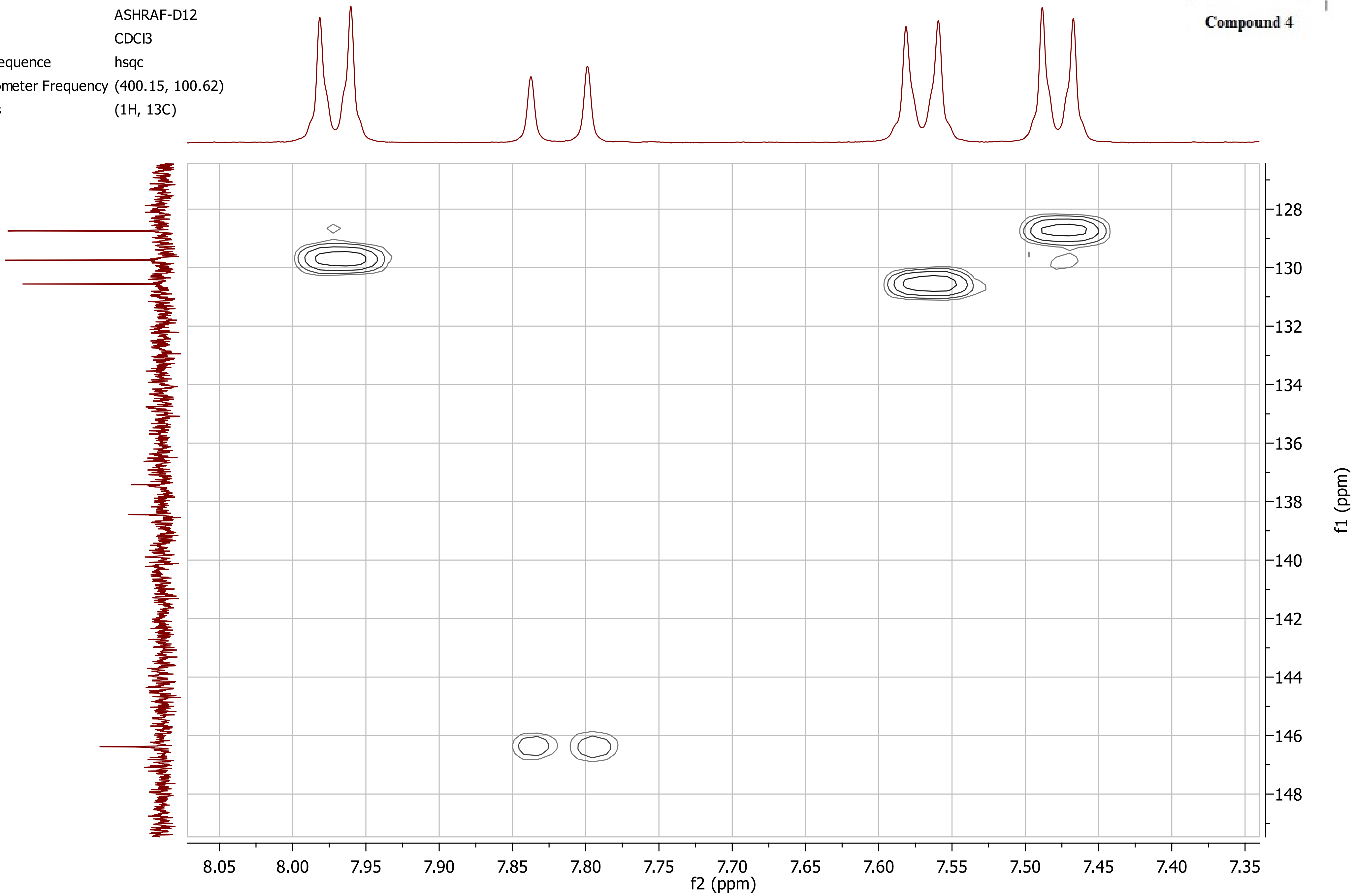

D12

Title	ASHRAF-D12
Solvent	CDCl3
Pulse Sequence	cosy
Spectrometer Frequency	(400.15, 400.15)
Nucleus	(1H, 1H)

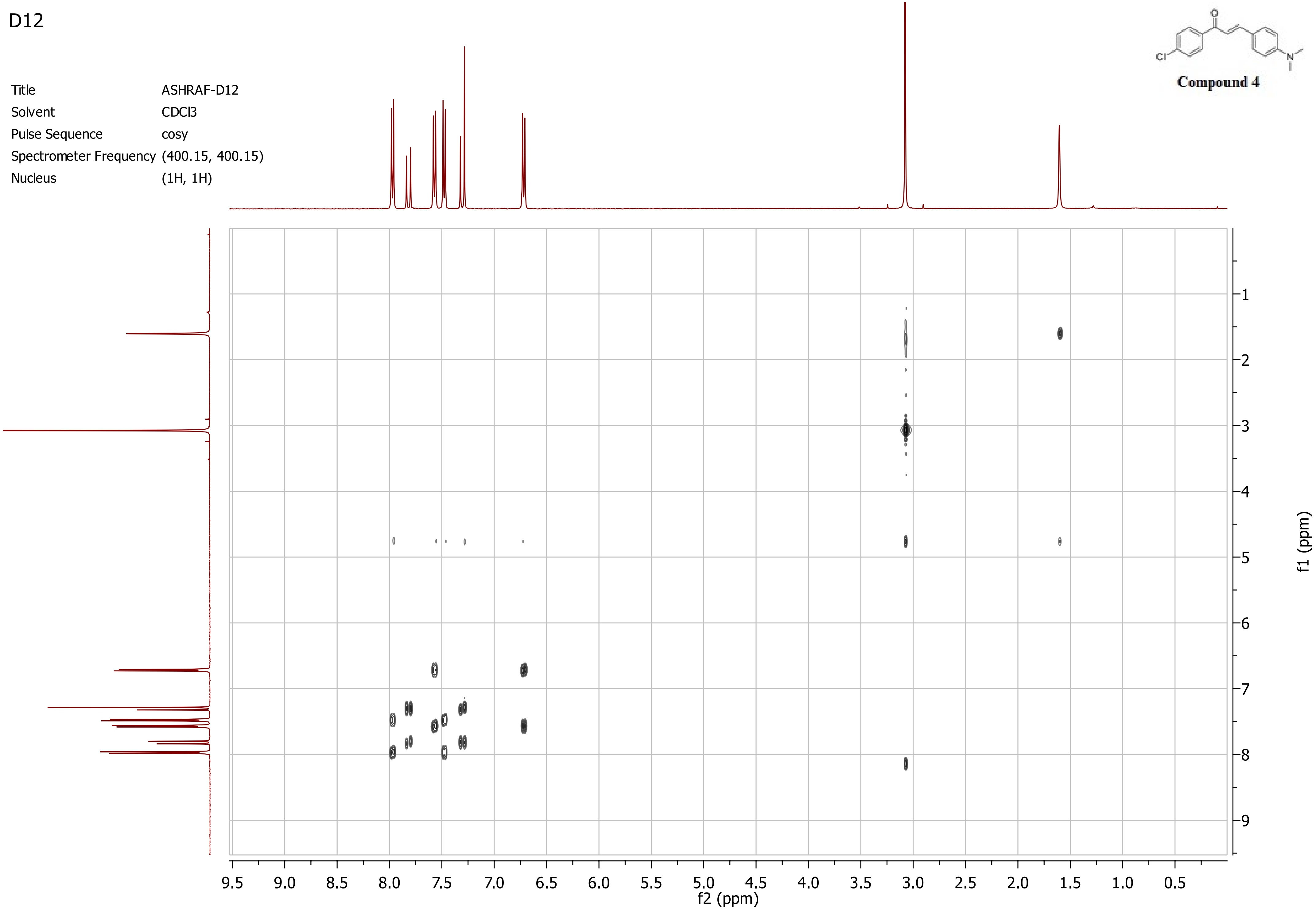

D12

Title	ASHRAF-D12
Solvent	CDCl3
Pulse Sequence	cosy
Spectrometer Frequency	(400.15, 400.15)
Nucleus	(1H, 1H)

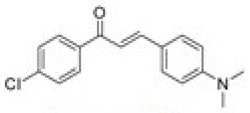

Compound 4

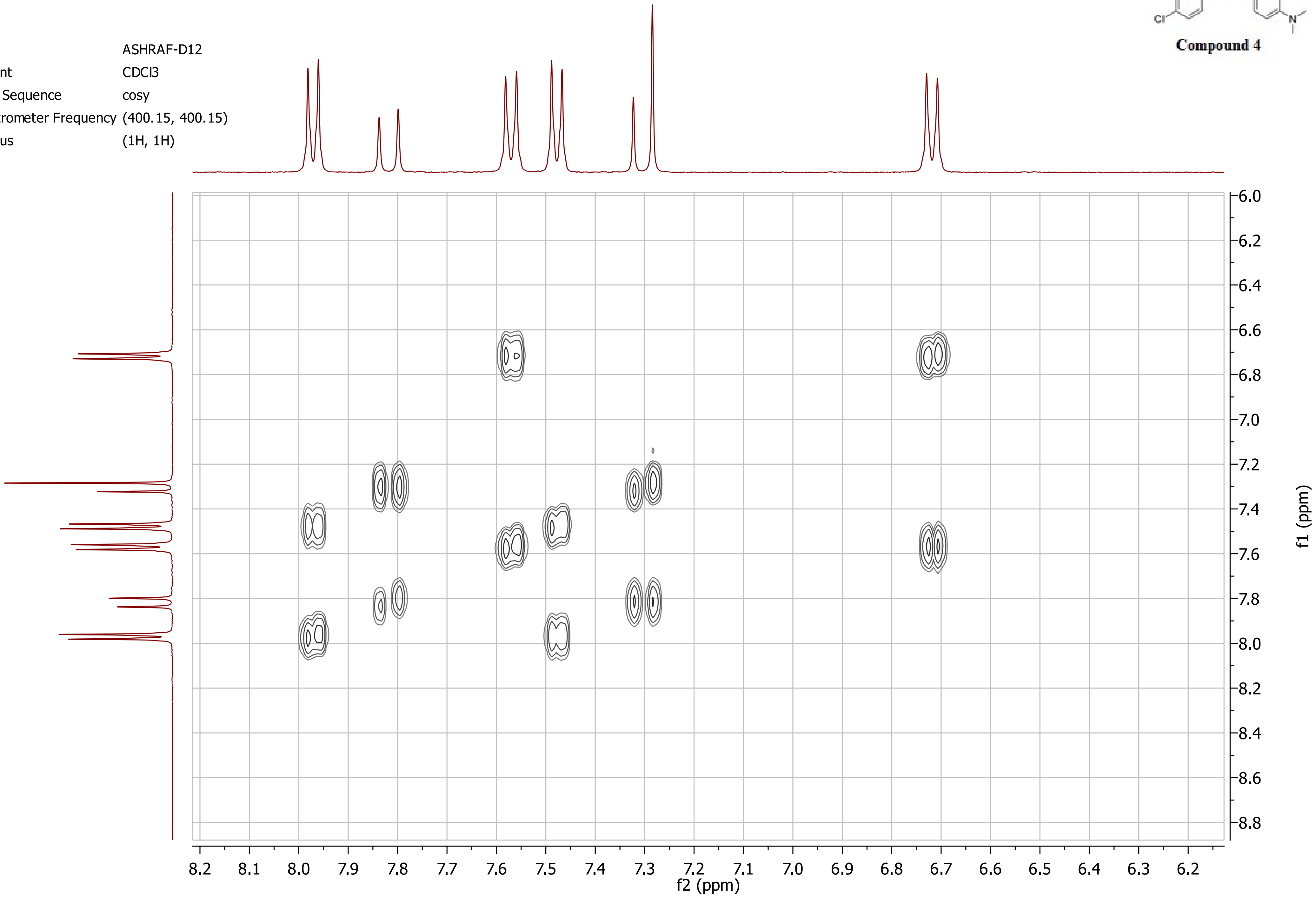

D12

Title	ASHRAF-D12
Solvent	CDCl3
Pulse Sequence	cosy
Spectrometer Frequency	(400.15, 400.15)
Nucleus	(1H, 1H)

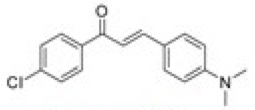

Compound 4

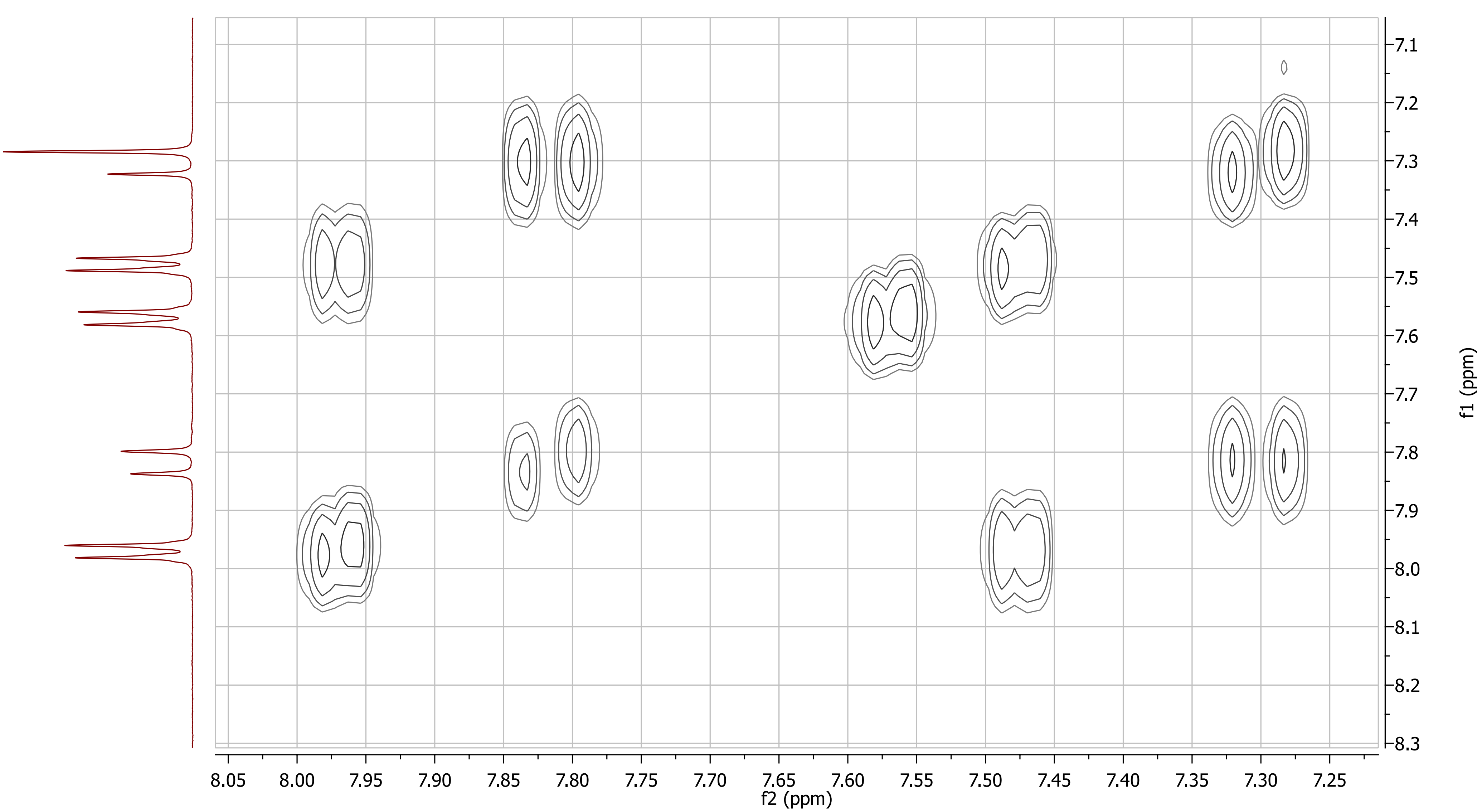

D12

Title	ASHRAF-D12
Solvent	CDCl3
Pulse Sequence	hmbc
Spectrometer Frequency	(400.15, 100.62)
Nucleus	(1H, 13C)

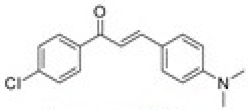

Compound 4

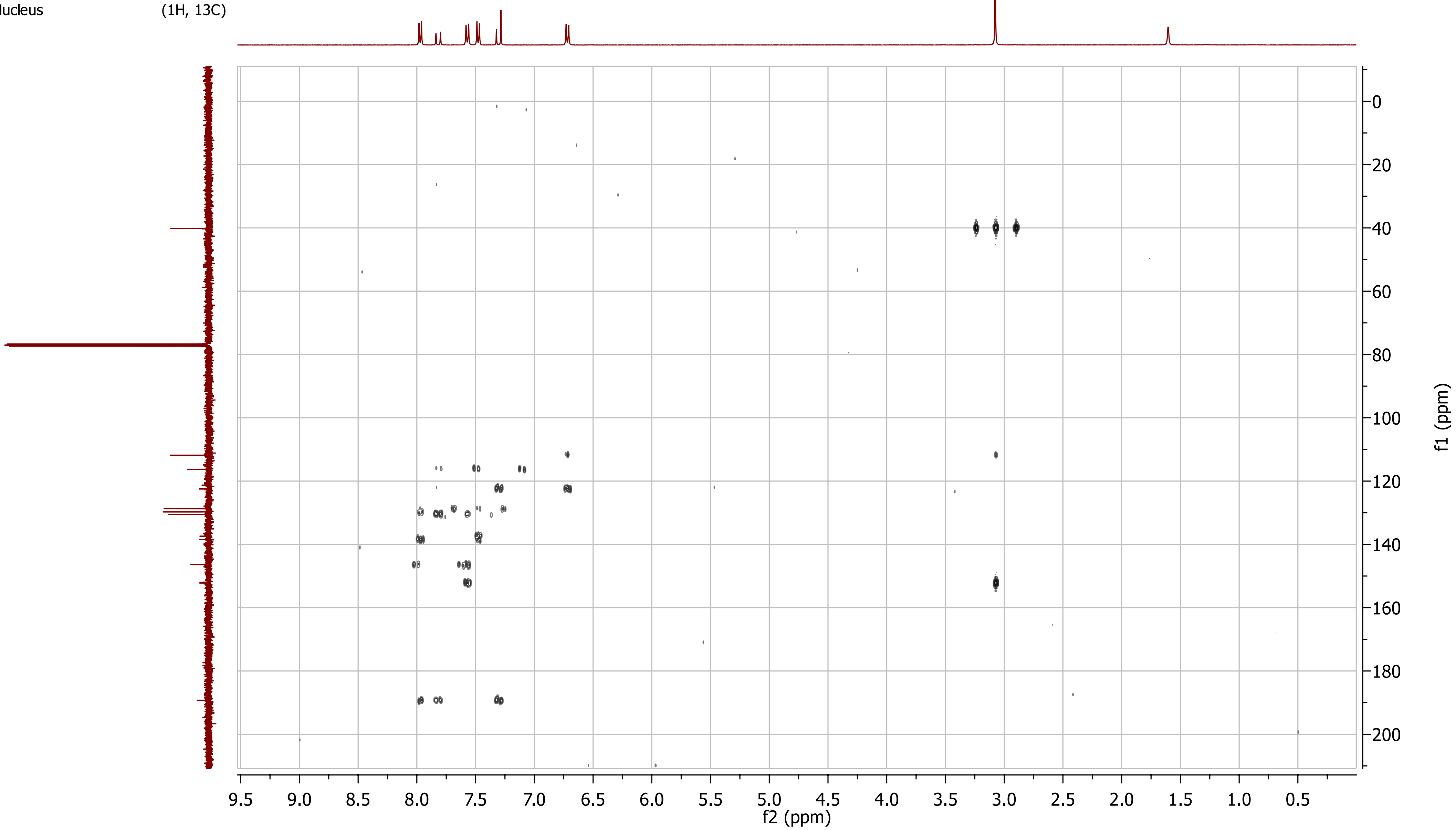

D12

Title	ASHRAF-D12
Solvent	CDCl3
Pulse Sequence	hmbc
Spectrometer Frequency	(400.15, 100.62)
Nucleus	(1H, 13C)

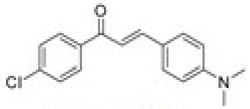

Compound 4

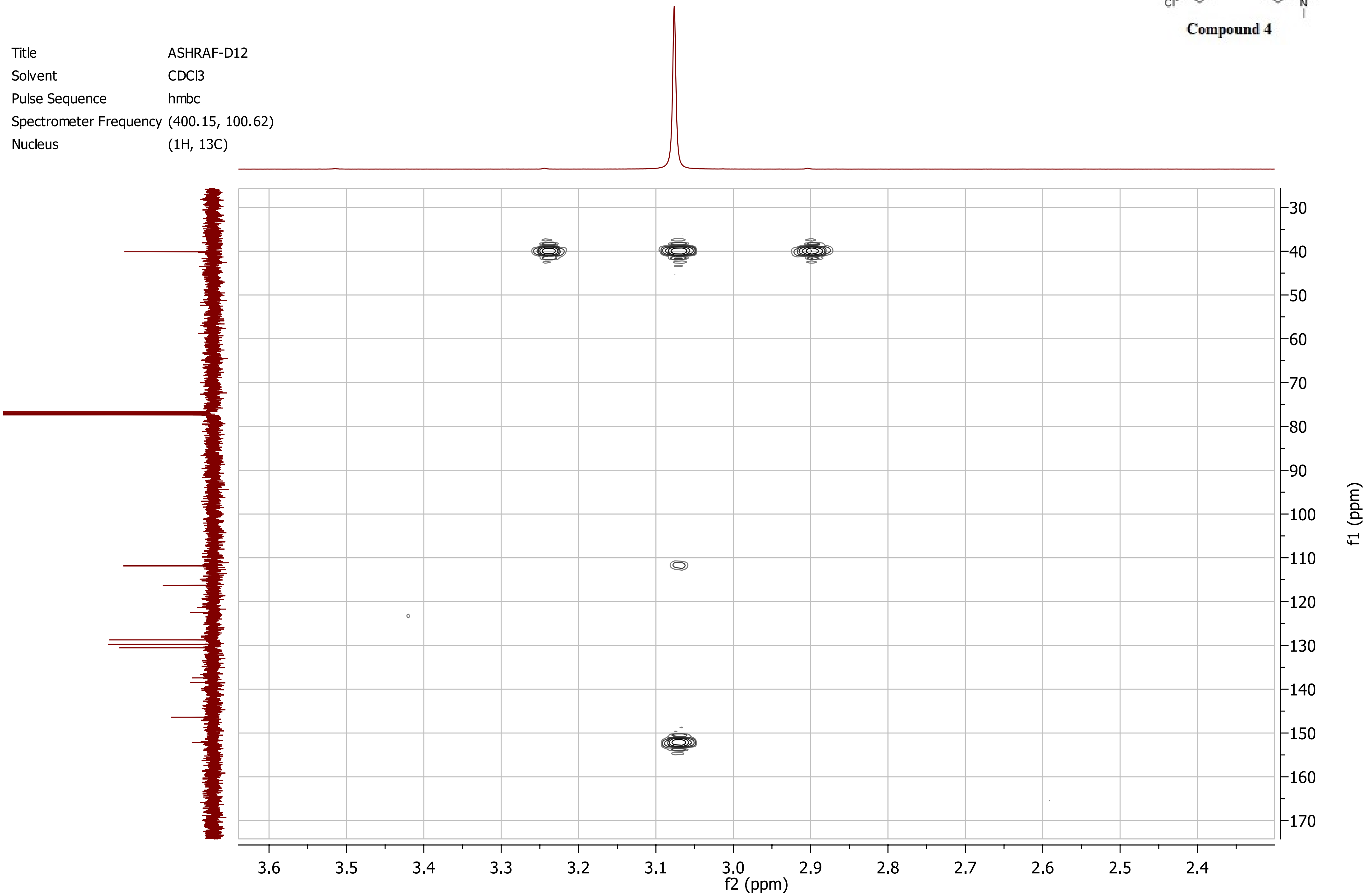

D12

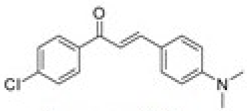

Compound 4

Title	ASHRAF-D12
Solvent	CDCl3
Pulse Sequence	hmbc
Spectrometer Frequency	(400.15, 100.62)
Nucleus	(1H, 13C)

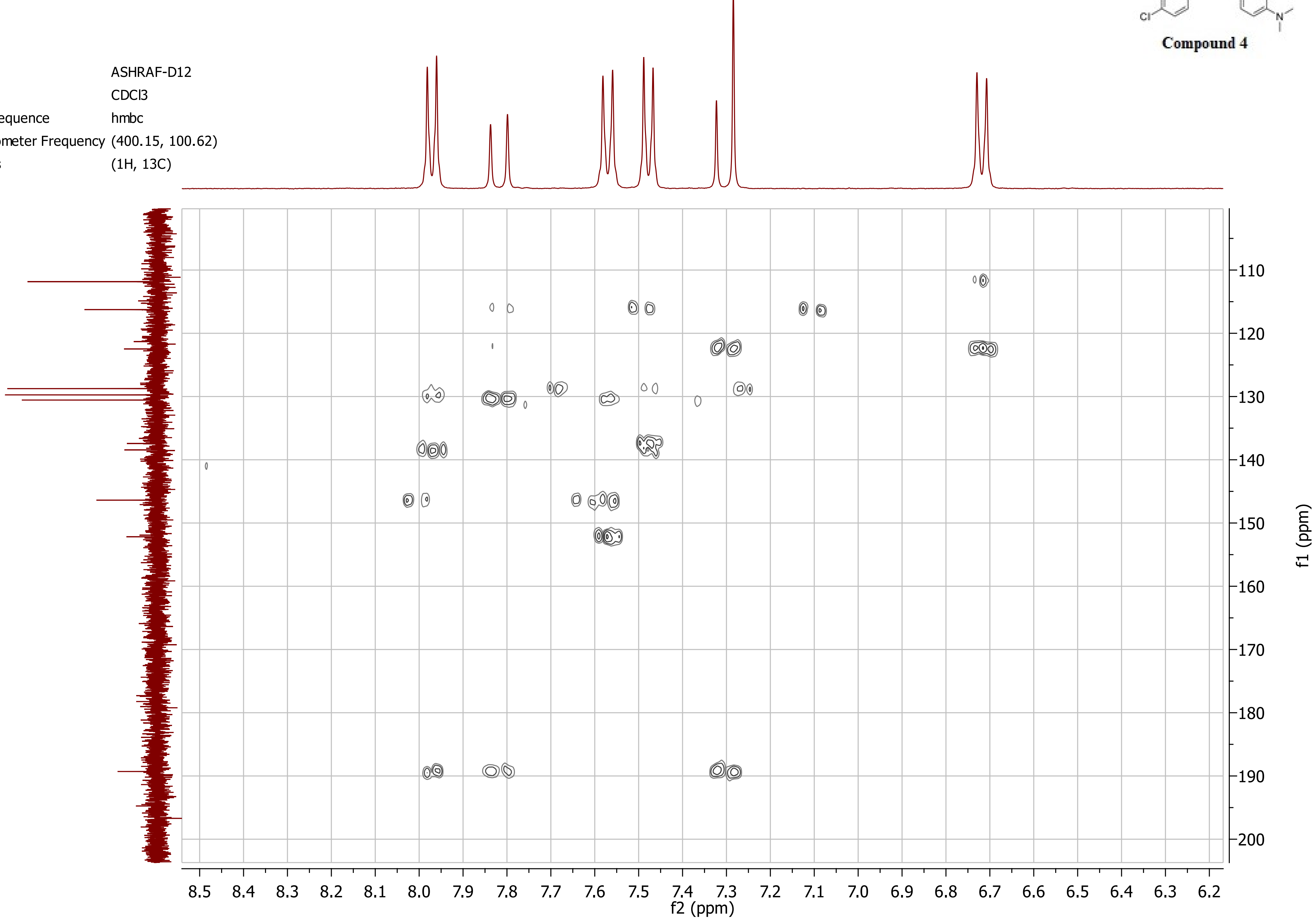

D12

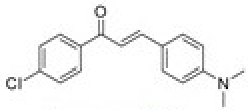

Compound 4

Title	ASHRAF-D12
Solvent	CDCl3
Pulse Sequence	hmbc
Spectrometer Frequency	(400.15, 100.62)
Nucleus	(1H, 13C)

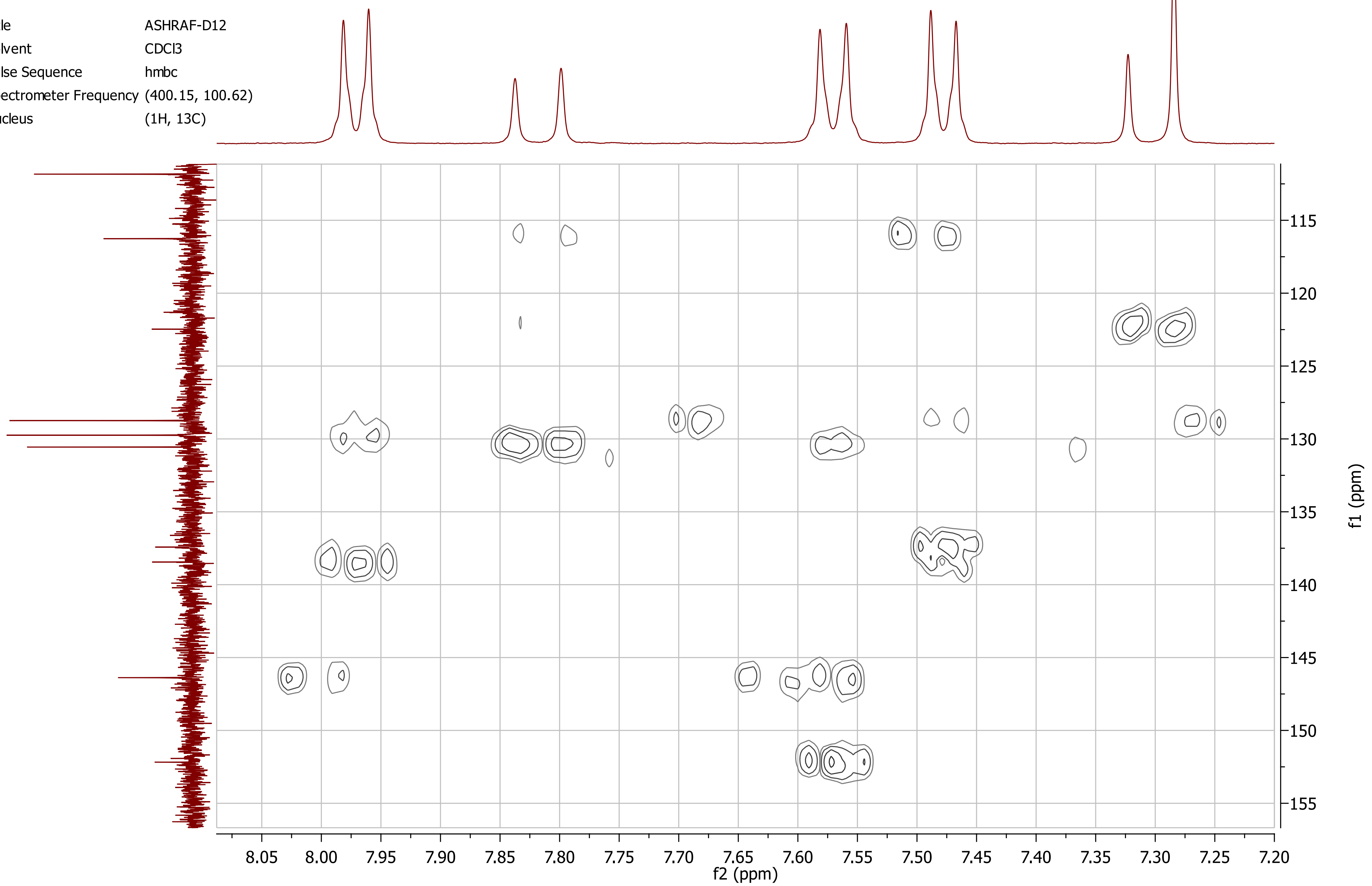

{C:\Bruker\TOPSF

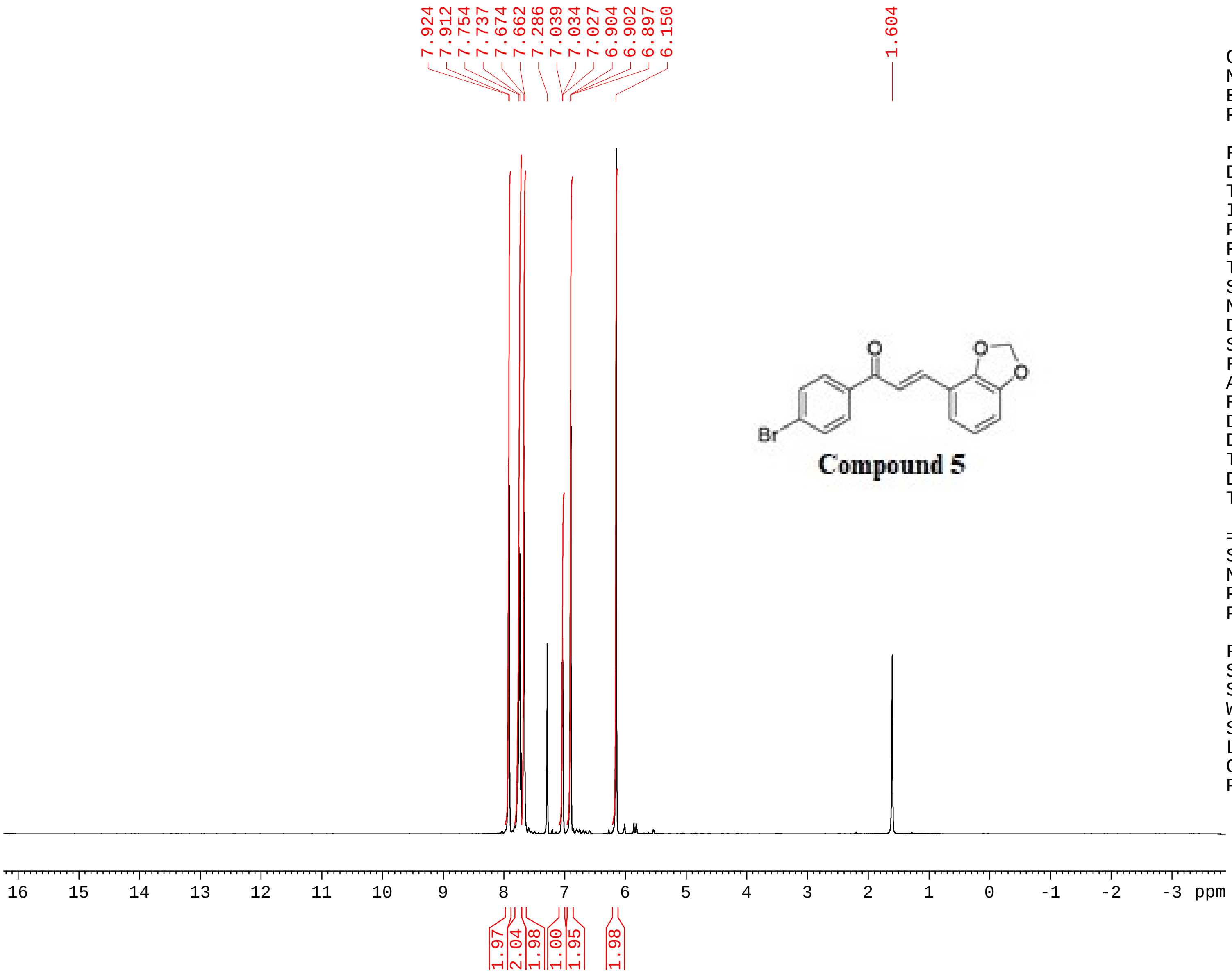

Compound 5

```
Current Data Parameters
NAME       QatarUni-D-5
EXPNO                10
PROCNO                1

F2 - Acquisition Parameters
Date_          20160517
Time              14.56
INSTRUM           spect
PROBHD   5 mm CPTCI 1H-
PULPROG            zg30
TD                65536
SOLVENT           CDCl3
NS                   16
DS                    2
SWH          14097.744 Hz
FIDRES        0.215115 Hz
AQ           2.3243434 sec
RG                83.85
DW               35.467 usec
DE                31.86 usec
TE                295.0 K
D1           1.00000000 sec
TD0                   1

======== CHANNEL f1 ========
SFO1        700.1743238 MHz
NUC1                 1H
P1                 8.00 usec
PLW1         9.64999962 W

F2 - Processing parameters
SI                65536
SF          700.1700000 MHz
WDW                  EM
SSB       0
LB                 0.30 Hz
GB        0
PC                 1.00
```

{C:\Bruker\TOPSF

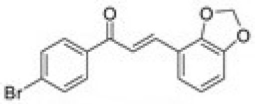

Compound 5

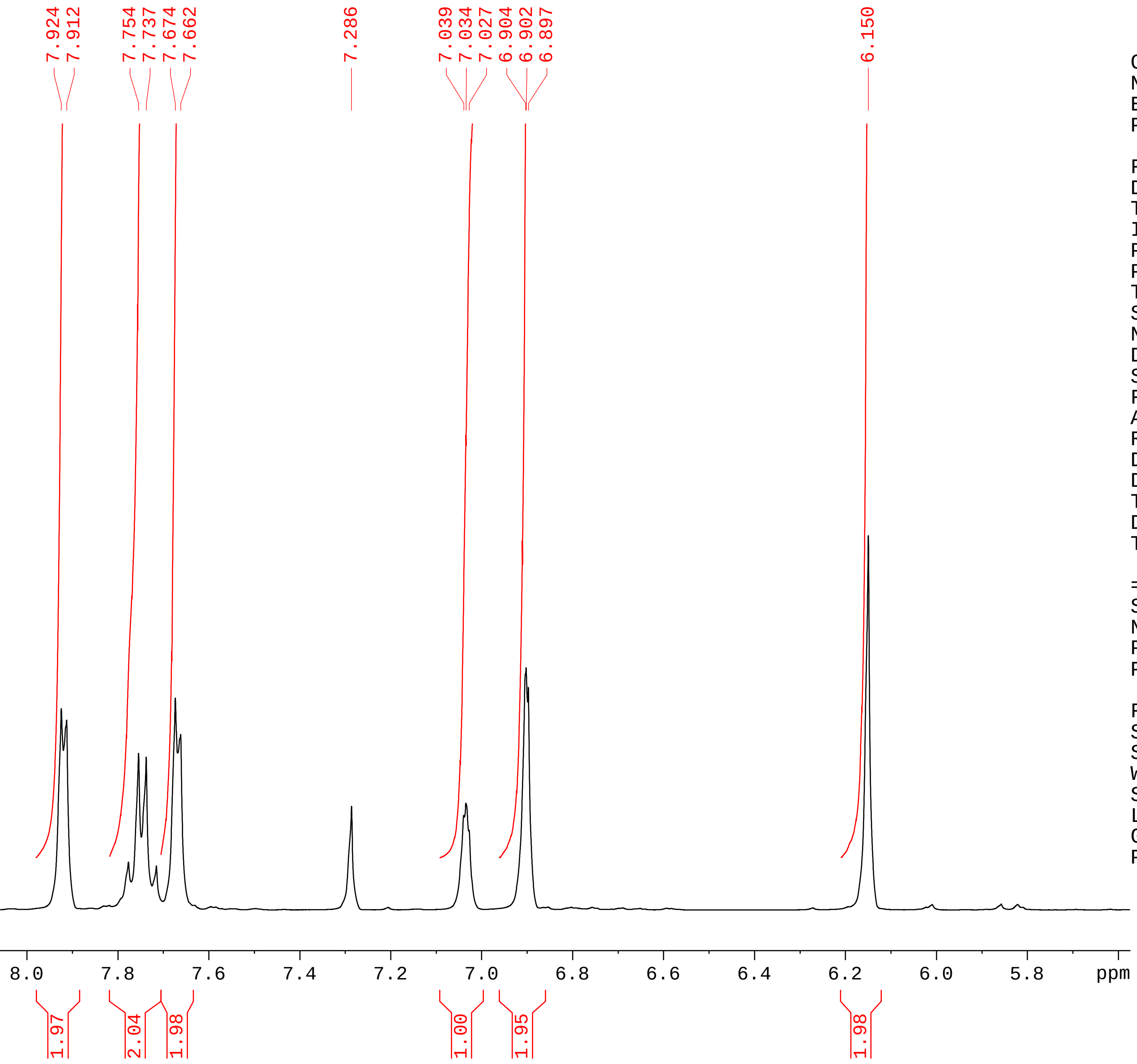

Current Data Parameters
NAME QatarUni-D-5
EXPNO 10
PROCNO 1

F2 - Acquisition Parameters
Date_ 20160517
Time 14.56
INSTRUM spect
PROBHD 5 mm CPTCI 1H-
PULPROG zg30
TD 65536
SOLVENT CDCl3
NS 16
DS 2
SWH 14097.744 Hz
FIDRES 0.215115 Hz
AQ 2.3243434 sec
RG 83.85
DW 35.467 usec
DE 31.86 usec
TE 295.0 K
D1 1.00000000 sec
TD0 1

======== CHANNEL f1 ========
SFO1 700.1743238 MHz
NUC1 1H
P1 8.00 usec
PLW1 9.64999962 W

F2 - Processing parameters
SI 65536
SF 700.1700000 MHz
WDW EM
SSB 0
LB 0.30 Hz
GB 0
PC 1.00

C13CPD CDCl3 {C:\Bruker\TOPSPIN} abari 38

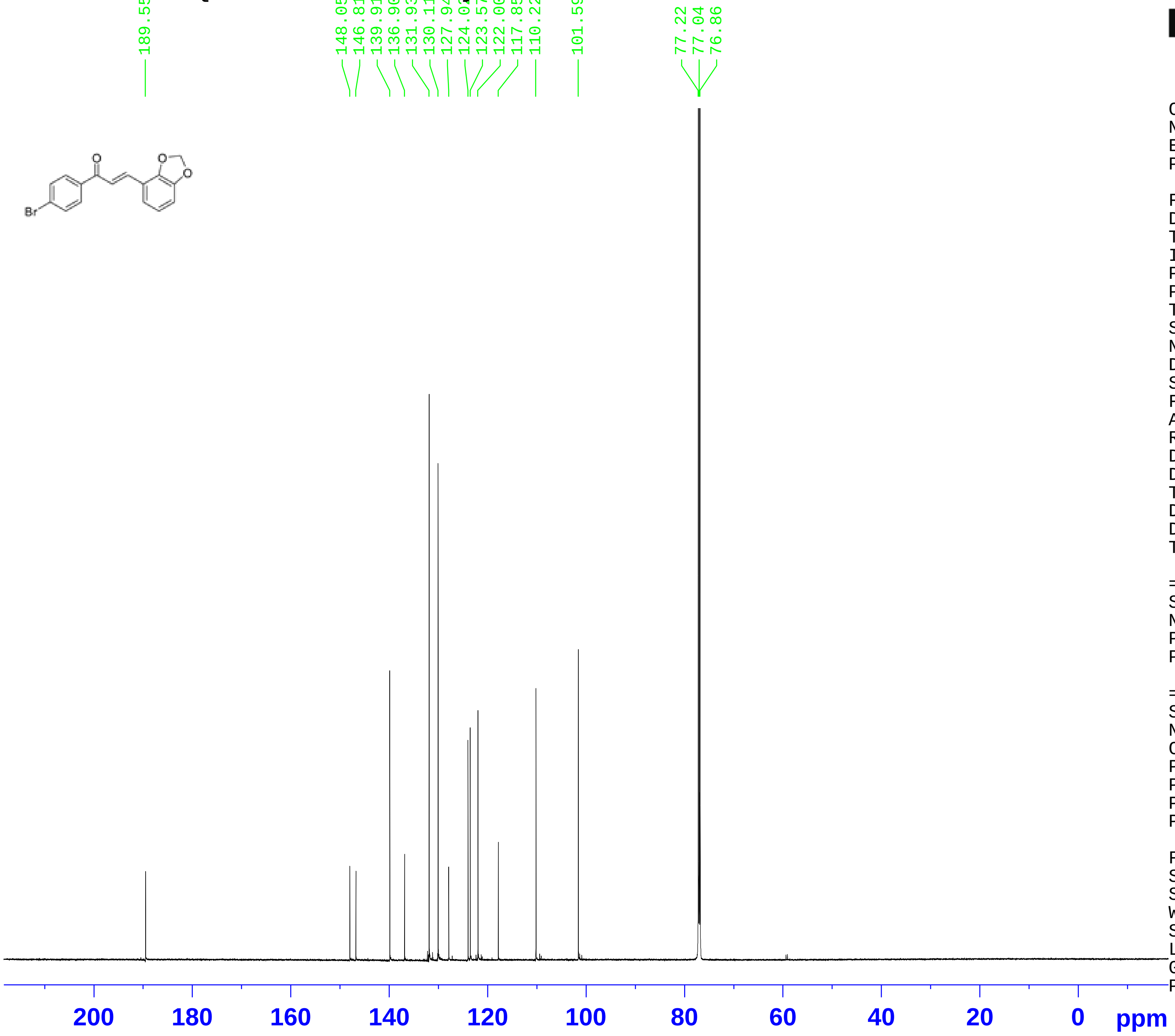


```
Current Data Parameters
NAME        QatarUni-D-5
EXPNO                 11
PROCNO                 1

F2 - Acquisition Parameters
Date_           20160517
Time               15.14
INSTRUM            spect
PROBHD   5 mm CPTCI 1H-
PULPROG           zgpg30
TD                 65536
SOLVENT            CDCl3
NS                  3091
DS                     4
SWH           41666.668 Hz
FIDRES         0.635783 Hz
AQ            0.7864320 sec
RG                 172.3
DW                12.000 usec
DE                 18.00 usec
TE                 295.0 K
D1            2.00000000 sec
D11           0.03000000 sec
TD0                    1

======== CHANNEL f1 ========
SFO1        176.0754915 MHz
NUC1                 13C
P1                 12.00 usec
PLW1        121.00000000 W

======== CHANNEL f2 ========
SFO2        700.1728007 MHz
NUC2                  1H
CPDPRG[2         waltz16
PCPD2              65.00 usec
PLW2          9.64999962 W
PLW12         0.14618000 W
PLW13         0.06176000 W

F2 - Processing parameters
SI                 32768
SF           176.0578870 MHz
WDW                   EM
SSB      0
LB                  1.00 Hz
GB       0
PC                  1.40
```

C13CPD CDCl3 {C:\Bruker\TOPSPIN} abari 38

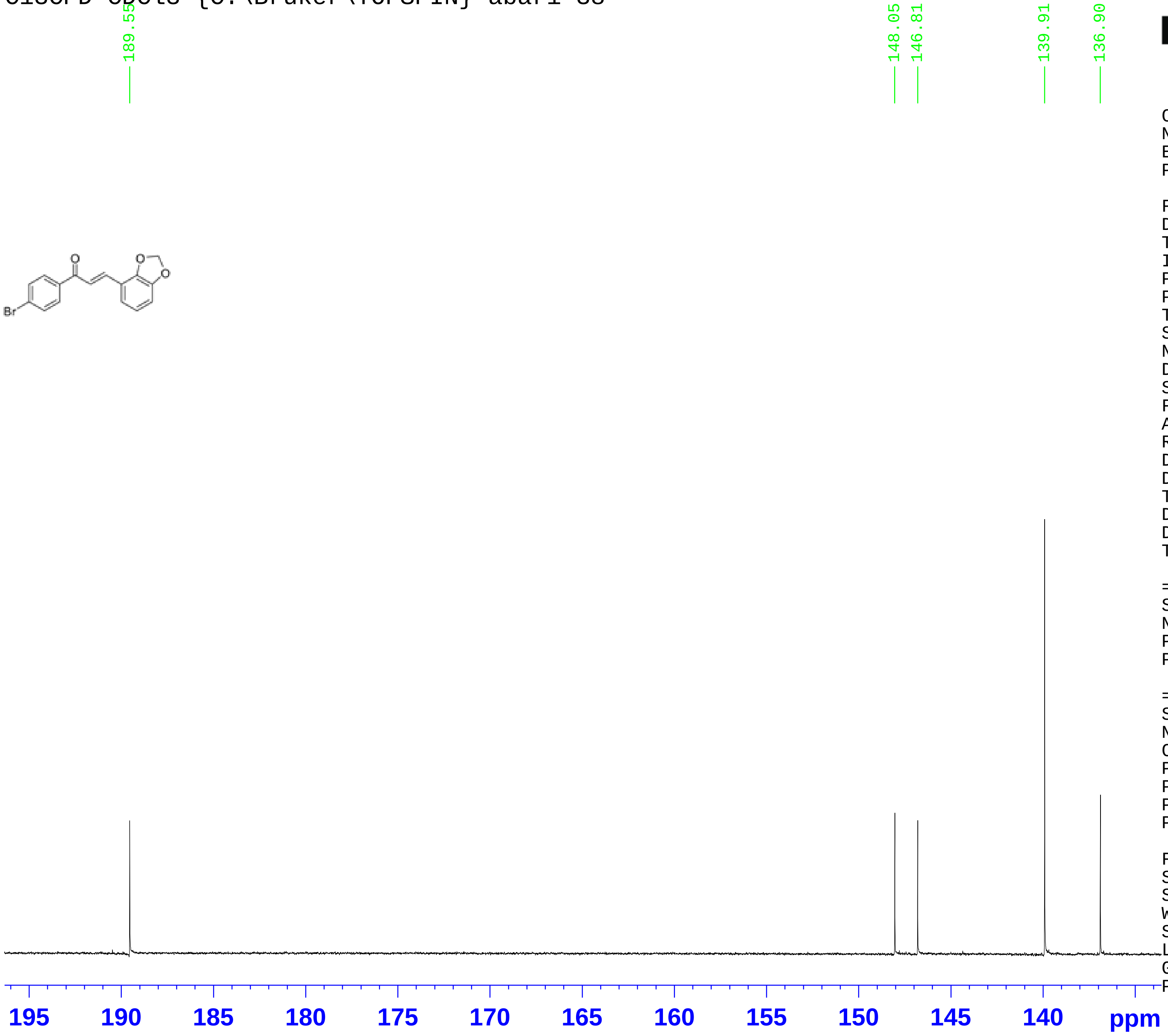


```
Current Data Parameters
NAME          QatarUni-D-5
EXPNO                   11
PROCNO                   1

F2 - Acquisition Parameters
Date_             20160517
Time                 15.14
INSTRUM              spect
PROBHD    5 mm CPTCI 1H-
PULPROG            zgpg30
TD                   65536
SOLVENT              CDCl3
NS                    3091
DS                       4
SWH            41666.668 Hz
FIDRES          0.635783 Hz
AQ             0.7864320 sec
RG                   172.3
DW                  12.000 usec
DE                   18.00 usec
TE                   295.0 K
D1            2.00000000 sec
D11           0.03000000 sec
TD0                      1

======== CHANNEL f1 ========
SFO1         176.0754915 MHz
NUC1                   13C
P1                   12.00 usec
PLW1        121.00000000 W

======== CHANNEL f2 ========
SFO2         700.1728007 MHz
NUC2                    1H
CPDPRG[2           waltz16
PCPD2                65.00 usec
PLW2          9.64999962 W
PLW12         0.14618000 W
PLW13         0.06176000 W

F2 - Processing parameters
SI                   32768
SF           176.0578870 MHz
WDW                     EM
SSB       0
LB                    1.00 Hz
GB        0
PC                    1.40
```

C13CPD CDCl3 {C:\Bruker\TOPSPIN} abari 38

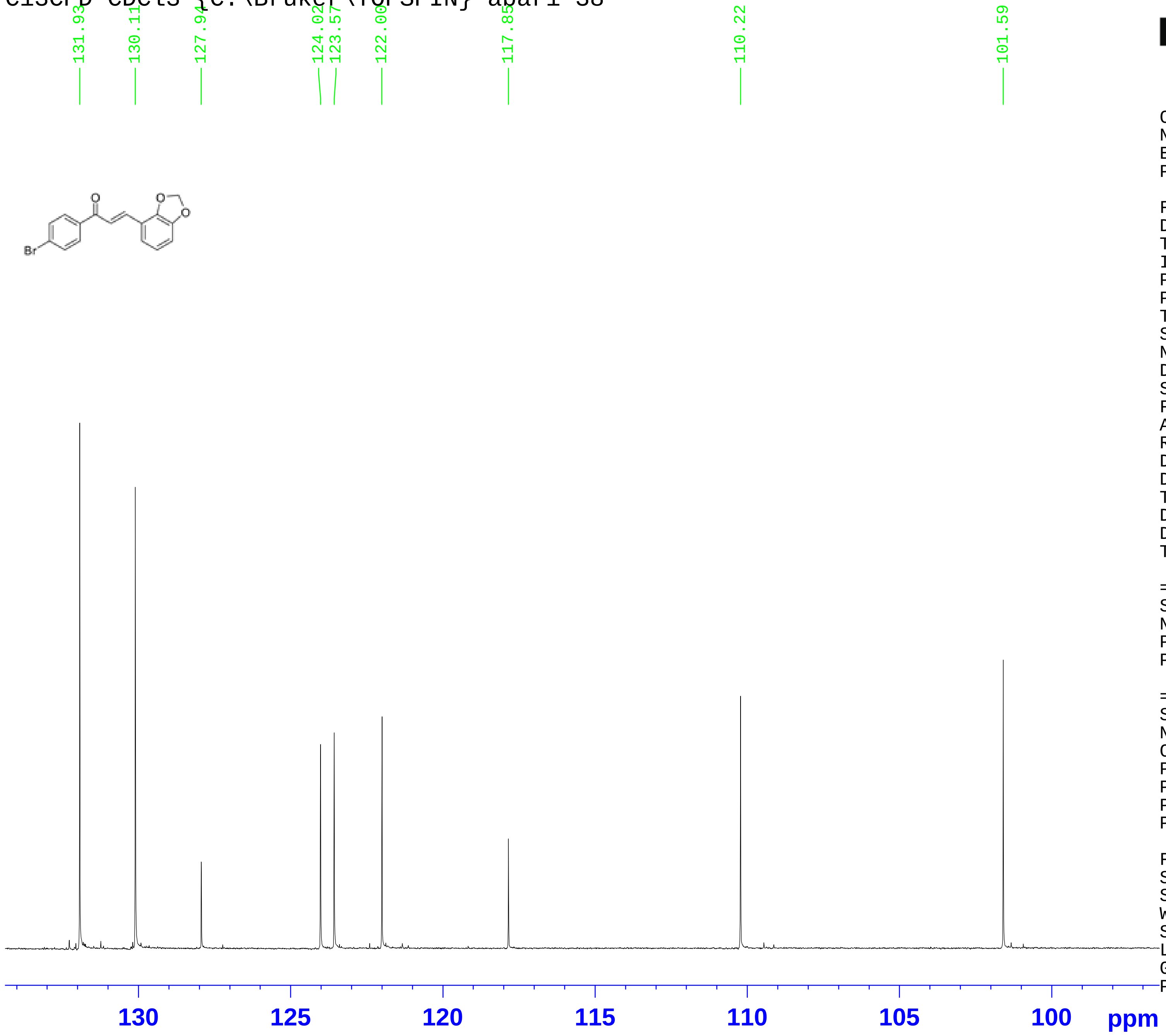

Current Data Parameters
NAME QatarUni-D-5
EXPNO 11
PROCNO 1

F2 - Acquisition Parameters
Date_ 20160517
Time 15.14
INSTRUM spect
PROBHD 5 mm CPTCI 1H-
PULPROG zgpg30
TD 65536
SOLVENT CDCl3
NS 3091
DS 4
SWH 41666.668 Hz
FIDRES 0.635783 Hz
AQ 0.7864320 sec
RG 172.3
DW 12.000 usec
DE 18.00 usec
TE 295.0 K
D1 2.00000000 sec
D11 0.03000000 sec
TD0 1

======== CHANNEL f1 ========
SFO1 176.0754915 MHz
NUC1 13C
P1 12.00 usec
PLW1 121.00000000 W

======== CHANNEL f2 ========
SFO2 700.1728007 MHz
NUC2 1H
CPDPRG[2 waltz16
PCPD2 65.00 usec
PLW2 9.64999962 W
PLW12 0.14618000 W
PLW13 0.06176000 W

F2 - Processing parameters
SI 32768
SF 176.0578870 MHz
WDW EM
SSB 0
LB 1.00 Hz
GB 0
PC 1.40

COSYGPDFPHSW CDCl3 {C:\Bruker\TOPSPIN} abari 38

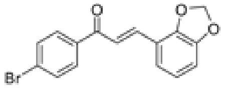

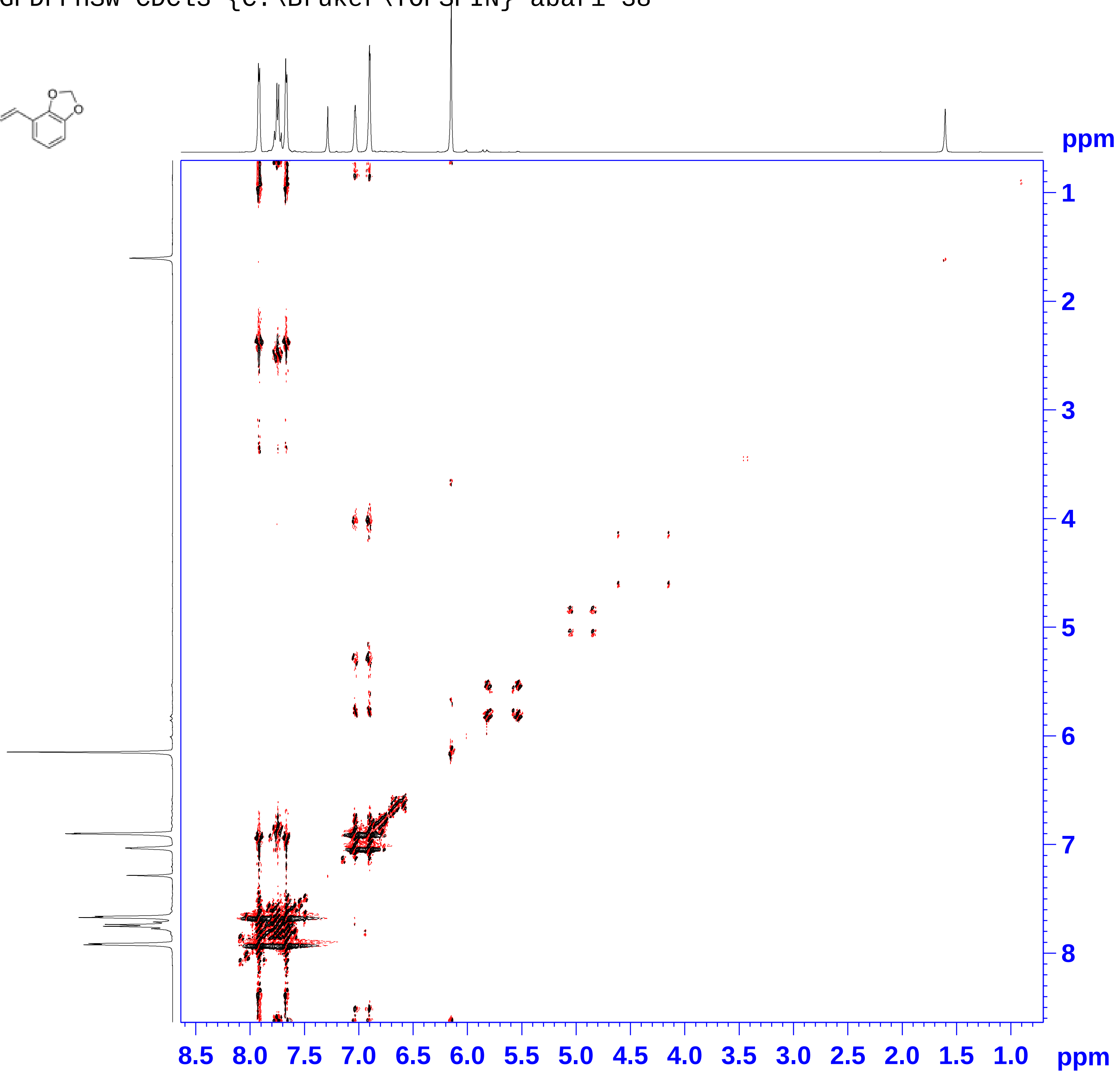


```
Current Data Parameters
NAME          QatarUni-D-5
EXPNO                   13
PROCNO                   1

F2 - Acquisition Parameters
Date_             20160517
Time                 18.23
INSTRUM              spect
PROBHD    5 mm CPTCI 1H-
PULPROG      cosygpmfphpp
TD                    2048
SOLVENT              CDCl3
NS                      32
DS                       4
SWH               5555.556 Hz
FIDRES            2.712674 Hz
AQ               0.1843200 sec
RG                   172.3
DW                  90.000 usec
DE                   30.00 usec
TE                   295.0 K
D0              0.00007981 sec
D1              1.93446398 sec
D11             0.03000000 sec
D12             0.00002000 sec
D16             0.00020000 sec
IN0             0.00018000 sec

======== CHANNEL f1 ========
SFO1           700.1732699 MHz
NUC1                    1H
P1                    8.00 usec
P2                   16.00 usec
P17                2500.00 usec
PLW1            9.64999962 W
PLW10           0.91360998 W

====== GRADIENT CHANNEL =====
GPNAM[1]        SMSQ10.100
GPNAM[2]        SMSQ10.100
GPZ1                 10.00 %
GPZ2                 20.00 %
P16                1000.00 usec

F1 - Acquisition parameters
TD                     256
SFO1              700.1733 MHz
FIDRES           21.701389 Hz
SW                   7.935 ppm
FnMODE         States-TPPI

F2 - Processing parameters
SI                    1024
SF             700.1700000 MHz
WDW                  QSINE
SSB                      2
LB            0 Hz
GB            0
PC                    1.40

F1 - Processing parameters
SI                    1024
MC2            States-TPPI
SF             700.1700000 MHz
WDW                  QSINE
SSB                      2
LB            0 Hz
GB            0
```

COSYGPDFPHSW CDCl3 {C:\Bruker\TOPSPIN} abari 38

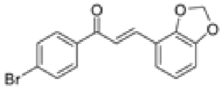

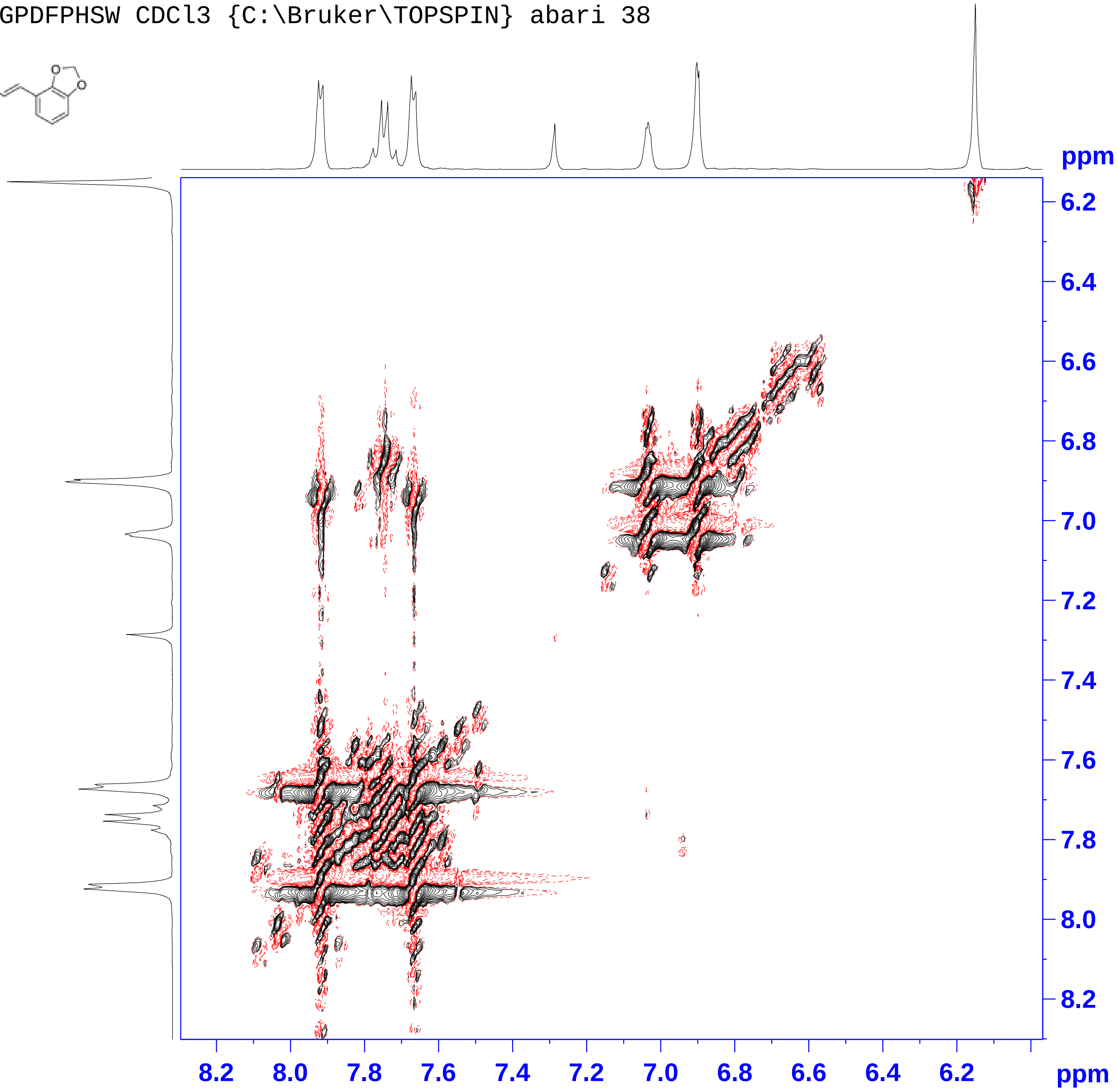

BRUKER

```
Current Data Parameters
NAME          QatarUni-D-5
EXPNO                   13
PROCNO                   1

F2 - Acquisition Parameters
Date_             20160517
Time                 18.23
INSTRUM              spect
PROBHD   5 mm CPTCI 1H-
PULPROG      cosygpmfphpp
TD                    2048
SOLVENT              CDCl3
NS                      32
DS                       4
SWH               5555.556 Hz
FIDRES            2.712674 Hz
AQ               0.1843200 sec
RG                   172.3
DW                  90.000 usec
DE                   30.00 usec
TE                   295.0 K
D0              0.00007981 sec
D1              1.93446398 sec
D11             0.03000000 sec
D12             0.00002000 sec
D16             0.00020000 sec
IN0             0.00018000 sec

======== CHANNEL f1 ========
SFO1           700.1732699 MHz
NUC1                    1H
P1                    8.00 usec
P2                   16.00 usec
P17                2500.00 usec
PLW1            9.64999962 W
PLW10           0.91360998 W

====== GRADIENT CHANNEL =====
GPNAM[1]       SMSQ10.100
GPNAM[2]       SMSQ10.100
GPZ1                 10.00 %
GPZ2                 20.00 %
P16                1000.00 usec

F1 - Acquisition parameters
TD                     256
SFO1              700.1733 MHz
FIDRES           21.701389 Hz
SW                   7.935 ppm
FnMODE          States-TPPI

F2 - Processing parameters
SI                    1024
SF             700.1700000 MHz
WDW                  QSINE
SSB                      2
LB           0 Hz
GB           0
PC                    1.40

F1 - Processing parameters
SI                    1024
MC2             States-TPPI
SF             700.1700000 MHz
WDW                  QSINE
SSB                      2
LB           0 Hz
GB           0
```

COSYGPDFPHSW CDCl3 {C:\Bruker\TOPSPIN} abari 38

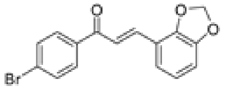

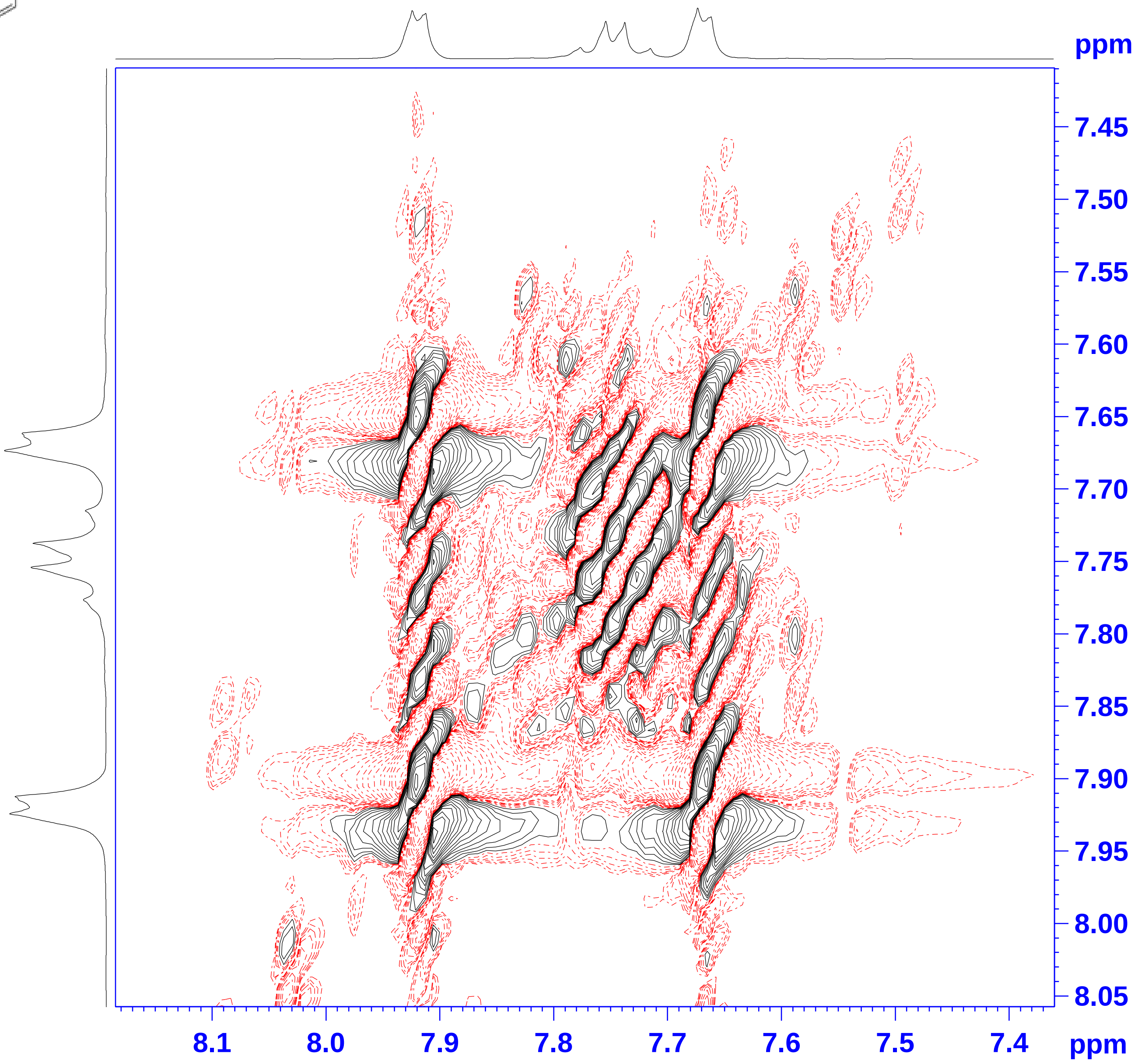


```
Current Data Parameters
NAME          QatarUni-D-5
EXPNO                   13
PROCNO                   1

F2 - Acquisition Parameters
Date_             20160517
Time                 18.23
INSTRUM              spect
PROBHD    5 mm CPTCI 1H-
PULPROG        cosygpmfphpp
TD                    2048
SOLVENT              CDCl3
NS                      32
DS                       4
SWH               5555.556 Hz
FIDRES            2.712674 Hz
AQ               0.1843200 sec
RG                   172.3
DW                  90.000 usec
DE                   30.00 usec
TE                   295.0 K
D0              0.00007981 sec
D1              1.93446398 sec
D11             0.03000000 sec
D12             0.00002000 sec
D16             0.00020000 sec
IN0             0.00018000 sec

======== CHANNEL f1 ========
SFO1          700.1732699 MHz
NUC1                    1H
P1                    8.00 usec
P2                   16.00 usec
P17                2500.00 usec
PLW1            9.64999962 W
PLW10           0.91360998 W

====== GRADIENT CHANNEL =====
GPNAM[1]        SMSQ10.100
GPNAM[2]        SMSQ10.100
GPZ1                 10.00 %
GPZ2                 20.00 %
P16                1000.00 usec

F1 - Acquisition parameters
TD                     256
SFO1              700.1733 MHz
FIDRES           21.701389 Hz
SW                   7.935 ppm
FnMODE          States-TPPI

F2 - Processing parameters
SI                    1024
SF             700.1700000 MHz
WDW                  QSINE
SSB                      2
LB        0 Hz
GB        0
PC                    1.40

F1 - Processing parameters
SI                    1024
MC2             States-TPPI
SF             700.1700000 MHz
WDW                  QSINE
SSB                      2
LB        0 Hz
GB        0
```

COSYGPDFPHSW CDCl3 {C:\Bruker\TOPSPIN} abari 38

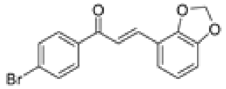

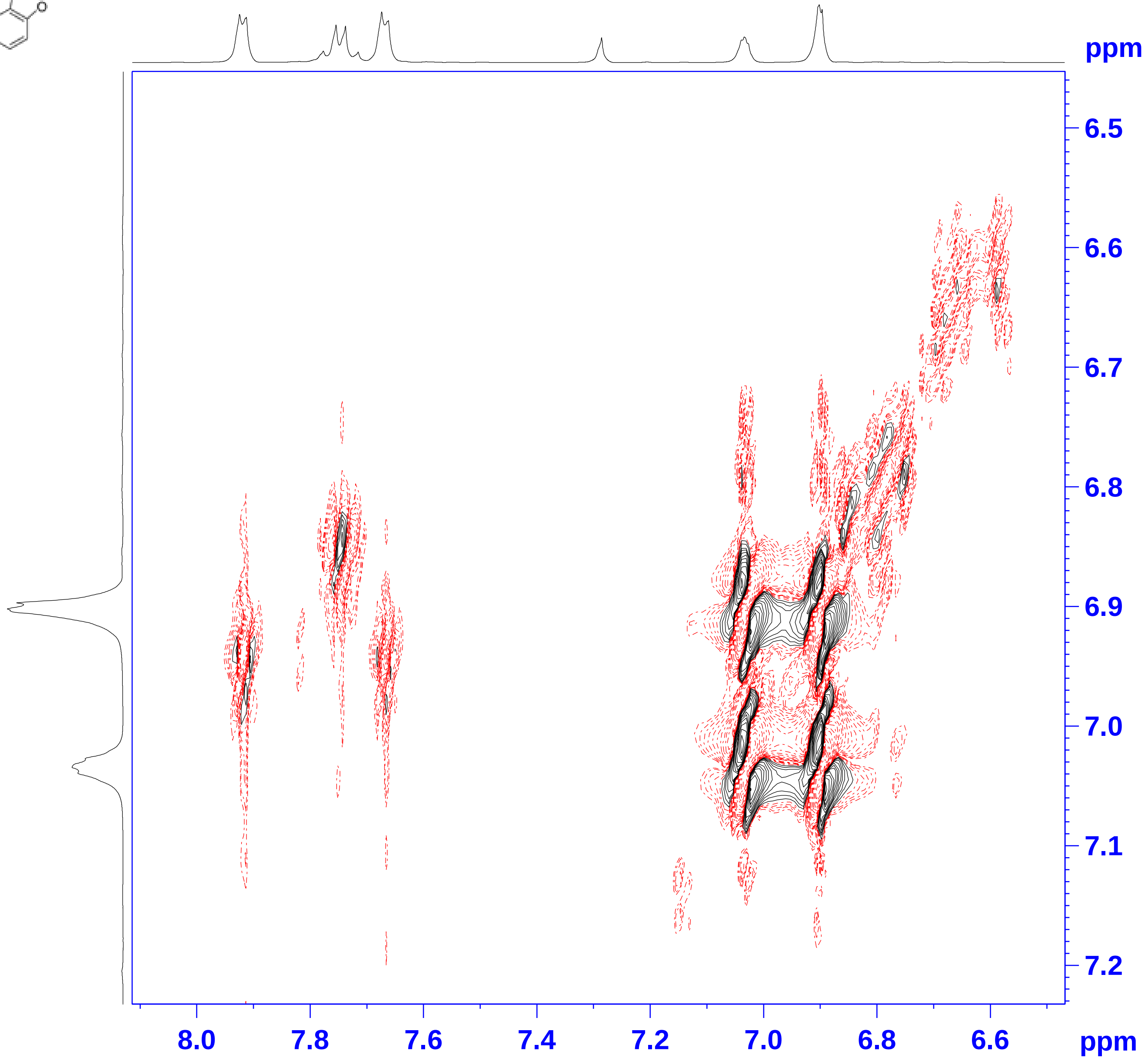


```
Current Data Parameters
NAME          QatarUni-D-5
EXPNO                   13
PROCNO                   1

F2 - Acquisition Parameters
Date_             20160517
Time                 18.23
INSTRUM              spect
PROBHD    5 mm CPTCI 1H-
PULPROG      cosygpmfphpp
TD                    2048
SOLVENT              CDCl3
NS                      32
DS                       4
SWH               5555.556 Hz
FIDRES            2.712674 Hz
AQ               0.1843200 sec
RG                   172.3
DW                  90.000 usec
DE                   30.00 usec
TE                   295.0 K
D0              0.00007981 sec
D1              1.93446398 sec
D11             0.03000000 sec
D12             0.00002000 sec
D16             0.00020000 sec
IN0             0.00018000 sec

======== CHANNEL f1 ========
SFO1           700.1732699 MHz
NUC1                    1H
P1                    8.00 usec
P2                   16.00 usec
P17                2500.00 usec
PLW1            9.64999962 W
PLW10           0.91360998 W

====== GRADIENT CHANNEL =====
GPNAM[1]       SMSQ10.100
GPNAM[2]       SMSQ10.100
GPZ1                 10.00 %
GPZ2                 20.00 %
P16                1000.00 usec

F1 - Acquisition parameters
TD                     256
SFO1              700.1733 MHz
FIDRES           21.701389 Hz
SW                   7.935 ppm
FnMODE         States-TPPI

F2 - Processing parameters
SI                    1024
SF             700.1700000 MHz
WDW                  QSINE
SSB                      2
LB         0 Hz
GB         0
PC                    1.40

F1 - Processing parameters
SI                    1024
MC2            States-TPPI
SF             700.1700000 MHz
WDW                  QSINE
SSB                      2
LB         0 Hz
GB         0
```

COSYGPDFPHSW CDCl3 {C:\Bruker\TOPSPIN} abari 38

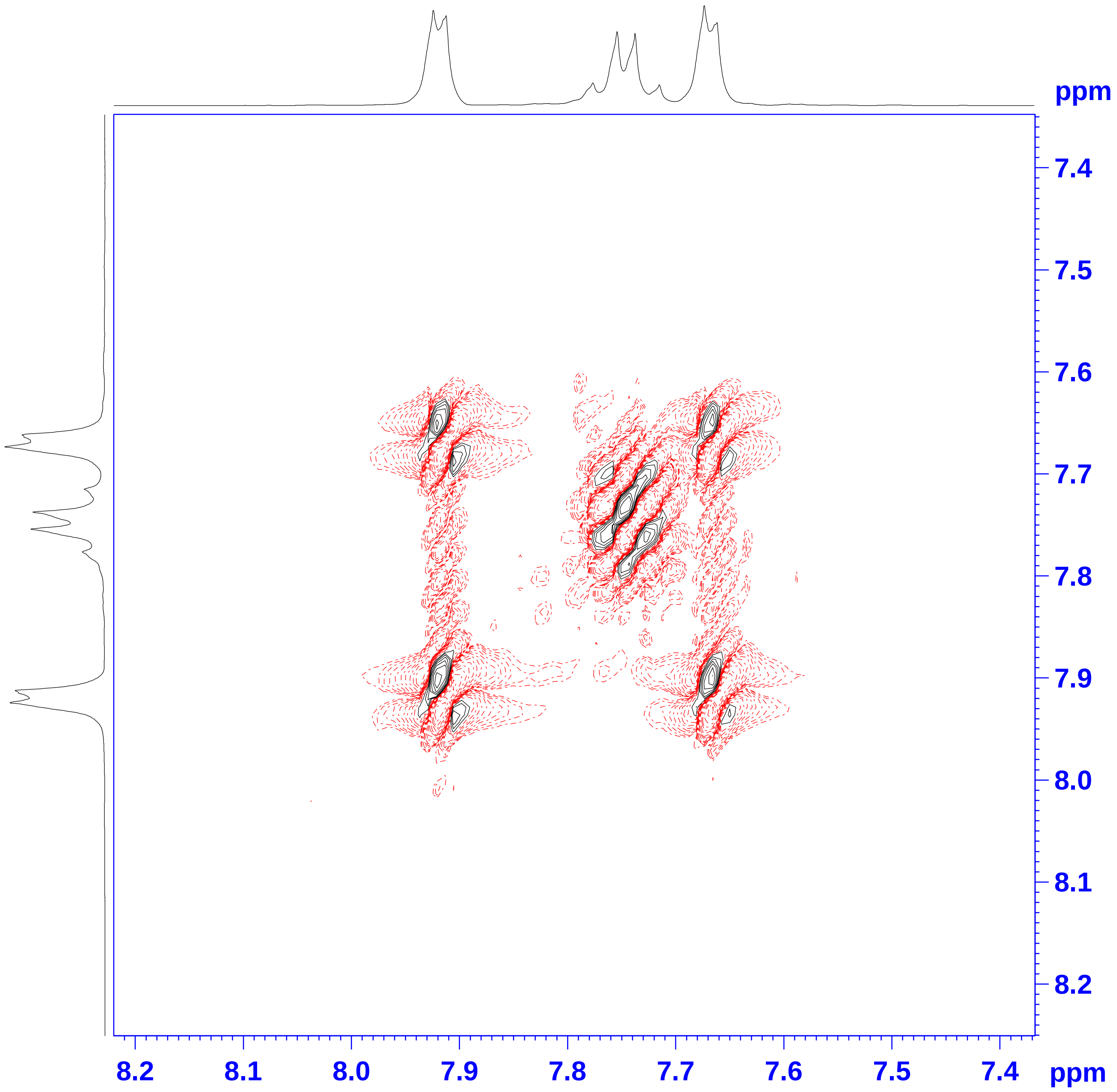

Current Data Parameters
NAME QatarUni-D-5
EXPNO 13
PROCNO 1

F2 - Acquisition Parameters
Date_ 20160517
Time 18.23
INSTRUM spect
PROBHD 5 mm CPTCI 1H-
PULPROG cosygpmfphpp
TD 2048
SOLVENT CDCl3
NS 32
DS 4
SWH 5555.556 Hz
FIDRES 2.712674 Hz
AQ 0.1843200 sec
RG 172.3
DW 90.000 usec
DE 30.00 usec
TE 295.0 K
D0 0.00007981 sec
D1 1.93446398 sec
D11 0.03000000 sec
D12 0.00002000 sec
D16 0.00020000 sec
IN0 0.00018000 sec

======== CHANNEL f1 ========
SFO1 700.1732699 MHz
NUC1 1H
P1 8.00 usec
P2 16.00 usec
P17 2500.00 usec
PLW1 9.64999962 W
PLW10 0.91360998 W

====== GRADIENT CHANNEL =====
GPNAM[1] SMSQ10.100
GPNAM[2] SMSQ10.100
GPZ1 10.00 %
GPZ2 20.00 %
P16 1000.00 usec

F1 - Acquisition parameters
TD 256
SFO1 700.1733 MHz
FIDRES 21.701389 Hz
SW 7.935 ppm
FnMODE States-TPPI

F2 - Processing parameters
SI 1024
SF 700.1700000 MHz
WDW QSINE
SSB 2
LB 0 Hz
GB 0
PC 1.40

F1 - Processing parameters
SI 1024
MC2 States-TPPI
SF 700.1700000 MHz
WDW QSINE
SSB 2
LB 0 Hz
GB 0

COSYGPDFPHSW CDCl3 {C:\Bruker\TOPSPIN} abari 38

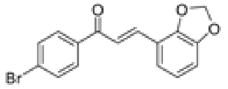

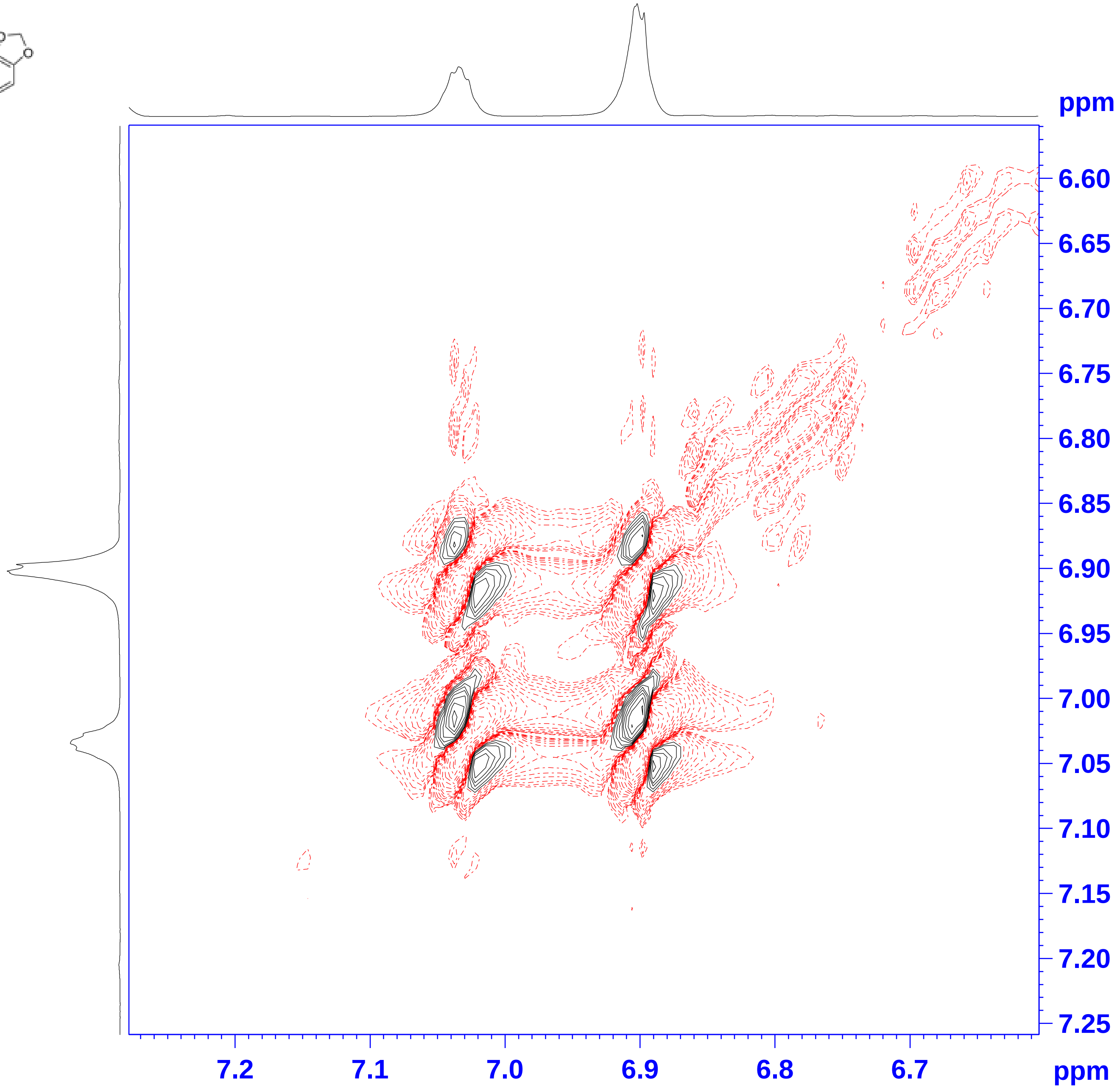


```
Current Data Parameters
NAME          QatarUni-D-5
EXPNO                   13
PROCNO                   1

F2 - Acquisition Parameters
Date_             20160517
Time                 18.23
INSTRUM              spect
PROBHD    5 mm CPTCI 1H-
PULPROG      cosygpmfphpp
TD                    2048
SOLVENT              CDCl3
NS                      32
DS                       4
SWH               5555.556 Hz
FIDRES            2.712674 Hz
AQ               0.1843200 sec
RG                   172.3
DW                  90.000 usec
DE                   30.00 usec
TE                   295.0 K
D0              0.00007981 sec
D1              1.93446398 sec
D11             0.03000000 sec
D12             0.00002000 sec
D16             0.00020000 sec
IN0             0.00018000 sec

======== CHANNEL f1 ========
SFO1           700.1732699 MHz
NUC1                    1H
P1                    8.00 usec
P2                   16.00 usec
P17                2500.00 usec
PLW1            9.64999962 W
PLW10           0.91360998 W

====== GRADIENT CHANNEL =====
GPNAM[1]        SMSQ10.100
GPNAM[2]        SMSQ10.100
GPZ1                 10.00 %
GPZ2                 20.00 %
P16                1000.00 usec

F1 - Acquisition parameters
TD                     256
SFO1              700.1733 MHz
FIDRES           21.701389 Hz
SW                   7.935 ppm
FnMODE          States-TPPI

F2 - Processing parameters
SI                    1024
SF             700.1700000 MHz
WDW                  QSINE
SSB                      2
LB            0 Hz
GB            0
PC                    1.40

F1 - Processing parameters
SI                    1024
MC2             States-TPPI
SF             700.1700000 MHz
WDW                  QSINE
SSB                      2
LB            0 Hz
GB            0
```

C13DEPT135 CDCl3 {C:\Bruker\TOPSPIN} abari 38

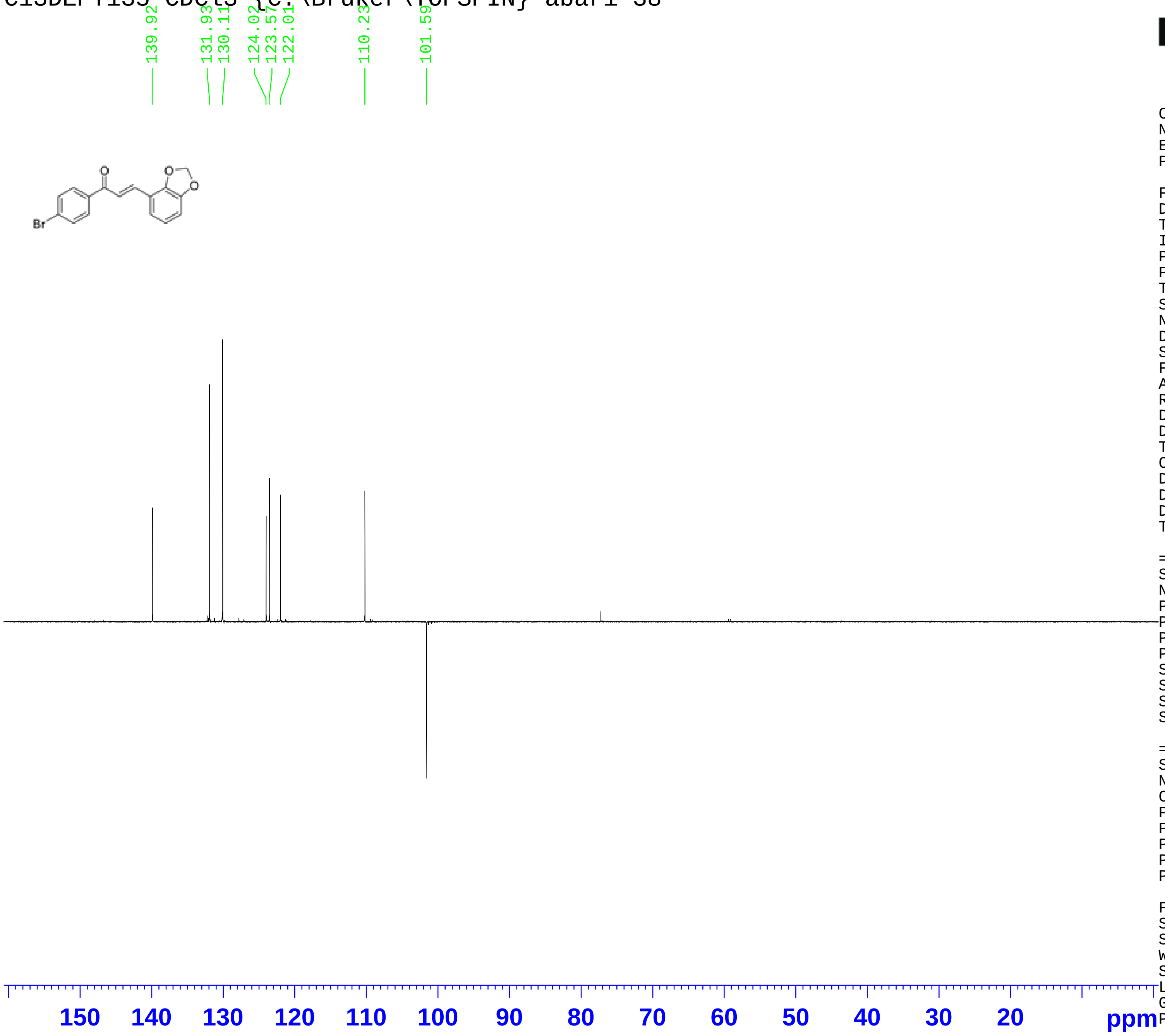

Current Data Parameters
NAME QatarUni-D-5
EXPNO 12
PROCNO 1

F2 - Acquisition Parameters
Date_ 20160517
Time 18.21
INSTRUM spect
PROBHD 5 mm CPTCI 1H-
PULPROG deptsp135
TD 65536
SOLVENT CDCl3
NS 1024
DS 4
SWH 28409.092 Hz
FIDRES 0.433488 Hz
AQ 1.1534336 sec
RG 105.16
DW 17.600 usec
DE 18.00 usec
TE 295.0 K
CNST2 145.0000000
D1 2.00000000 sec
D2 0.00344828 sec
D12 0.00002000 sec
TD0 1

======== CHANNEL f1 ========
SFO1 176.0719703 MHz
NUC1 13C
P1 12.00 usec
P13 2000.00 usec
PLW0 0 W
PLW1 121.00000000 W
SPNAM[5] Crp60comp.4
SPOAL5 0.500
SPOFFS5 0 Hz
SPW5 26.62199974 W

======== CHANNEL f2 ========
SFO2 700.1722392 MHz
NUC2 1H
CPDPRG[2 waltz16
P3 8.00 usec
P4 16.00 usec
PCPD2 65.00 usec
PLW2 9.64999962 W
PLW12 0.14618000 W

F2 - Processing parameters
SI 32768
SF 176.0578870 MHz
WDW EM
SSB 0
LB 1.00 Hz
GB 0
PC 1.40

C13DEPT135 CDCl3 {C:\Bruker\TOPSPIN} abari 38

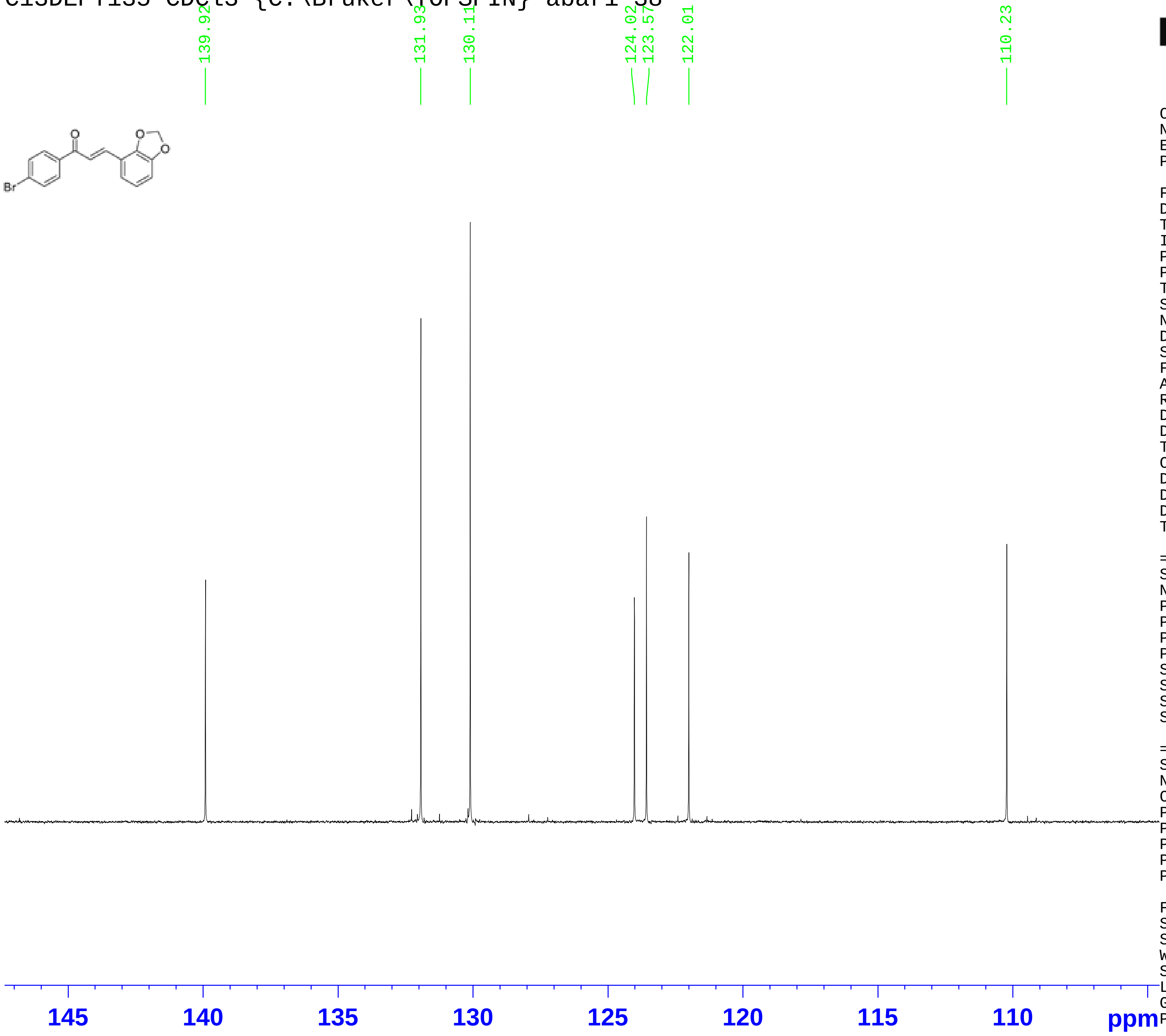


```
Current Data Parameters
NAME        QatarUni-D-5
EXPNO                 12
PROCNO                 1

F2 - Acquisition Parameters
Date_           20160517
Time               18.21
INSTRUM            spect
PROBHD    5 mm CPTCI 1H-
PULPROG        deptsp135
TD                 65536
SOLVENT            CDCl3
NS                  1024
DS                     4
SWH            28409.092 Hz
FIDRES          0.433488 Hz
AQ             1.1534336 sec
RG                105.16
DW                17.600 usec
DE                 18.00 usec
TE                 295.0 K
CNST2        145.0000000
D1            2.00000000 sec
D2            0.00344828 sec
D12           0.00002000 sec
TD0                    1

======== CHANNEL f1 ========
SFO1         176.0719703 MHz
NUC1                 13C
P1                 12.00 usec
P13              2000.00 usec
PLW0      0 W
PLW1        121.00000000 W
SPNAM[5]     Crp60comp.4
SPOAL5             0.500
SPOFFS5   0 Hz
SPW5         26.62199974 W

======== CHANNEL f2 ========
SFO2         700.1722392 MHz
NUC2                  1H
CPDPRG[2         waltz16
P3                  8.00 usec
P4                 16.00 usec
PCPD2              65.00 usec
PLW2          9.64999962 W
PLW12         0.14618000 W

F2 - Processing parameters
SI                 32768
SF           176.0578870 MHz
WDW                   EM
SSB       0
LB                  1.00 Hz
GB        0
PC                  1.40
```

HMBCGP CDCl3 {C:\Bruker\TOPSPIN} abari 38

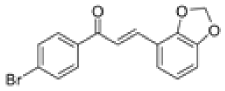

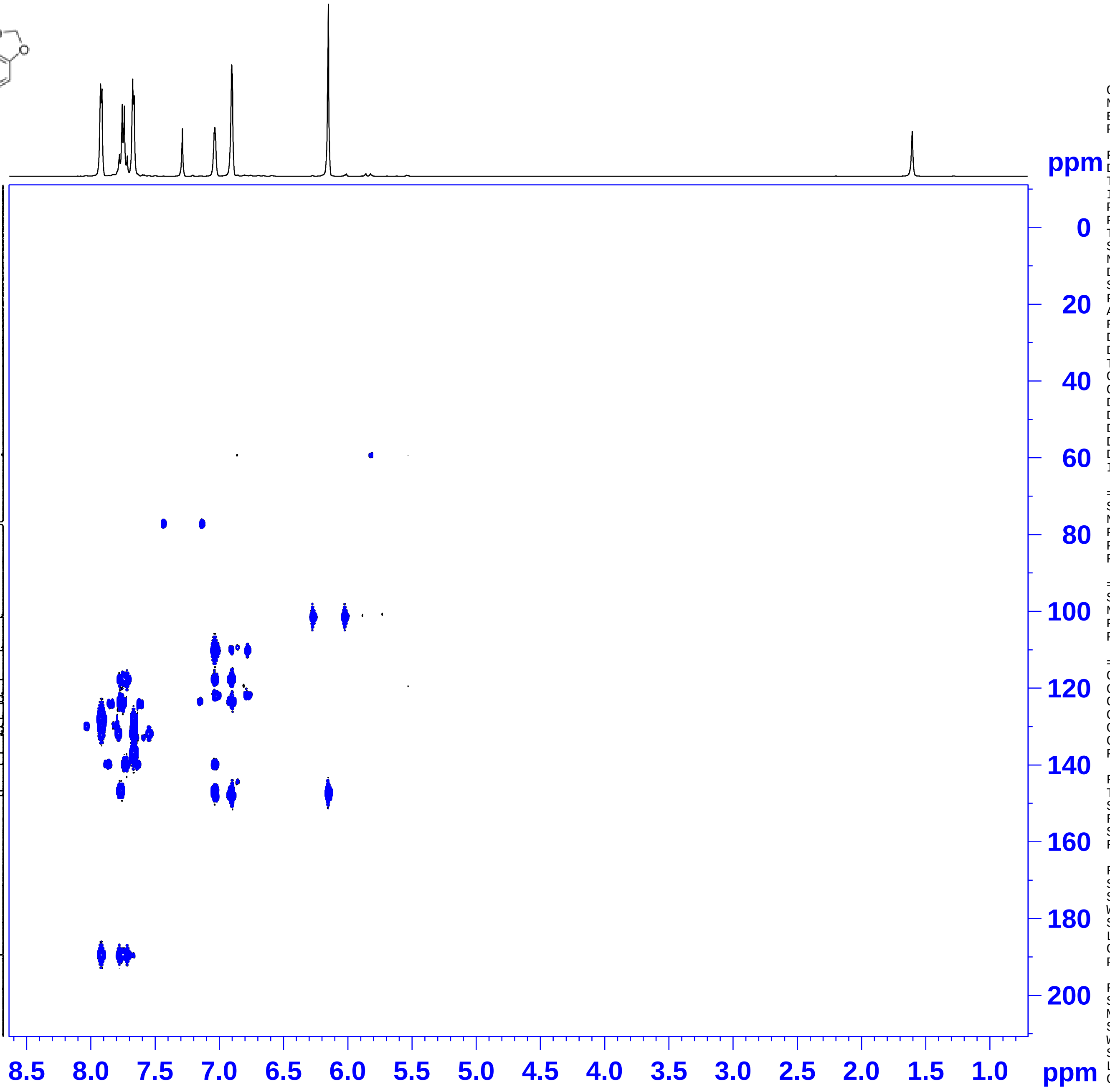


```
Current Data Parameters
NAME        QatarUni-D-5
EXPNO                 14
PROCNO                 1

F2 - Acquisition Parameters
Date_           20160517
Time               23.21
INSTRUM            spect
PROBHD   5 mm CPTCI 1H-
PULPROG    hmbcgplpndqf
TD                  2048
SOLVENT            CDCl3
NS                    32
DS                    16
SWH             5555.556 Hz
FIDRES          2.712674 Hz
AQ             0.1843200 sec
RG                 172.3
DW                90.000 usec
DE                 30.00 usec
TE                 295.0 K
CNST2        145.0000000
CNST13        10.0000000
D0            0.00000300 sec
D1            1.42832005 sec
D2            0.00344828 sec
D6            0.05000000 sec
D16           0.00020000 sec
IN0           0.00001280 sec

======== CHANNEL f1 ========
SFO1         700.1732699 MHz
NUC1                  1H
P1                  8.00 usec
P2                 16.00 usec
PLW1          9.64999962 W

======== CHANNEL f2 ========
SFO2         176.0754635 MHz
NUC2                 13C
P3                 12.00 usec
PLW2        121.00000000 W

====== GRADIENT CHANNEL =====
GPNAM[1]      SMSQ10.100
GPNAM[2]      SMSQ10.100
GPNAM[3]      SMSQ10.100
GPZ1               50.00 %
GPZ2               30.00 %
GPZ3               40.10 %
P16              1000.00 usec

F1 - Acquisition parameters
TD                   128
SFO1            176.0755 MHz
FIDRES        305.175781 Hz
SW               221.851 ppm
FnMODE                QF

F2 - Processing parameters
SI                  2048
SF           700.1700000 MHz
WDW                 SINE
SSB       0
LB        0 Hz
GB        0
PC                  1.40

F1 - Processing parameters
SI                  1024
MC2                   QF
SF           176.0578870 MHz
WDW                 SINE
SSB       0
LB        0 Hz
GB        0
```

HMBCGP CDCl3 {C:\Bruker\TOPSPIN} abari 38

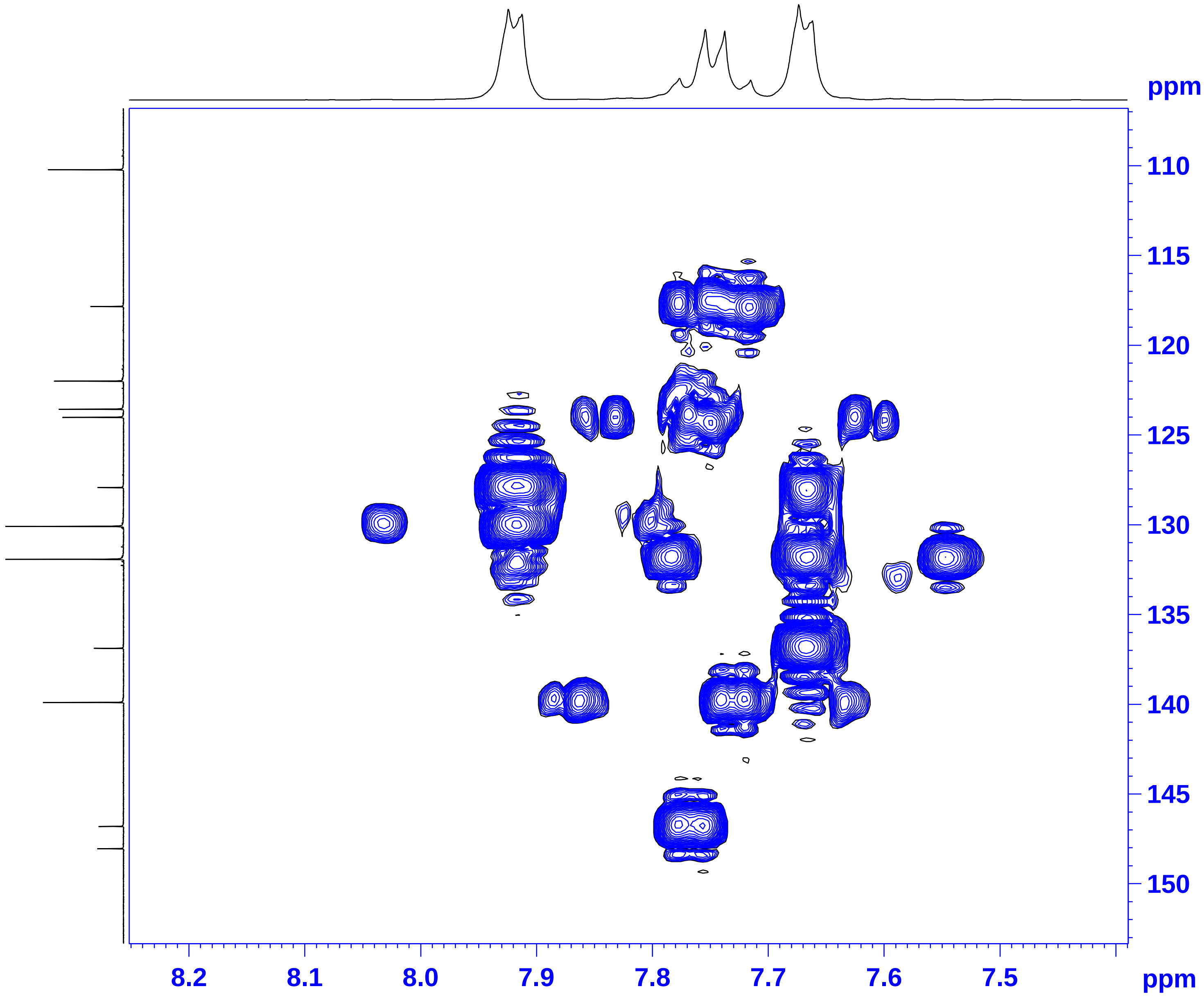


```
Current Data Parameters
NAME          QatarUni-D-5
EXPNO                   14
PROCNO                   1

F2 - Acquisition Parameters
Date_             20160517
Time                 23.21
INSTRUM              spect
PROBHD   5 mm CPTCI 1H-
PULPROG    hmbcgplpndqf
TD                    2048
SOLVENT              CDCl3
NS                      32
DS                      16
SWH              5555.556 Hz
FIDRES           2.712674 Hz
AQ              0.1843200 sec
RG                   172.3
DW                  90.000 usec
DE                   30.00 usec
TE                   295.0 K
CNST2         145.0000000
CNST13         10.0000000
D0             0.00000300 sec
D1             1.42832005 sec
D2             0.00344828 sec
D6             0.05000000 sec
D16            0.00020000 sec
IN0            0.00001280 sec

======== CHANNEL f1 ========
SFO1         700.1732699 MHz
NUC1                    1H
P1                    8.00 usec
P2                   16.00 usec
PLW1            9.64999962 W

======== CHANNEL f2 ========
SFO2         176.0754635 MHz
NUC2                   13C
P3                   12.00 usec
PLW2          121.00000000 W

====== GRADIENT CHANNEL =====
GPNAM[1]      SMSQ10.100
GPNAM[2]      SMSQ10.100
GPNAM[3]      SMSQ10.100
GPZ1                 50.00 %
GPZ2                 30.00 %
GPZ3                 40.10 %
P16                1000.00 usec

F1 - Acquisition parameters
TD                     128
SFO1           176.0755 MHz
FIDRES       305.175781 Hz
SW              221.851 ppm
FnMODE                  QF

F2 - Processing parameters
SI                    2048
SF           700.1700000 MHz
WDW                   SINE
SSB      0
LB       0 Hz
GB       0
PC                    1.40

F1 - Processing parameters
SI                    1024
MC2                     QF
SF           176.0578870 MHz
WDW                   SINE
SSB      0
LB       0 Hz
GB       0
```

HMBCGP CDCl3 {C:\Bruker\TOPSPIN} abari 38

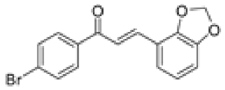

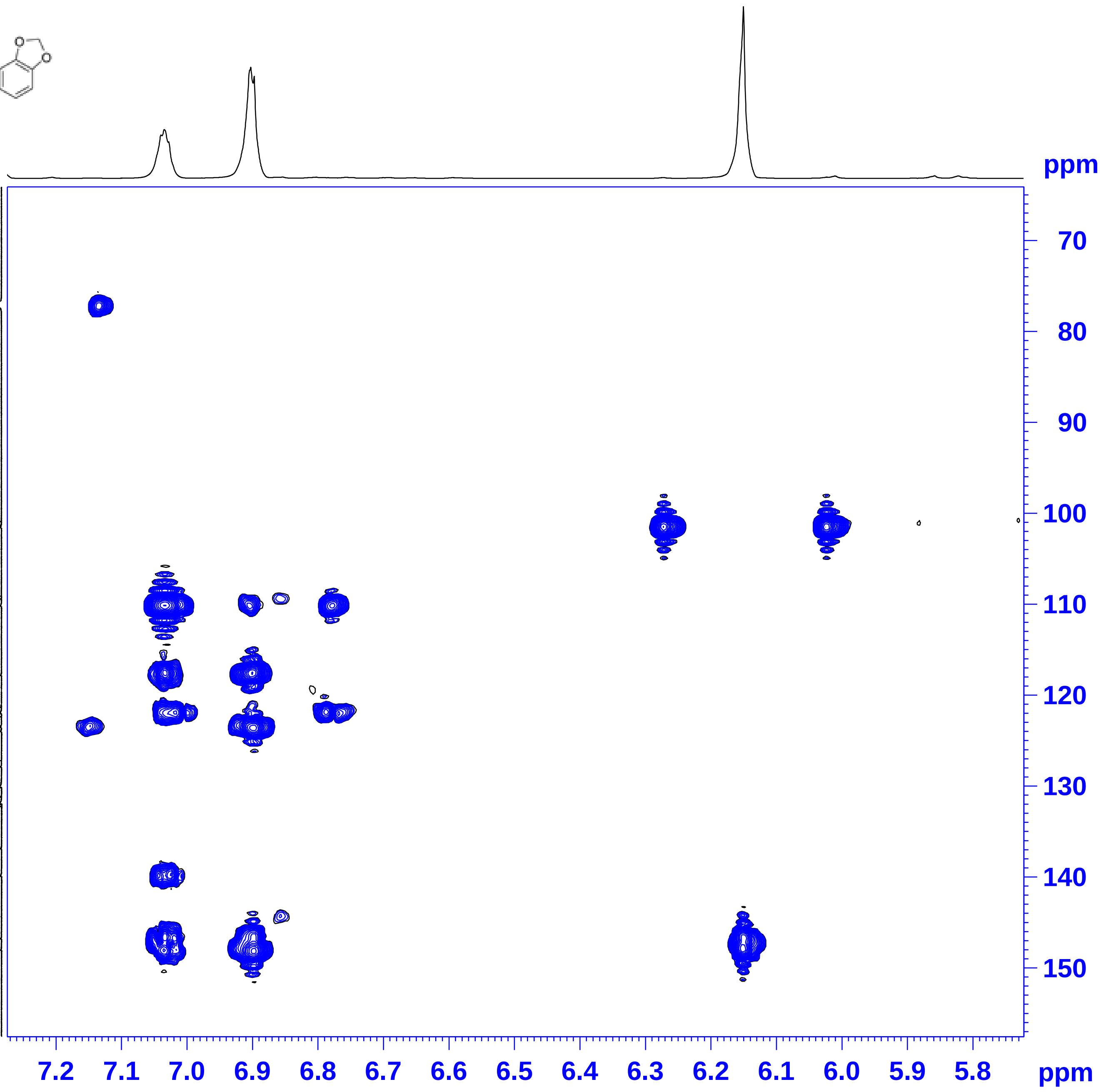


```
Current Data Parameters
NAME        QatarUni-D-5
EXPNO                 14
PROCNO                 1

F2 - Acquisition Parameters
Date_           20160517
Time               23.21
INSTRUM            spect
PROBHD   5 mm CPTCI 1H-
PULPROG    hmbcgplpndqf
TD                  2048
SOLVENT            CDCl3
NS                    32
DS                    16
SWH             5555.556 Hz
FIDRES          2.712674 Hz
AQ             0.1843200 sec
RG                 172.3
DW                90.000 usec
DE                 30.00 usec
TE                 295.0 K
CNST2        145.0000000
CNST13        10.0000000
D0            0.00000300 sec
D1            1.42832005 sec
D2            0.00344828 sec
D6            0.05000000 sec
D16           0.00020000 sec
IN0           0.00001280 sec

======== CHANNEL f1 ========
SFO1         700.1732699 MHz
NUC1                  1H
P1                  8.00 usec
P2                 16.00 usec
PLW1          9.64999962 W

======== CHANNEL f2 ========
SFO2         176.0754635 MHz
NUC2                 13C
P3                 12.00 usec
PLW2        121.00000000 W

====== GRADIENT CHANNEL =====
GPNAM[1]      SMSQ10.100
GPNAM[2]      SMSQ10.100
GPNAM[3]      SMSQ10.100
GPZ1               50.00 %
GPZ2               30.00 %
GPZ3               40.10 %
P16              1000.00 usec

F1 - Acquisition parameters
TD                   128
SFO1            176.0755 MHz
FIDRES        305.175781 Hz
SW               221.851 ppm
FnMODE                QF

F2 - Processing parameters
SI                  2048
SF           700.1700000 MHz
WDW                 SINE
SSB      0
LB       0 Hz
GB       0
PC                  1.40

F1 - Processing parameters
SI                  1024
MC2                   QF
SF           176.0578870 MHz
WDW                 SINE
SSB      0
LB       0 Hz
GB       0
```

HMBCGP CDCl3 {C:\Bruker\TOPSPIN} abari 38

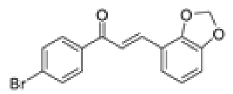

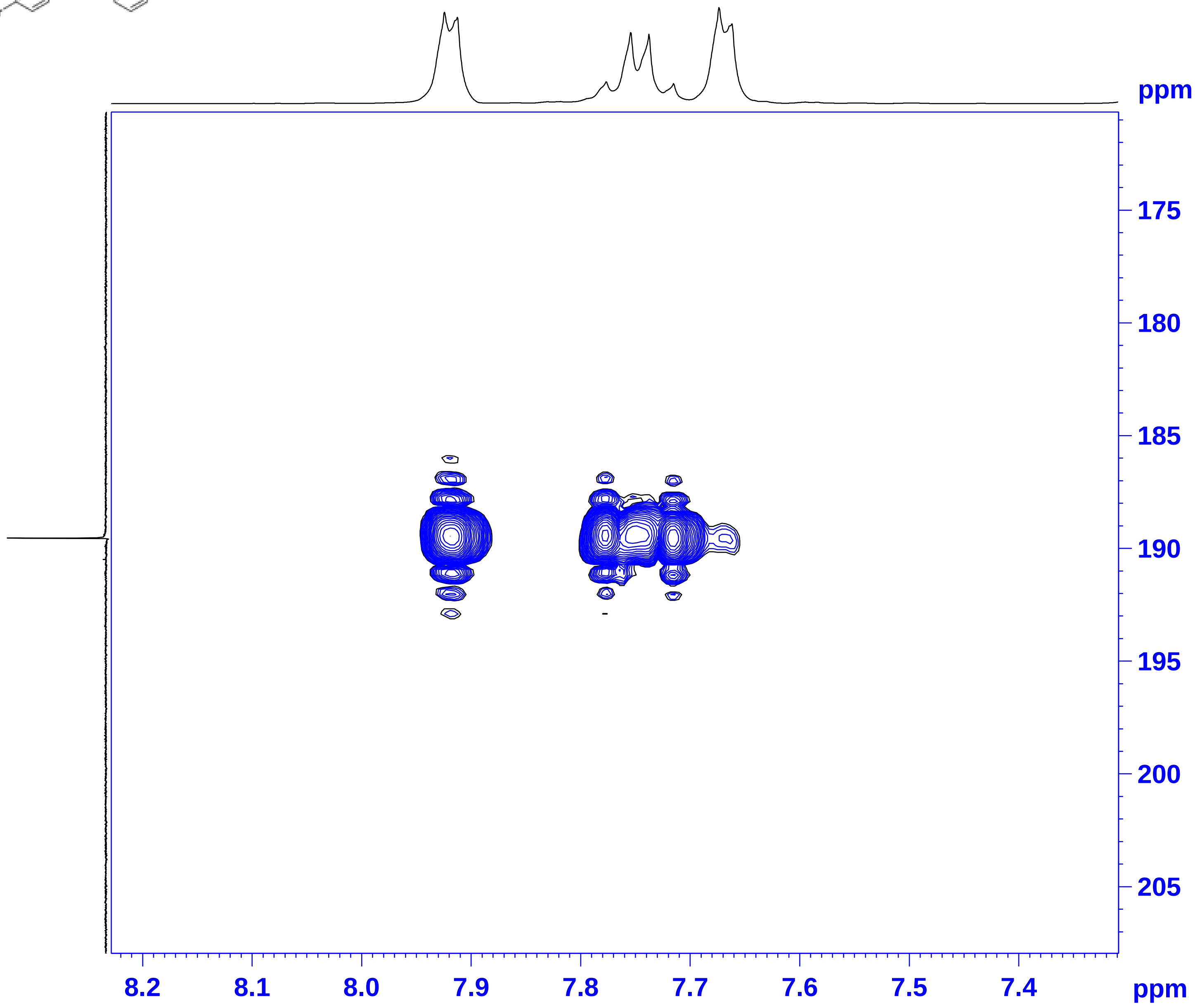


```
Current Data Parameters
NAME        QatarUni-D-5
EXPNO                 14
PROCNO                 1

F2 - Acquisition Parameters
Date_           20160517
Time               23.21
INSTRUM            spect
PROBHD   5 mm CPTCI 1H-
PULPROG    hmbcgplpndqf
TD                  2048
SOLVENT            CDCl3
NS                    32
DS                    16
SWH             5555.556 Hz
FIDRES          2.712674 Hz
AQ             0.1843200 sec
RG                 172.3
DW                90.000 usec
DE                 30.00 usec
TE                 295.0 K
CNST2        145.0000000
CNST13        10.0000000
D0            0.00000300 sec
D1            1.42832005 sec
D2            0.00344828 sec
D6            0.05000000 sec
D16           0.00020000 sec
IN0           0.00001280 sec

======== CHANNEL f1 ========
SFO1         700.1732699 MHz
NUC1                  1H
P1                  8.00 usec
P2                 16.00 usec
PLW1          9.64999962 W

======== CHANNEL f2 ========
SFO2         176.0754635 MHz
NUC2                 13C
P3                 12.00 usec
PLW2        121.00000000 W

====== GRADIENT CHANNEL =====
GPNAM[1]      SMSQ10.100
GPNAM[2]      SMSQ10.100
GPNAM[3]      SMSQ10.100
GPZ1               50.00 %
GPZ2               30.00 %
GPZ3               40.10 %
P16              1000.00 usec

F1 - Acquisition parameters
TD                   128
SFO1            176.0755 MHz
FIDRES        305.175781 Hz
SW               221.851 ppm
FnMODE                QF

F2 - Processing parameters
SI                  2048
SF           700.1700000 MHz
WDW                 SINE
SSB      0
LB       0 Hz
GB       0
PC                  1.40

F1 - Processing parameters
SI                  1024
MC2                   QF
SF           176.0578870 MHz
WDW                 SINE
SSB      0
LB       0 Hz
GB       0
```

HMQCGP CDCl3 {C:\Bruker\TOPSPIN} abari 38

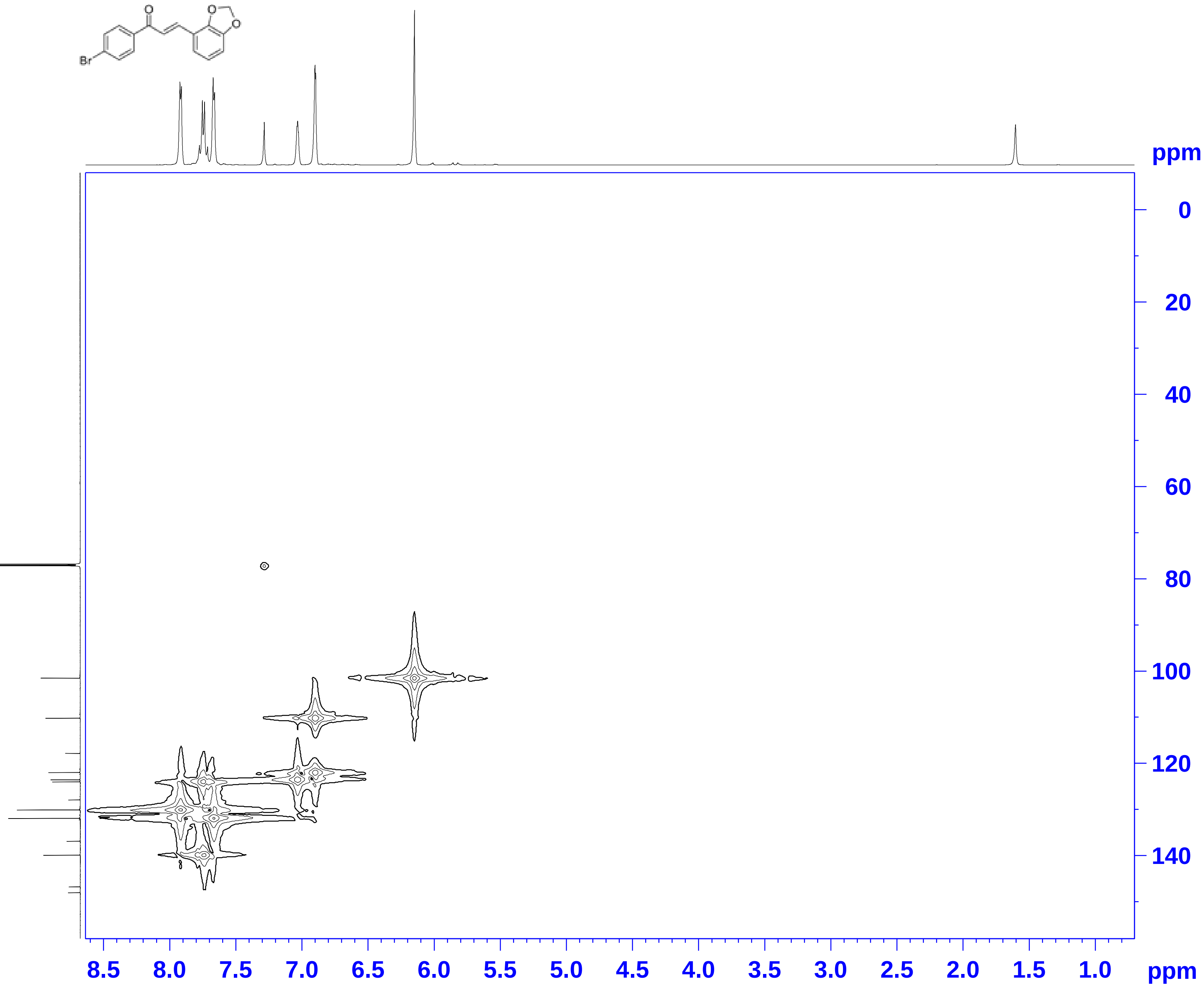


```
Current Data Parameters
NAME        QatarUni-D-5
EXPNO                 15
PROCNO                 1

F2 - Acquisition Parameters
Date_           20160518
Time                1.16
INSTRUM            spect
PROBHD   5 mm CPTCI 1H-
PULPROG         hmqcgpqf
TD                  1024
SOLVENT            CDCl3
NS                     4
DS                    16
SWH             5555.556 Hz
FIDRES          5.425347 Hz
AQ             0.0921600 sec
RG                 172.3
DW                90.000 usec
DE                 30.00 usec
TE                 295.0 K
CNST2        145.0000000
D0            0.00000300 sec
D1            1.48095298 sec
D2            0.00344828 sec
D12           0.00002000 sec
D13           0.00000400 sec
D16           0.00020000 sec
IN0           0.00001710 sec

======== CHANNEL f1 ========
SFO1         700.1732699 MHz
NUC1                  1H
P1                  8.00 usec
P2                 16.00 usec
PLW1          9.64999962 W

======== CHANNEL f2 ========
SFO2         176.0710900 MHz
NUC2                 13C
CPDPRG[2            garp
P3                 12.00 usec
PCPD2              55.00 usec
PLW2        121.00000000 W
PLW12         5.76000023 W

====== GRADIENT CHANNEL =====
GPNAM[1]      SMSQ10.100
GPNAM[2]      SMSQ10.100
GPNAM[3]      SMSQ10.100
GPZ1               50.00 %
GPZ2               30.00 %
GPZ3               40.10 %
P16              1000.00 usec

F1 - Acquisition parameters
TD                   128
SFO1            176.0711 MHz
FIDRES        228.435669 Hz
SW               166.068 ppm
FnMODE                QF

F2 - Processing parameters
SI                  1024
SF           700.1700000 MHz
WDW                QSINE
SSB                    2
LB         0 Hz
GB         0
PC                  1.40

F1 - Processing parameters
SI                  1024
MC2                   QF
SF           176.0578870 MHz
WDW                QSINE
SSB                    2
LB         0 Hz
GB         0
```

HMQCGP CDCl3 {C:\Bruker\TOPSPIN} abari 38

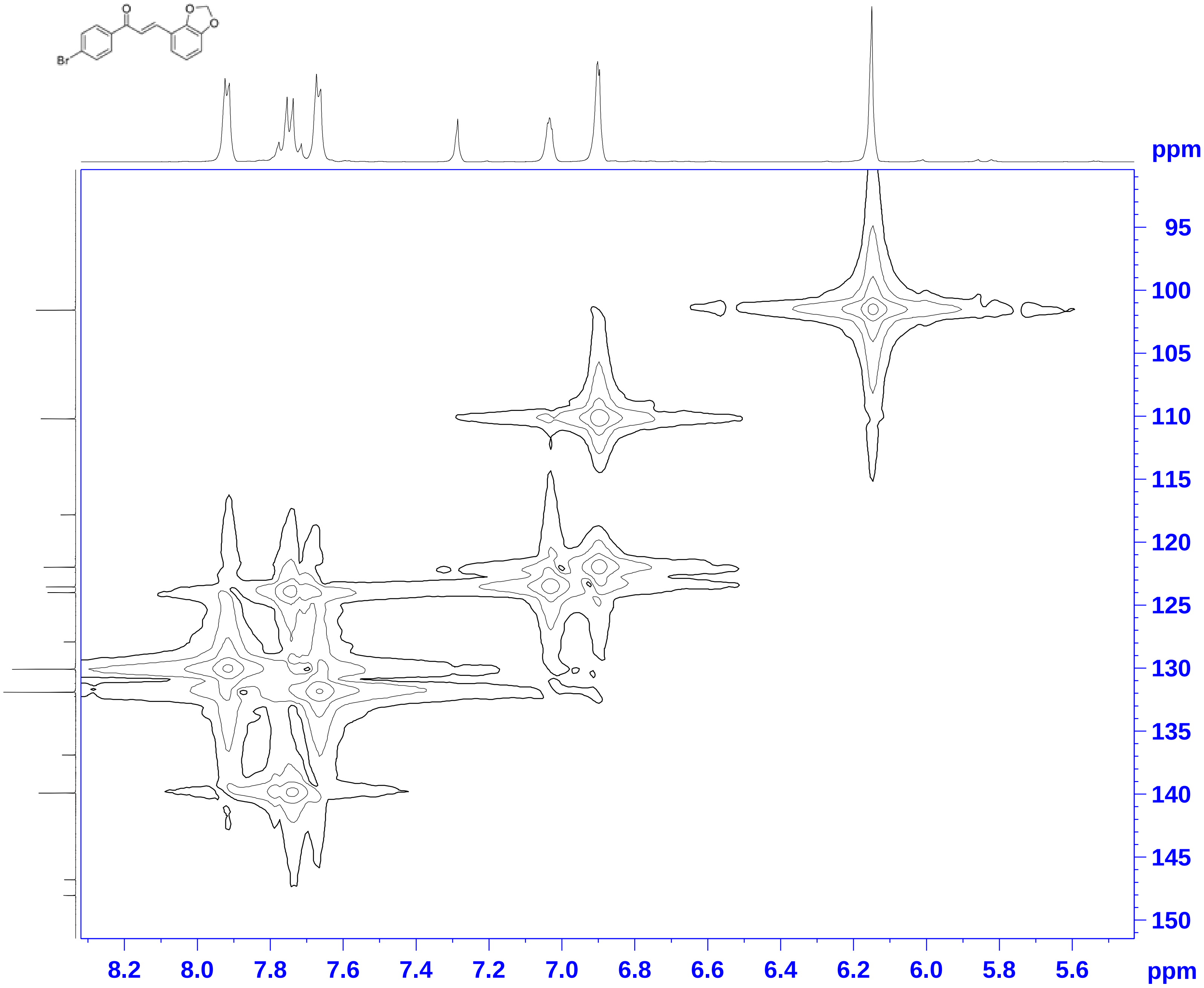


```
Current Data Parameters
NAME        QatarUni-D-5
EXPNO                 15
PROCNO                 1

F2 - Acquisition Parameters
Date_           20160518
Time                1.16
INSTRUM            spect
PROBHD   5 mm CPTCI 1H-
PULPROG         hmqcgpqf
TD                  1024
SOLVENT            CDCl3
NS                     4
DS                    16
SWH             5555.556 Hz
FIDRES          5.425347 Hz
AQ             0.0921600 sec
RG                 172.3
DW                90.000 usec
DE                 30.00 usec
TE                 295.0 K
CNST2        145.0000000
D0            0.00000300 sec
D1            1.48095298 sec
D2            0.00344828 sec
D12           0.00002000 sec
D13           0.00000400 sec
D16           0.00020000 sec
IN0           0.00001710 sec

======== CHANNEL f1 ========
SFO1         700.1732699 MHz
NUC1                  1H
P1                  8.00 usec
P2                 16.00 usec
PLW1          9.64999962 W

======== CHANNEL f2 ========
SFO2         176.0710900 MHz
NUC2                 13C
CPDPRG[2            garp
P3                 12.00 usec
PCPD2              55.00 usec
PLW2        121.00000000 W
PLW12         5.76000023 W

====== GRADIENT CHANNEL =====
GPNAM[1]      SMSQ10.100
GPNAM[2]      SMSQ10.100
GPNAM[3]      SMSQ10.100
GPZ1               50.00 %
GPZ2               30.00 %
GPZ3               40.10 %
P16              1000.00 usec

F1 - Acquisition parameters
TD                   128
SFO1            176.0711 MHz
FIDRES        228.435669 Hz
SW               166.068 ppm
FnMODE                QF

F2 - Processing parameters
SI                  1024
SF           700.1700000 MHz
WDW                QSINE
SSB                    2
LB         0 Hz
GB         0
PC                  1.40

F1 - Processing parameters
SI                  1024
MC2                   QF
SF           176.0578870 MHz
WDW                QSINE
SSB                    2
LB         0 Hz
GB         0
```

HMQCGP CDCl3 {C:\Bruker\TOPSPIN} abari 38

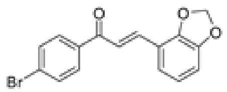

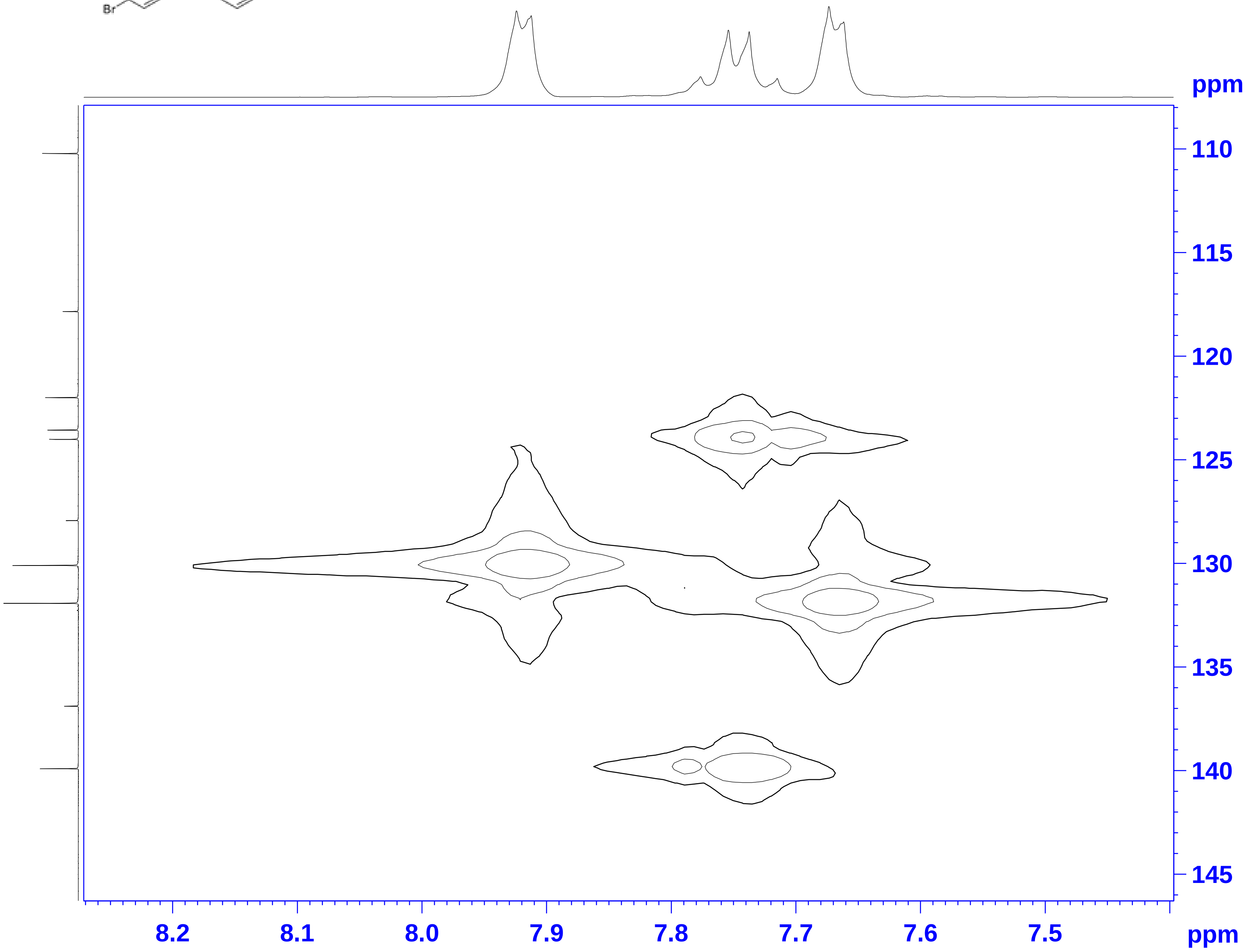


```
Current Data Parameters
NAME        QatarUni-D-5
EXPNO                 15
PROCNO                 1

F2 - Acquisition Parameters
Date_           20160518
Time                1.16
INSTRUM            spect
PROBHD    5 mm CPTCI 1H-
PULPROG         hmqcgpqf
TD                  1024
SOLVENT            CDCl3
NS                     4
DS                    16
SWH             5555.556 Hz
FIDRES          5.425347 Hz
AQ             0.0921600 sec
RG                 172.3
DW                90.000 usec
DE                 30.00 usec
TE                 295.0 K
CNST2        145.0000000
D0            0.00000300 sec
D1            1.48095298 sec
D2            0.00344828 sec
D12           0.00002000 sec
D13           0.00000400 sec
D16           0.00020000 sec
IN0           0.00001710 sec

======== CHANNEL f1 ========
SFO1         700.1732699 MHz
NUC1                  1H
P1                  8.00 usec
P2                 16.00 usec
PLW1          9.64999962 W

======== CHANNEL f2 ========
SFO2         176.0710900 MHz
NUC2                 13C
CPDPRG[2            garp
P3                 12.00 usec
PCPD2              55.00 usec
PLW2        121.00000000 W
PLW12         5.76000023 W

====== GRADIENT CHANNEL =====
GPNAM[1]      SMSQ10.100
GPNAM[2]      SMSQ10.100
GPNAM[3]      SMSQ10.100
GPZ1               50.00 %
GPZ2               30.00 %
GPZ3               40.10 %
P16              1000.00 usec

F1 - Acquisition parameters
TD                   128
SFO1            176.0711 MHz
FIDRES        228.435669 Hz
SW               166.068 ppm
FnMODE                QF

F2 - Processing parameters
SI                  1024
SF           700.1700000 MHz
WDW                QSINE
SSB                    2
LB        0 Hz
GB        0
PC                  1.40

F1 - Processing parameters
SI                  1024
MC2                   QF
SF           176.0578870 MHz
WDW                QSINE
SSB                    2
LB        0 Hz
GB        0
```

HMQCGP CDCl3 {C:\Bruker\TOPSPIN} abari 38

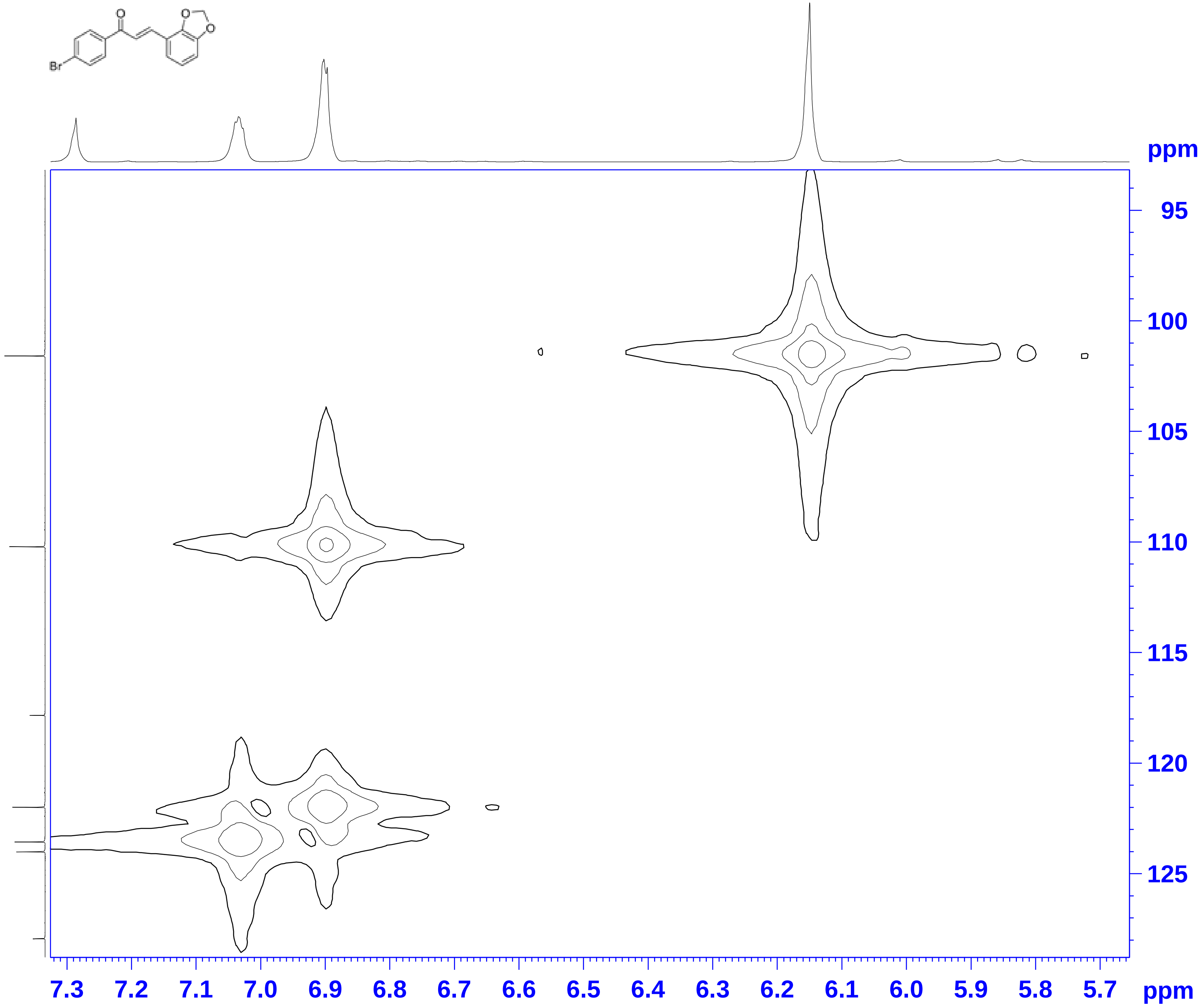

BRUKER

```
Current Data Parameters
NAME        QatarUni-D-5
EXPNO                 15
PROCNO                 1

F2 - Acquisition Parameters
Date_           20160518
Time                1.16
INSTRUM            spect
PROBHD   5 mm CPTCI 1H-
PULPROG         hmqcgpqf
TD                  1024
SOLVENT            CDCl3
NS                     4
DS                    16
SWH             5555.556 Hz
FIDRES          5.425347 Hz
AQ             0.0921600 sec
RG                 172.3
DW                90.000 usec
DE                 30.00 usec
TE                 295.0 K
CNST2        145.0000000
D0            0.00000300 sec
D1            1.48095298 sec
D2            0.00344828 sec
D12           0.00002000 sec
D13           0.00000400 sec
D16           0.00020000 sec
IN0           0.00001710 sec

======== CHANNEL f1 ========
SFO1         700.1732699 MHz
NUC1                  1H
P1                  8.00 usec
P2                 16.00 usec
PLW1          9.64999962 W

======== CHANNEL f2 ========
SFO2         176.0710900 MHz
NUC2                 13C
CPDPRG[2            garp
P3                 12.00 usec
PCPD2              55.00 usec
PLW2        121.00000000 W
PLW12         5.76000023 W

====== GRADIENT CHANNEL =====
GPNAM[1]      SMSQ10.100
GPNAM[2]      SMSQ10.100
GPNAM[3]      SMSQ10.100
GPZ1               50.00 %
GPZ2               30.00 %
GPZ3               40.10 %
P16              1000.00 usec

F1 - Acquisition parameters
TD                   128
SFO1            176.0711 MHz
FIDRES        228.435669 Hz
SW               166.068 ppm
FnMODE                QF

F2 - Processing parameters
SI                  1024
SF           700.1700000 MHz
WDW                QSINE
SSB                    2
LB         0 Hz
GB         0
PC                  1.40

F1 - Processing parameters
SI                  1024
MC2                   QF
SF           176.0578870 MHz
WDW                QSINE
SSB                    2
LB         0 Hz
GB         0
```

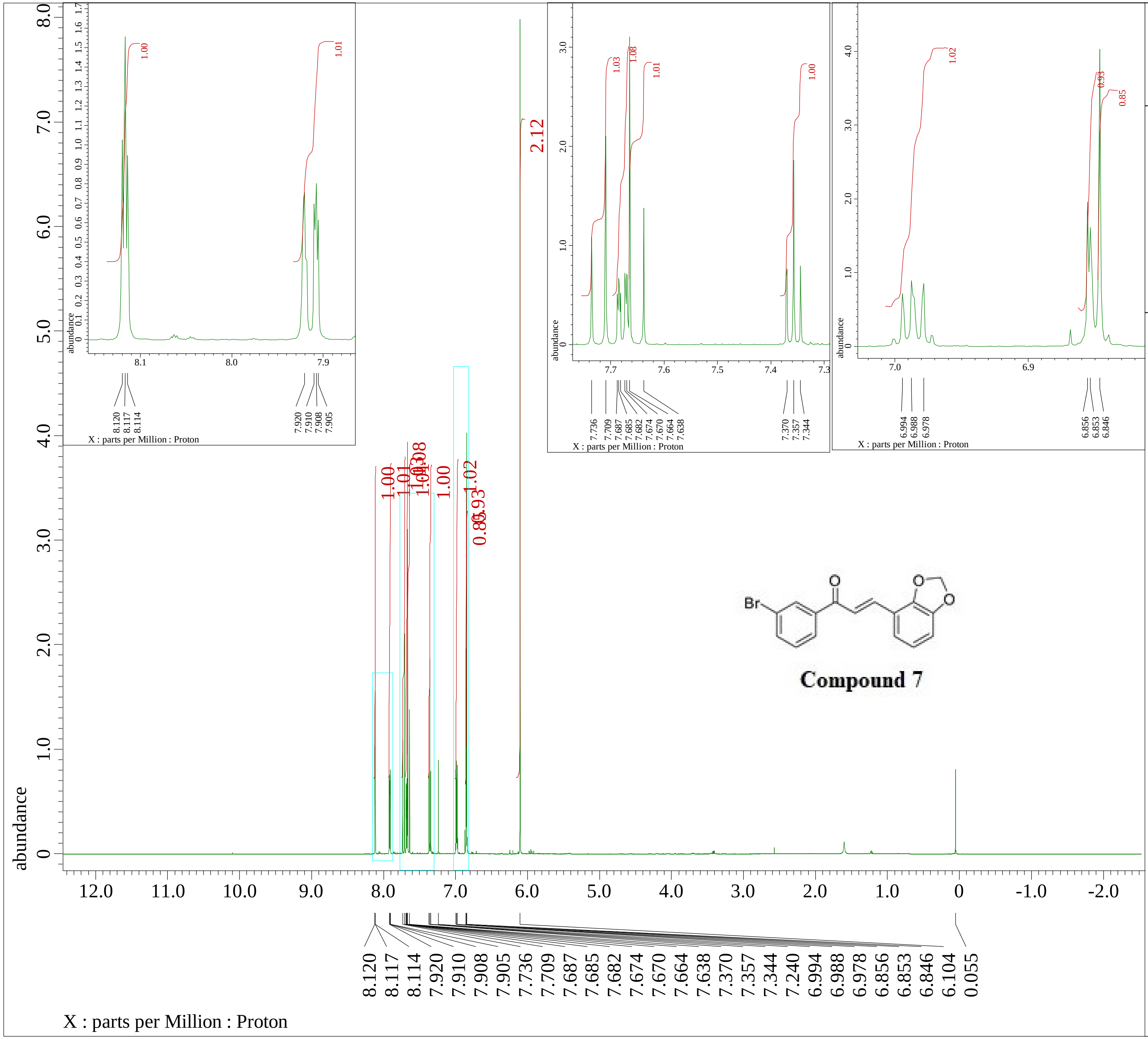


```
---- PROCESSING PARAMETERS ----
sexp( 0.2[Hz], 0.0[s] )
trapezoid( 0[%], 0[%], 80[%], 100[%] )
zerofill( 1 )
fft( 1, TRUE, TRUE )
machinephase
ppm
phase( -0.35742, -1.08529, 42.73615[%] )
phase( -1.05581, 0, 50[%] )

Derived from: CLU-18-106-D33_proton-1-1.jdf
```

```
Filename             = CLU-18-106-D33_proton
Author               = delta
Experiment           = proton.jxp
Sample_Id            = CLU-18-106-D33
Solvent              = CHLOROFORM-D
Actual_Start_Time    = 12-MAR-2018 13:29:45
Revision_Time        = 14-MAR-2018 08:30:35
Comment              = single_pulse
Data_Format          = 1D COMPLEX
Dim_Size             = 13107
Dim_Title            = Proton
Dim_Units            = [ppm]
Dimensions           = X
Spectrometer         = JNM-ECZ600R/S1

Field_Strength       = 14.09636928[T] (600[M
X_Acq_Duration       = 1.45227776[s]
X_Domain             = 1H
X_Freq               = 600.1723046[MHz]
X_Offset             = 5[ppm]
X_Points             = 16384
X_Prescans           = 1
X_Resolution         = 0.68857351[Hz]
X_Sweep              = 11.28158845[kHz]
X_Sweep_Clipped      = 9.02527076[kHz]
Irr_Domain           = Proton
Irr_Freq             = 600.1723046[MHz]
Irr_Offset           = 5[ppm]
Tri_Domain           = Proton
Tri_Freq             = 600.1723046[MHz]
Tri_Offset           = 5[ppm]
Blanking             = 2[us]
Clipped              = FALSE
Scans                = 32
Total_Scans          = 32

Relaxation_Delay     = 5[s]
Recvr_Gain           = 56
Temp_Get             = 22.7[dC]
X_90_Width           = 7.29[us]
X_Acq_Time           = 1.45227776[s]
X_Angle              = 45[deg]
X_Atn                = 7.5[dB]
X_Pulse              = 3.645[us]
Irr_Mode             = Off
Tri_Mode             = Off
Dante_Loop           = 500
Dante_Presat         = FALSE
```

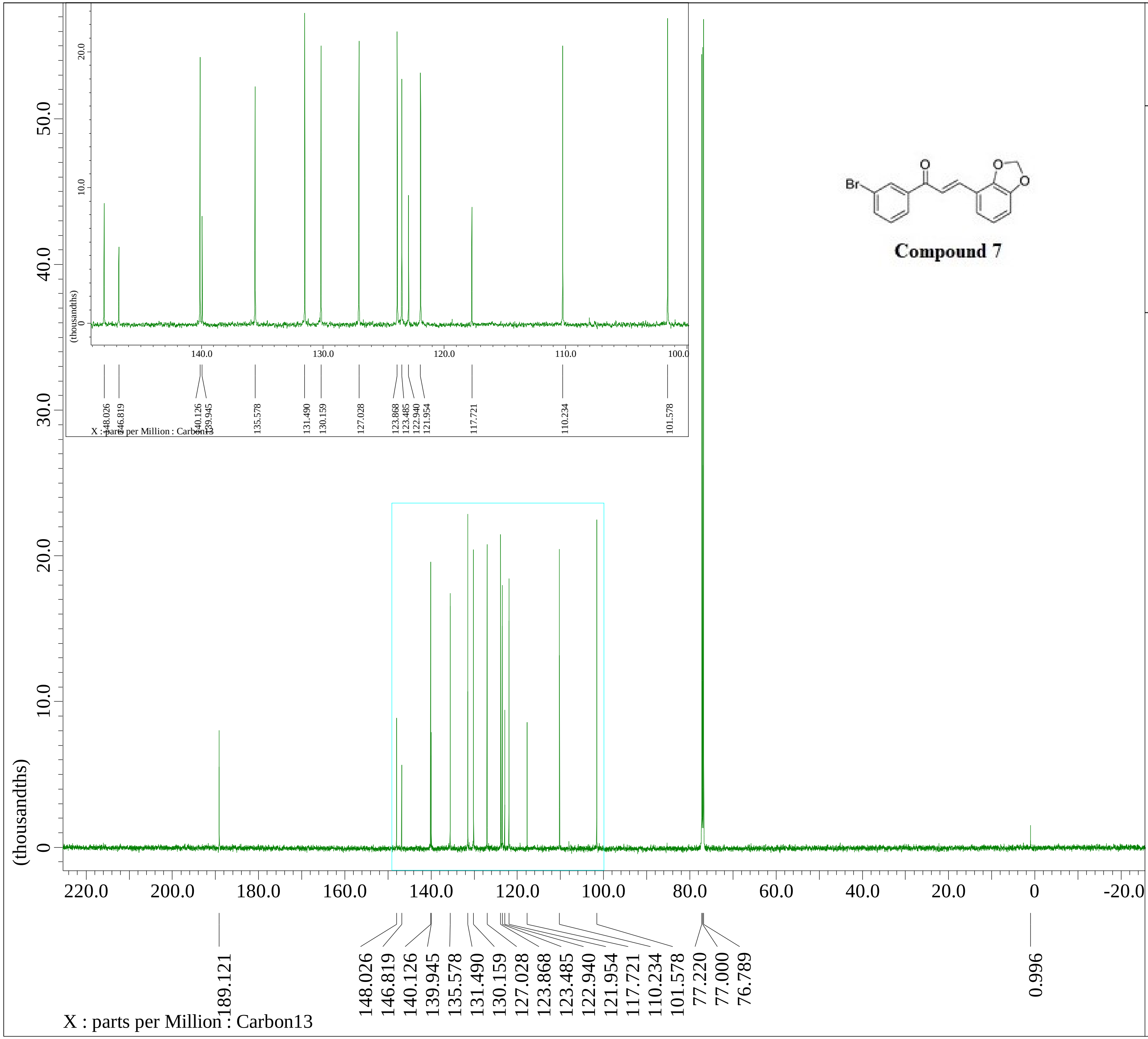

Compound 7

JEOL

```
---- PROCESSING PARAMETERS ----
sexp( 2.0[Hz], 0.0[s] )
trapezoid( 0[%], 0[%], 80[%], 100[%] )
zerofill( 1 )
fft( 1, TRUE, TRUE )
machinephase
ppm

Derived from: CLU-18-106-D33_carbon-1-1.jdf
```

```
Filename                   = CLU-18-106-D33_car
Author                     = delta
Experiment                 = carbon.jxp
Sample_Id                  = CLU-18-106-D33
Solvent                    = CHLOROFORM-D
Actual_Start_Time          = 12-MAR-2018 13:33:
Revision_Time              = 14-MAR-2018 08:45:
Comment                    = single pulse decou
Data_Format                = 1D COMPLEX
Dim_Size                   = 26214
Dim_Title                  = Carbon13
Dim_Units                  = [ppm]
Dimensions                 = X
Spectrometer               = JNM-ECZ600R/S1

Field_Strength             = 14.09636928[T] (60
X_Acq_Duration             = 0.69206016[s]
X_Domain                   = 13C
X_Freq                     = 150.91343039[MHz]
X_Offset                   = 100[ppm]
X_Points                   = 32768
X_Prescans                 = 4
X_Resolution               = 1.44496109[Hz]
X_Sweep                    = 47.34848485[kHz]
X_Sweep_Clipped            = 37.87878788[kHz]
Irr_Domain                 = Proton
Irr_Freq                   = 600.1723046[MHz]
Irr_Offset                 = 5[ppm]
Blanking                   = 2[us]
Clipped                    = TRUE
Scans                      = 1024
Total_Scans                = 1024

Relaxation_Delay           = 2[s]
Recvr_Gain                 = 56
Temp_Get                   = 22.7[dC]
X_90_Width                 = 11[us]
X_Acq_Time                 = 0.69206016[s]
X_Angle                    = 30[deg]
X_Atn                      = 6.8[dB]
X_Pulse                    = 3.66666667[us]
Irr_Atn_Dec                = 27.862[dB]
Irr_Atn_Dec_Calc           = 27.862[dB]
Irr_Atn_Dec_Default_Calc   = 27.862[dB]
Irr_Atn_Noe                = 27.862[dB]
Irr_Dec_Bandwidth_Hz       = 7.23684211[kHz]
Irr_Dec_Bandwidth_Ppm      = 12.05794078[ppm]
Irr_Dec_Freq               = 600.1723046[MHz]
```

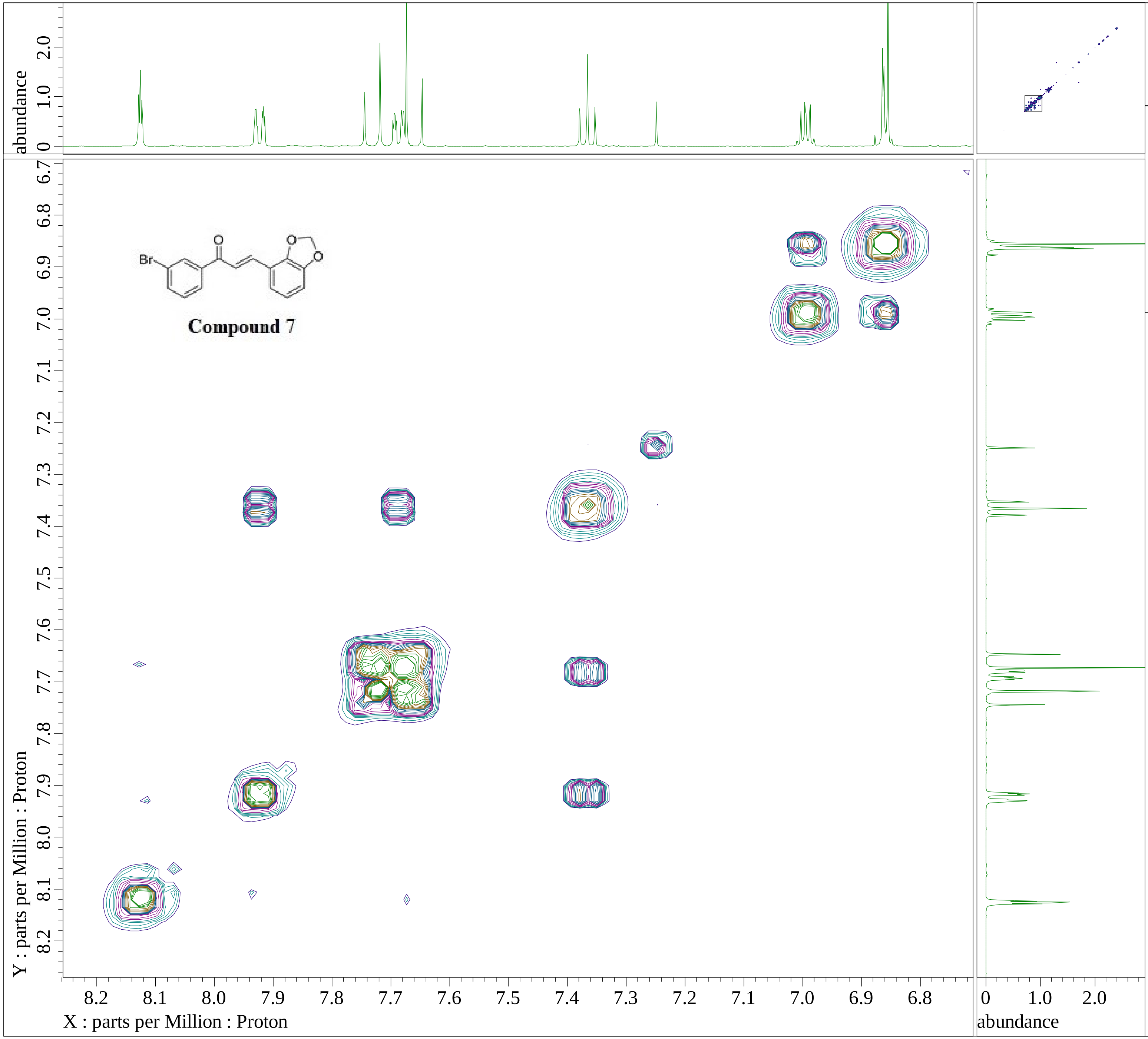


```
---- PROCESSING PARAMETERS ----
sinbell_auto
fft( 1, TRUE, TRUE )
ppm
[transpose]
sinbell_auto
zerofill( 4 )
fft( 1, TRUE, TRUE )
ppm
abs
symmetrize( Cosy, 24 )
[transpose]
```

```
Filename               = CLU-18-106-D33_cosy-1
Author                 = delta
Experiment             = cosy.jxp
Sample_Id              = CLU-18-106-D33
Solvent                = CHLOROFORM-D
Actual_Start_Time      = 13-MAR-2018 09:36:42
Revision_Time          = 13-MAR-2018 09:50:06
Comment                = gradient absolute val
Data_Format            = 2D REAL REAL
Dim_Size               = 1024, 1024
Dim_Title              = Proton Proton
Dim_Units              = [ppm] [ppm]
Dimensions             = X Y
Spectrometer           = JNM-ECZ600R/S1

Field_Strength         = 14.09636928[T] (600[M
X_Acq_Duration         = 0.1134592[s]
X_Domain               = 1H
X_Freq                 = 600.1723046[MHz]
X_Offset               = 5[ppm]
X_Points               = 1280
X_Prescans             = 4
X_Resolution           = 8.81374097[Hz]
X_Sweep                = 11.28158845[kHz]
X_Sweep_Clipped        = 9.02527076[kHz]
Y_Domain               = 1H
Y_Freq                 = 600.1723046[MHz]
Y_Offset               = 5[ppm]
Y_Points               = 256
Y_Prescans             = 0
Y_Resolution           = 35.17242932[Hz]
Y_Sweep                = 9.00414191[kHz]
Irr_Domain             = Proton
Irr_Freq               = 600.1723046[MHz]
Irr_Offset             = 5[ppm]
Tri_Domain             = Proton
Tri_Freq               = 600.1723046[MHz]
Tri_Offset             = 5[ppm]
Blanking               = 2[us]
Clipped                = FALSE
Scans                  = 2
Total_Scans            = 512

Relaxation_Delay       = 1.5[s]
Recvr_Gain             = 56
Temp_Get               = 21.4[dC]
X_90_Width             = 7.29[us]
X_Acq_Time             = 0.1134592[s]
```

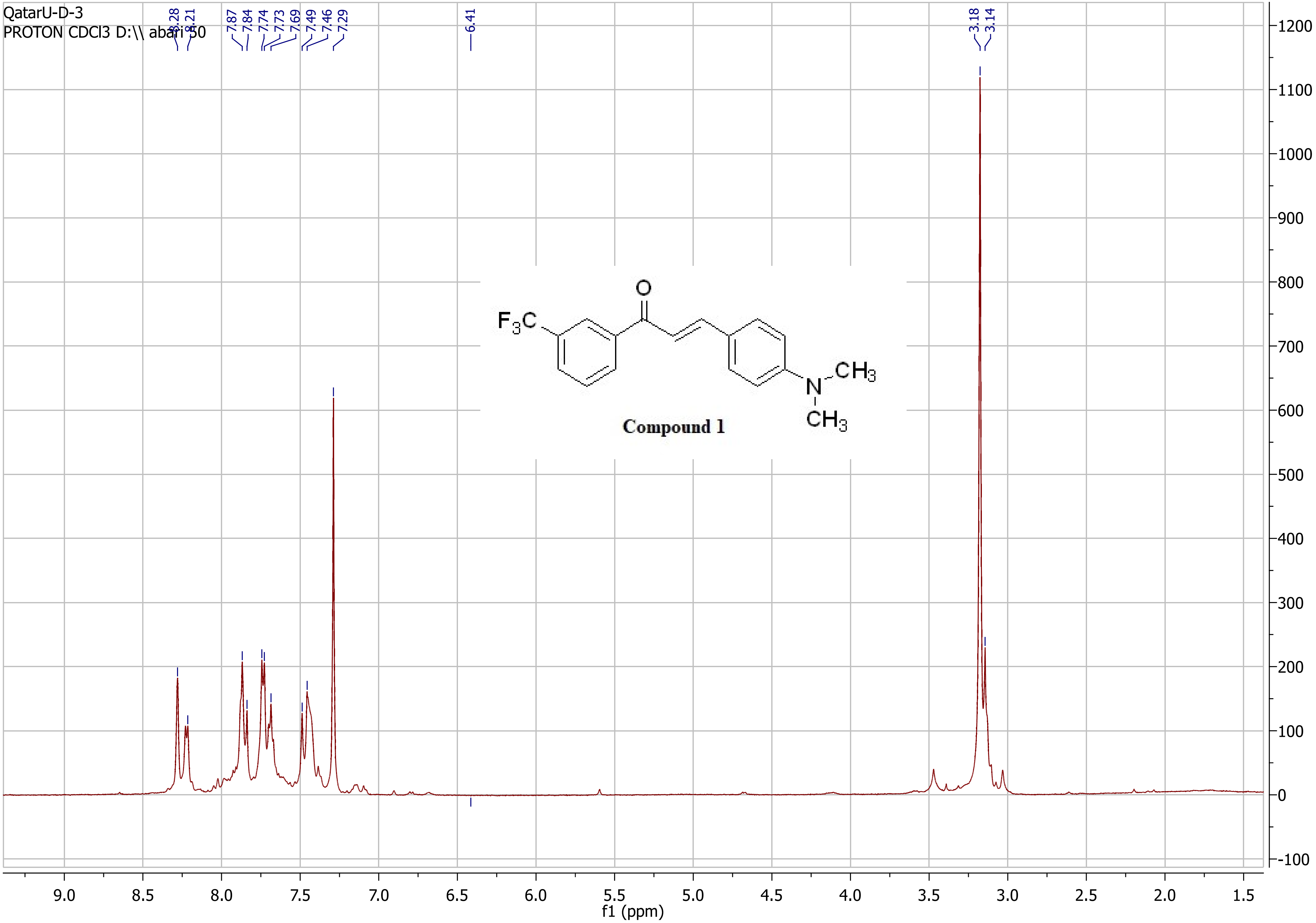

QatarU-D-3
PROTON CDCl3 D:\\ abari 50
8.28
8.21
7.87
7.84
7.74
7.73
7.69
7.49
7.46
7.29
6.41
3.18
3.14
F_3C
O
N
CH_3
CH_3
Compound 1
f1 (ppm)
9.0
8.5
8.0
7.5
7.0
6.5
6.0
5.5
5.0
4.5
4.0
3.5
3.0
2.5
2.0
1.5
1200
1100
1000
900
800
700
600
500
400
300
200
100
0
-100

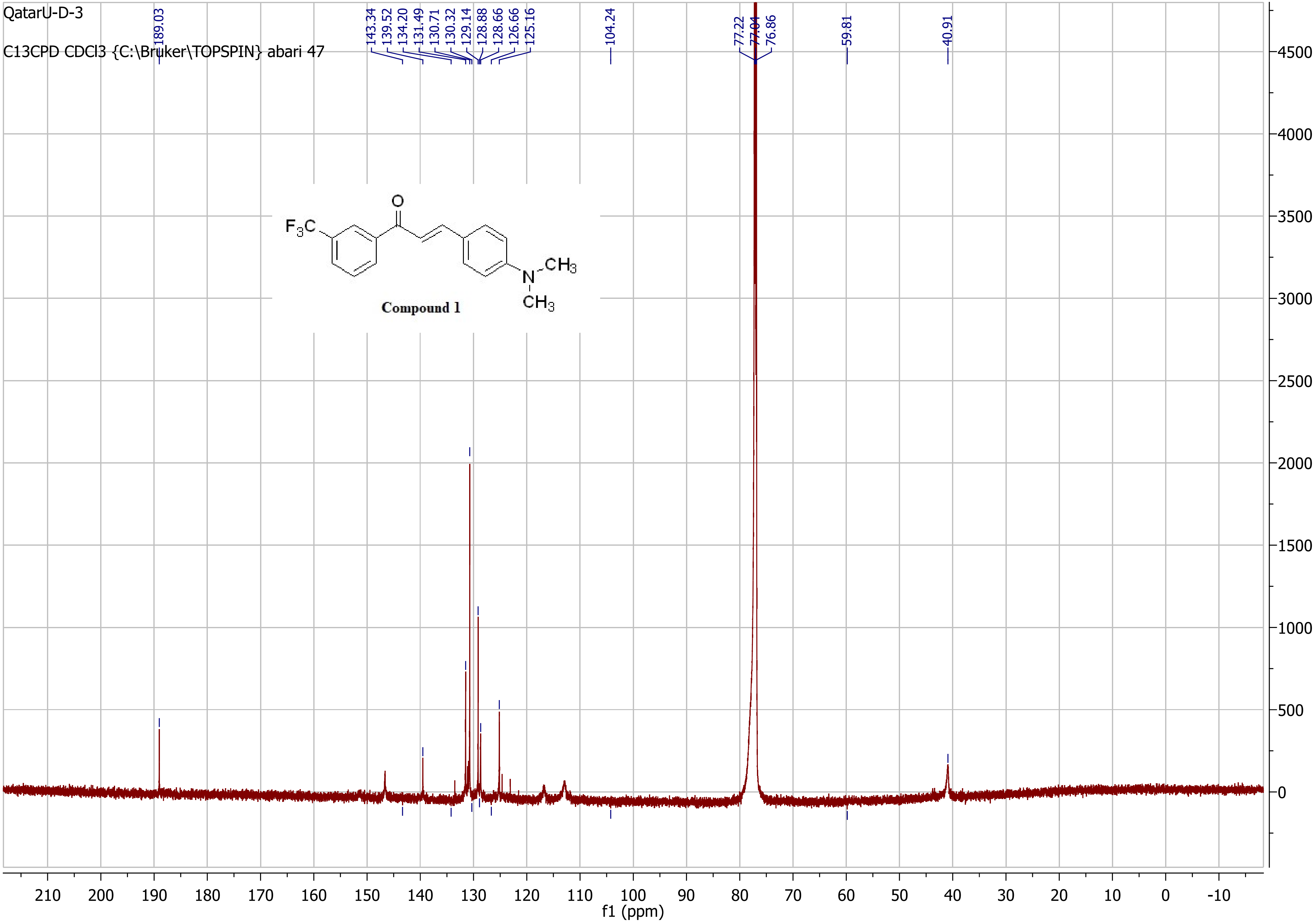

QatarU-D-3
C13CPD CDCl3 {C:\Bruker\TOPSPIN} abari 47
189.03
143.34
139.52
134.20
131.49
130.71
130.32
129.14
128.88
128.66
126.66
125.16
104.24
77.22
77.04
76.86
59.81
40.91
O
F3C
N
CH3
CH3
f1 (ppm)

Compound 1

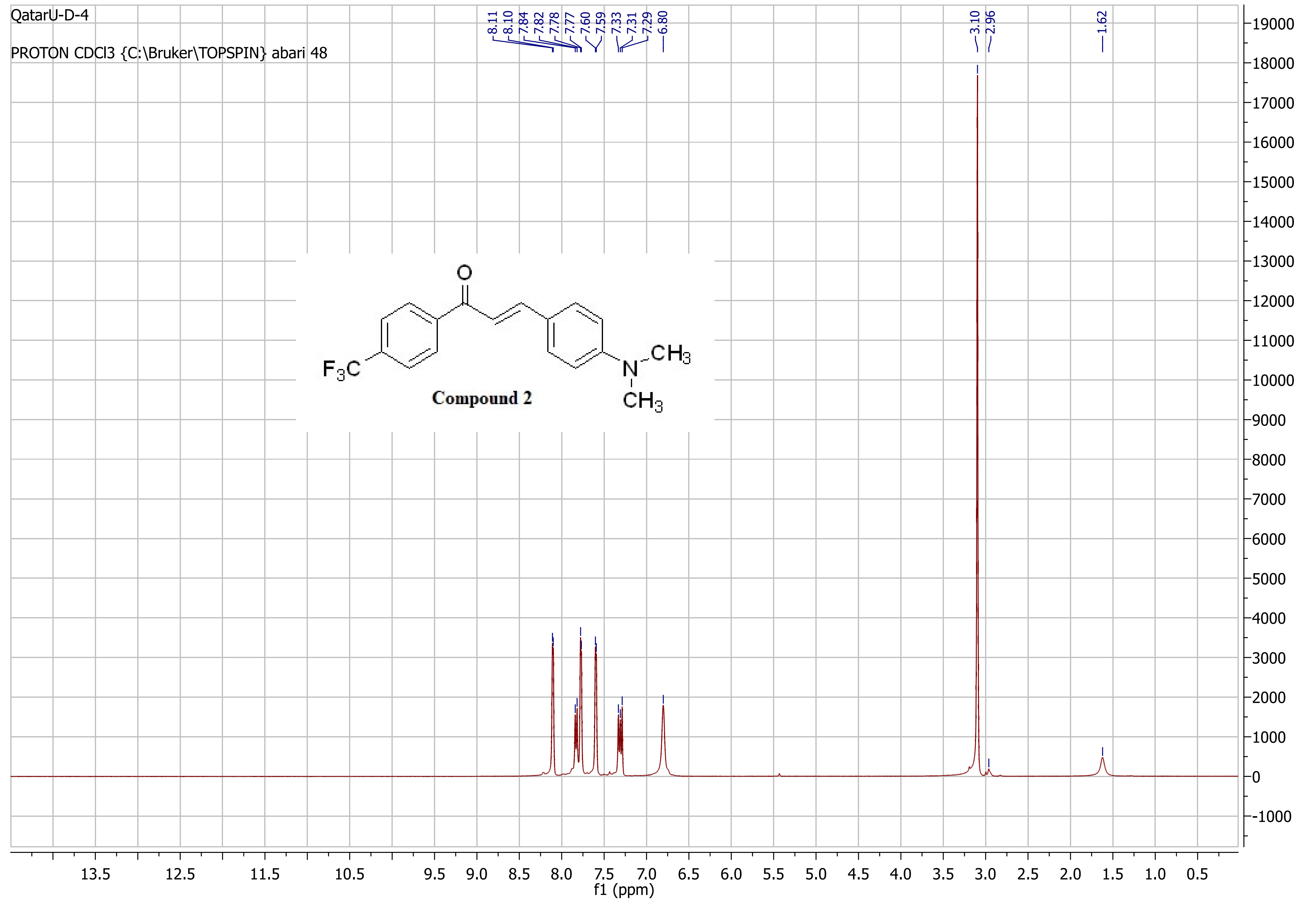

QatarU-D-4
PROTON CDCl3 {C:\Bruker\TOPSPIN} abari 48
8.11
8.10
7.84
7.82
7.78
7.77
7.60
7.59
7.33
7.31
7.29
6.80
3.10
2.96
1.62
O
F3C
N
CH3
CH3
Compound 2
f1 (ppm)

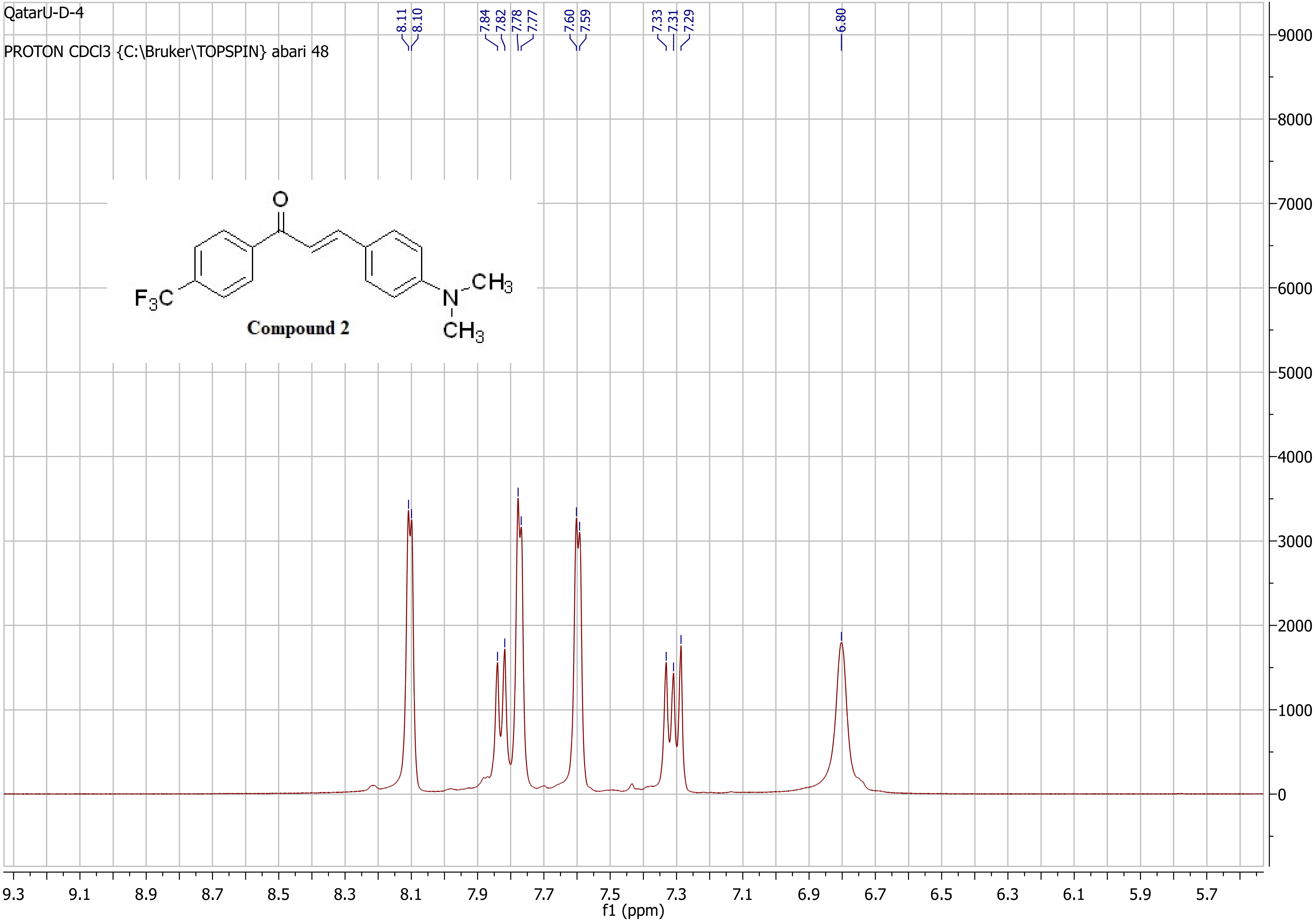
QatarU-D-4
PROTON CDCl3 {C:\Bruker\TOPSPIN} abari 48
8.11
8.10
7.84
7.82
7.78
7.77
7.60
7.59
7.33
7.31
7.29
6.80
O
F3C
N
CH3
CH3
Compound 2
9000
8000
7000
6000
5000
4000
3000
2000
1000
0
9.3
9.1
8.9
8.7
8.5
8.3
8.1
7.9
7.7
7.5
7.3
7.1
6.9
6.7
6.5
6.3
6.1
5.9
5.7
f1 (ppm)

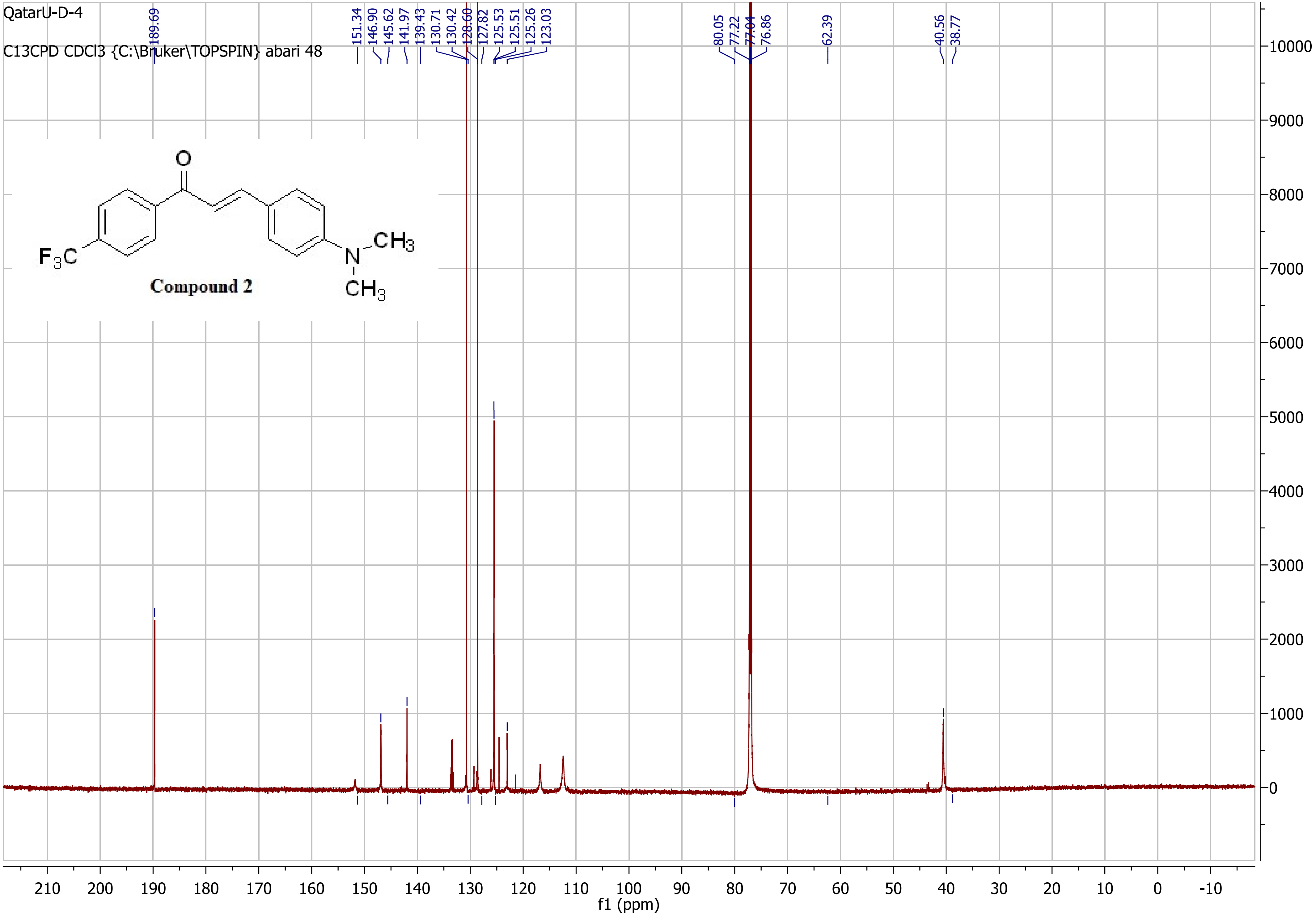
QatarU-D-4
C13CPD CDCl3 {C:\Bruker\TOPSPIN} abari 48
189.69
151.34
146.90
145.62
141.97
139.43
130.71
130.42
128.60
127.82
125.53
125.51
125.26
123.03
80.05
77.22
77.04
76.86
62.39
40.56
38.77
O
F3C
N
CH3
CH3
Compound 2
f1 (ppm)

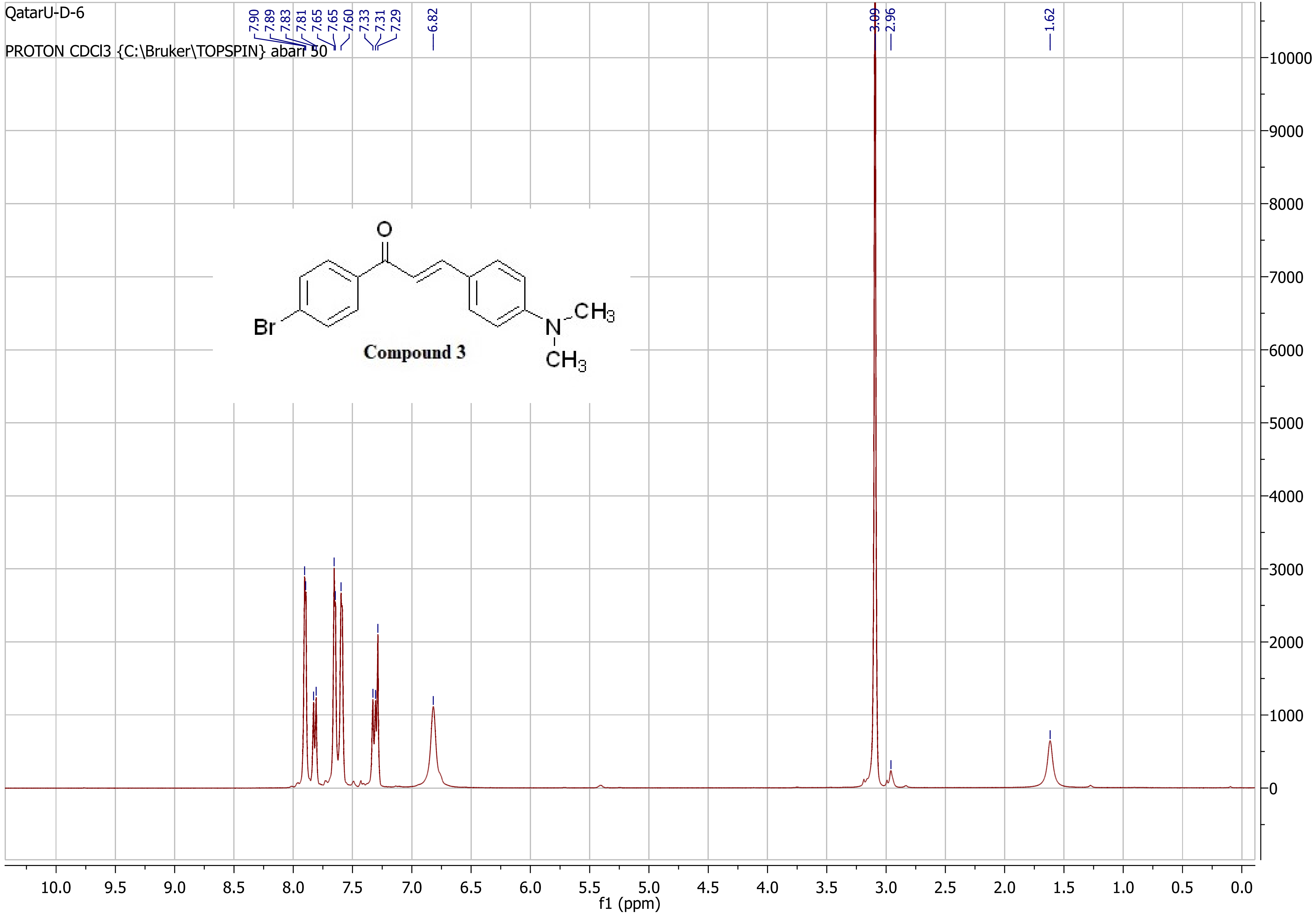
QatarU-D-6
PROTON CDCl3 {C:\Bruker\TOPSPIN} abari 50
7.90
7.89
7.83
7.81
7.65
7.65
7.60
7.33
7.31
7.29
6.82
3.09
2.96
1.62
O
Br
N
CH3
CH3
Compound 3
10000
9000
8000
7000
6000
5000
4000
3000
2000
1000
0
10.0
9.5
9.0
8.5
8.0
7.5
7.0
6.5
6.0
5.5
5.0
4.5
4.0
3.5
3.0
2.5
2.0
1.5
1.0
0.5
0.0
f1 (ppm)

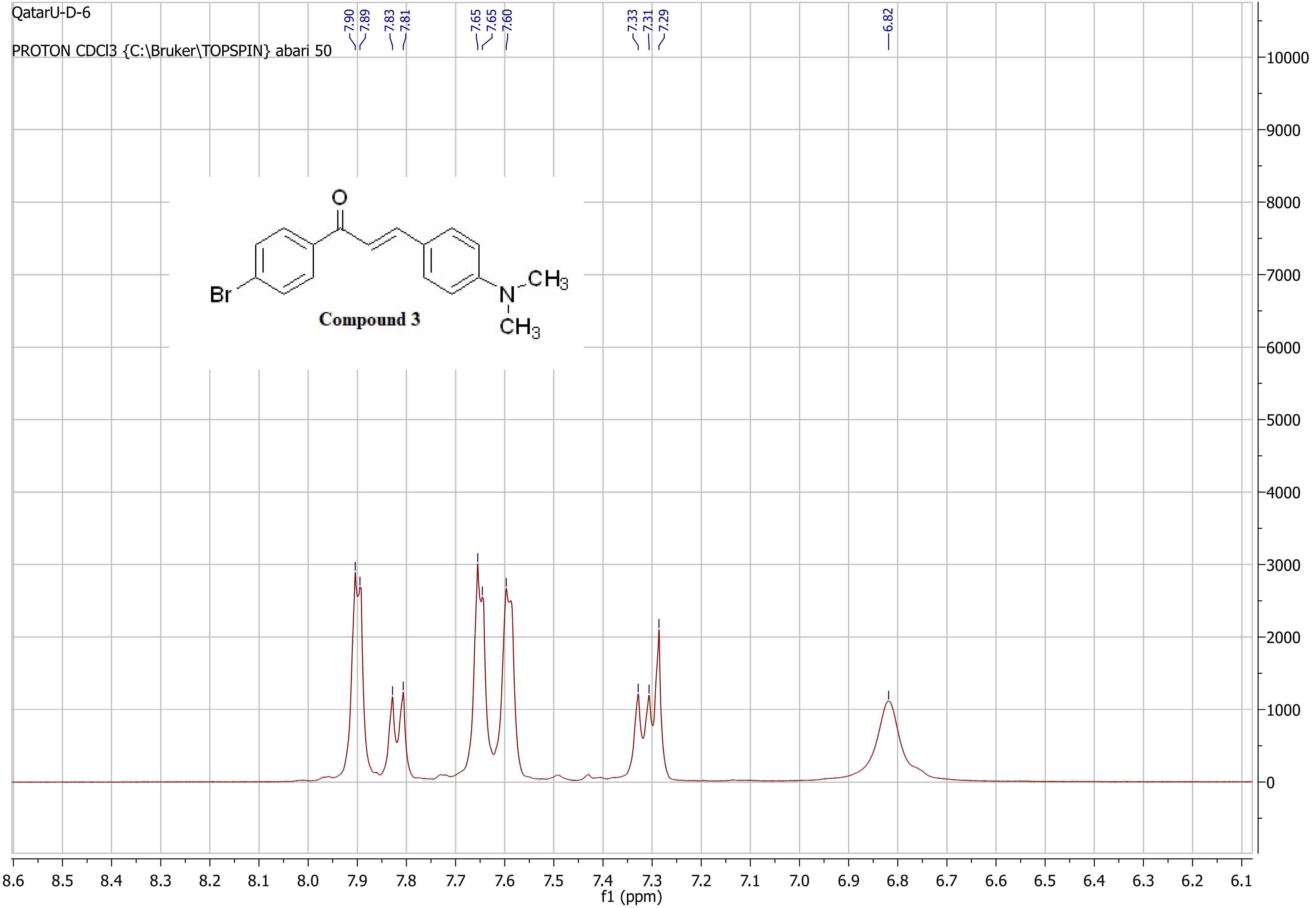
QatarU-D-6
PROTON CDCl3 {C:\Bruker\TOPSPIN} abari 50
7.90
7.89
7.83
7.81
7.65
7.65
7.60
7.33
7.31
7.29
6.82
O
Br
N
CH3
CH3
Compound 3
10000
9000
8000
7000
6000
5000
4000
3000
2000
1000
0
8.6 8.5 8.4 8.3 8.2 8.1 8.0 7.9 7.8 7.7 7.6 7.5 7.4 7.3 7.2 7.1 7.0 6.9 6.8 6.7 6.6 6.5 6.4 6.3 6.2 6.1
f1 (ppm)

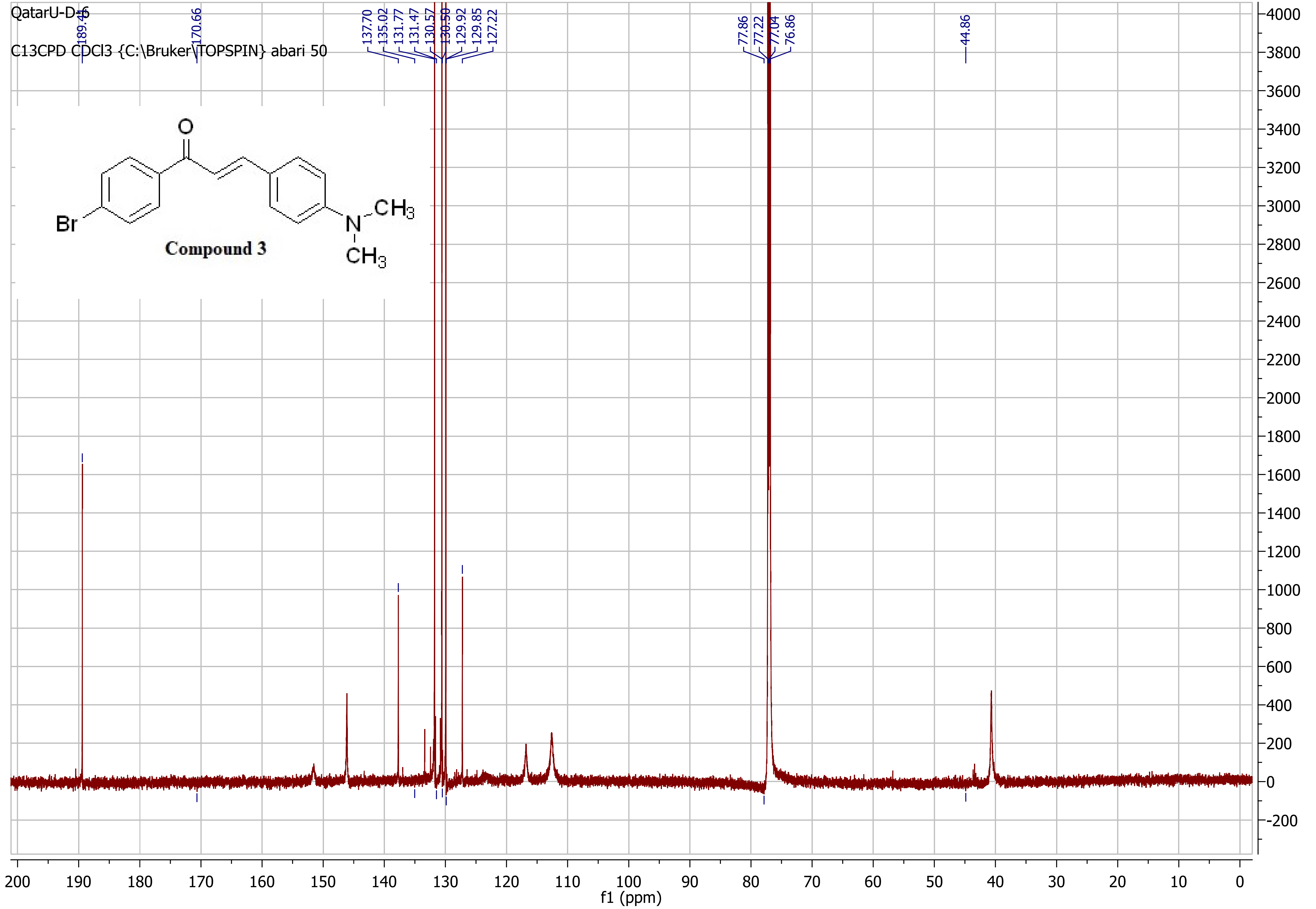
QatarU-D-6
C13CPD CDCl3 {C:\Bruker\TOPSPIN} abari 50
189.41
170.66
137.70
135.02
131.77
131.47
130.57
130.50
129.92
129.85
127.22
77.86
77.22
77.04
76.86
44.86
O
Br
N
CH3
CH3
Compound 3
f1 (ppm)

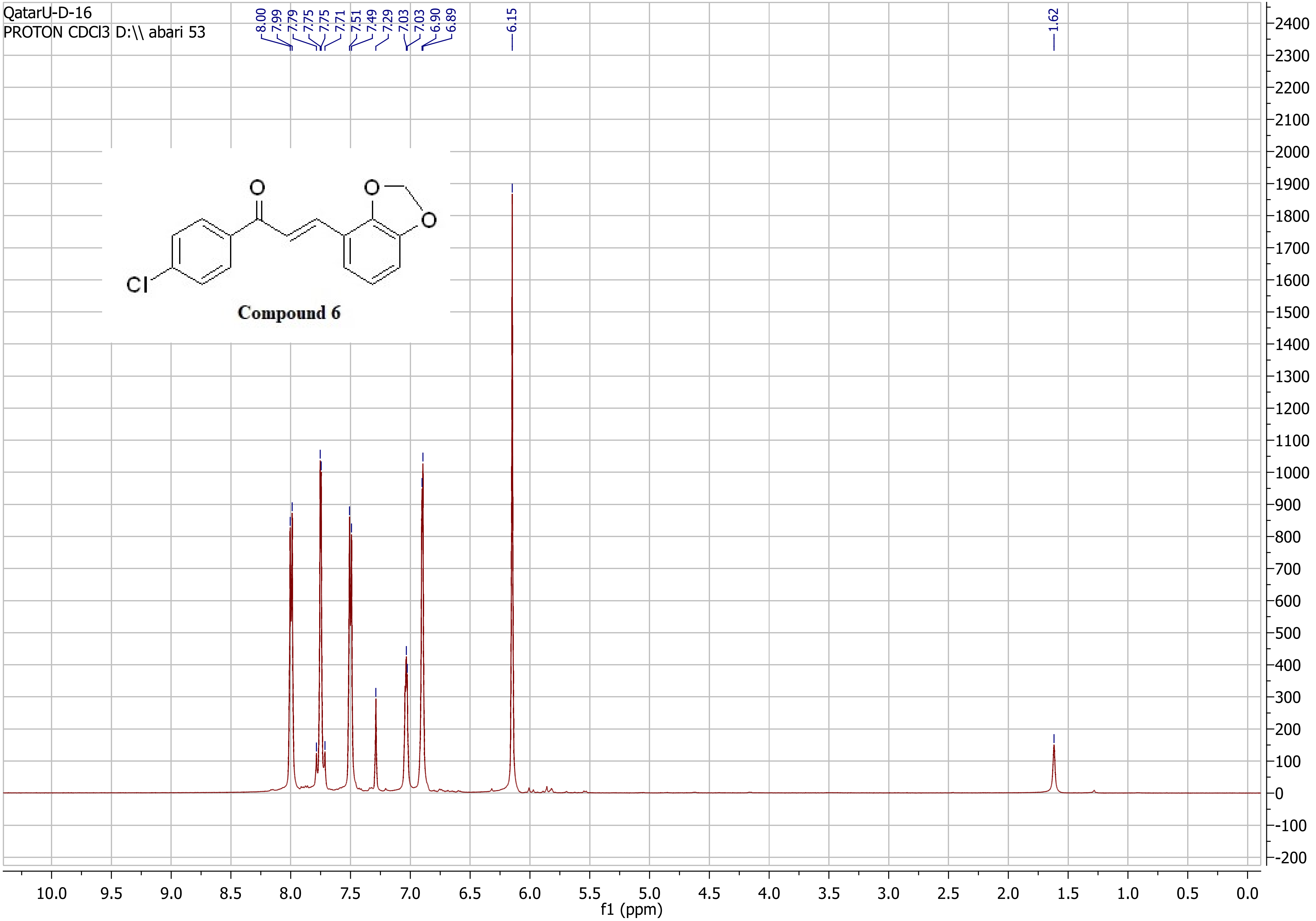
QatarU-D-16
PROTON CDCl3 D:\\ abari 53
8.00
7.99
7.79
7.75
7.75
7.71
7.51
7.49
7.29
7.03
7.03
6.90
6.89
6.15
1.62
O
O
O
Cl
Compound 6
f1 (ppm)

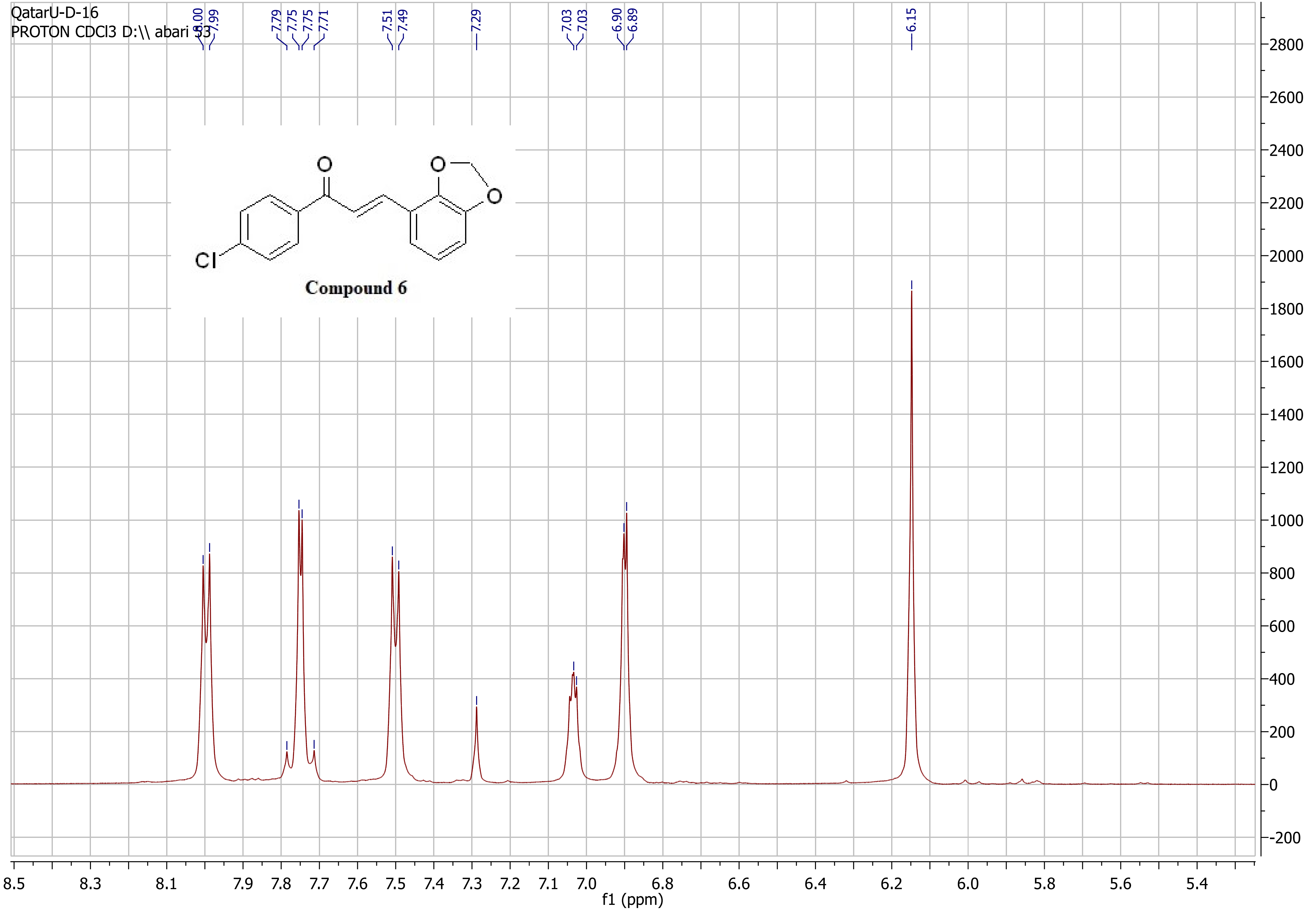
QatarU-D-16
PROTON CDCl3 D:\\ abari 53
8.00
7.99
7.79
7.75
7.75
7.71
7.51
7.49
7.29
7.03
7.03
6.90
6.89
6.15
O
O
O
Cl
Compound 6
f1 (ppm)

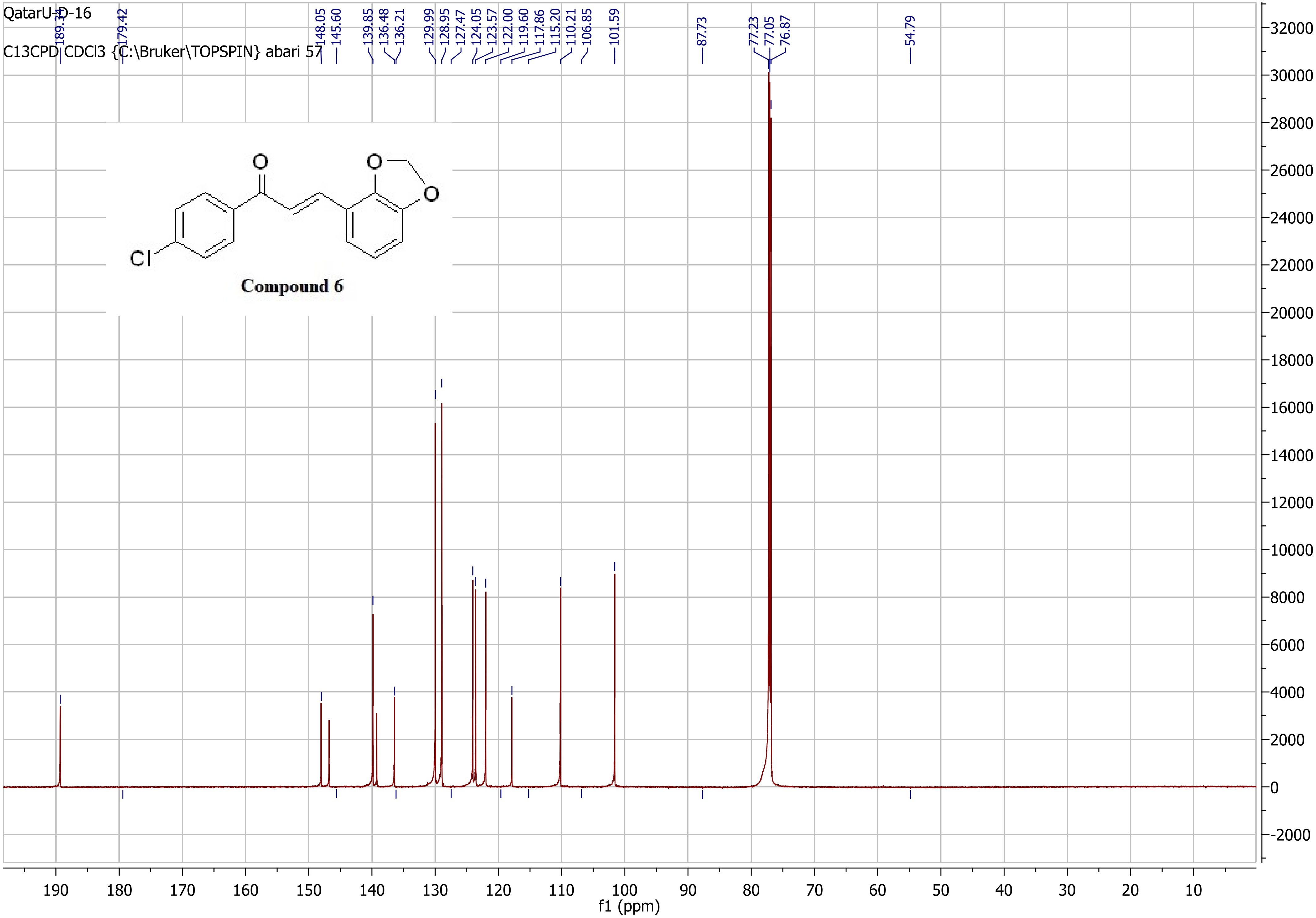

QatarU-D-16
C13CPD CDCl3 {C:\Bruker\TOPSPIN} abari 57
189.3?
179.42
148.05
145.60
139.85
136.48
136.21
129.99
128.95
127.47
124.05
123.57
122.00
119.60
117.86
115.20
110.21
106.85
101.59
87.73
77.23
77.05
76.87
54.79
O
O
O
Cl
Compound 6
f1 (ppm)

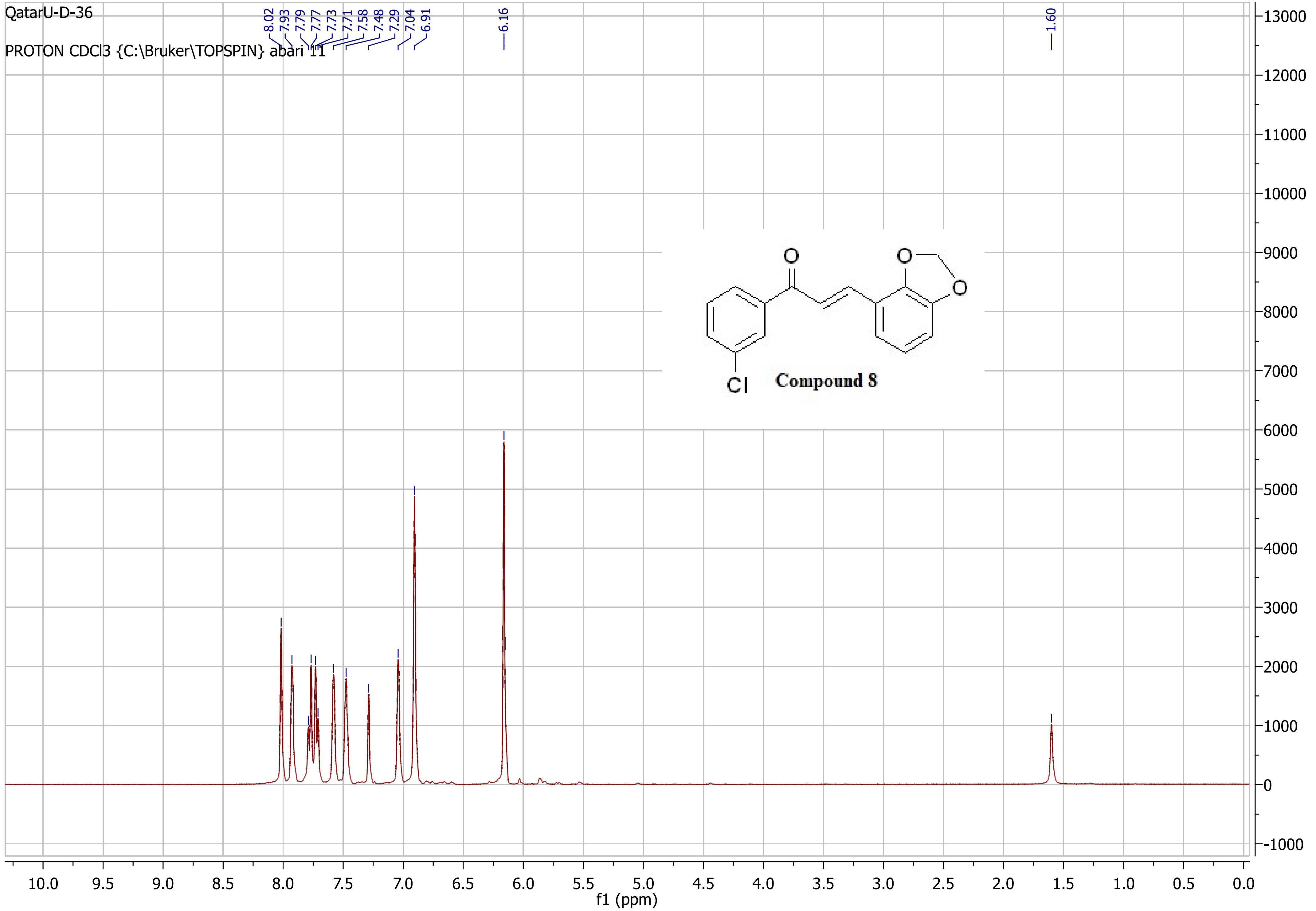
QatarU-D-36
PROTON CDCl3 {C:\Bruker\TOPSPIN} abari 11
8.02
7.93
7.79
7.77
7.73
7.71
7.58
7.48
7.29
7.04
6.91
6.16
1.60
O
O
O
Cl
Compound 8
f1 (ppm)

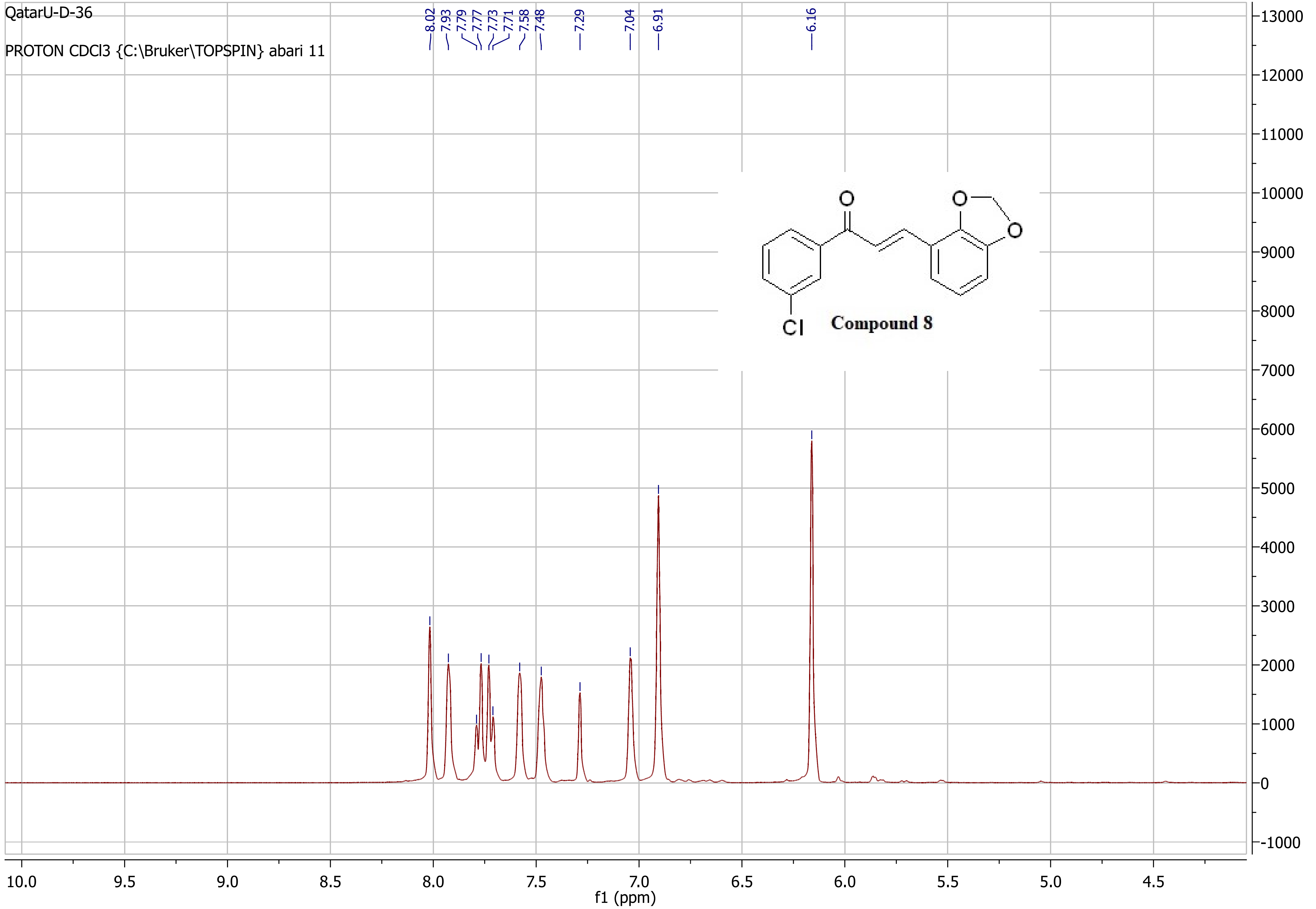
QatarU-D-36
PROTON CDCl3 {C:\Bruker\TOPSPIN} abari 11
8.02
7.93
7.79
7.77
7.73
7.71
7.58
7.48
7.29
7.04
6.91
6.16
O
O
O
Cl
Compound 8
f1 (ppm)
10.0
9.5
9.0
8.5
8.0
7.5
7.0
6.5
6.0
5.5
5.0
4.5
13000
12000
11000
10000
9000
8000
7000
6000
5000
4000
3000
2000
1000
0
-1000

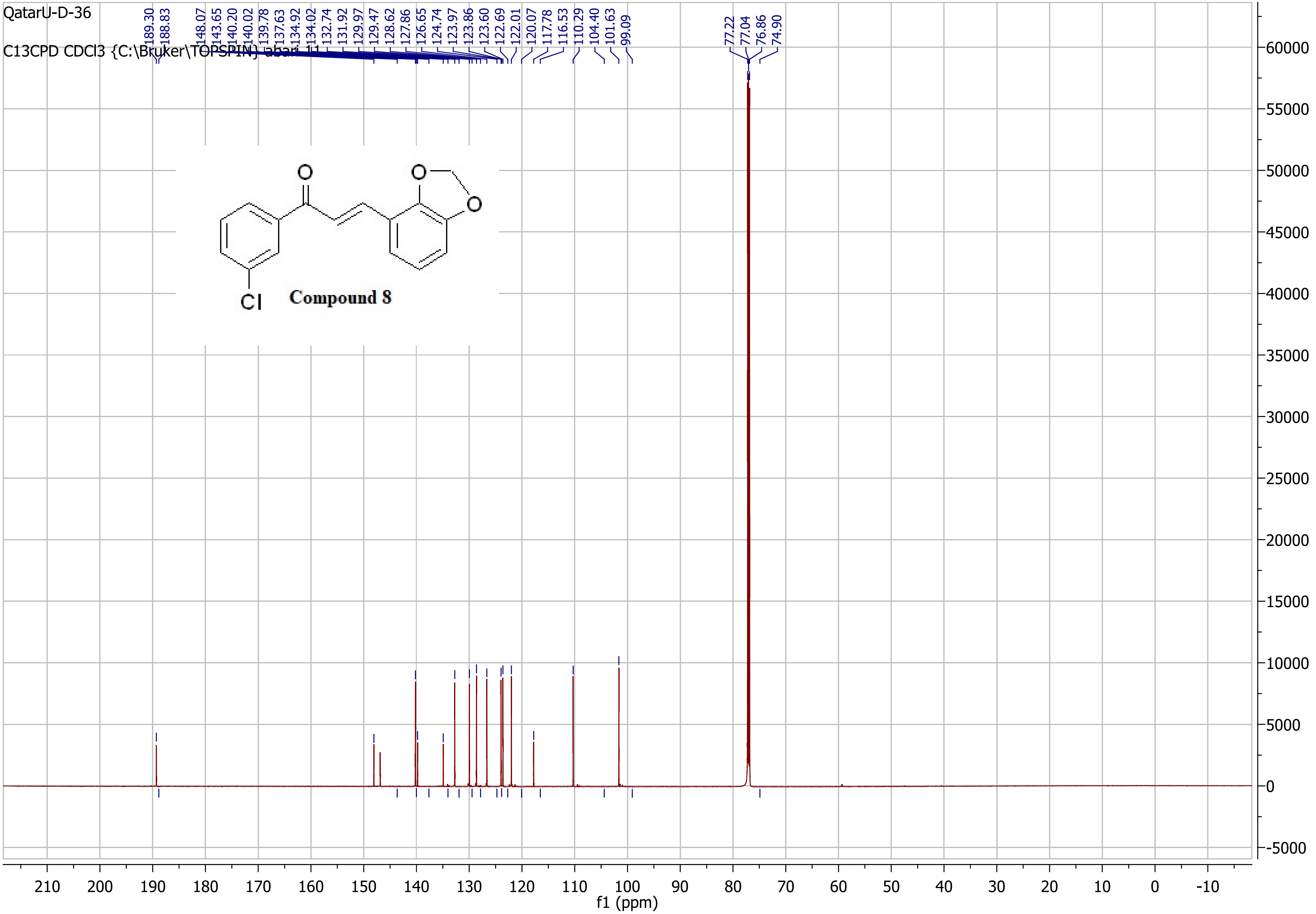
QatarU-D-36
C13CPD CDCl3 {C:\Bruker\TOPSPIN} abari 11
189.30
188.83
148.07
143.65
140.20
140.02
139.78
137.63
134.92
134.02
132.74
131.92
129.97
129.47
128.62
127.86
126.65
124.74
123.97
123.86
123.60
122.69
122.01
120.07
117.78
116.53
110.29
104.40
101.63
99.09
77.22
77.04
76.86
74.90
O
O
O
Cl
Compound 8
f1 (ppm)

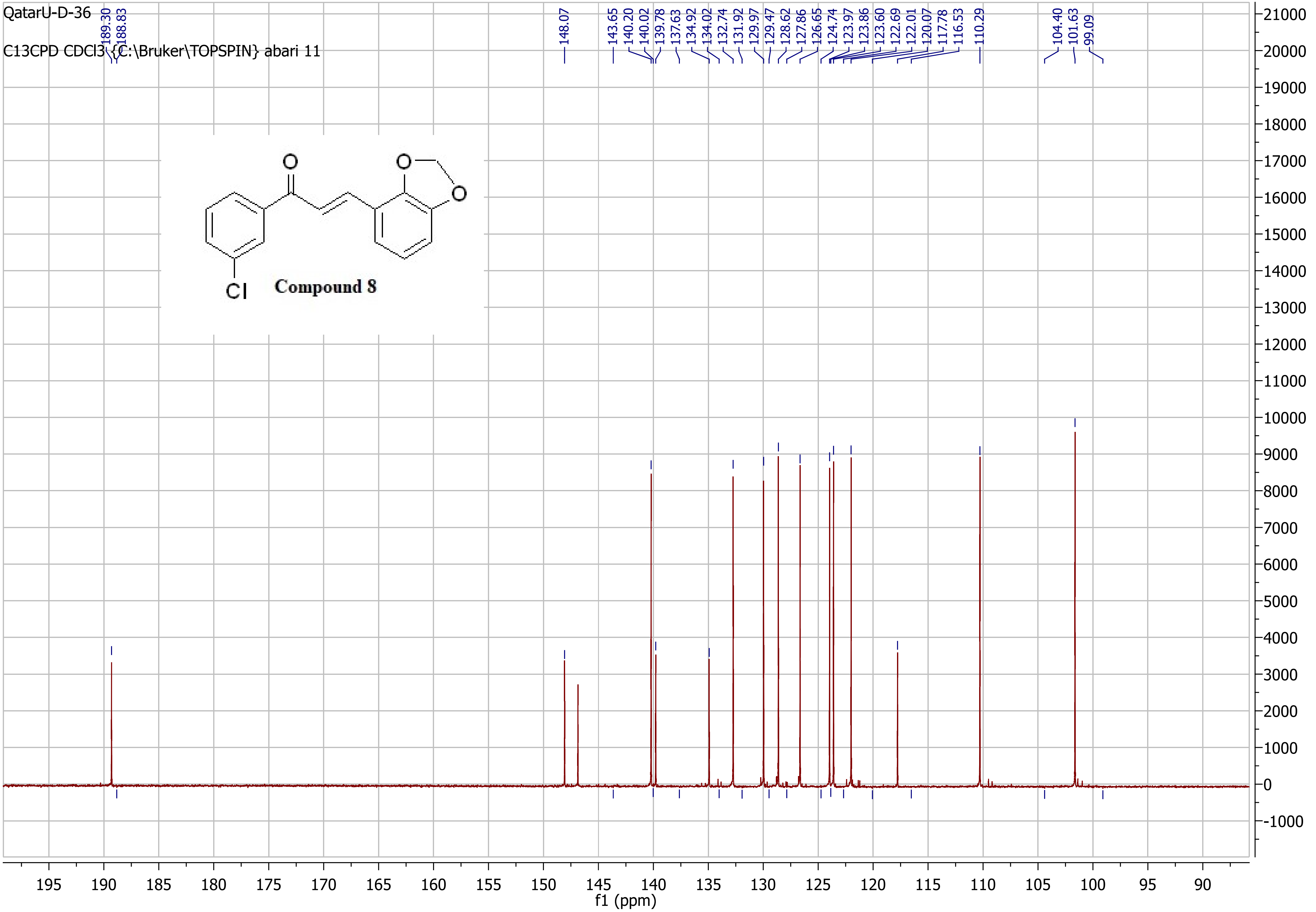

QatarU-D-36
C13CPD CDCl3 {C:\Bruker\TOPSPIN} abari 11
189.30
188.83
148.07
143.65
140.20
140.02
139.78
137.63
134.92
134.02
132.74
131.92
129.97
129.47
128.62
127.86
126.65
124.74
123.97
123.86
123.60
122.69
122.01
120.07
117.78
116.53
110.29
104.40
101.63
99.09
O
O
O
Cl
Compound 8
f1 (ppm)

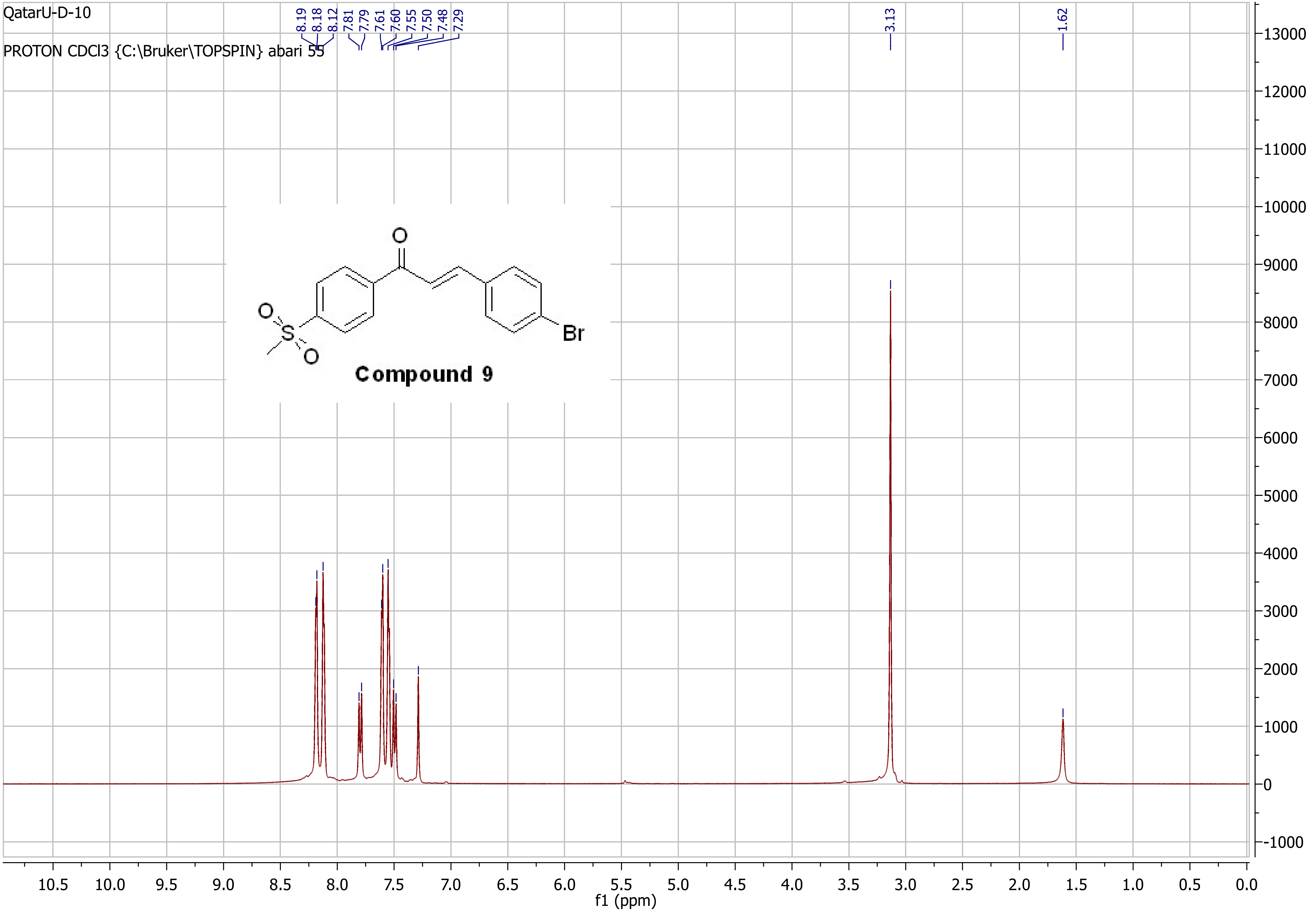
QatarU-D-10
PROTON CDCl3 {C:\Bruker\TOPSPIN} abari 55
8.19 8.18 8.12 7.81 7.79 7.61 7.60 7.55 7.50 7.48 7.29
3.13
1.62
O
S
O
O
Br
Compound 9
f1 (ppm)

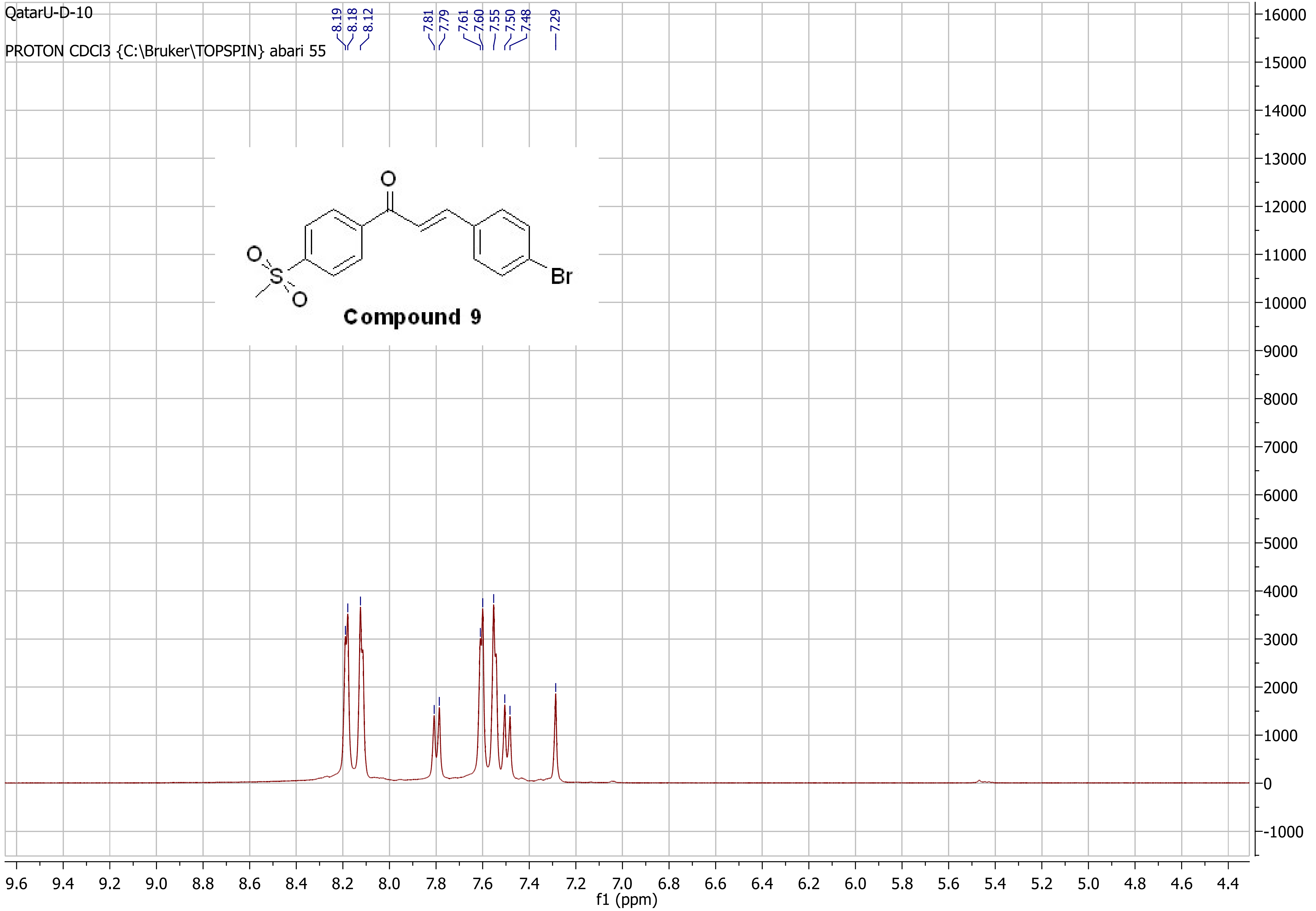

QatarU-D-10
PROTON CDCl3 {C:\Bruker\TOPSPIN} abari 55
8.19
8.18
8.12
7.81
7.79
7.61
7.60
7.55
7.50
7.48
7.29
O
O
S
O
Br
Compound 9
f1 (ppm)

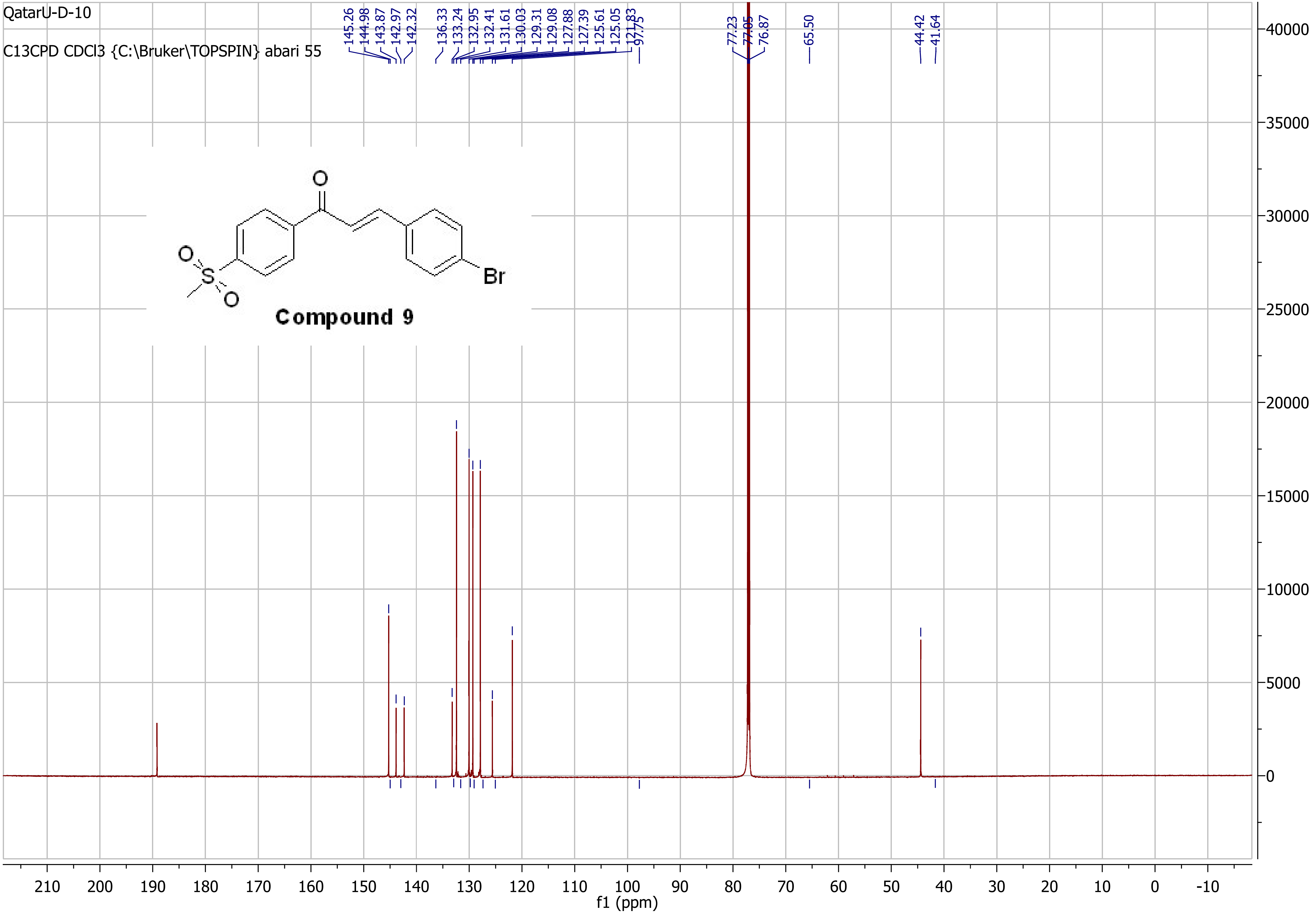
QatarU-D-10
C13CPD CDCl3 {C:\Bruker\TOPSPIN} abari 55
145.26
144.98
143.87
142.97
142.32
136.33
133.24
132.95
132.41
131.61
130.03
129.31
129.08
127.88
127.39
125.61
125.05
121.83
97.75
77.23
77.05
76.87
65.50
44.42
41.64
O
O
S
O
Br
Compound 9
40000
35000
30000
25000
20000
15000
10000
5000
0
210 200 190 180 170 160 150 140 130 120 110 100 90 80 70 60 50 40 30 20 10 0 -10
f1 (ppm)

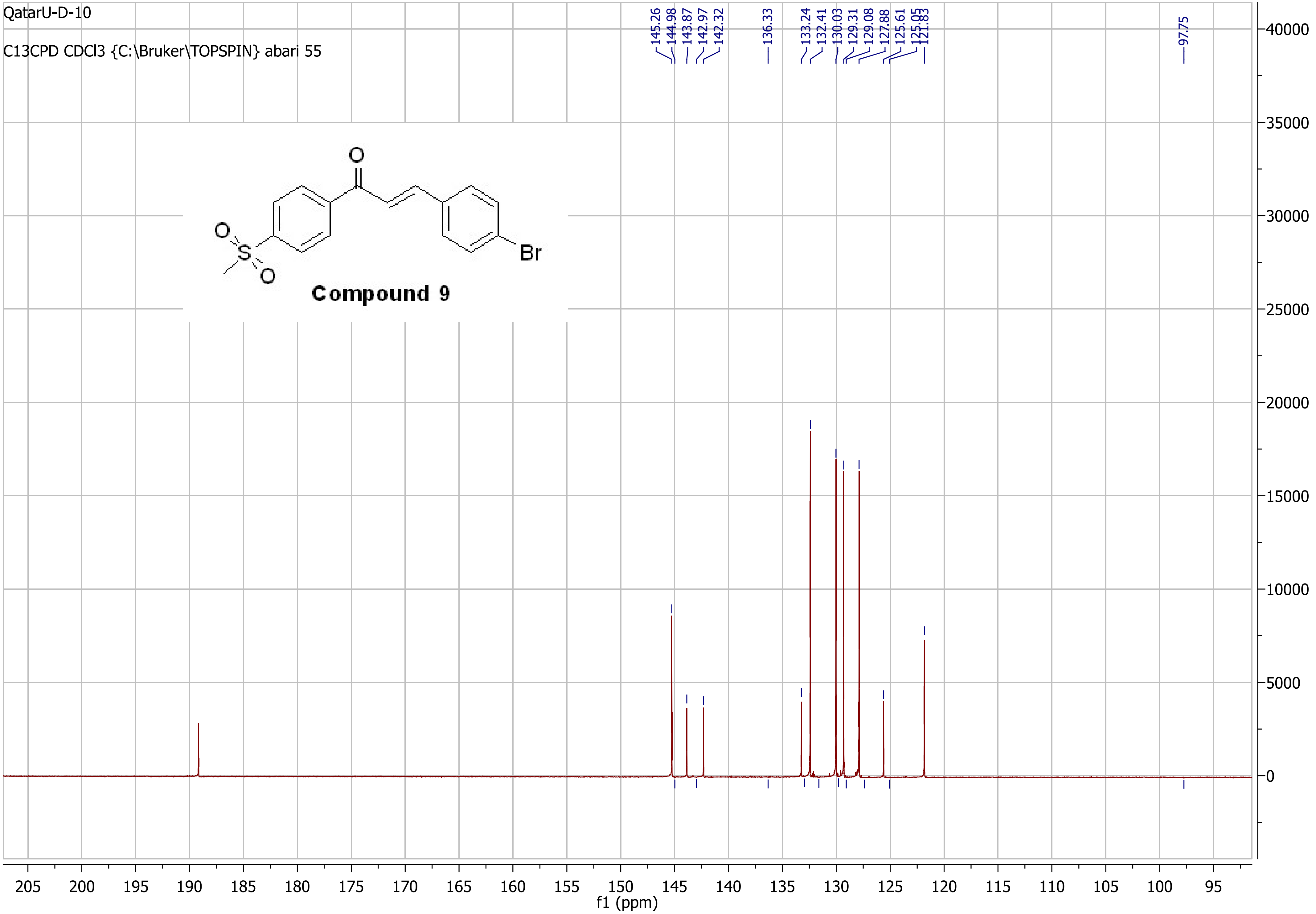
QatarU-D-10
C13CPD CDCl3 {C:\Bruker\TOPSPIN} abari 55
145.26
144.98
143.87
142.97
142.32
136.33
133.24
132.41
130.03
129.31
129.08
127.88
125.61
121.83
97.75
O
O
S
O
Br
Compound 9
40000
35000
30000
25000
20000
15000
10000
5000
0
205
200
195
190
185
180
175
170
165
160
155
150
145
140
135
130
125
120
115
110
105
100
95
f1 (ppm)

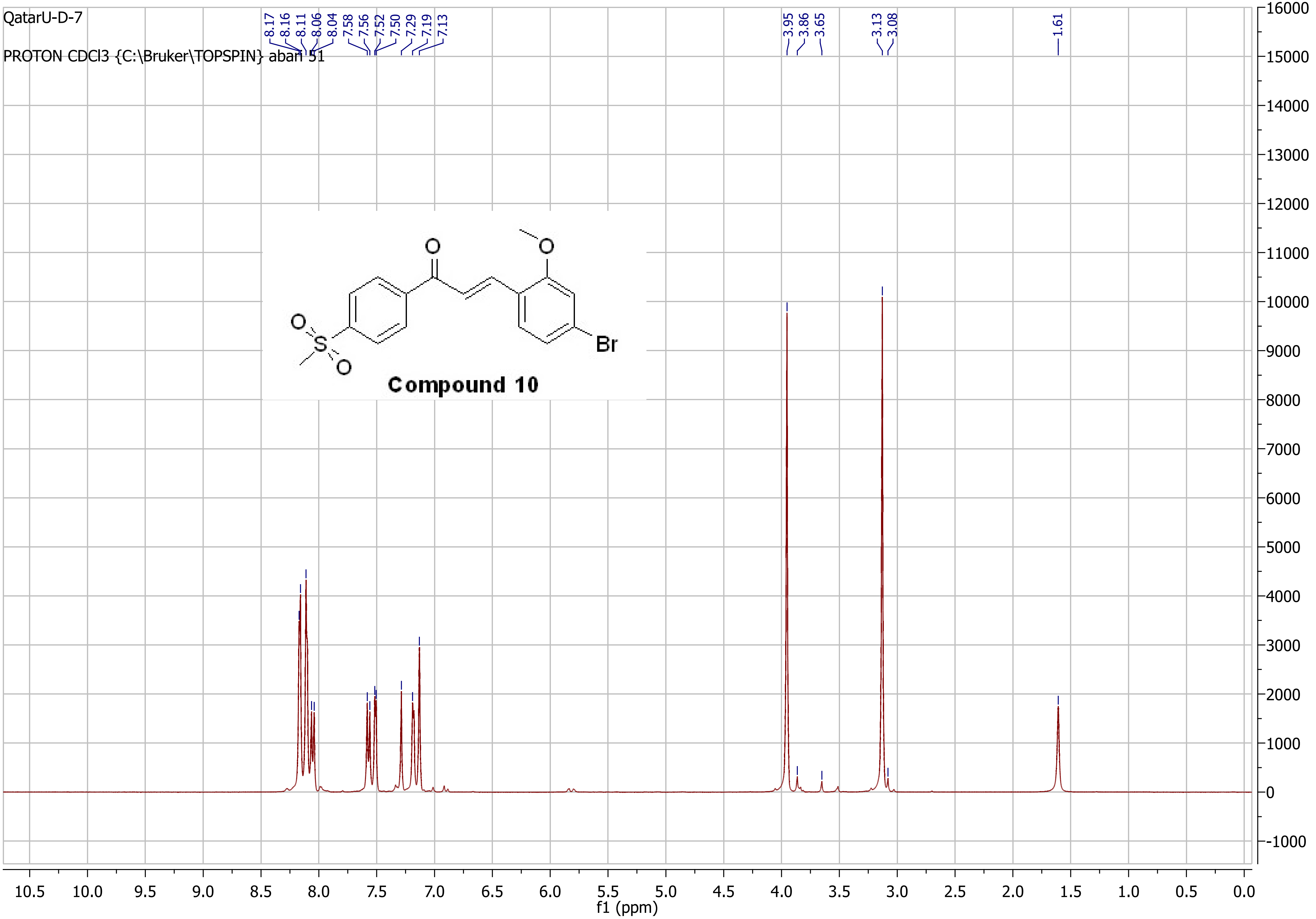
QatarU-D-7
PROTON CDCl3 {C:\Bruker\TOPSPIN} abari 51
8.17 8.16 8.11 8.06 8.04 7.58 7.56 7.52 7.50 7.29 7.19 7.13
3.95 3.86 3.65
3.13 3.08
1.61
O
O
O
S
O
Br
Compound 10
f1 (ppm)

QatarU-D-7

PROTON CDCl3 {C:\Bruker\TOPSPIN} abari 51

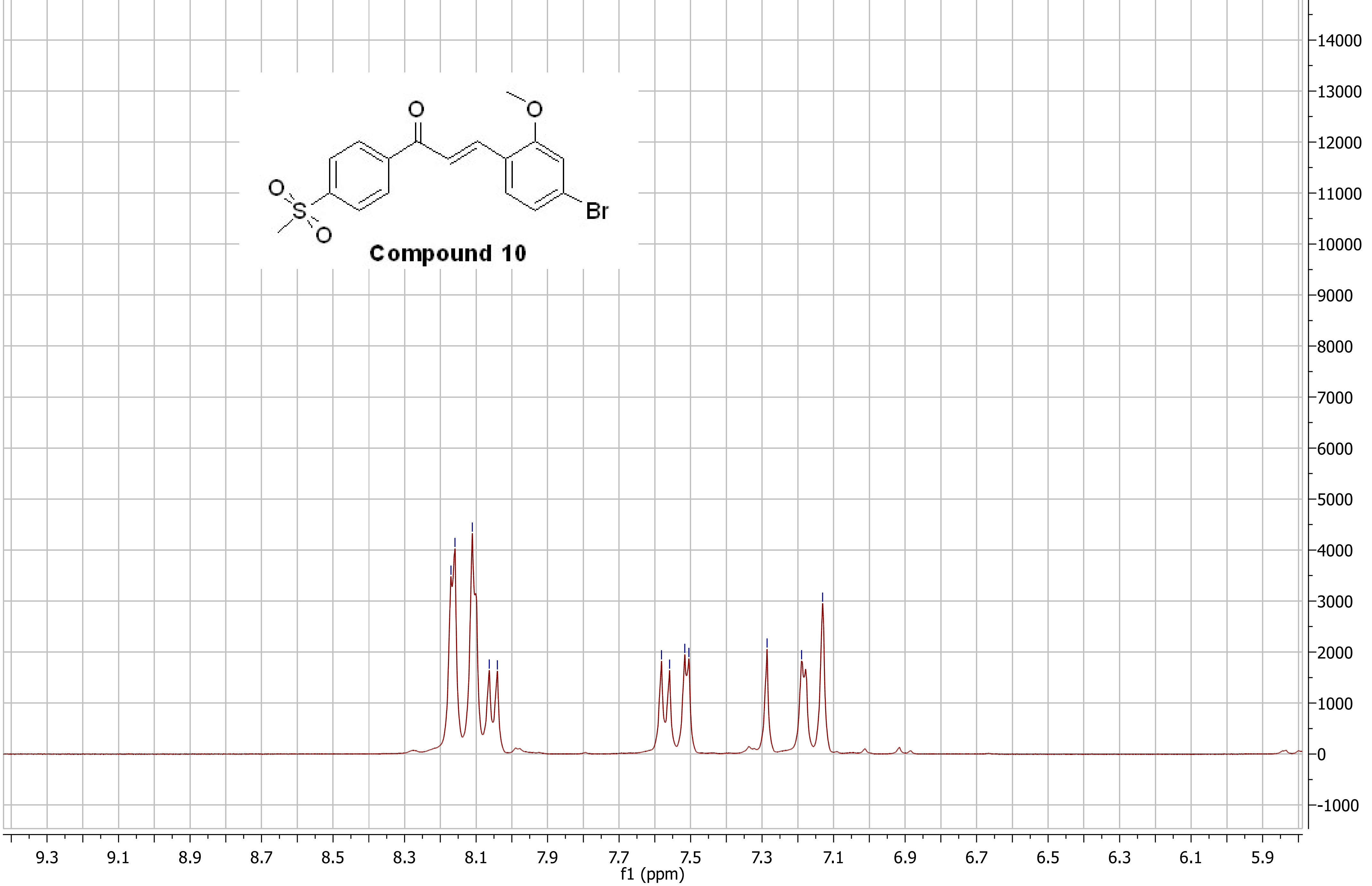

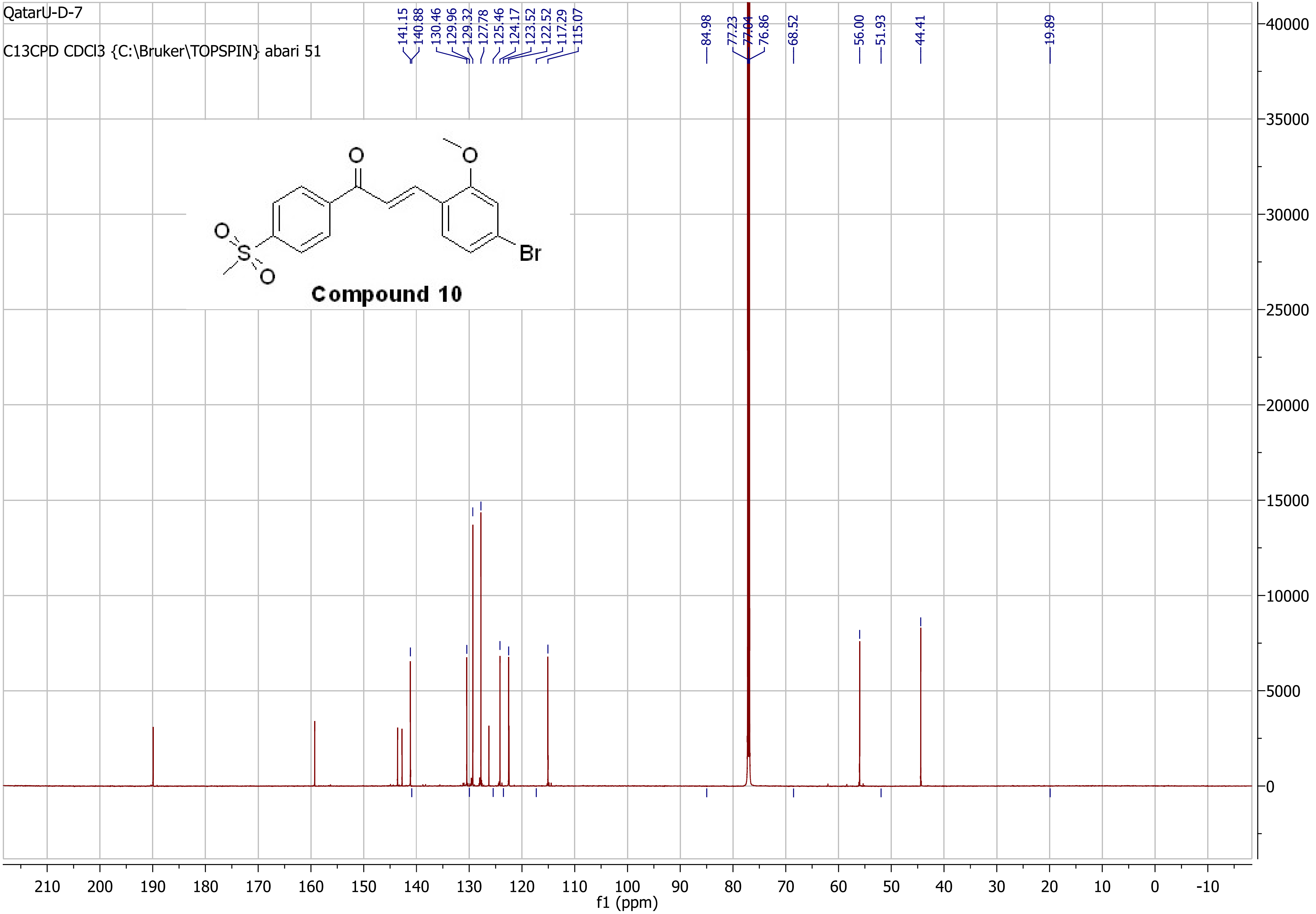
QatarU-D-7
C13CPD CDCl3 {C:\Bruker\TOPSPIN} abari 51
141.15
140.88
130.46
129.96
129.32
127.78
125.46
124.17
123.52
122.52
117.29
115.07
84.98
77.23
77.04
76.86
68.52
56.00
51.93
44.41
19.89
O
O
O
S
O
Br
Compound 10
40000
35000
30000
25000
20000
15000
10000
5000
0
210
200
190
180
170
160
150
140
130
120
110
100
90
80
70
60
50
40
30
20
10
0
-10
f1 (ppm)

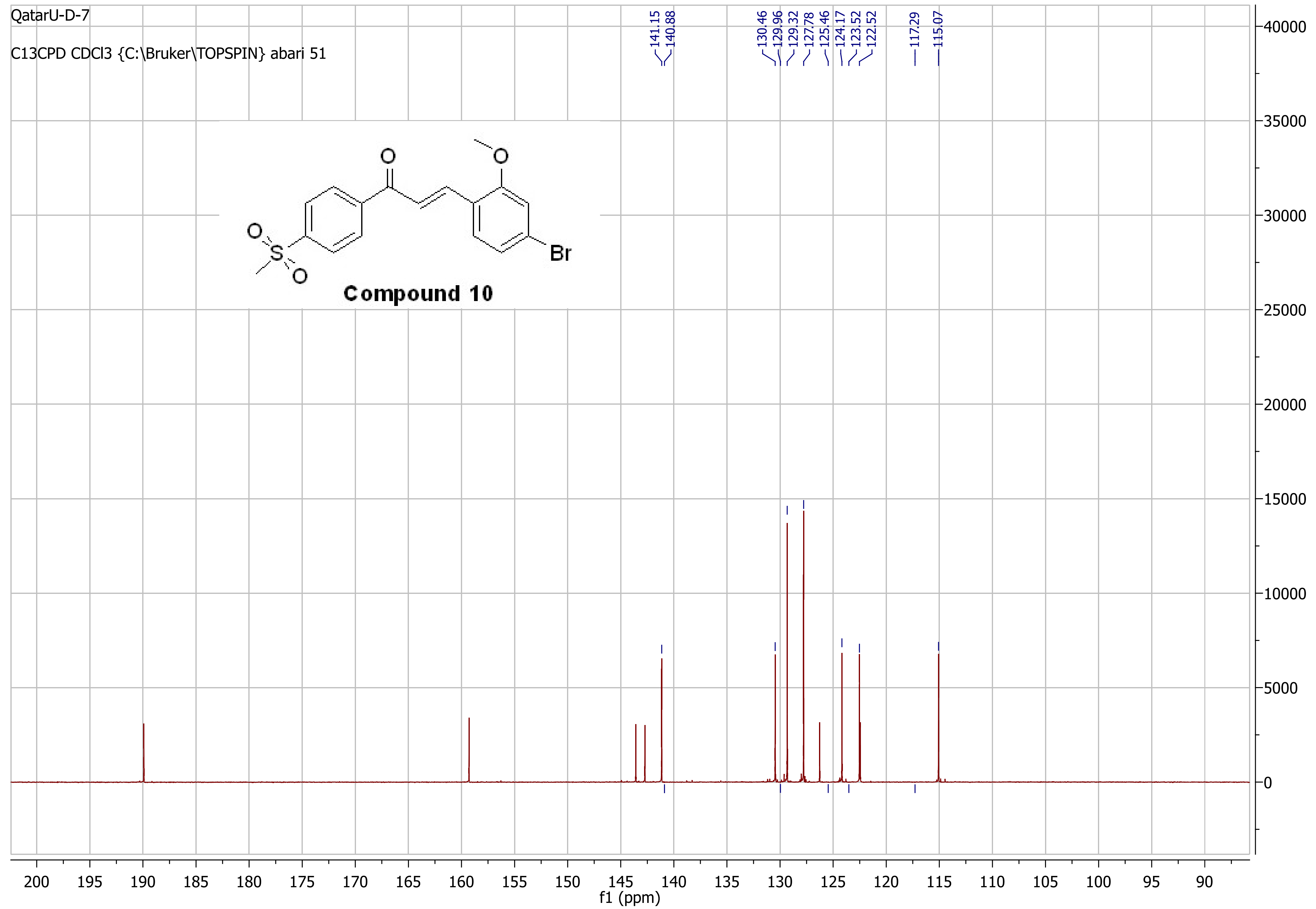
QatarU-D-7
C13CPD CDCl3 {C:\Bruker\TOPSPIN} abari 51
141.15
140.88
130.46
129.96
129.32
127.78
125.46
124.17
123.52
122.52
117.29
115.07
Compound 10
f1 (ppm)

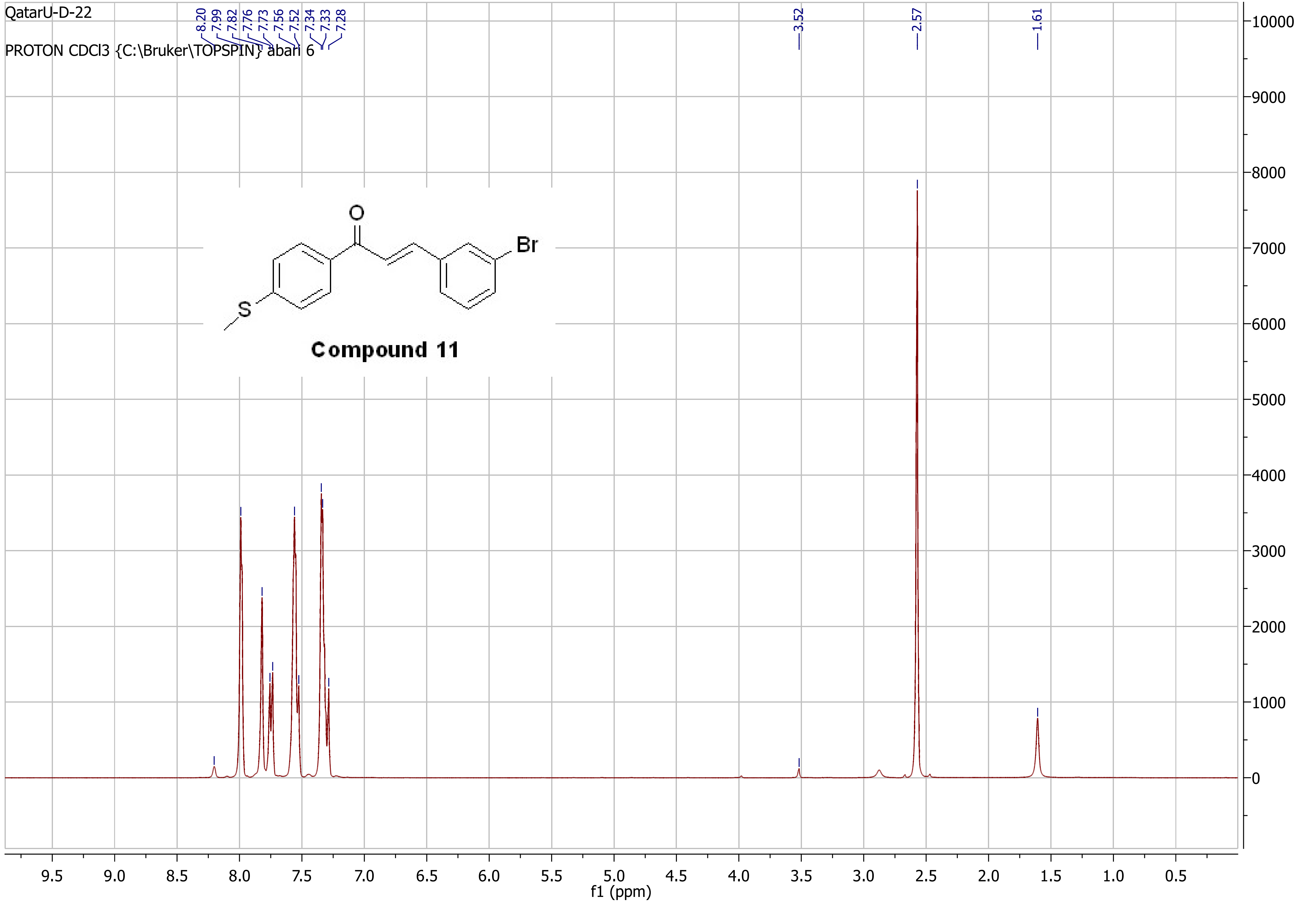
QatarU-D-22
PROTON CDCl3 {C:\Bruker\TOPSPIN} abari 6
8.20
7.99
7.82
7.76
7.73
7.56
7.52
7.34
7.33
7.28
3.52
2.57
1.61
O
Br
S
Compound 11
10000
9000
8000
7000
6000
5000
4000
3000
2000
1000
0
9.5
9.0
8.5
8.0
7.5
7.0
6.5
6.0
5.5
5.0
4.5
4.0
3.5
3.0
2.5
2.0
1.5
1.0
0.5
f1 (ppm)

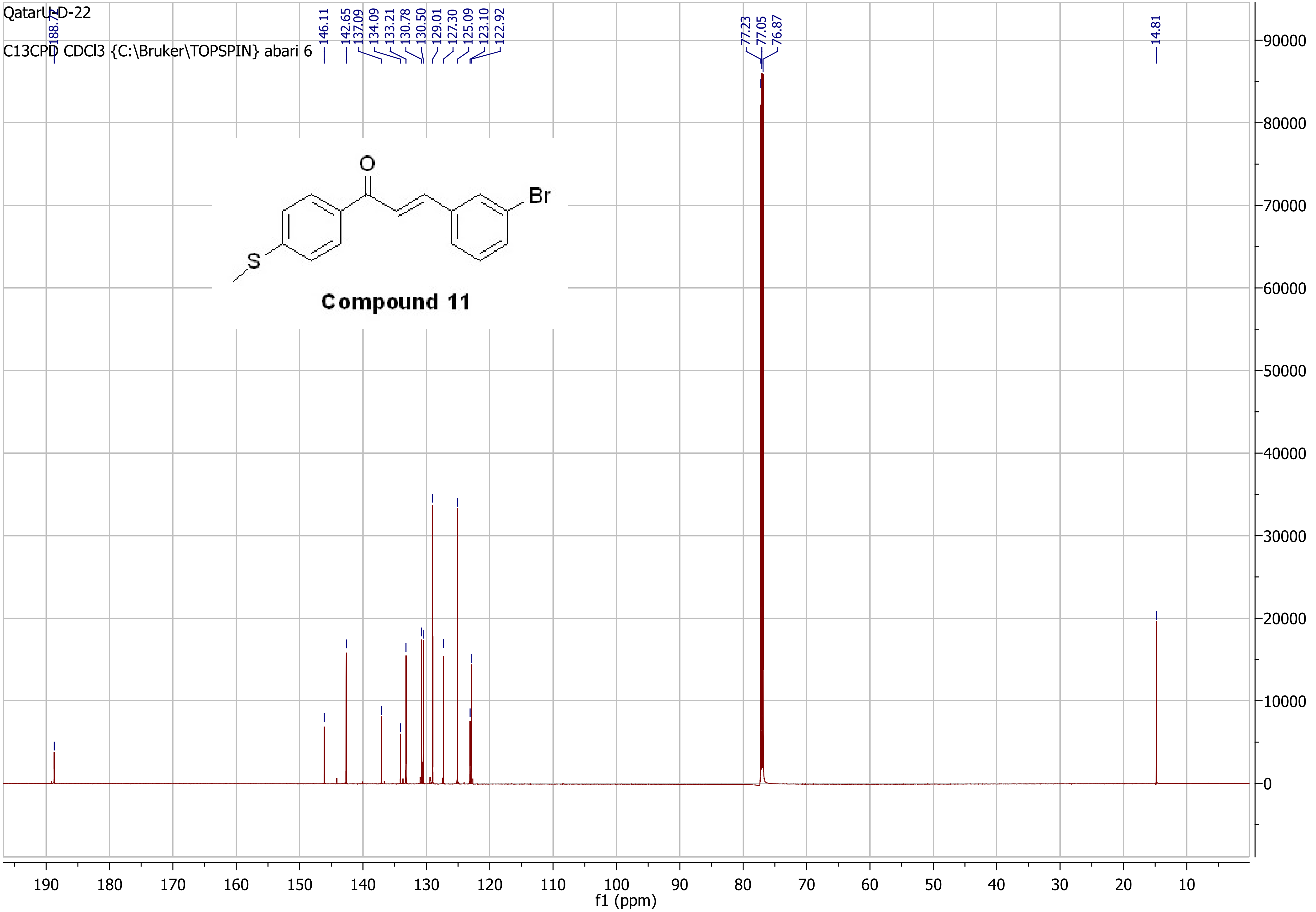
QatarU-D-22
C13CPD CDCl3 {C:\Bruker\TOPSPIN} abari 6
188.72
146.11
142.65
137.09
134.09
133.21
130.78
130.50
129.01
127.30
125.09
123.10
122.92
77.23
77.05
76.87
14.81
O
Br
S
Compound 11
f1 (ppm)

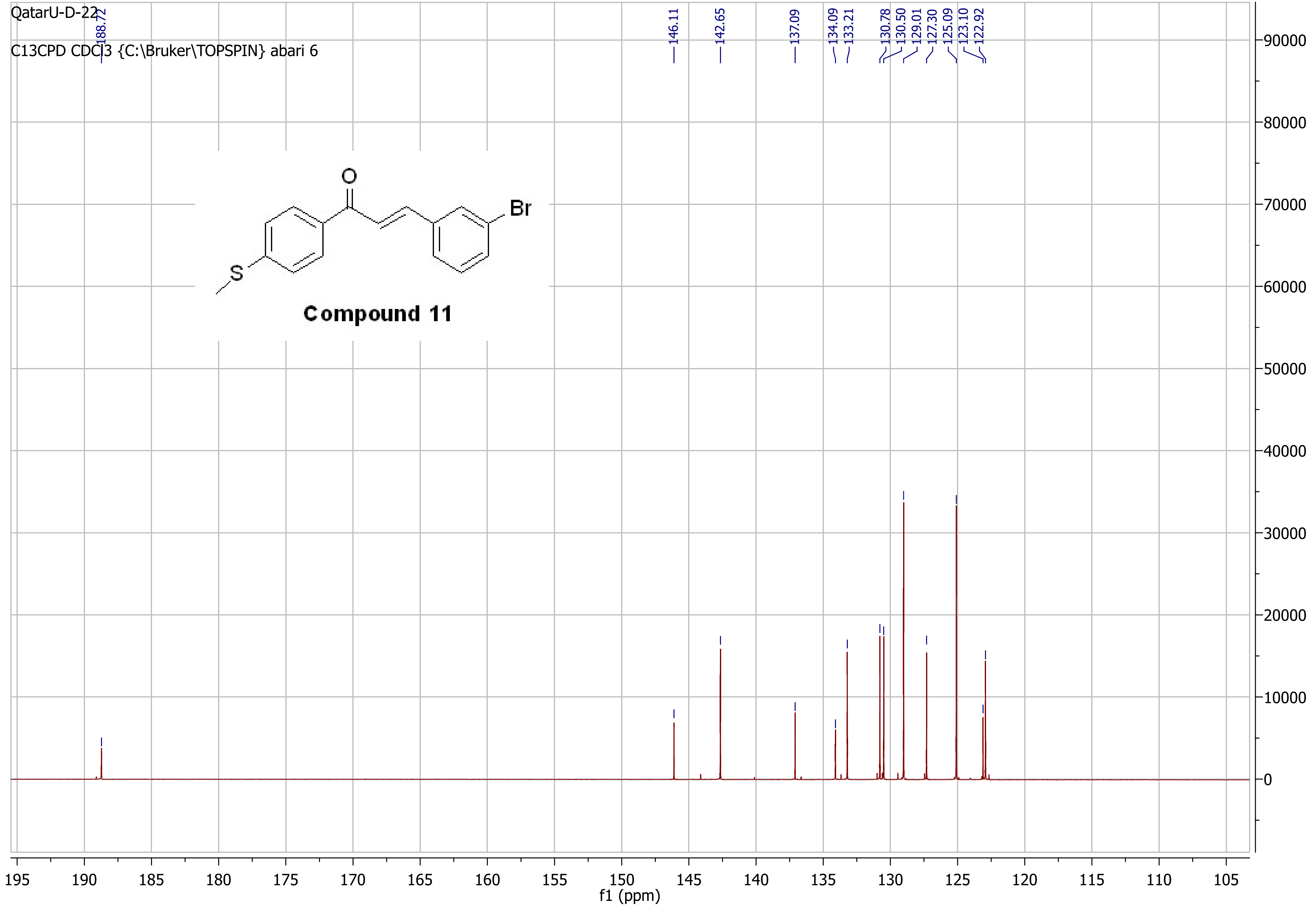
QatarU-D-22
C13CPD CDCl3 {C:\Bruker\TOPSPIN} abari 6
188.72
146.11
142.65
137.09
134.09
133.21
130.78
130.50
129.01
127.30
125.09
123.10
122.92
O
S
Br
Compound 11
f1 (ppm)

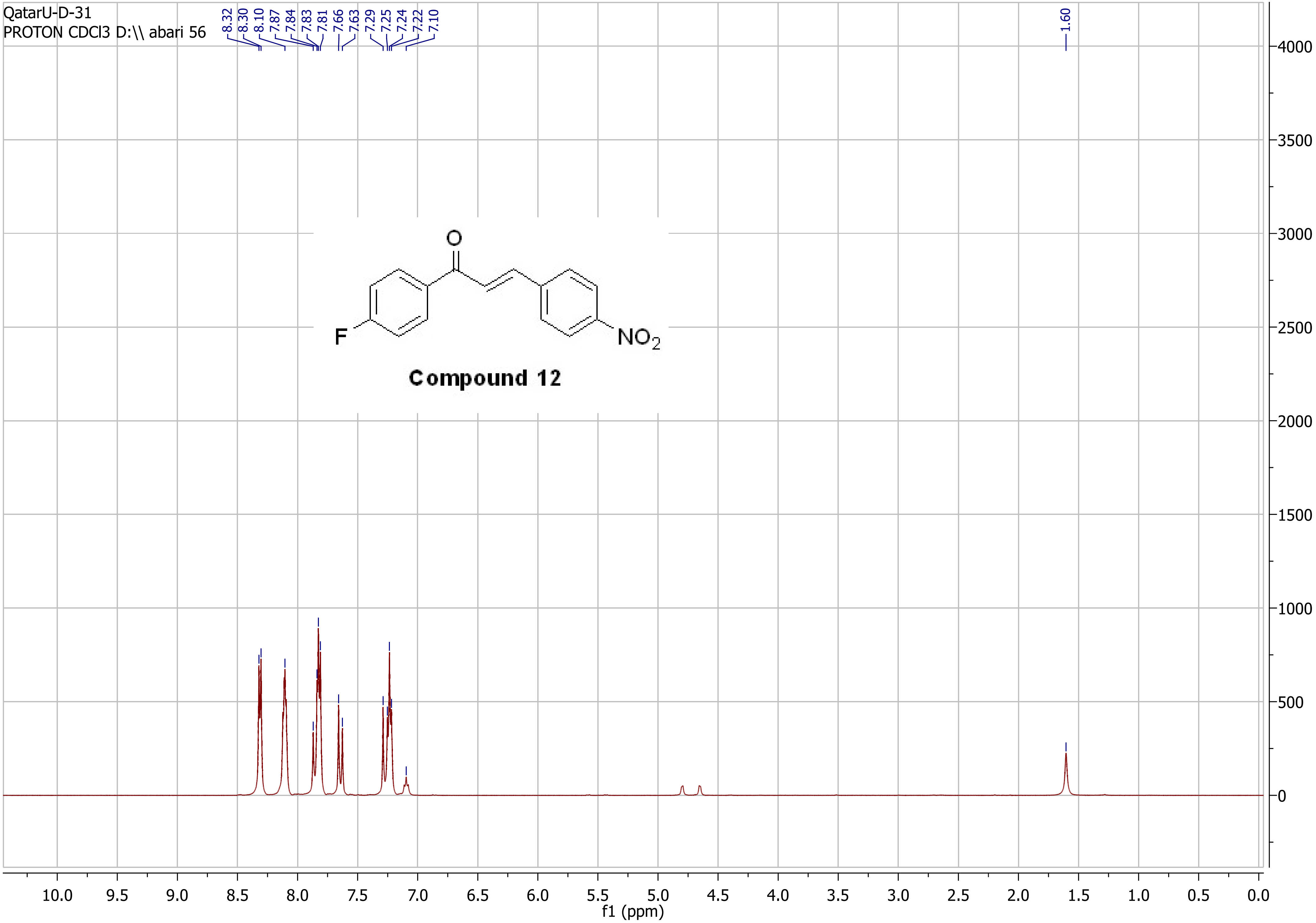
QatarU-D-31
PROTON CDCl3 D:\\ abari 56
8.32
8.30
8.10
7.87
7.84
7.83
7.81
7.66
7.63
7.29
7.25
7.24
7.22
7.10
1.60
O
F
NO2
Compound 12
4000
3500
3000
2500
2000
1500
1000
500
0
10.0
9.5
9.0
8.5
8.0
7.5
7.0
6.5
6.0
5.5
5.0
4.5
4.0
3.5
3.0
2.5
2.0
1.5
1.0
0.5
0.0
f1 (ppm)

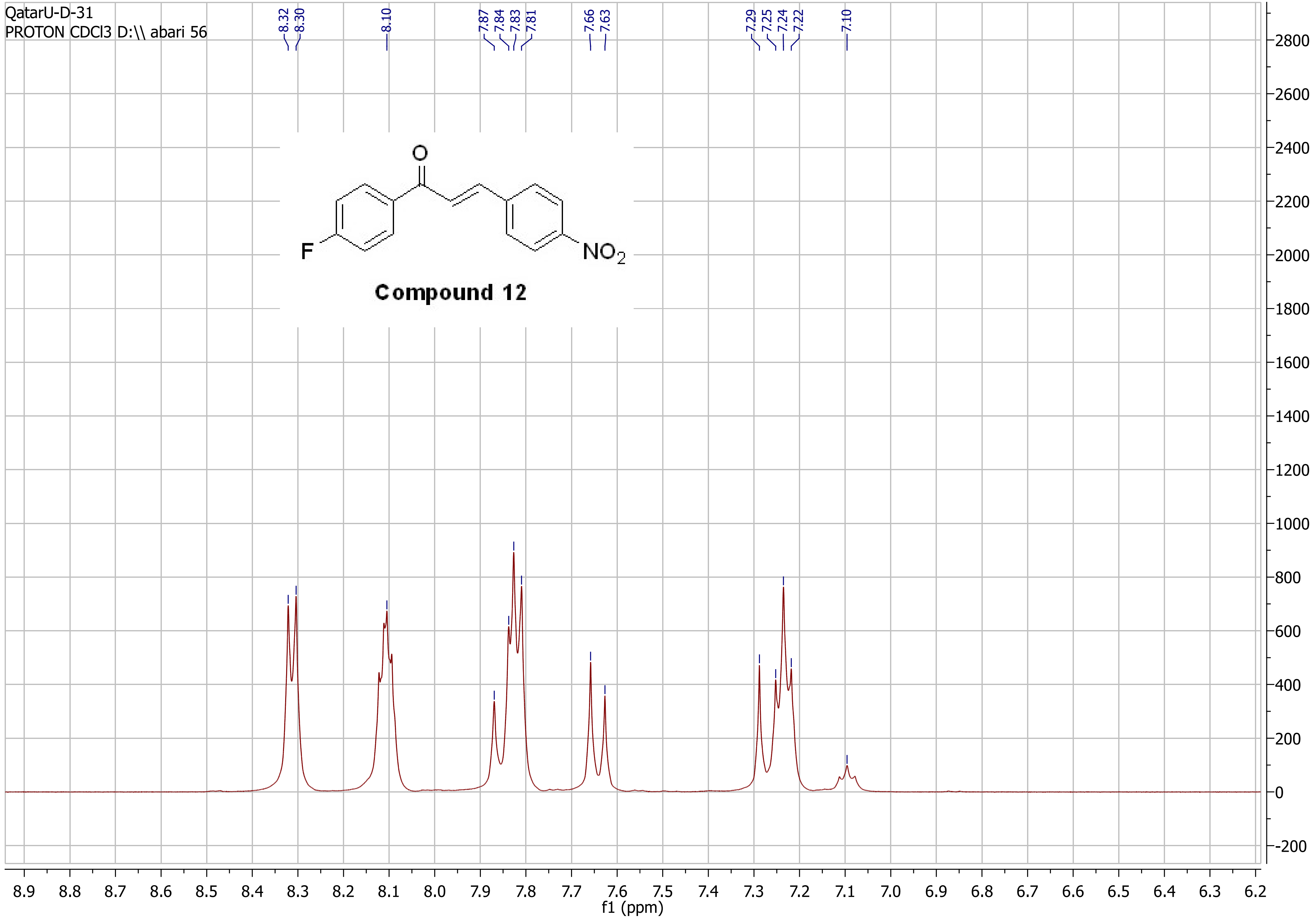
QatarU-D-31
PROTON CDCl3 D:\\ abari 56
8.32
8.30
8.10
7.87
7.84
7.83
7.81
7.66
7.63
7.29
7.25
7.24
7.22
7.10
O
F
NO2
Compound 12
2800
2600
2400
2200
2000
1800
1600
1400
1200
1000
800
600
400
200
0
-200
8.9 8.8 8.7 8.6 8.5 8.4 8.3 8.2 8.1 8.0 7.9 7.8 7.7 7.6 7.5 7.4 7.3 7.2 7.1 7.0 6.9 6.8 6.7 6.6 6.5 6.4 6.3 6.2
f1 (ppm)

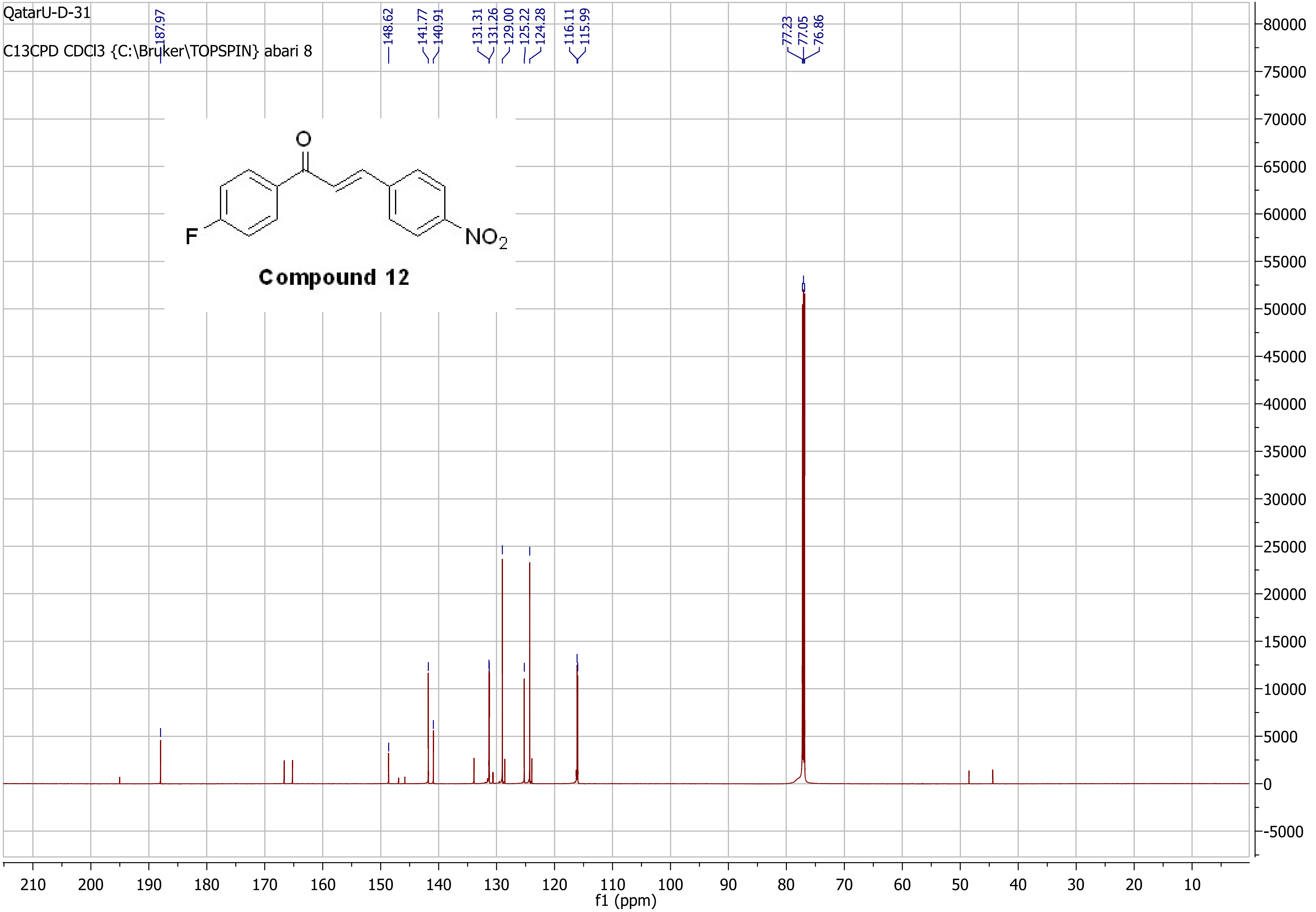
QatarU-D-31
C13CPD CDCl3 {C:\Bruker\TOPSPIN} abari 8
187.97
148.62
141.77
140.91
131.31
131.26
129.00
125.22
124.28
116.11
115.99
77.23
77.05
76.86
O
F
NO2
Compound 12
f1 (ppm)

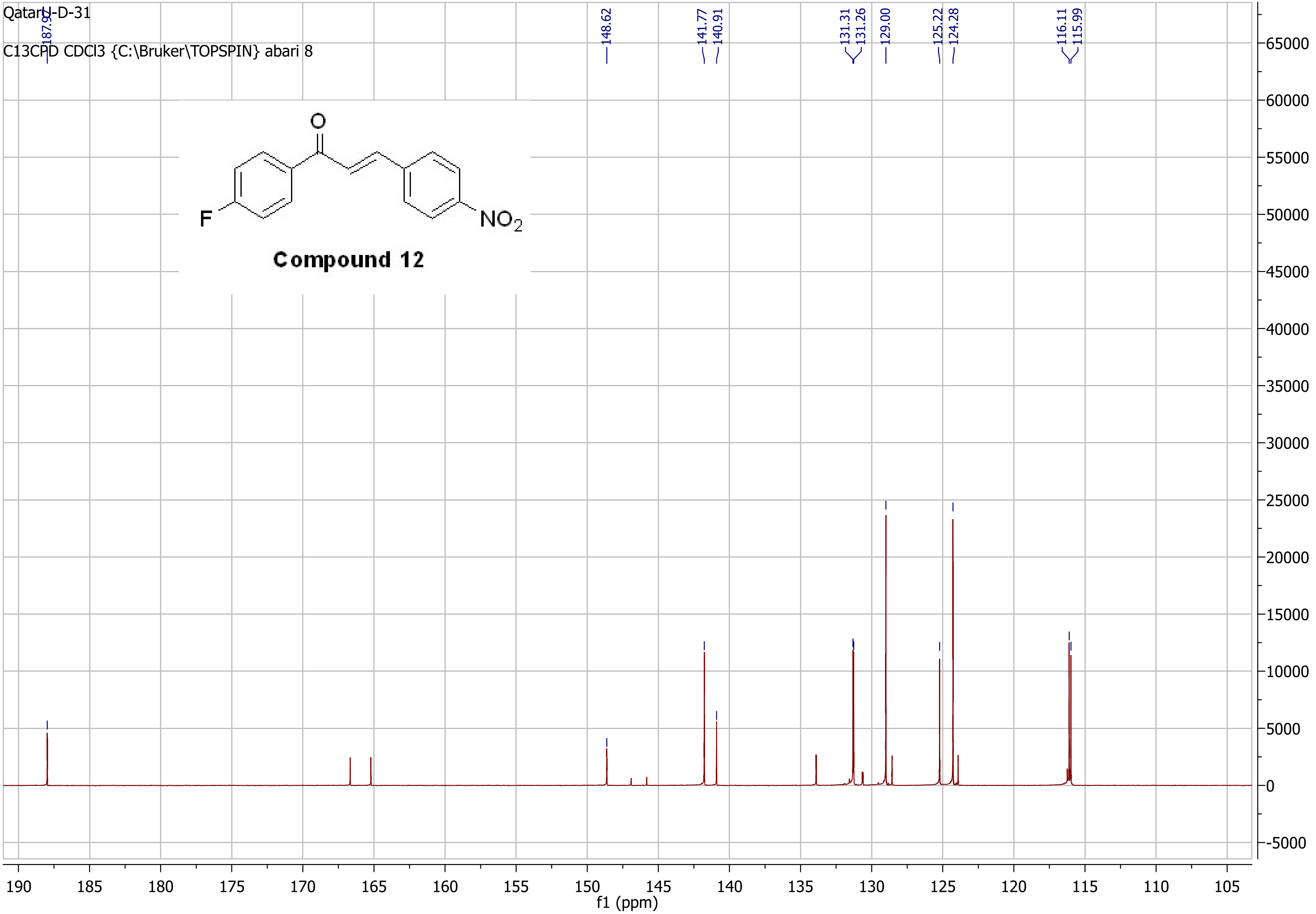

QatarU-D-31
C13CPD CDCl3 {C:\Bruker\TOPSPIN} abari 8
187.9
148.62
141.77
140.91
131.31
131.26
129.00
125.22
124.28
116.11
115.99
O
F
NO_2
Compound 12
f1 (ppm)

D31

^{1}H NMR (400 MHz, $CDCl_3$) δ 8.27 (d, *J* = 8.7 Hz, 2H), 8.06 (dd, *J* = 8.7, 5.4 Hz, 2H), 7.81 (d, *J* = 15.8 Hz, 1H), 7.77 (d, *J* = 8.8 Hz, 2H), 7.60 (d, *J* = 15.7 Hz, 1H), 7.19 (t, *J* = 8.5 Hz, 2H).

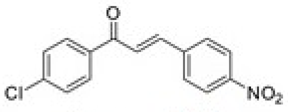

Compound 13

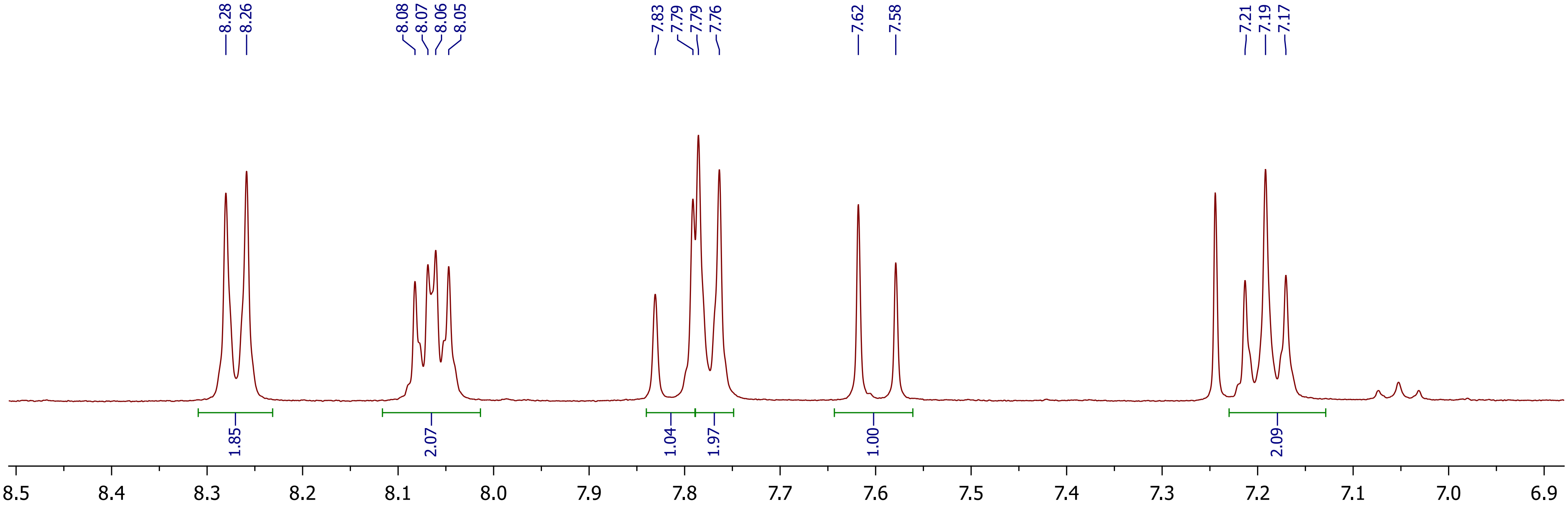

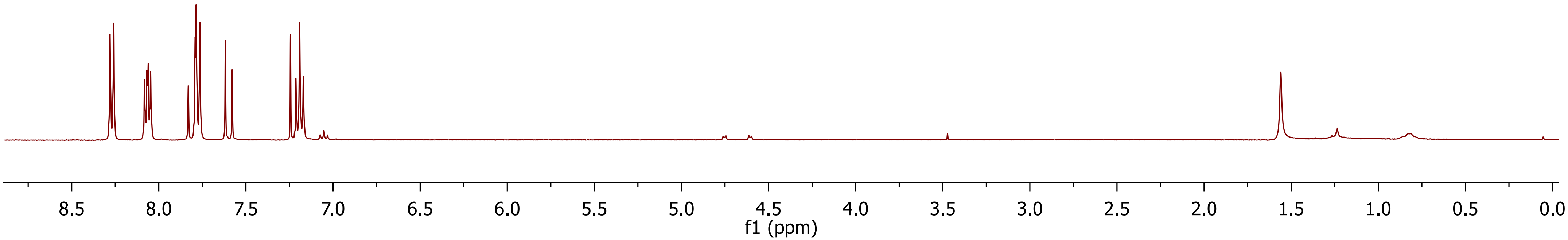

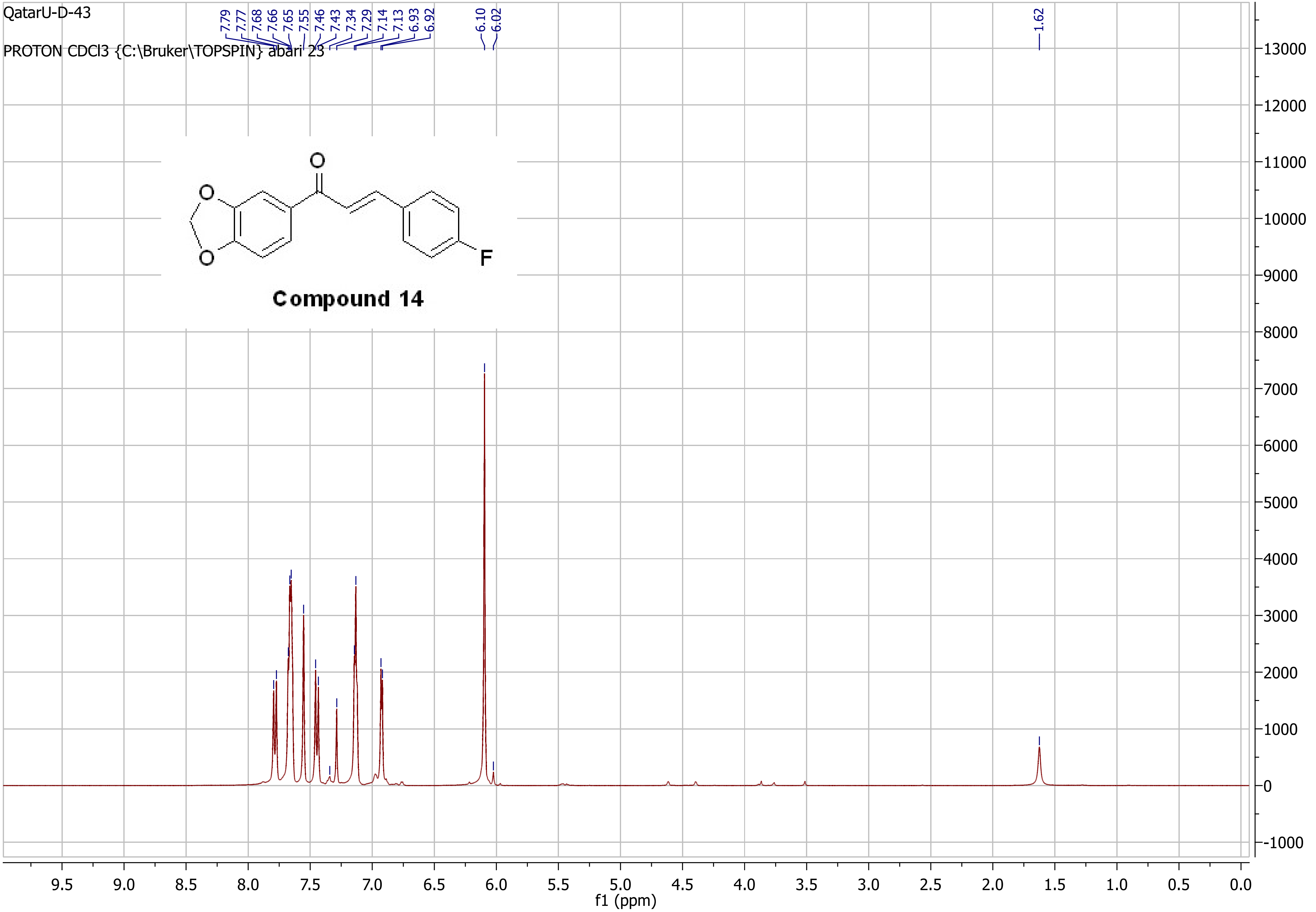

QatarU-D-43
PROTON CDCl3 {C:\Bruker\TOPSPIN} abari 23
7.79
7.77
7.68
7.66
7.65
7.55
7.46
7.43
7.34
7.29
7.14
7.13
6.93
6.92
6.10
6.02
1.62
O
O
O
F
Compound 14
f1 (ppm)

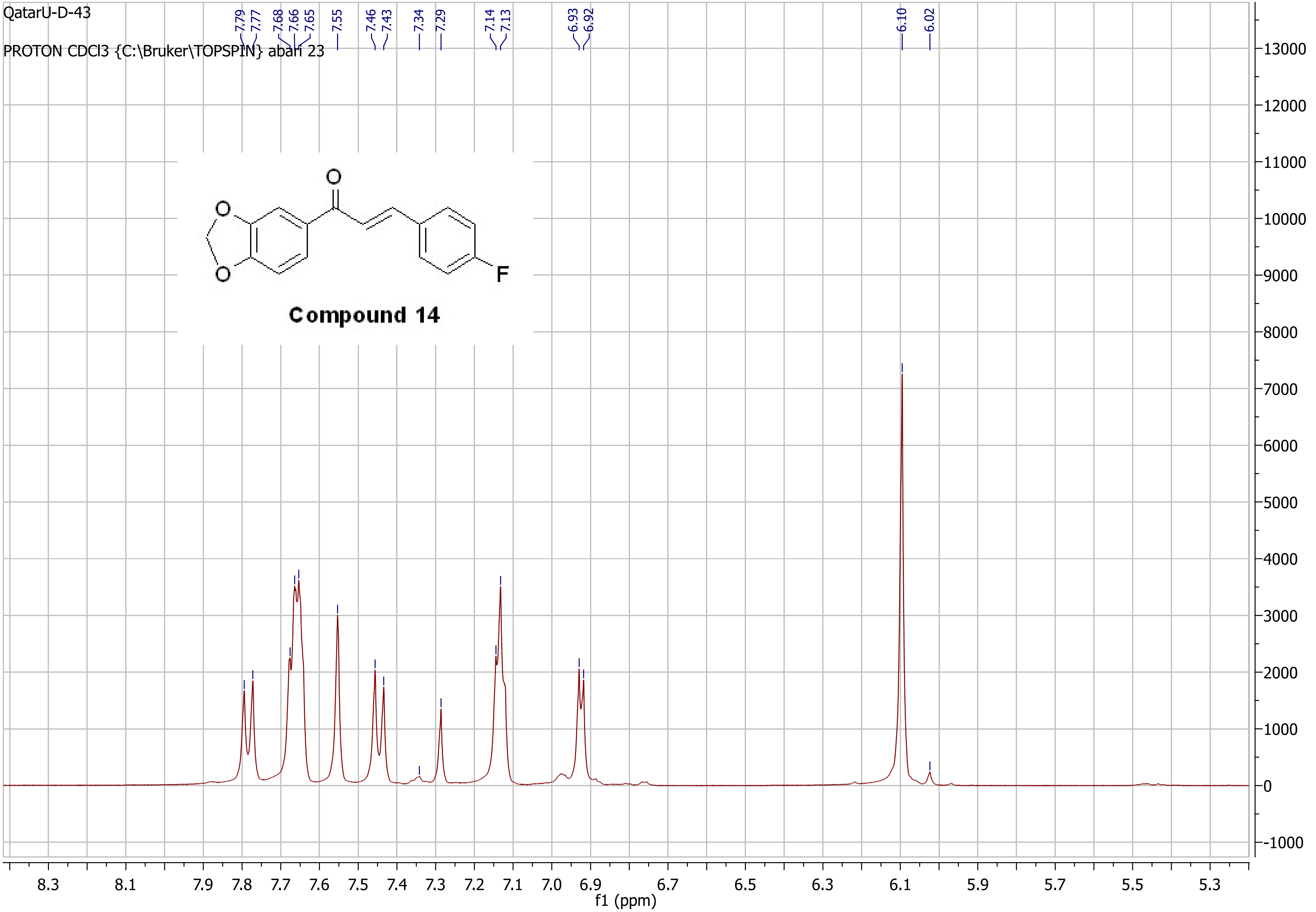
QatarU-D-43
PROTON CDCl3 {C:\Bruker\TOPSPIN} abari 23
7.79
7.77
7.68
7.66
7.65
7.55
7.46
7.43
7.34
7.29
7.14
7.13
6.93
6.92
6.10
6.02
O
O
O
F
Compound 14
13000
12000
11000
10000
9000
8000
7000
6000
5000
4000
3000
2000
1000
0
-1000
8.3
8.1
7.9
7.8
7.7
7.6
7.5
7.4
7.3
7.2
7.1
7.0
6.9
6.7
6.5
6.3
6.1
5.9
5.7
5.5
5.3
f1 (ppm)

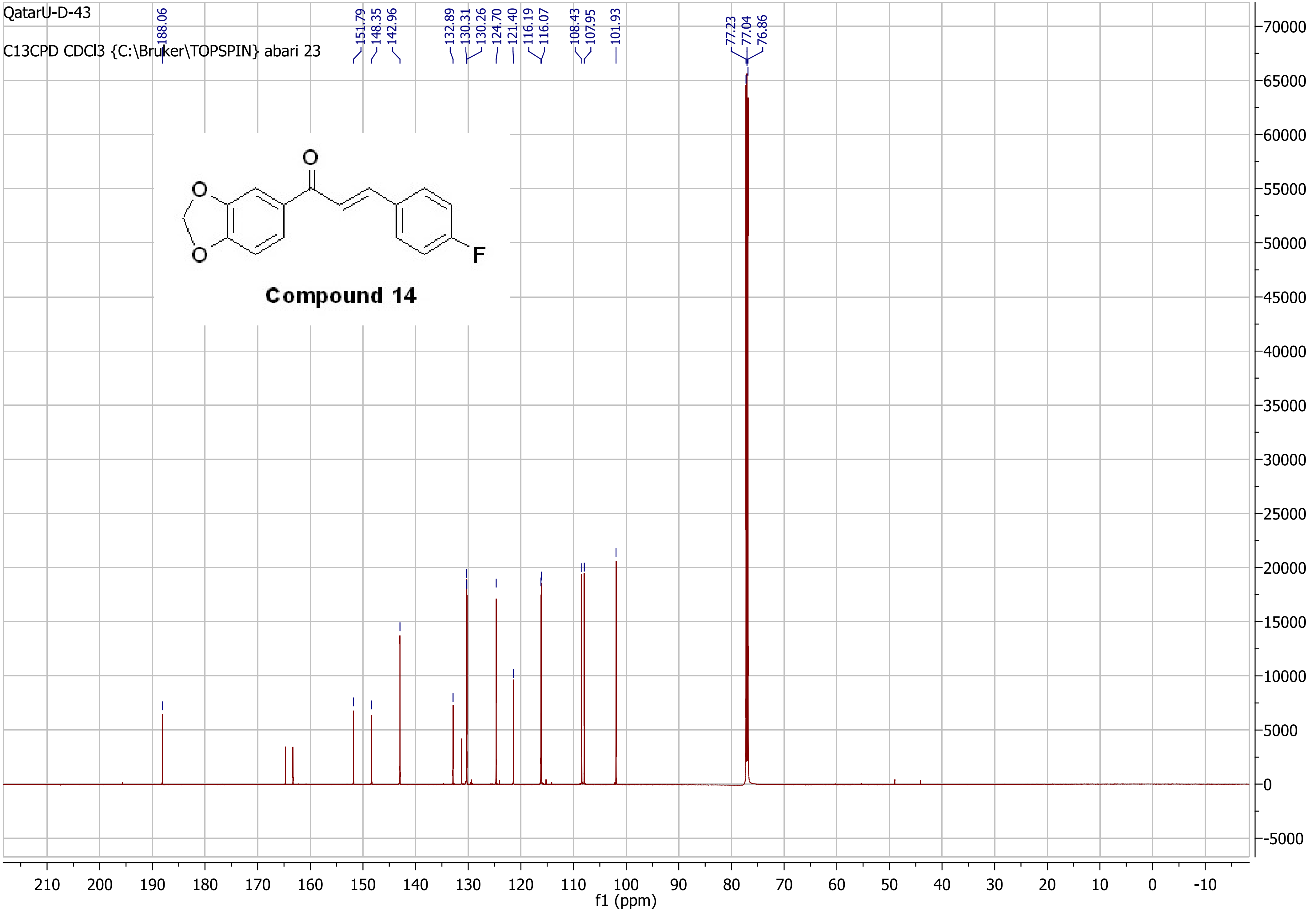
QatarU-D-43
C13CPD CDCl3 {C:\Bruker\TOPSPIN} abari 23
188.06
151.79
148.35
142.96
132.89
130.31
130.26
124.70
121.40
116.19
116.07
108.43
107.95
101.93
77.23
77.04
76.86
O
O
O
F
Compound 14
f1 (ppm)

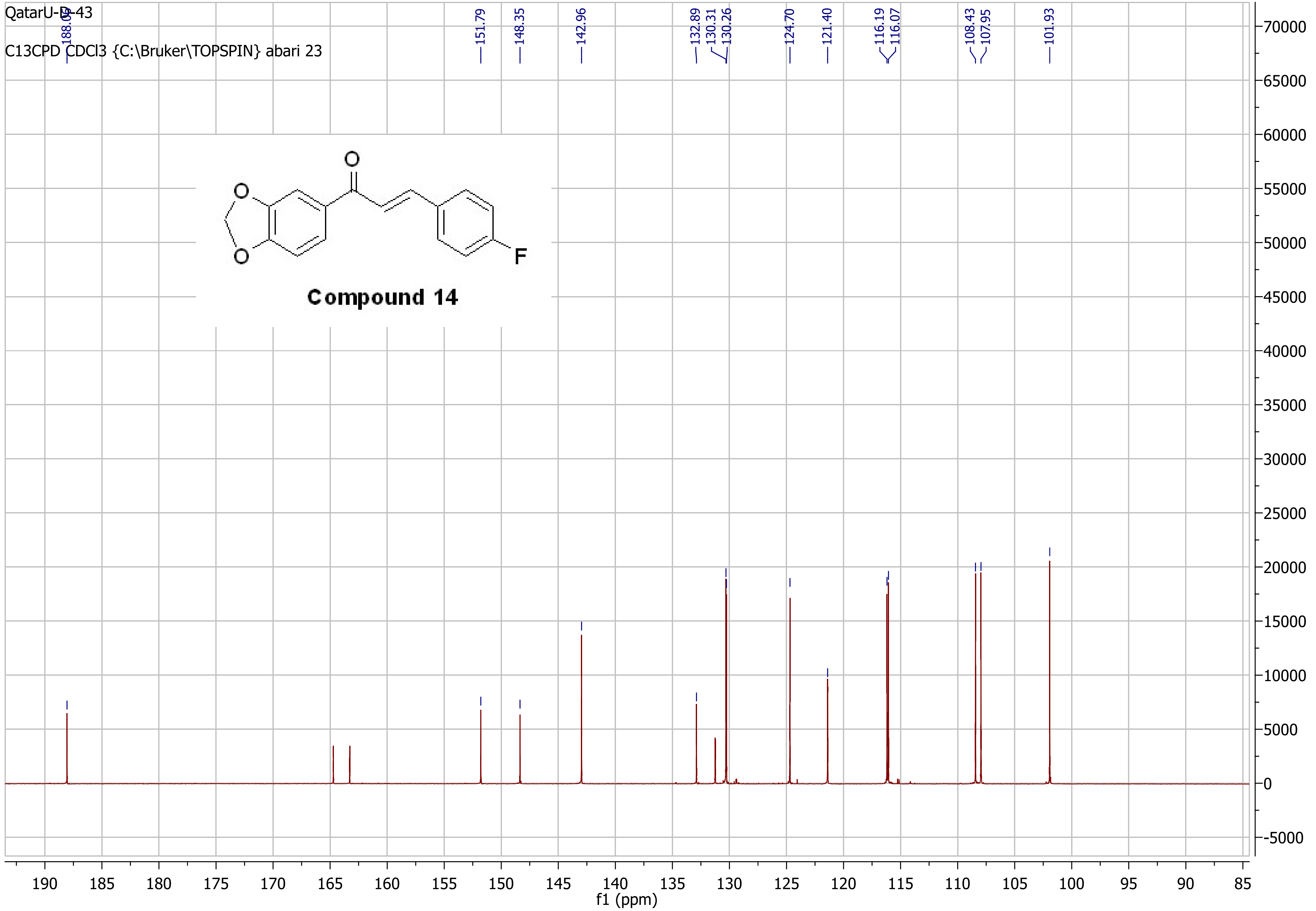

QatarU-D-43
C13CPD CDCl3 {C:\Bruker\TOPSPIN} abari 23
188.06
151.79
148.35
142.96
132.89
130.31
130.26
124.70
121.40
116.19
116.07
108.43
107.95
101.93
O
O
O
F
Compound 14
f1 (ppm)

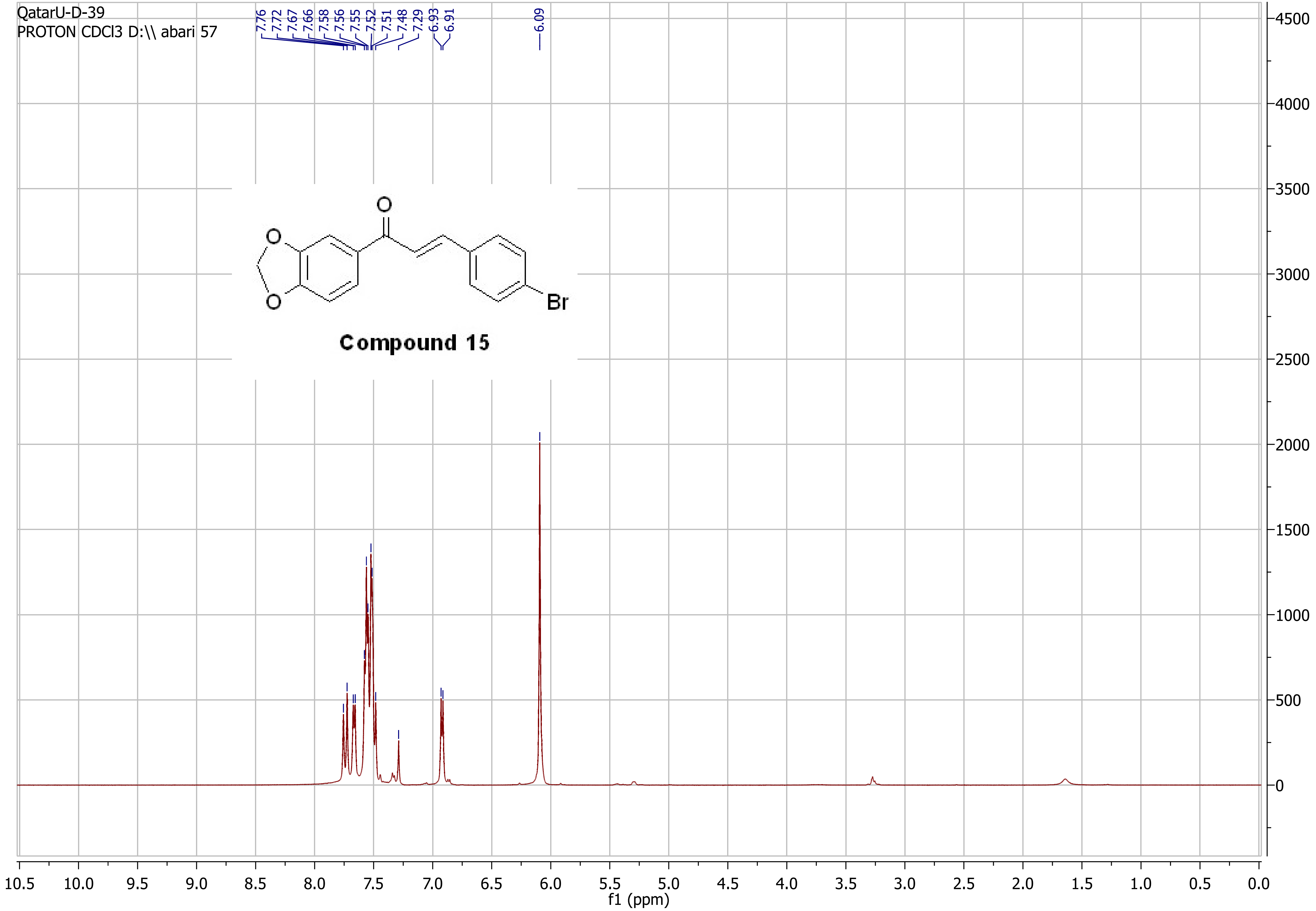

QatarU-D-39
PROTON CDCl3 D:\\ abari 57
7.76
7.72
7.67
7.66
7.58
7.56
7.55
7.52
7.51
7.48
7.29
6.93
6.91
6.09
O
O
O
Br
Compound 15
f1 (ppm)

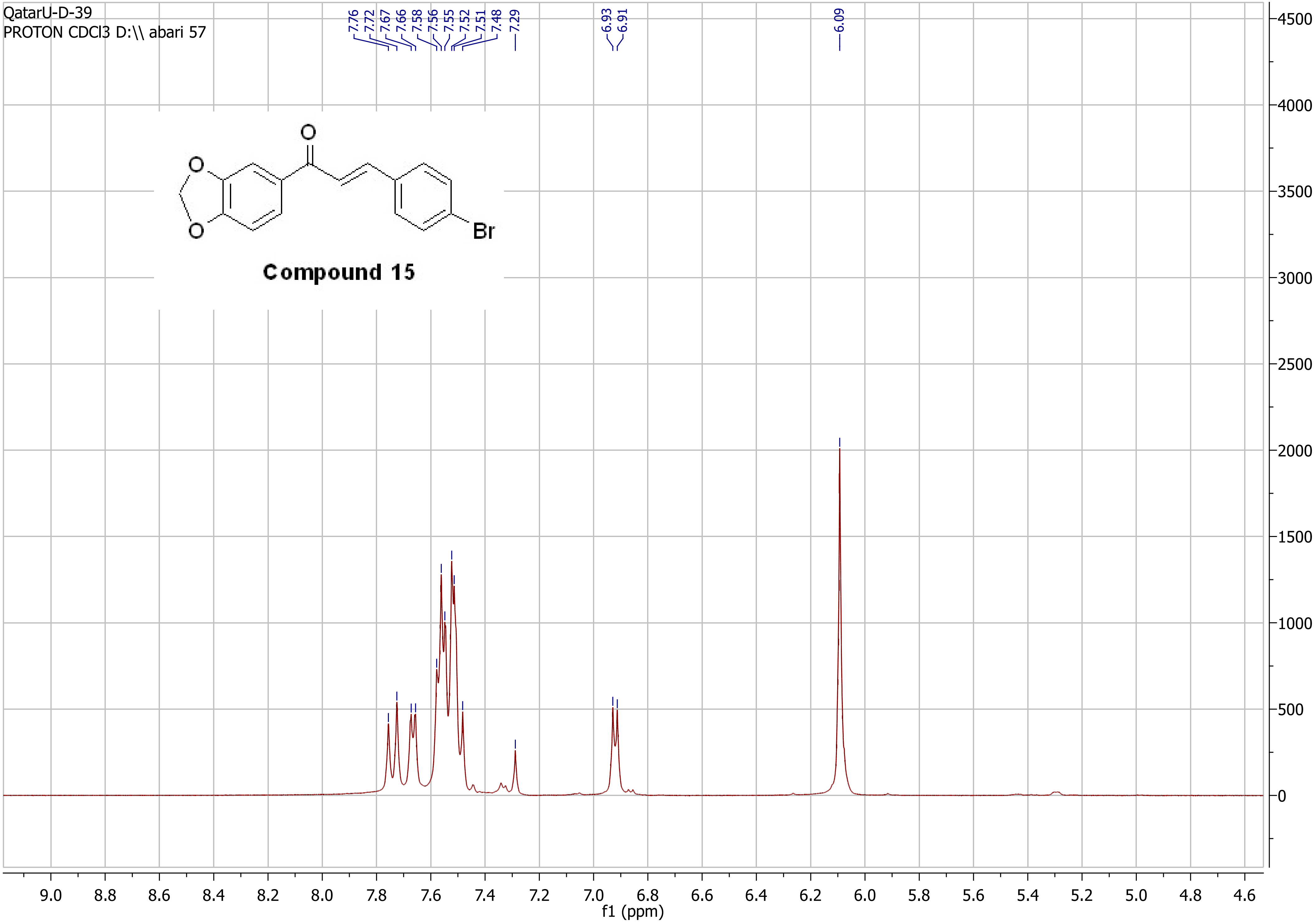
QatarU-D-39
PROTON CDCl3 D:\\ abari 57
7.76
7.72
7.67
7.66
7.58
7.56
7.55
7.52
7.51
7.48
7.29
6.93
6.91
6.09
O
O
O
Br
Compound 15
4500
4000
3500
3000
2500
2000
1500
1000
500
0
9.0
8.8
8.6
8.4
8.2
8.0
7.8
7.6
7.4
7.2
7.0
6.8
6.6
6.4
6.2
6.0
5.8
5.6
5.4
5.2
5.0
4.8
4.6
f1 (ppm)

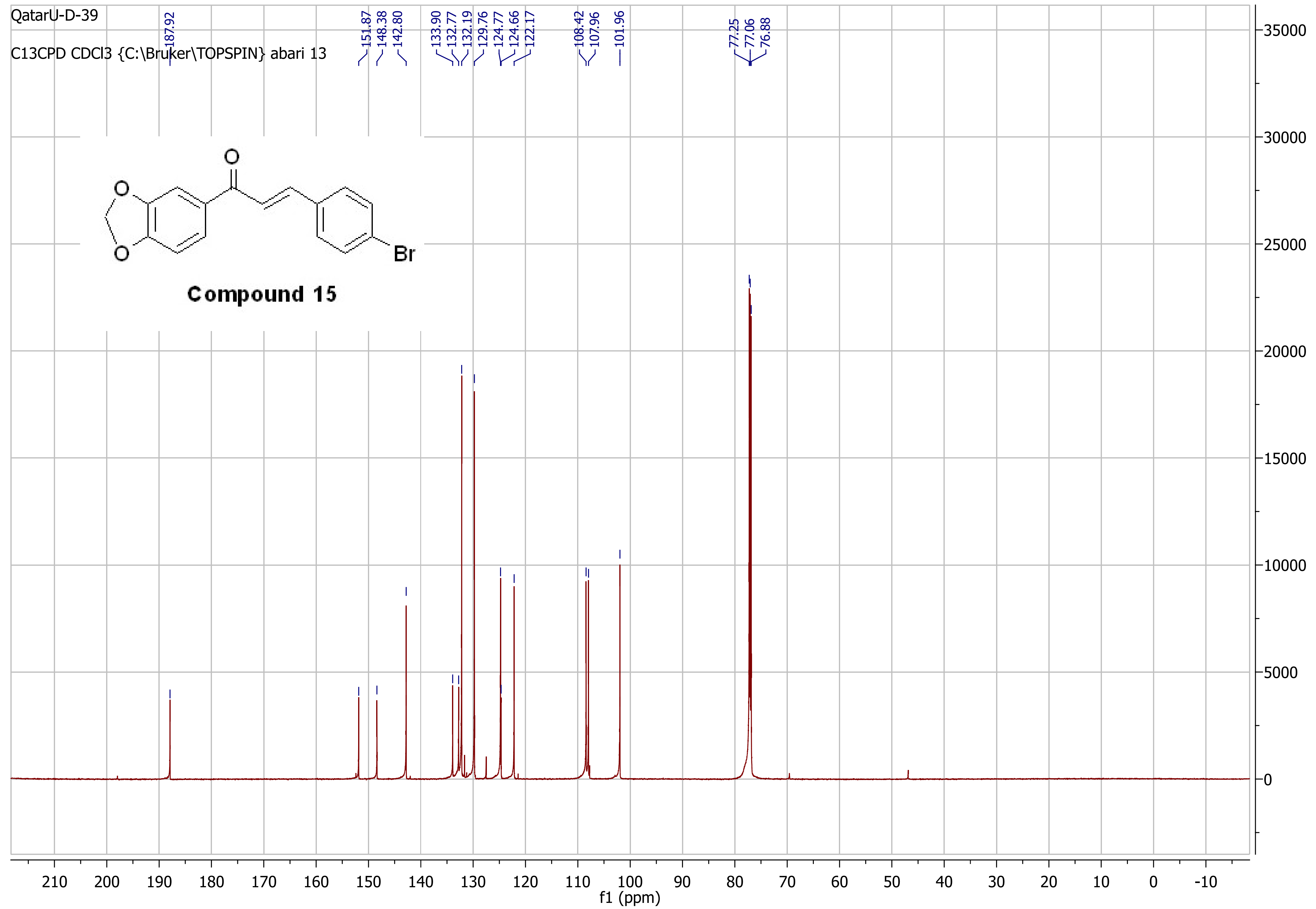
QatarU-D-39
C13CPD CDCl3 {C:\Bruker\TOPSPIN} abari 13
187.92
151.87
148.38
142.80
133.90
132.77
132.19
129.76
124.77
124.66
122.17
108.42
107.96
101.96
77.25
77.06
76.88
O
O
O
Br
Compound 15
f1 (ppm)

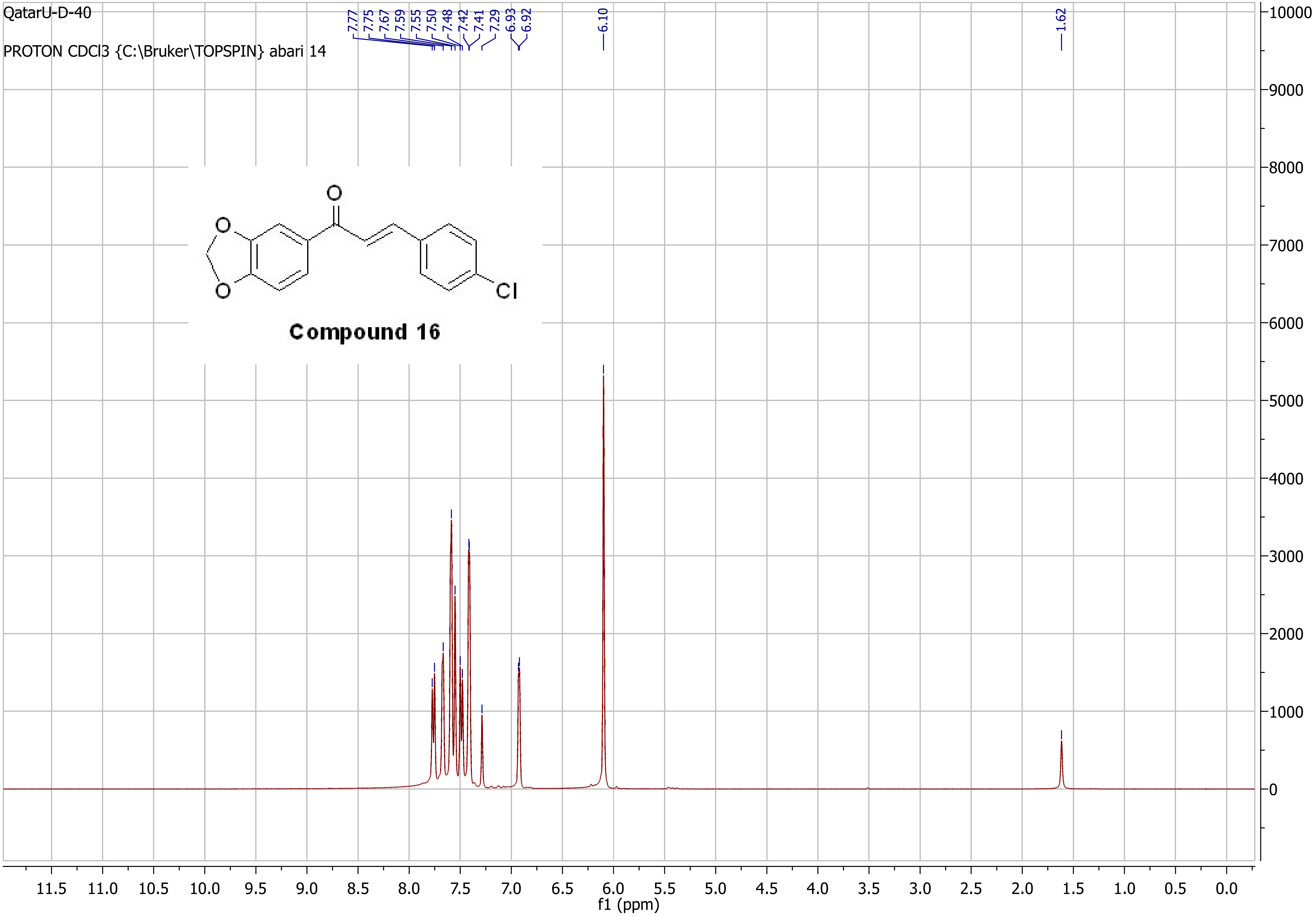
QatarU-D-40
PROTON CDCl3 {C:\Bruker\TOPSPIN} abari 14
7.77
7.75
7.67
7.59
7.55
7.50
7.48
7.42
7.41
7.29
6.93
6.92
6.10
1.62
O
O
O
Cl
Compound 16
f1 (ppm)

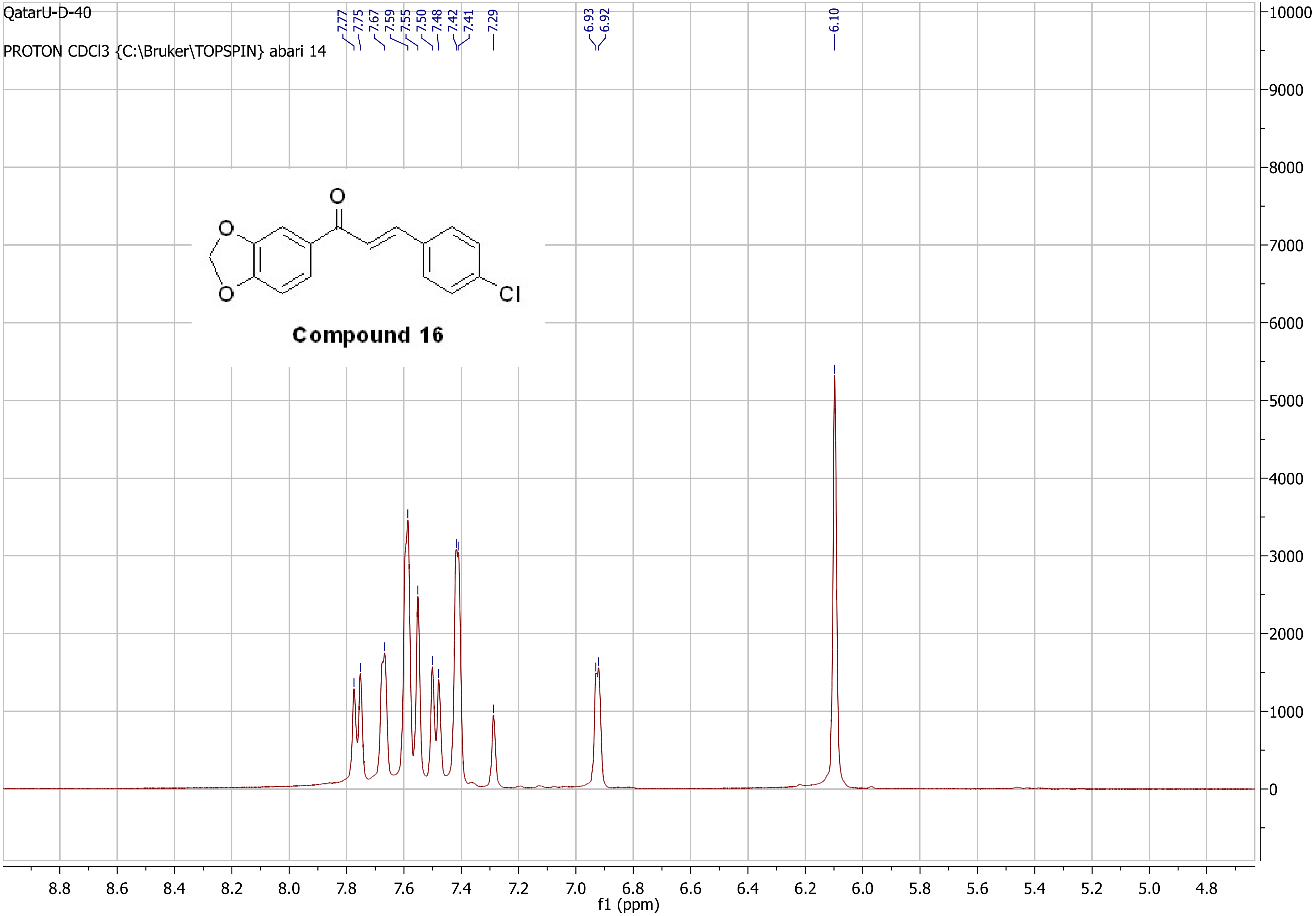

QatarU-D-40
PROTON CDCl3 {C:\Bruker\TOPSPIN} abari 14
7.77
7.75
7.67
7.59
7.55
7.50
7.48
7.42
7.41
7.29
6.93
6.92
6.10
O
O
O
Cl
Compound 16
f1 (ppm)

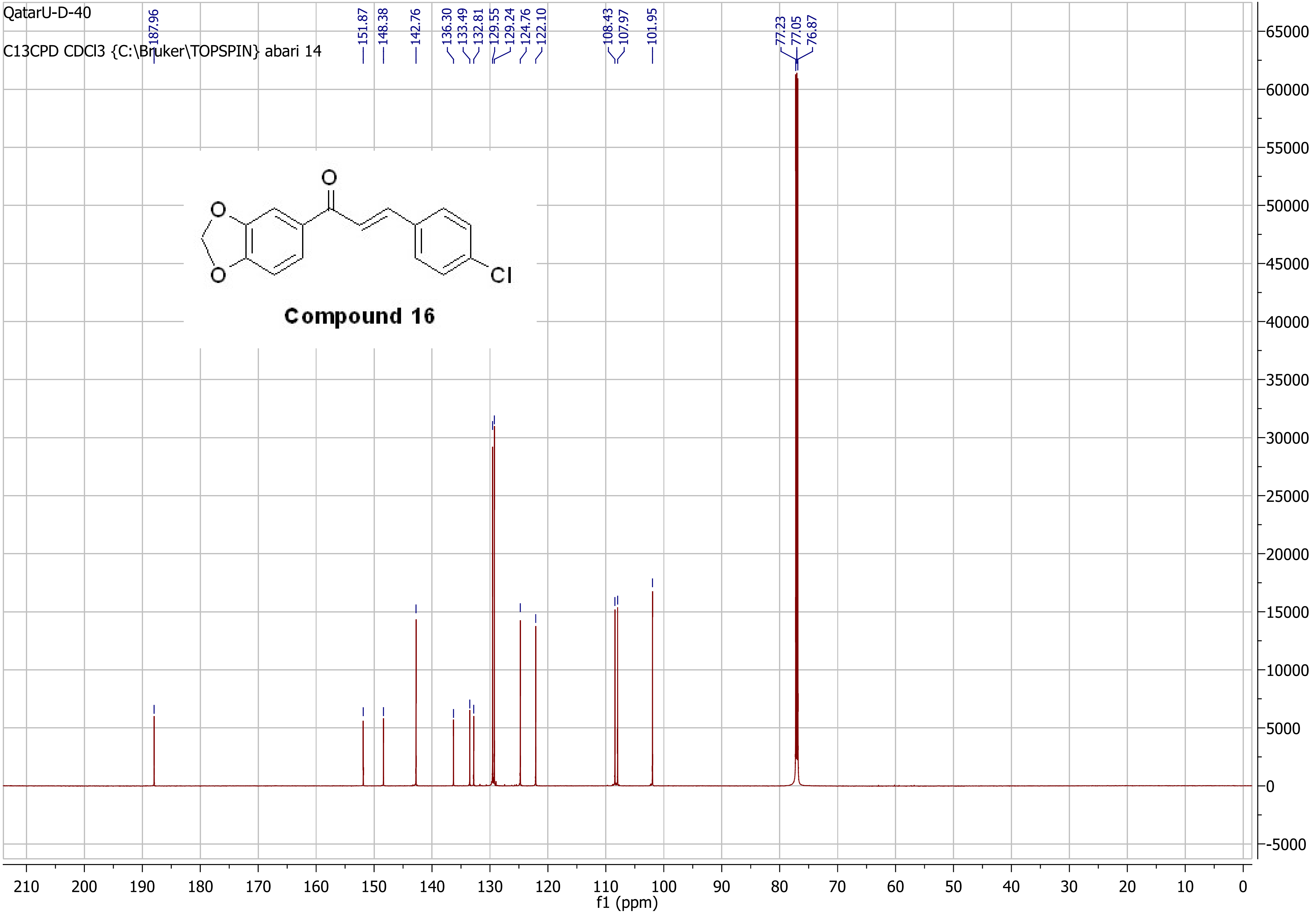
QatarU-D-40
C13CPD CDCl3 {C:\Bruker\TOPSPIN} abari 14
187.96
151.87
148.38
142.76
136.30
133.49
132.81
129.55
129.24
124.76
122.10
108.43
107.97
101.95
77.23
77.05
76.87
O
O
O
Cl
Compound 16
f1 (ppm)

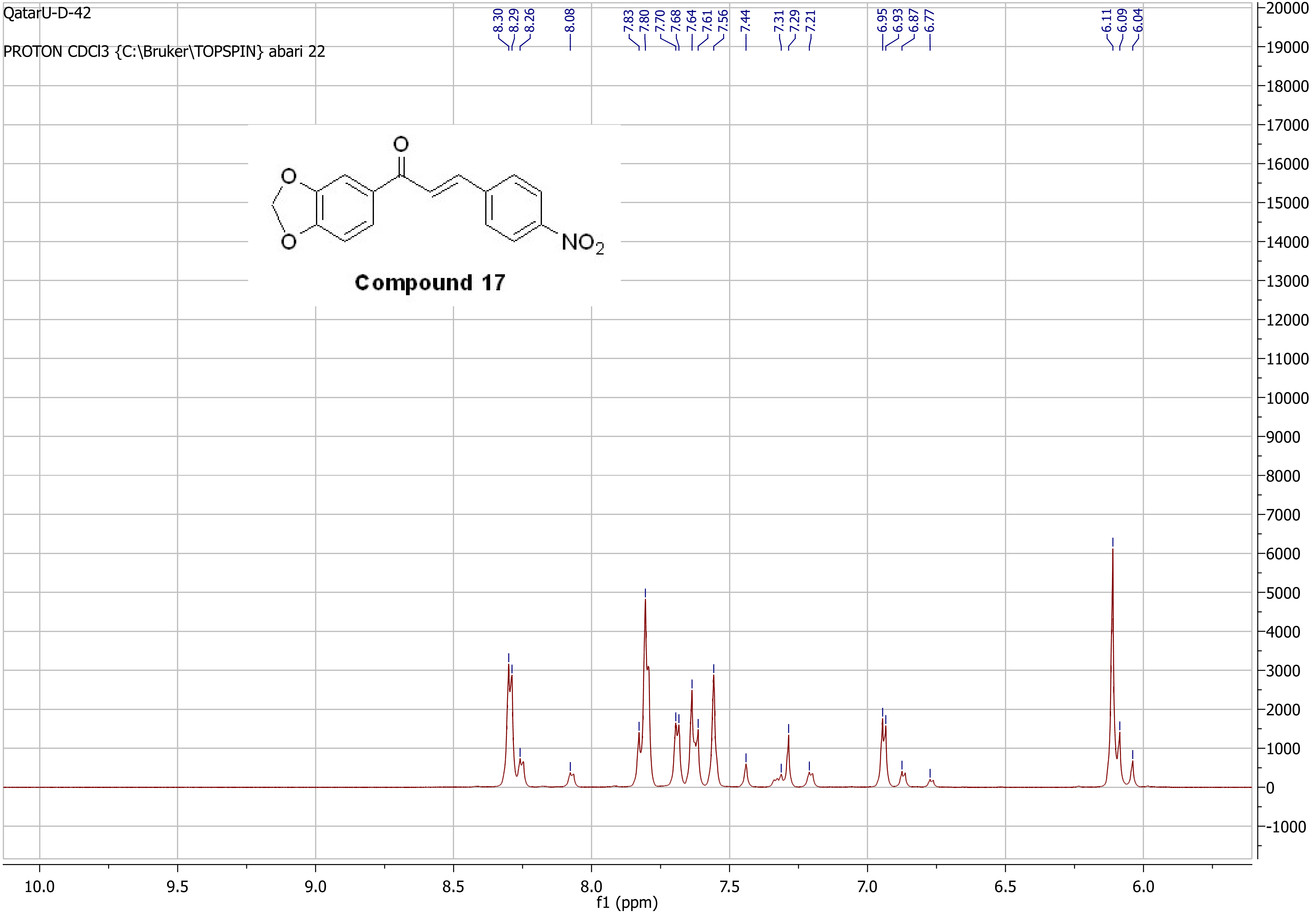
QatarU-D-42
PROTON CDCl3 {C:\Bruker\TOPSPIN} abari 22
8.30
8.29
8.26
8.08
7.83
7.80
7.70
7.68
7.64
7.61
7.56
7.44
7.31
7.29
7.21
6.95
6.93
6.87
6.77
6.11
6.09
6.04
O
O
O
NO2
Compound 17
20000
19000
18000
17000
16000
15000
14000
13000
12000
11000
10000
9000
8000
7000
6000
5000
4000
3000
2000
1000
0
-1000
10.0
9.5
9.0
8.5
8.0
7.5
7.0
6.5
6.0
f1 (ppm)

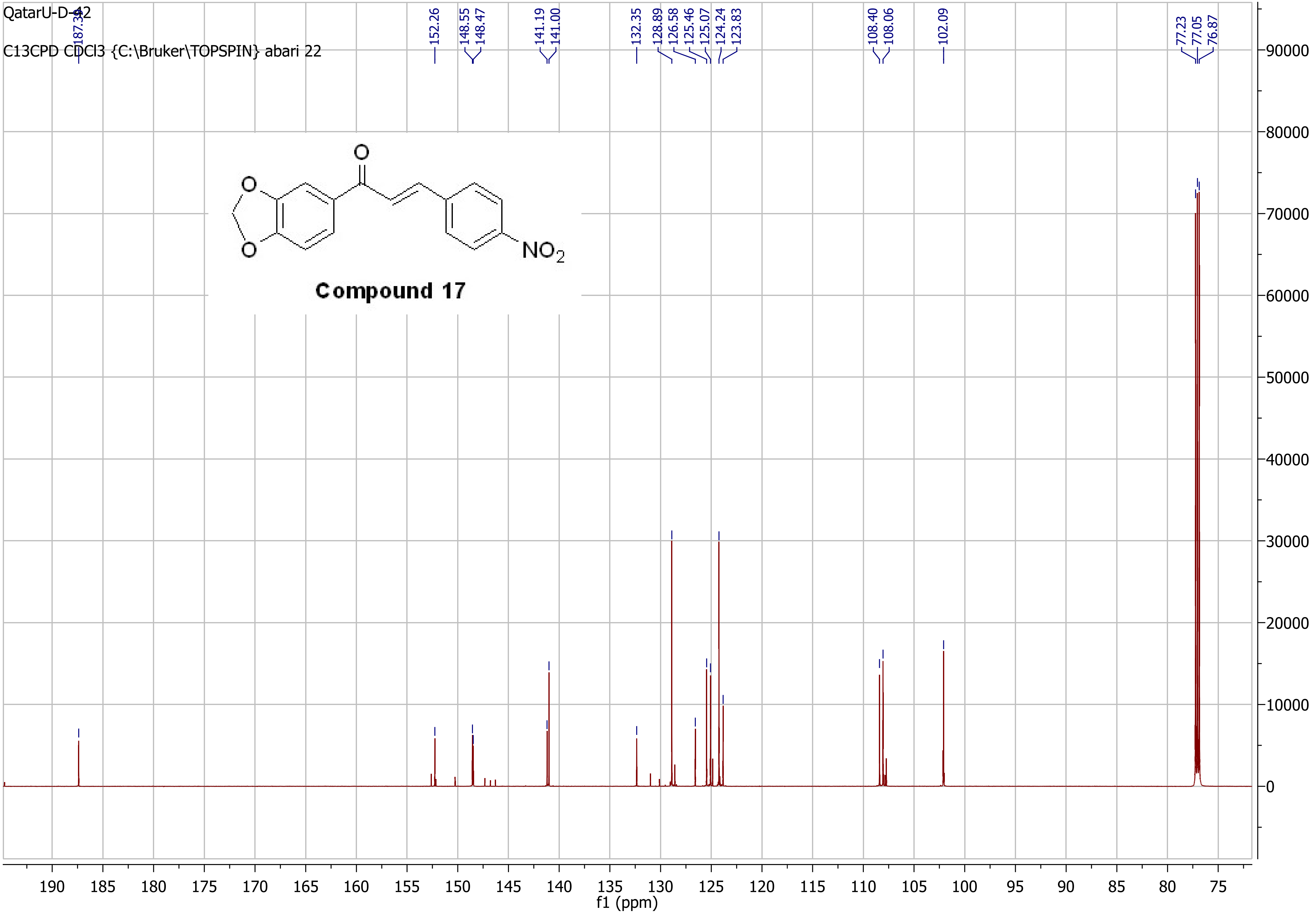
QatarU-D-42
C13CPD CDCl3 {C:\Bruker\TOPSPIN} abari 22
187.34
152.26
148.55
148.47
141.19
141.00
132.35
128.89
126.58
125.46
125.07
124.24
123.83
108.40
108.06
102.09
77.23
77.05
76.87
O
O
O
NO2
Compound 17
f1 (ppm)
190 185 180 175 170 165 160 155 150 145 140 135 130 125 120 115 110 105 100 95 90 85 80 75
90000 80000 70000 60000 50000 40000 30000 20000 10000 0

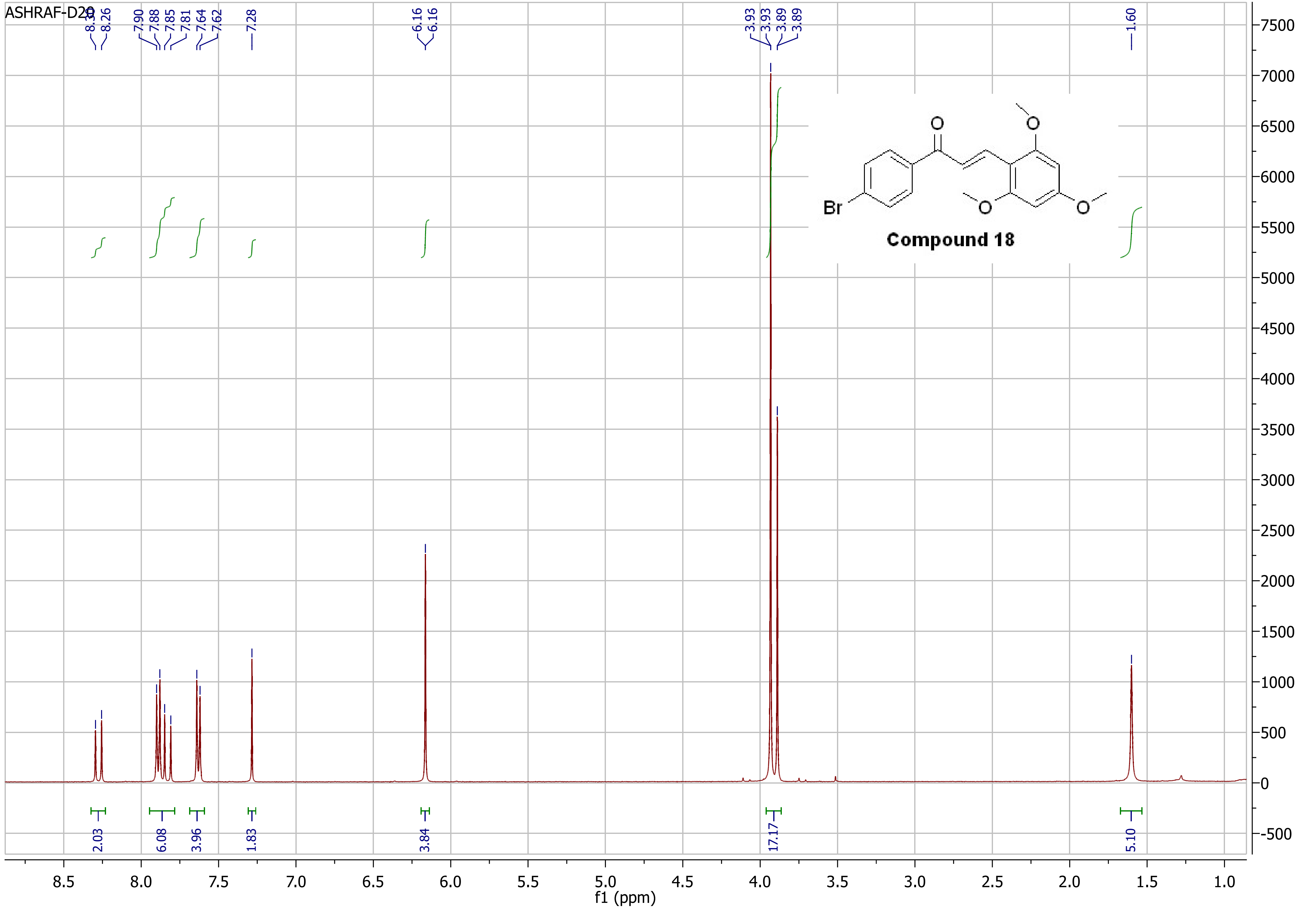
ASHRAF-D20
8.26
7.90
7.88
7.85
7.81
7.64
7.62
7.28
6.16
6.16
3.93
3.93
3.89
3.89
1.60
O
O
Br
O
O
Compound 18
2.03
6.08
3.96
1.83
3.84
17.17
5.10
7500
7000
6500
6000
5500
5000
4500
4000
3500
3000
2500
2000
1500
1000
500
0
-500
8.5
8.0
7.5
7.0
6.5
6.0
5.5
5.0
4.5
4.0
3.5
3.0
2.5
2.0
1.5
1.0
f1 (ppm)

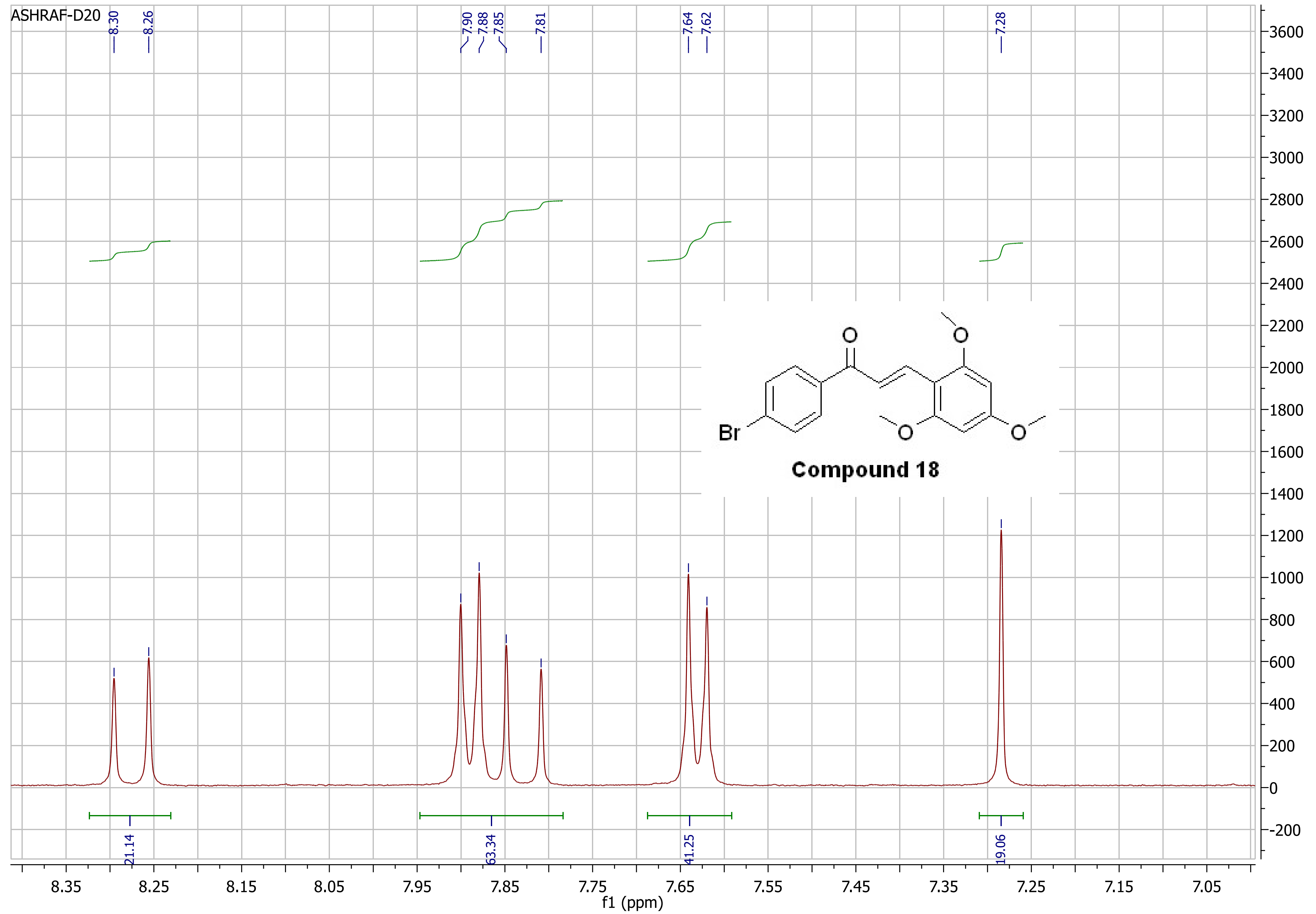
ASHRAF-D20
8.30
8.26
7.90
7.88
7.85
7.81
7.64
7.62
7.28
Br
O
Compound 18
21.14
63.34
41.25
19.06
f1 (ppm)

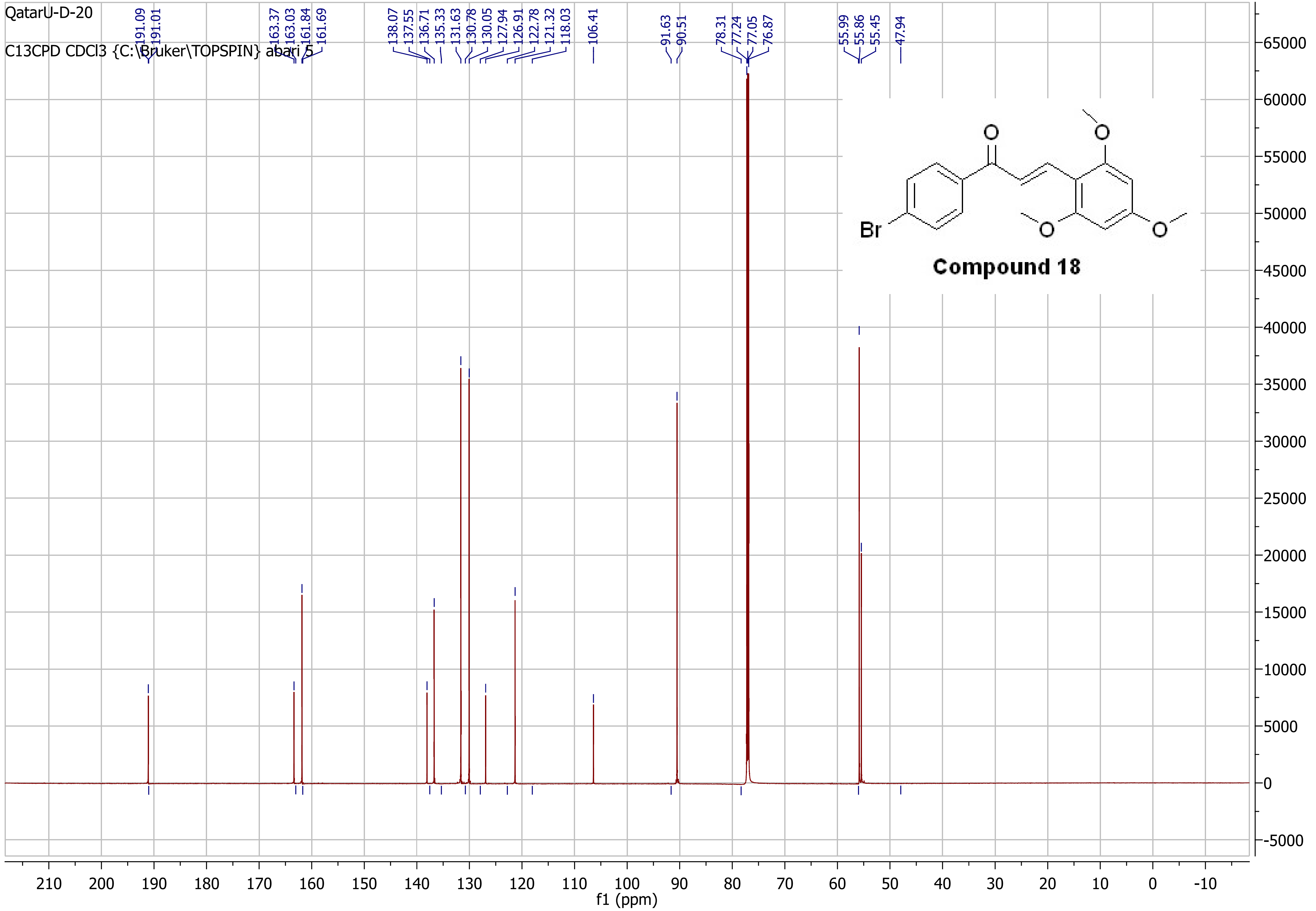
QatarU-D-20
C13CPD CDCl3 {C:\Bruker\TOPSPIN} abari 5
191.09
191.01
163.37
163.03
161.84
161.69
138.07
137.55
136.71
135.33
131.63
130.78
130.05
127.94
126.91
122.78
121.32
118.03
106.41
91.63
90.51
78.31
77.24
77.05
76.87
55.99
55.86
55.45
47.94
O
O
O
O
Br
Compound 18
65000
60000
55000
50000
45000
40000
35000
30000
25000
20000
15000
10000
5000
0
-5000
210 200 190 180 170 160 150 140 130 120 110 100 90 80 70 60 50 40 30 20 10 0 -10
f1 (ppm)

D20

^{1}H NMR (400 MHz, $CDCl_3$) δ 8.25 (d, *J* = 15.8 Hz, 1H), 7.87 (d, *J* = 8.4 Hz, 2H), 7.80 (d, *J* = 15.9 Hz, 1H), 7.61 (d, *J* = 8.4 Hz, 2H), 6.14 (s, 2H), 3.91 (s, 6H), 3.87 (s, 3H).

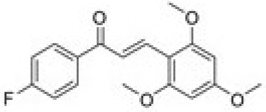

Compound 19

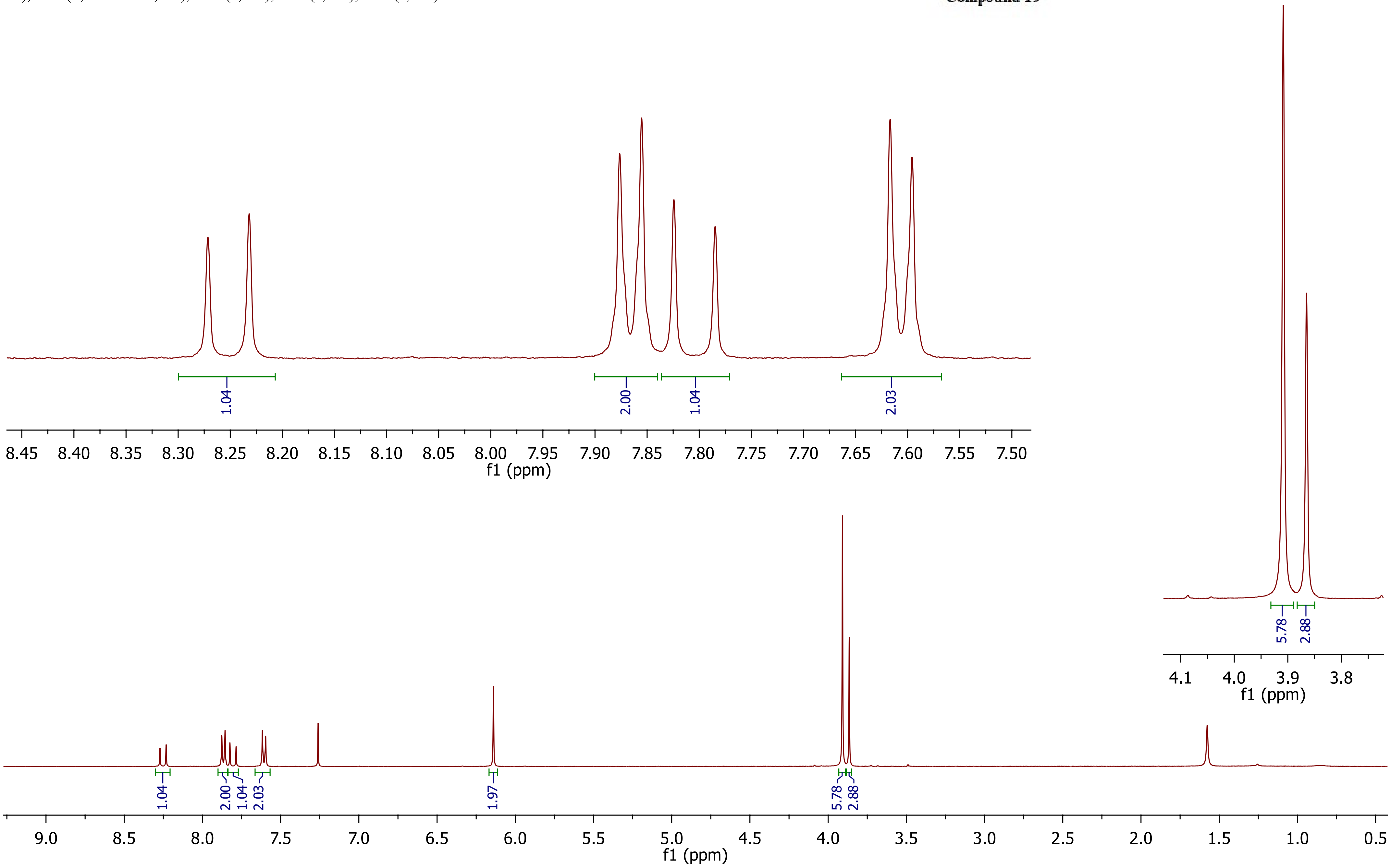

D20

^{13}C NMR (101 MHz, $CDCl_3$) δ 191.11 (s), 163.36 (s), 161.85 (s), 138.09 (s), 136.69 (s), 131.62 (s), 130.05 (s), 121.41 (s), 106.48 (s), 90.55 (s), 90.55 (s), 55.86 (s), 55.44 (s).

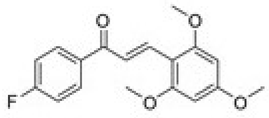

Compound 19

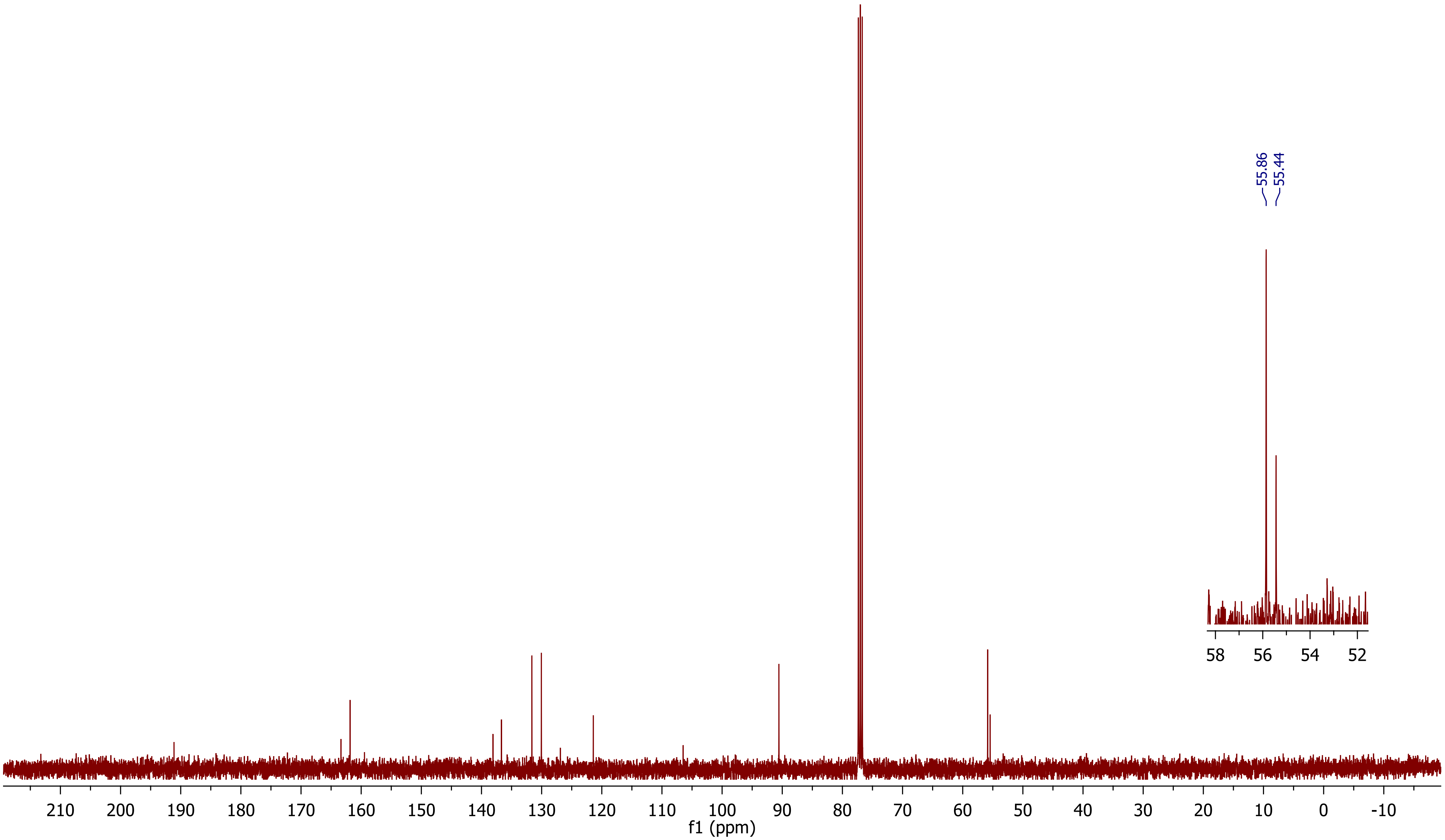

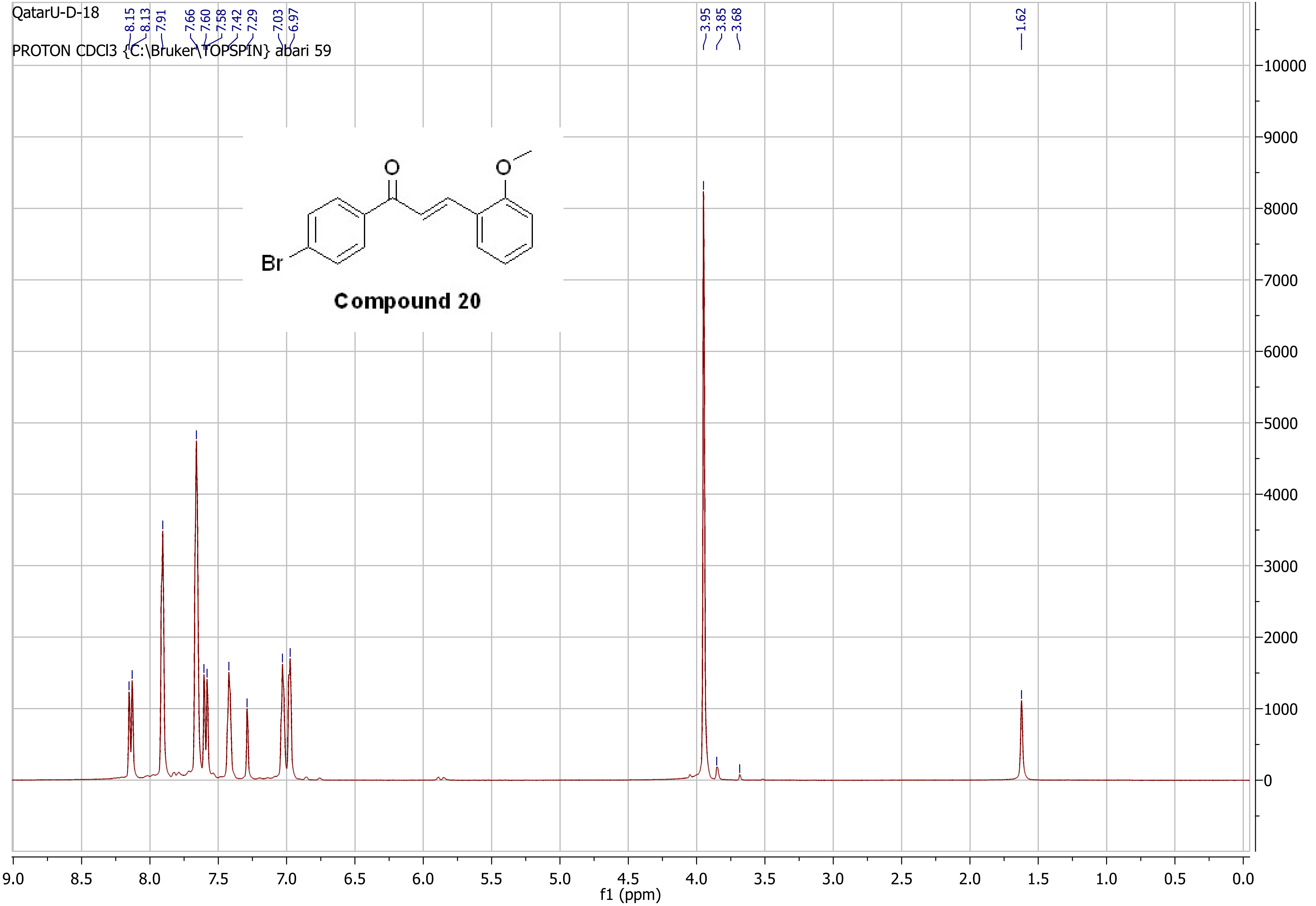
QatarU-D-18
PROTON CDCl3 {C:\Bruker\TOPSPIN} abari 59
8.15
8.13
7.91
7.66
7.60
7.58
7.42
7.29
7.03
6.97
3.95
3.85
3.68
1.62
O
O
Br
Compound 20
10000
9000
8000
7000
6000
5000
4000
3000
2000
1000
0
9.0 8.5 8.0 7.5 7.0 6.5 6.0 5.5 5.0 4.5 4.0 3.5 3.0 2.5 2.0 1.5 1.0 0.5 0.0
f1 (ppm)

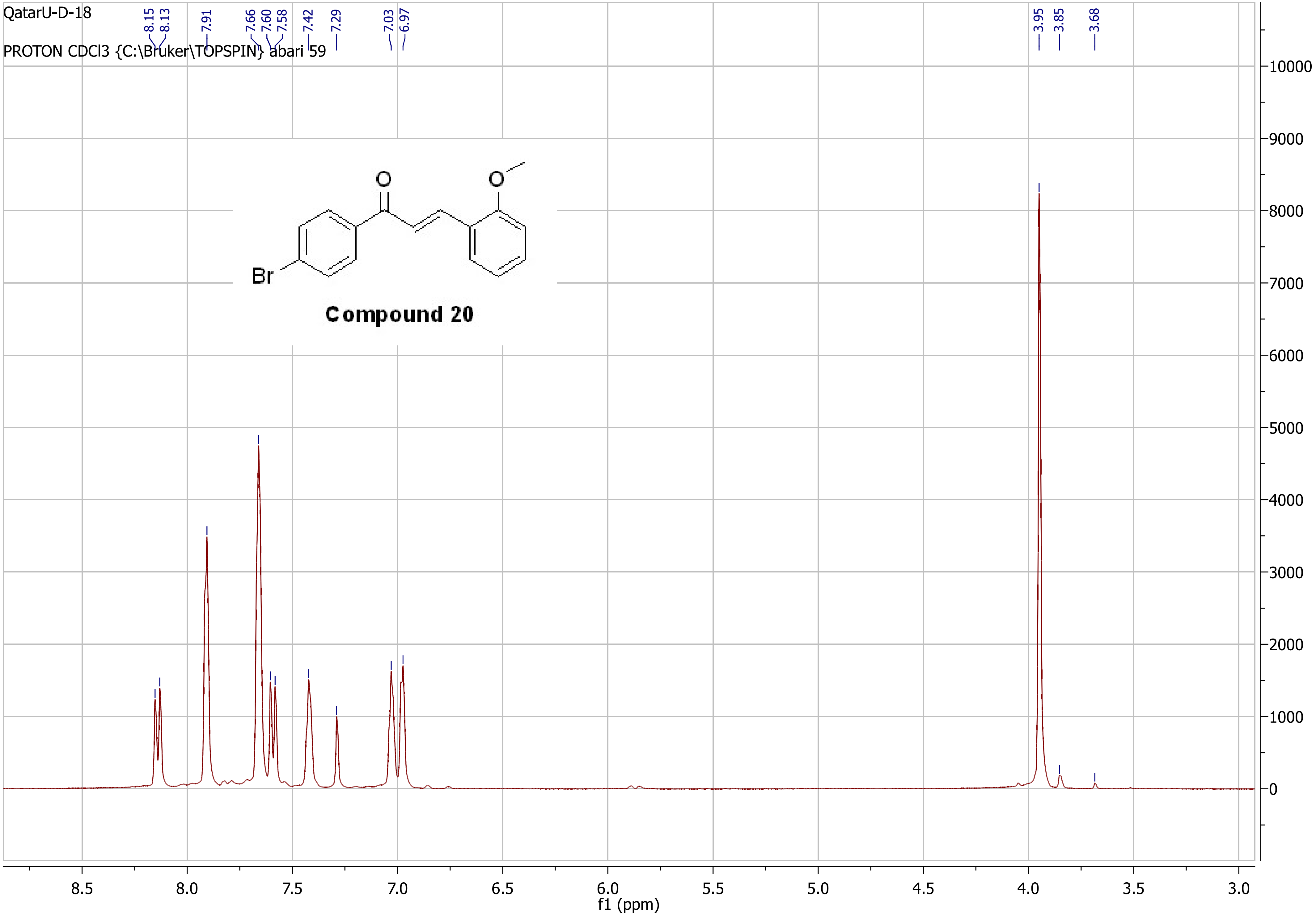
QatarU-D-18
PROTON CDCl3 {C:\Bruker\TOPSPIN} abari 59
8.15
8.13
7.91
7.66
7.60
7.58
7.42
7.29
7.03
6.97
3.95
3.85
3.68
O
O
Br
Compound 20
10000
9000
8000
7000
6000
5000
4000
3000
2000
1000
0
8.5
8.0
7.5
7.0
6.5
6.0
5.5
5.0
4.5
4.0
3.5
3.0
f1 (ppm)

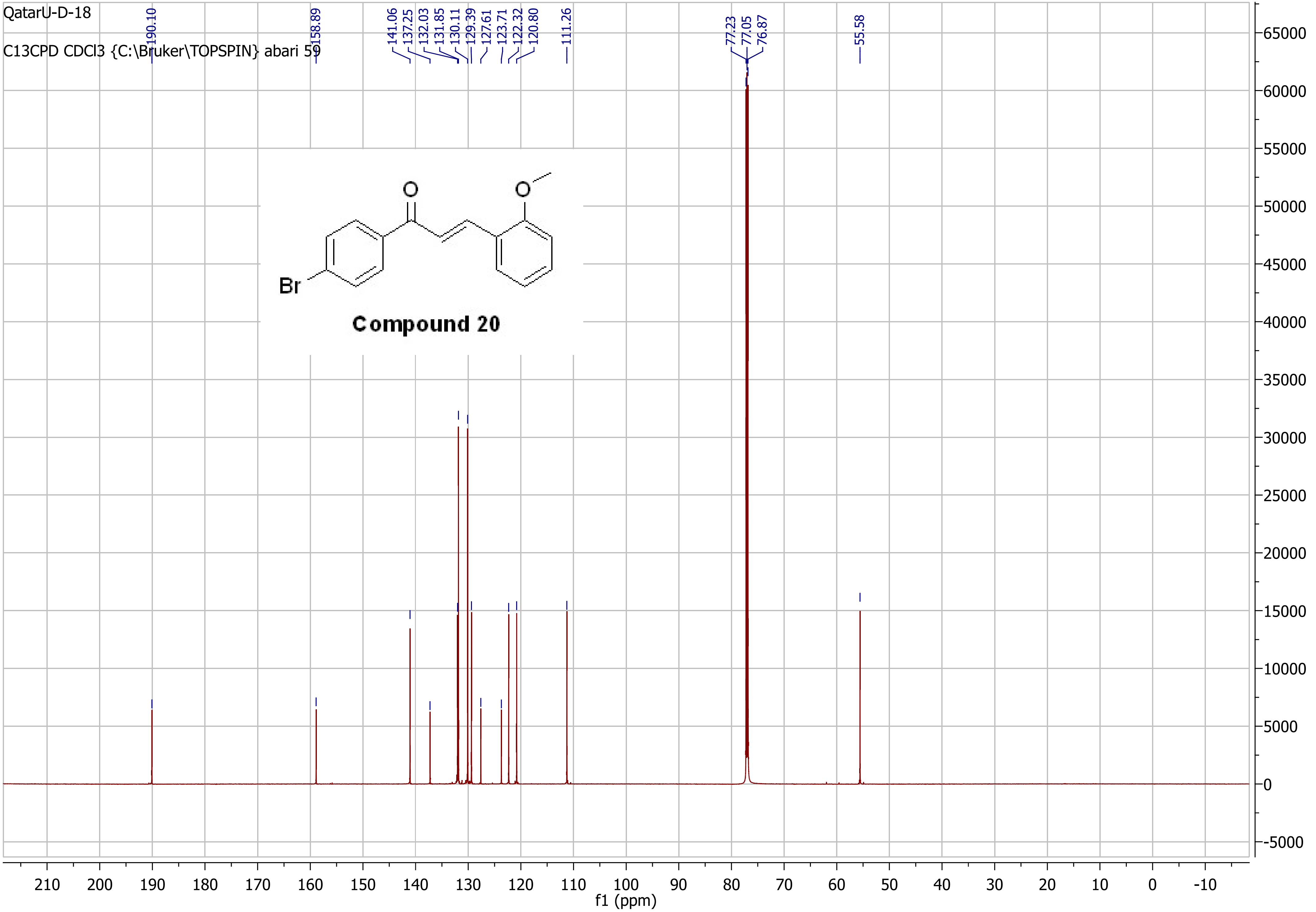
QatarU-D-18
C13CPD CDCl3 {C:\Bruker\TOPSPIN} abari 59
190.10
158.89
141.06
137.25
132.03
131.85
130.11
129.39
127.61
123.71
122.32
120.80
111.26
77.23
77.05
76.87
55.58
Br
O
Compound 20
f1 (ppm)

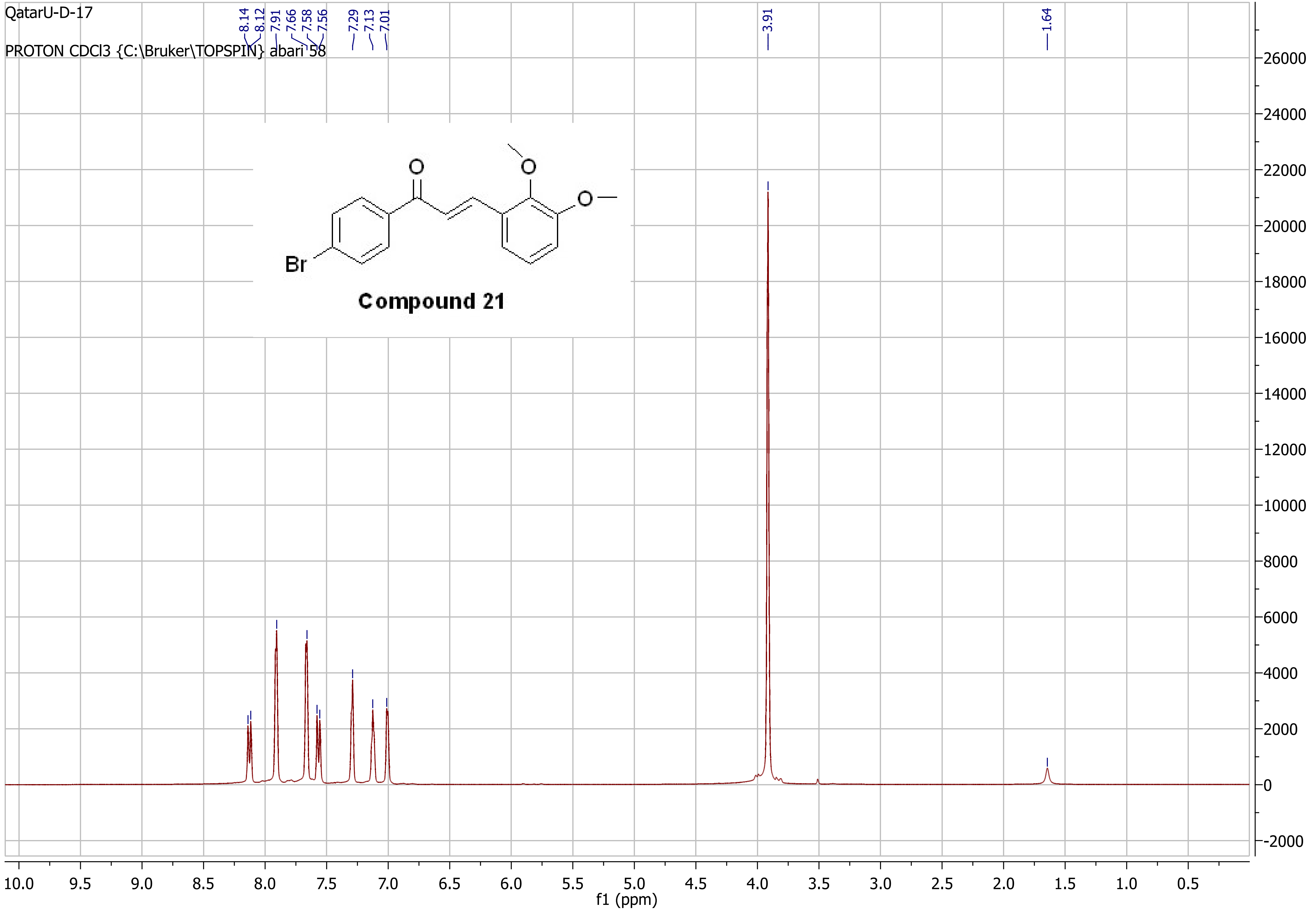
QatarU-D-17
PROTON CDCl3 {C:\Bruker\TOPSPIN} abari 58
8.14
8.12
7.91
7.66
7.58
7.56
7.29
7.13
7.01
3.91
1.64
O
O
O
Br
Compound 21
26000
24000
22000
20000
18000
16000
14000
12000
10000
8000
6000
4000
2000
0
-2000
10.0 9.5 9.0 8.5 8.0 7.5 7.0 6.5 6.0 5.5 5.0 4.5 4.0 3.5 3.0 2.5 2.0 1.5 1.0 0.5
f1 (ppm)

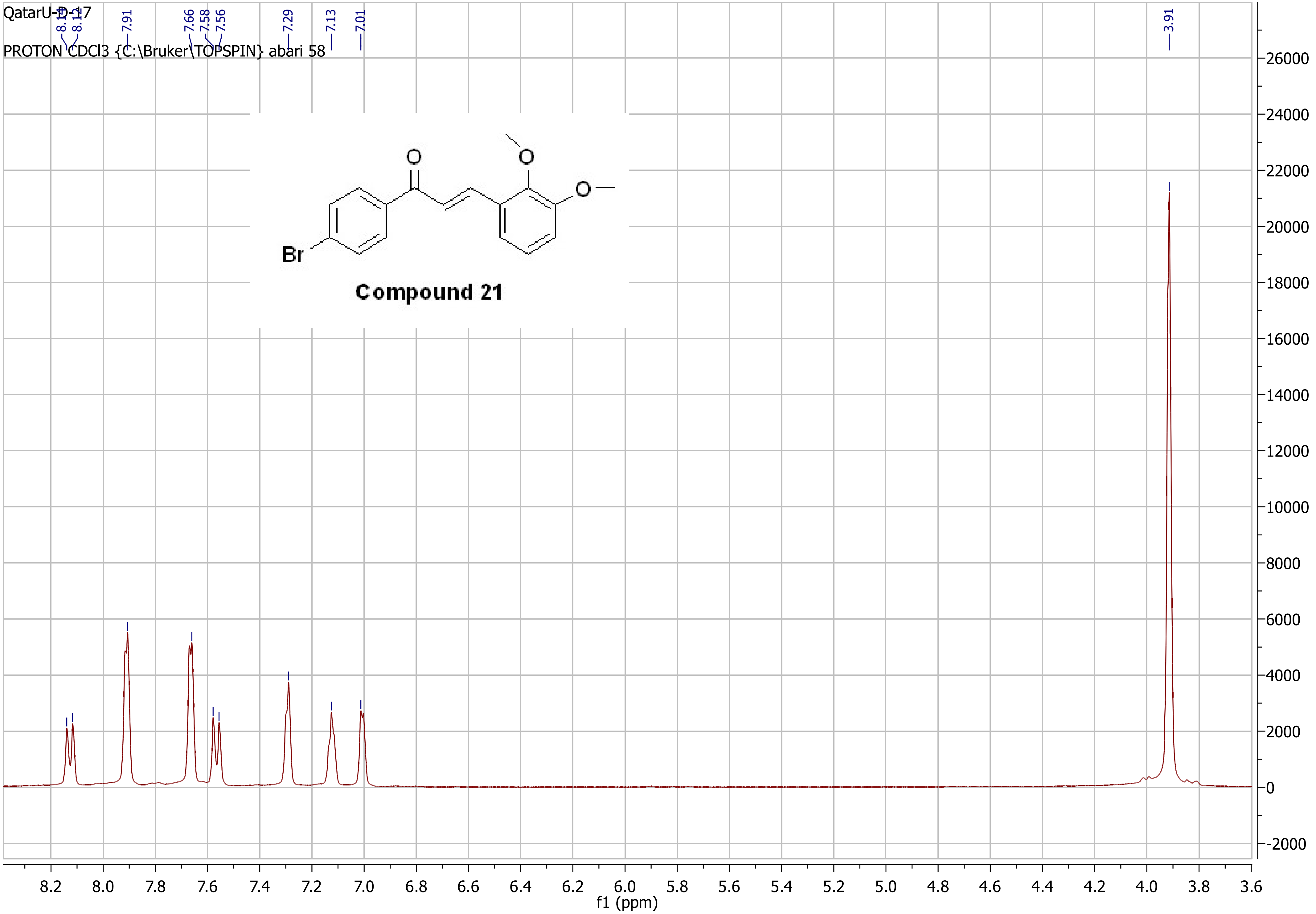
QatarU-D-17
PROTON CDCl3 {C:\Bruker\TOPSPIN} abari 58
8.15 8.11 7.91 7.66 7.58 7.56 7.29 7.13 7.01 3.91
O
O
O
Br
Compound 21
26000
24000
22000
20000
18000
16000
14000
12000
10000
8000
6000
4000
2000
0
-2000
8.2 8.0 7.8 7.6 7.4 7.2 7.0 6.8 6.6 6.4 6.2 6.0 5.8 5.6 5.4 5.2 5.0 4.8 4.6 4.4 4.2 4.0 3.8 3.6
f1 (ppm)

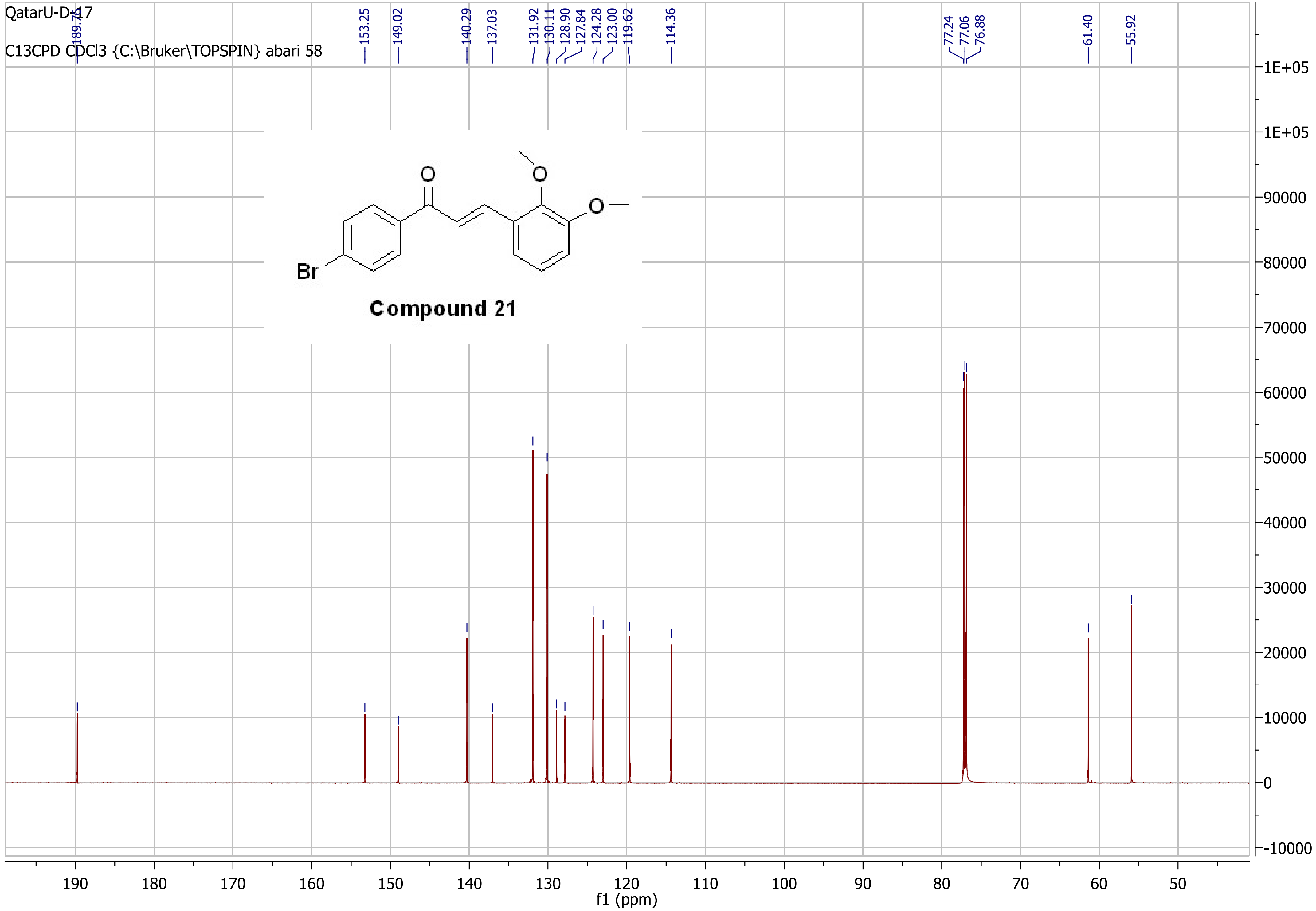
QatarU-D-17
C13CPD CDCl3 {C:\Bruker\TOPSPIN} abari 58
189.76
153.25
149.02
140.29
137.03
131.92
130.11
128.90
127.84
124.28
123.00
119.62
114.36
77.24
77.06
76.88
61.40
55.92
Br
O
O
O
Compound 21
1E+05
1E+05
90000
80000
70000
60000
50000
40000
30000
20000
10000
0
-10000
190
180
170
160
150
140
130
120
110
100
90
80
70
60
50
f1 (ppm)

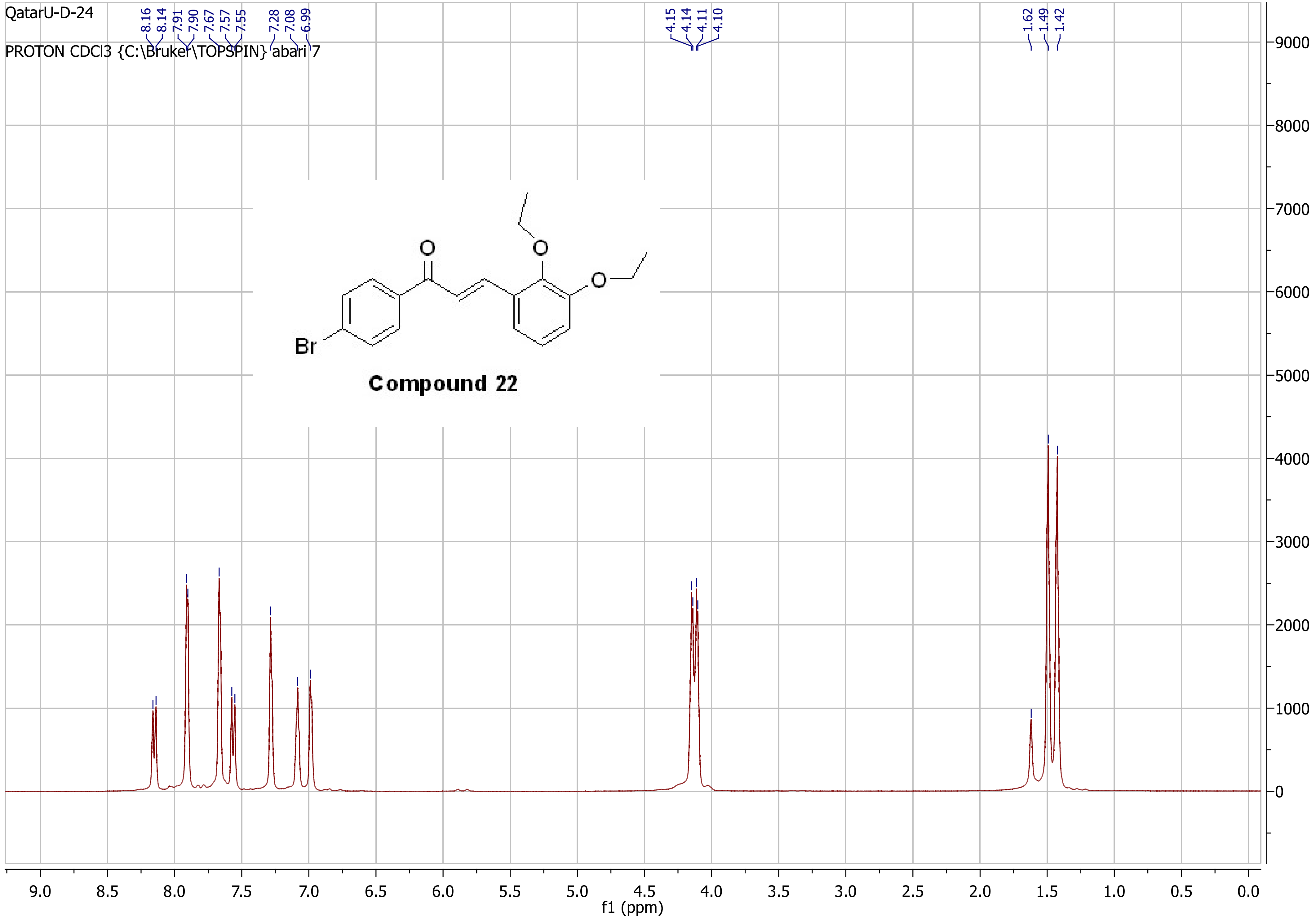
QatarU-D-24
PROTON CDCl3 {C:\Bruker\TOPSPIN} abari 7
8.16 8.14 7.91 7.90 7.67 7.57 7.55 7.28 7.08 6.99
4.15 4.14 4.11 4.10
1.62 1.49 1.42
O
O
O
Br
Compound 22
f1 (ppm)

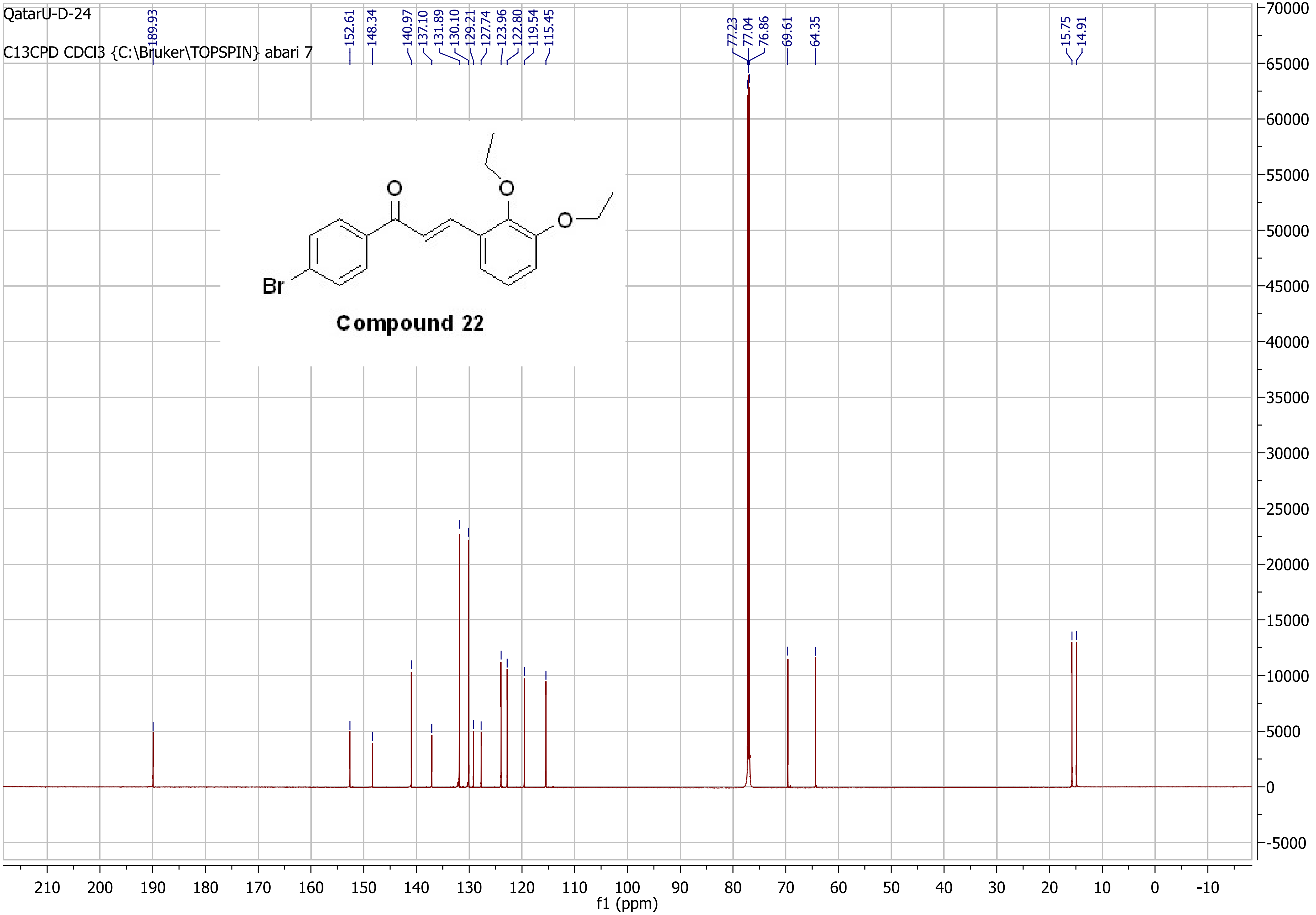
QatarU-D-24
C13CPD CDCl3 {C:\Bruker\TOPSPIN} abari 7
189.93
152.61
148.34
140.97
137.10
131.89
130.10
129.21
127.74
123.96
122.80
119.54
115.45
77.23
77.04
76.86
69.61
64.35
15.75
14.91
O
O
O
Br
Compound 22
f1 (ppm)
