# Supplementary material for: SAR and molecular mechanism studies of monoamine oxidase inhibition by selected chalcone analogs
Source: J Enzyme Inhib Med Chem. 2019 Mar 27;34(1):863–76. doi: 10.1080/14756366.2019.1593158 (PMC6442233; doi:10.1080/14756366.2019.1593158)

Compound 1

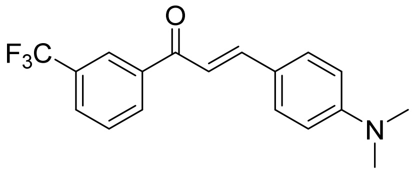

Molecular Weight: 319.33
m/z: 319.12 (100.0%), 320.12 (19.5%), 321.13 (1.8%)

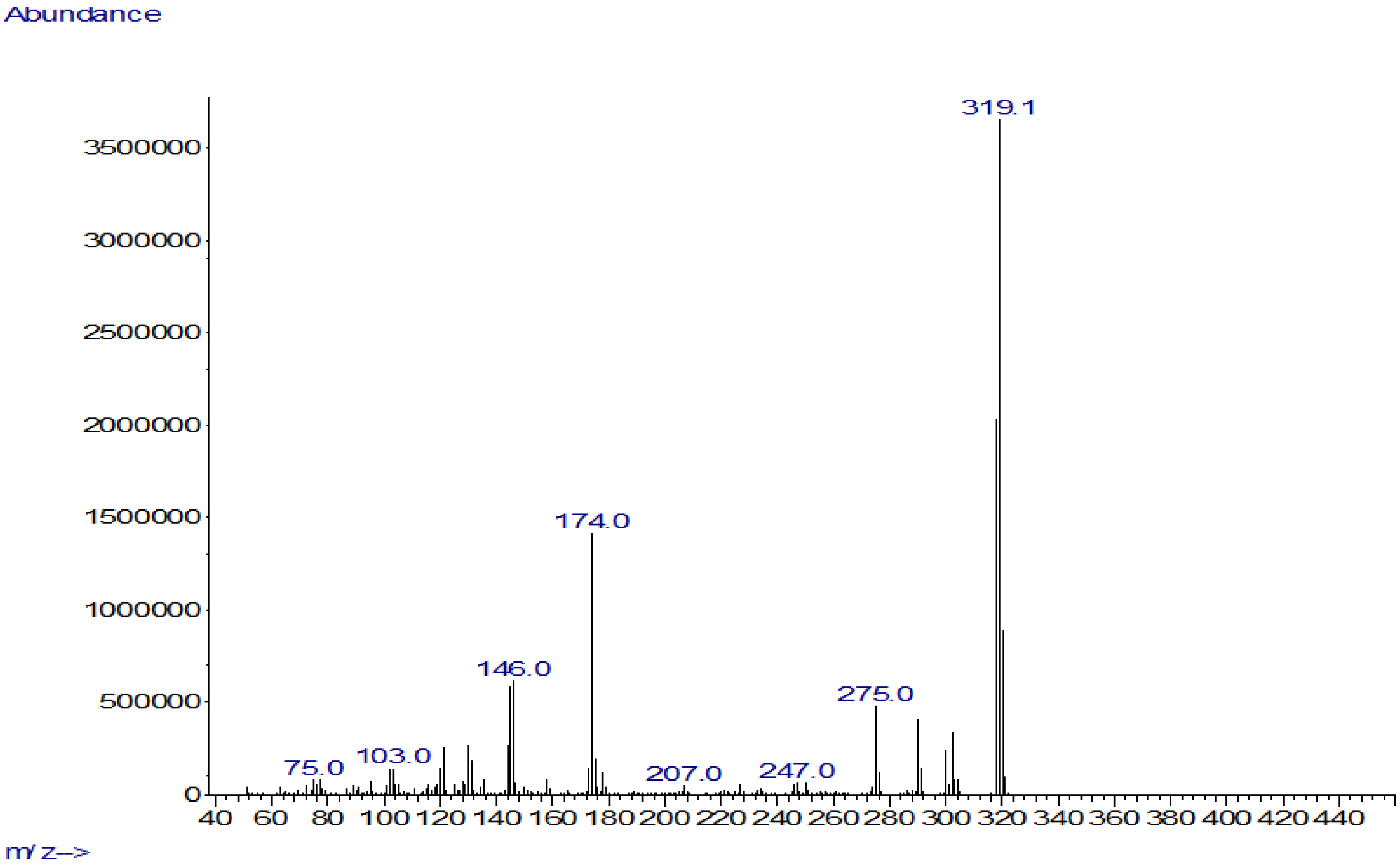

Compound 2

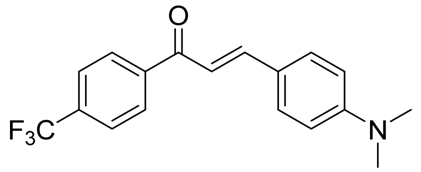

Molecular Weight: 319.33
m/z: 319.12 (100.0%), 320.12 (19.5%), 321.13 (1.8%)

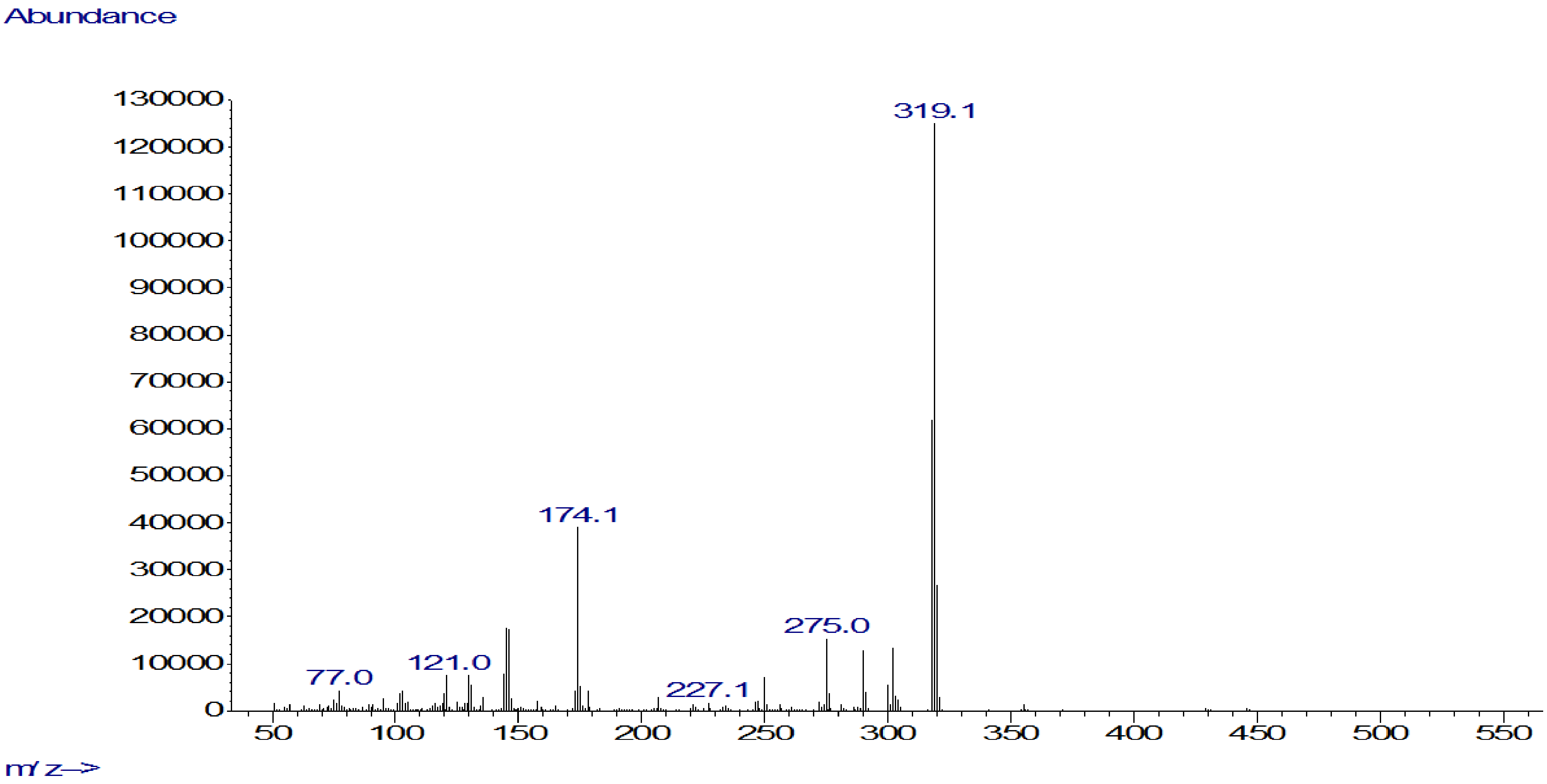

Compound 3

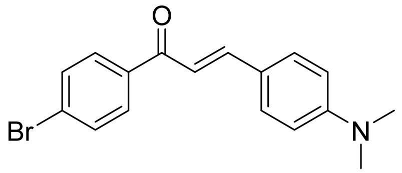

Molecular Weight: 330.23

m/z: 329.04 (100.0%), 331.04 (97.3%), 332.04 (17.9%), 330.04 (16.2%), 330.04 (2.2%), 333.05 (1.2%), 331.05 (1.1%)

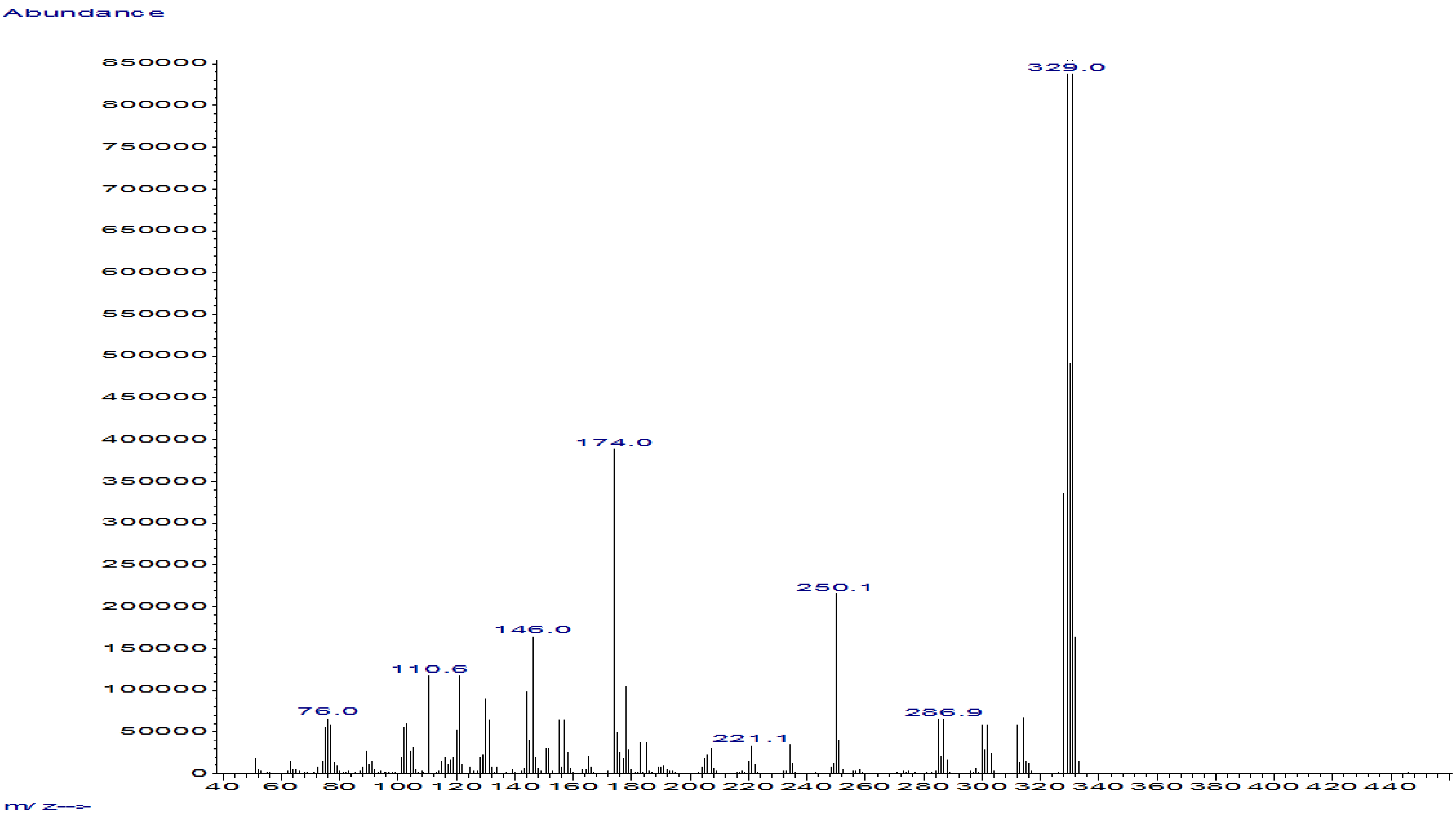

Compound 4

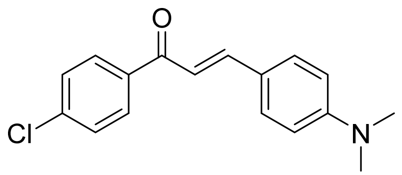

Molecular Weight: 285.77

m/z: 285.09 (100.0%), 287.09 (32.0%), 286.10 (18.4%), 288.09 (5.9%), 287.10 (1.6%)

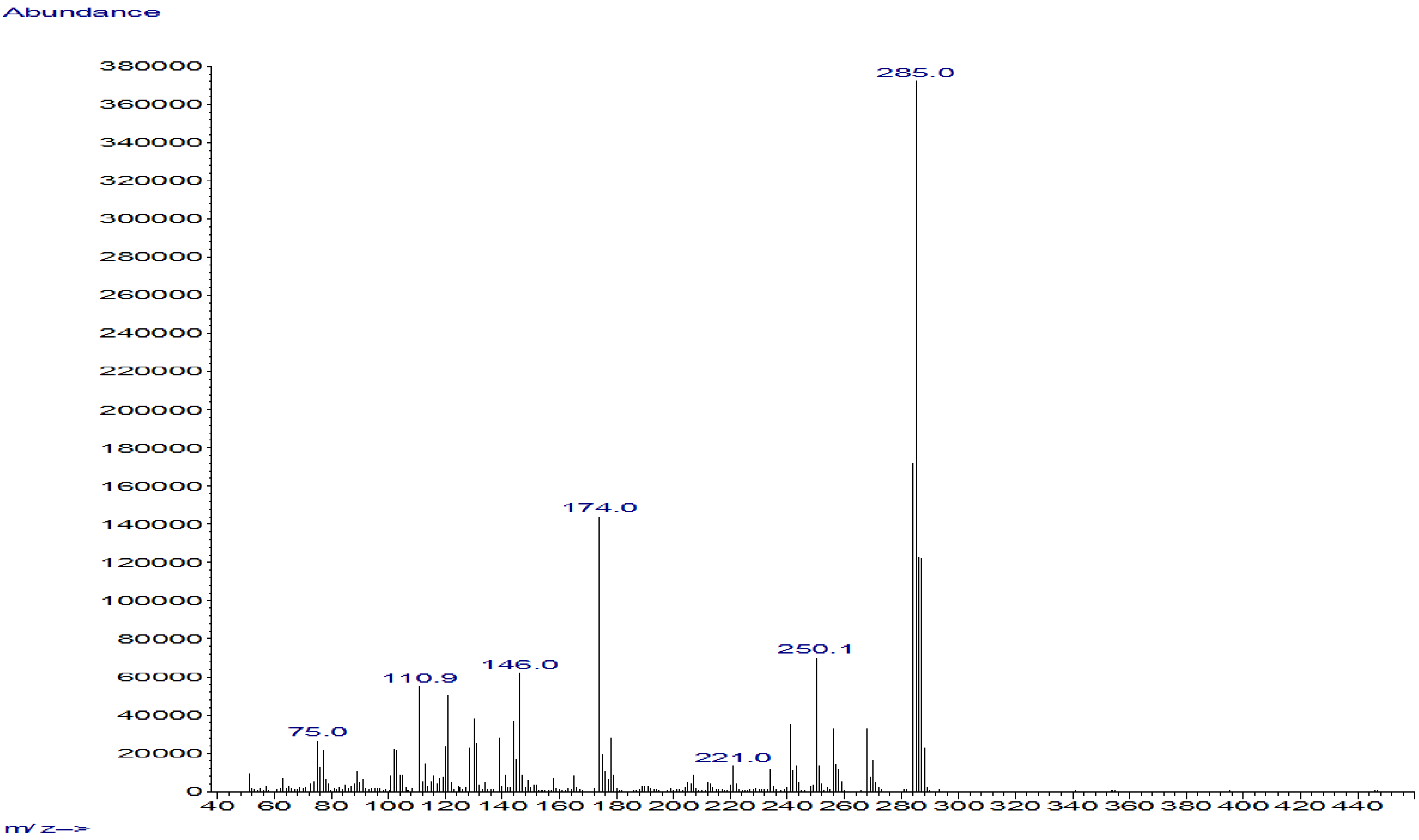

Compound 5

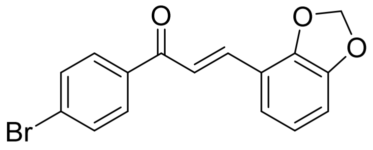

Molecular Weight: 331.17
m/z: 329.99 (100.0%), 331.99 (97.3%), 332.99 (16.8%), 330.99 (16.2%), 333.99 (1.2%), 330.99 (1.1%), 332.00 (1.1%)

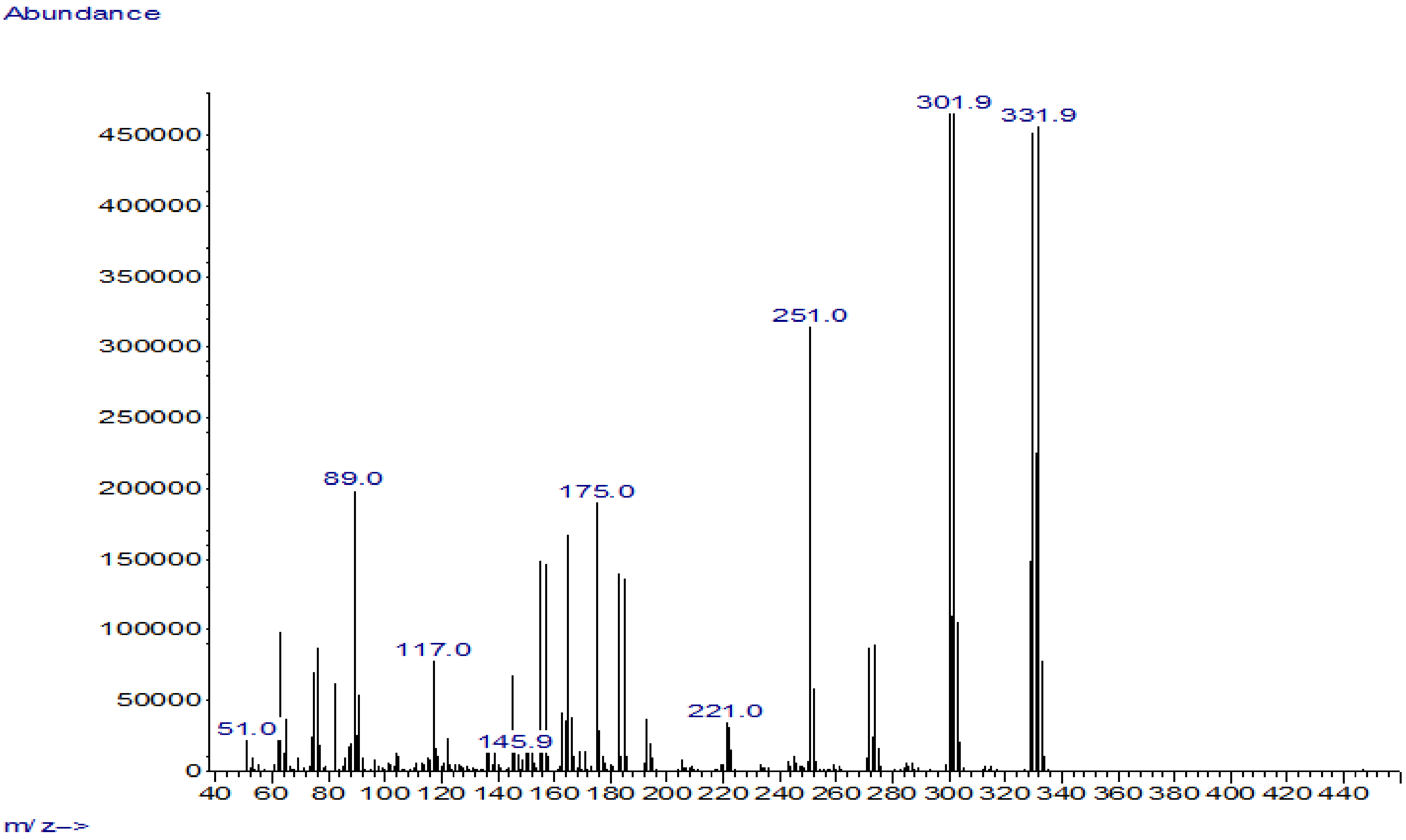

Compound 6

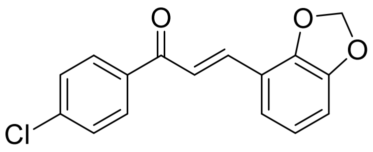

Molecular Weight: 286.71

m/z: 286.04 (100.0%), 288.04 (32.0%), 287.04 (17.3%), 289.04 (5.5%), 288.05 (1.4%)

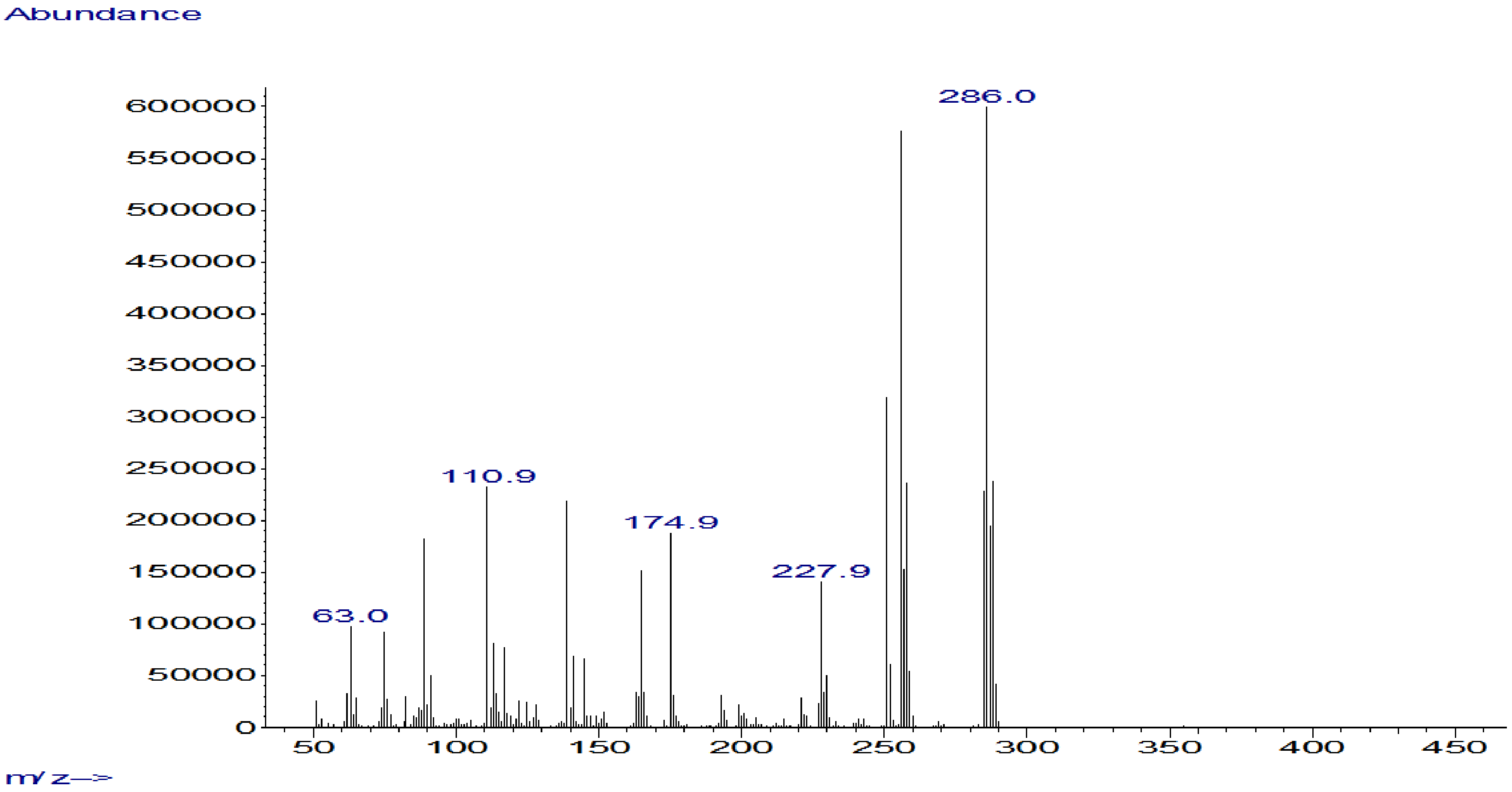

Compound 7

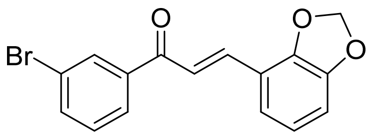

Molecular Weight: 331.17
m/z: 329.99 (100.0%), 331.99 (97.3%), 332.99 (16.8%), 330.99 (16.2%), 333.99 (1.2%), 330.99 (1.1%), 332.00 (1.1%)

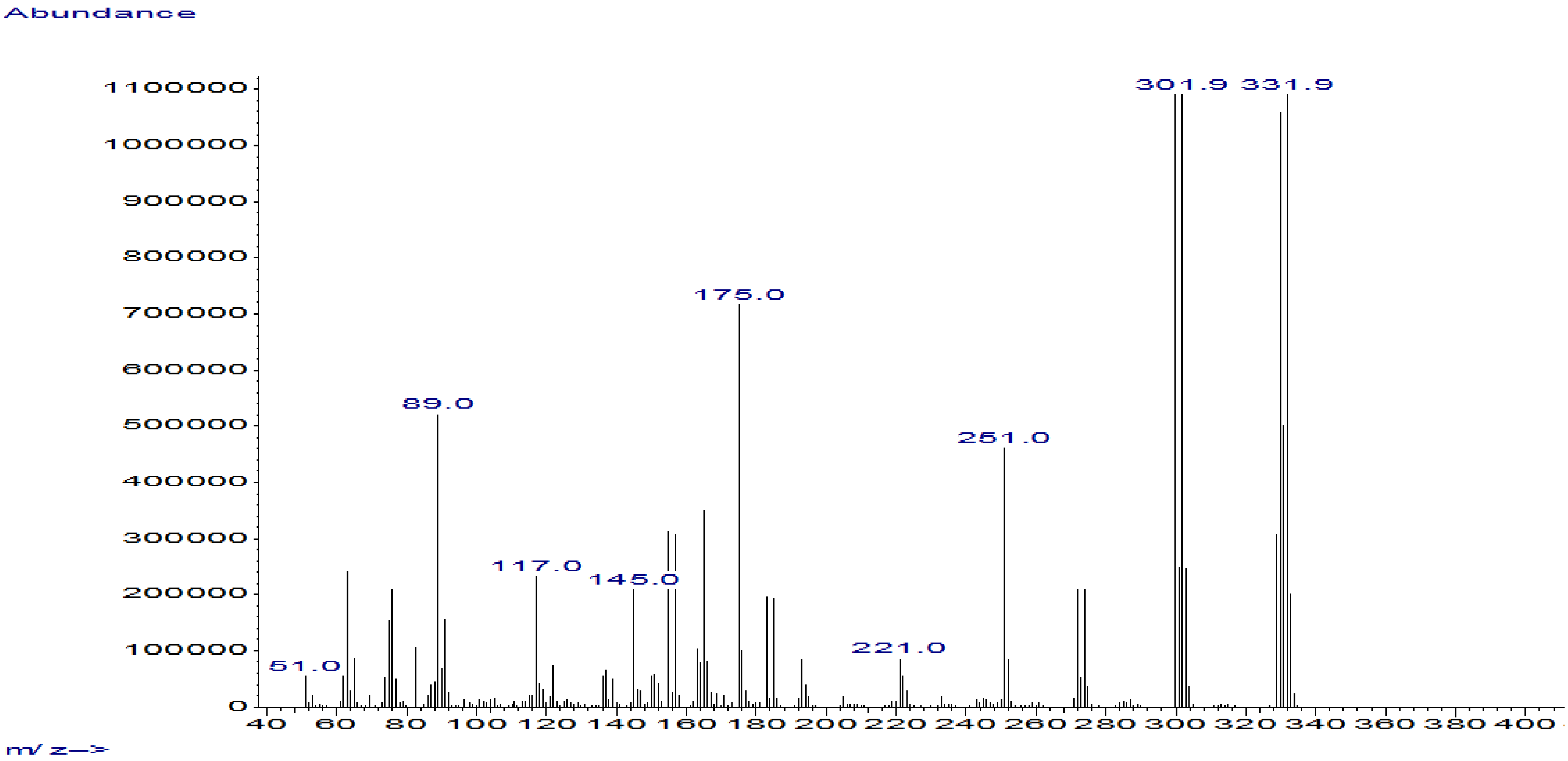

Compound 8

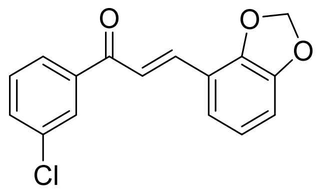

Molecular Weight: 286.71
m/z: 286.04 (100.0%), 288.04 (32.0%), 287.04 (17.3%), 289.04 (5.5%), 288.05 (1.4%)

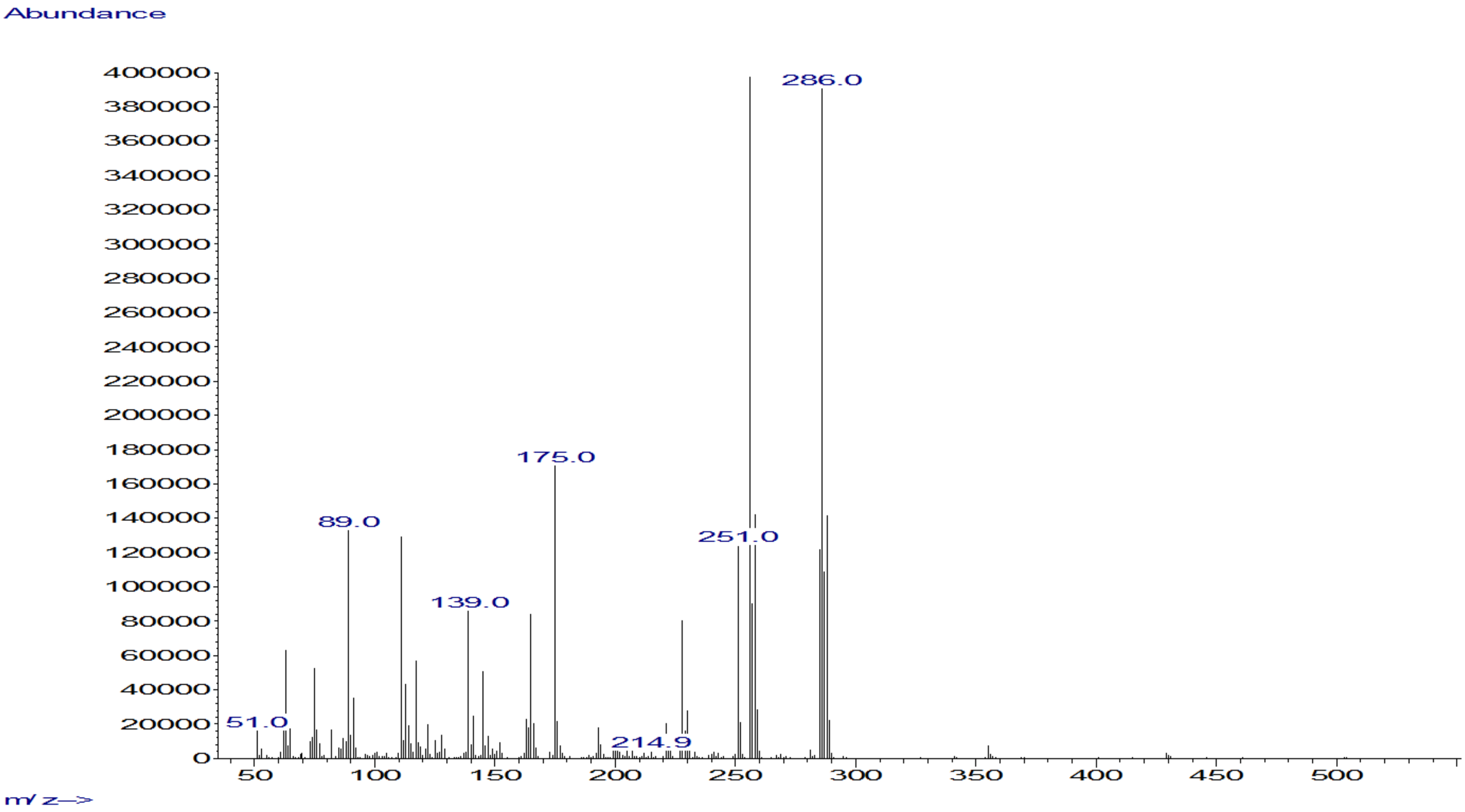

Compound 9

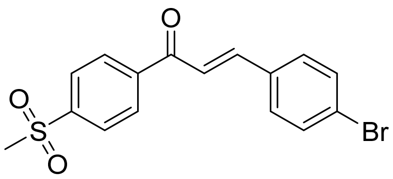

Molecular Weight: 365.24
m/z: 363.98 (100.0%), 365.97 (97.3%), 366.98 (16.8%), 364.98 (16.2%), 365.97 (4.5%), 367.97 (4.4%), 367.98 (1.2%), 364.98 (1.1%), 365.98 (1.1%)

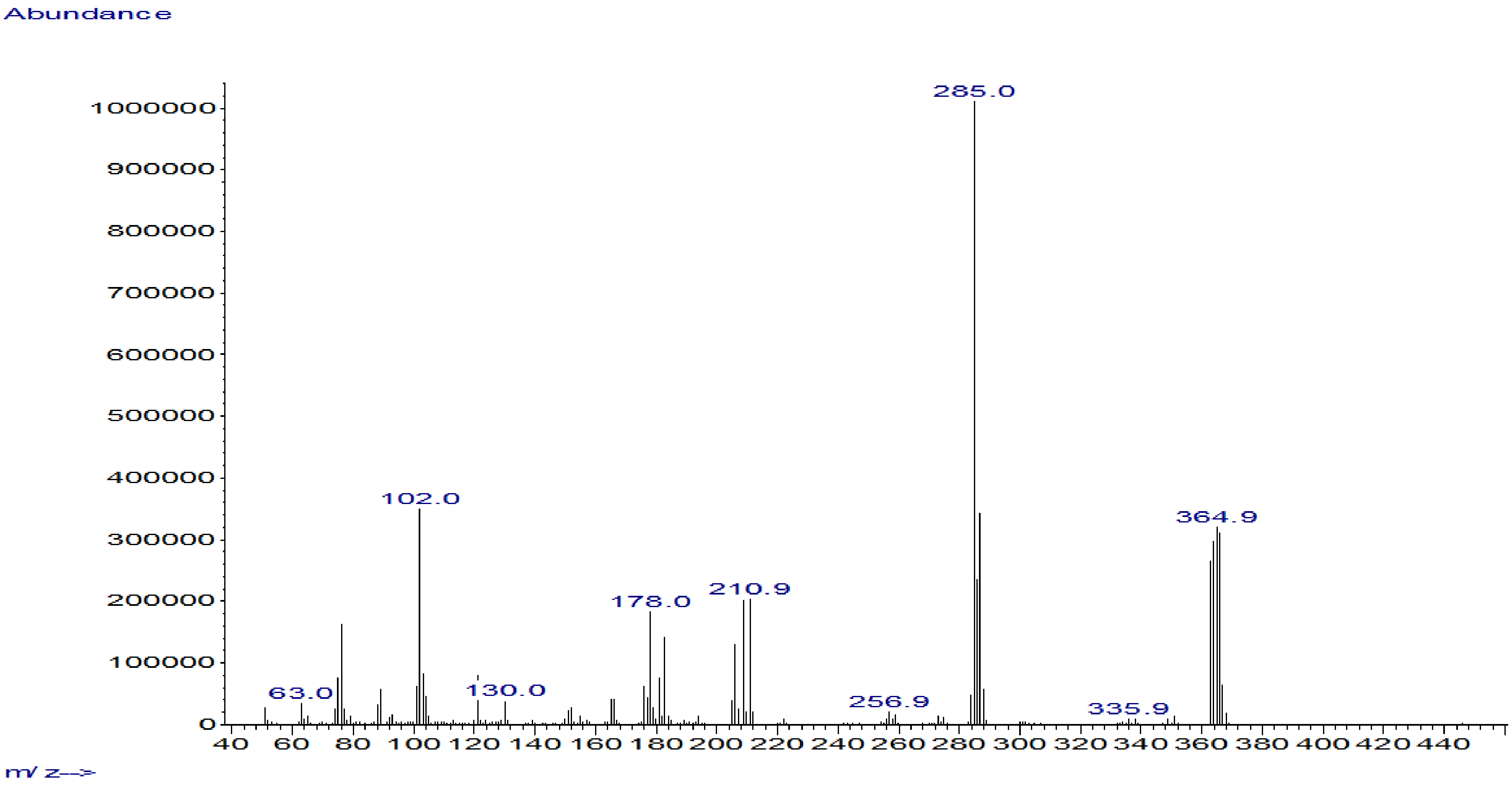

Compound 10

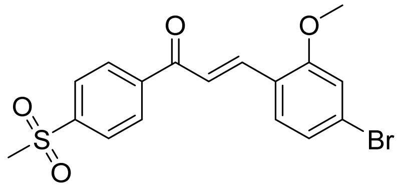

Molecular Weight: 395.27
m/z: 393.99 (100.0%), 395.99 (97.3%), 396.99 (17.9%), 394.99 (16.2%), 395.98 (4.5%), 397.98 (4.4%), 394.99 (2.2%), 397.99 (1.2%), 395.99 (1.1%)

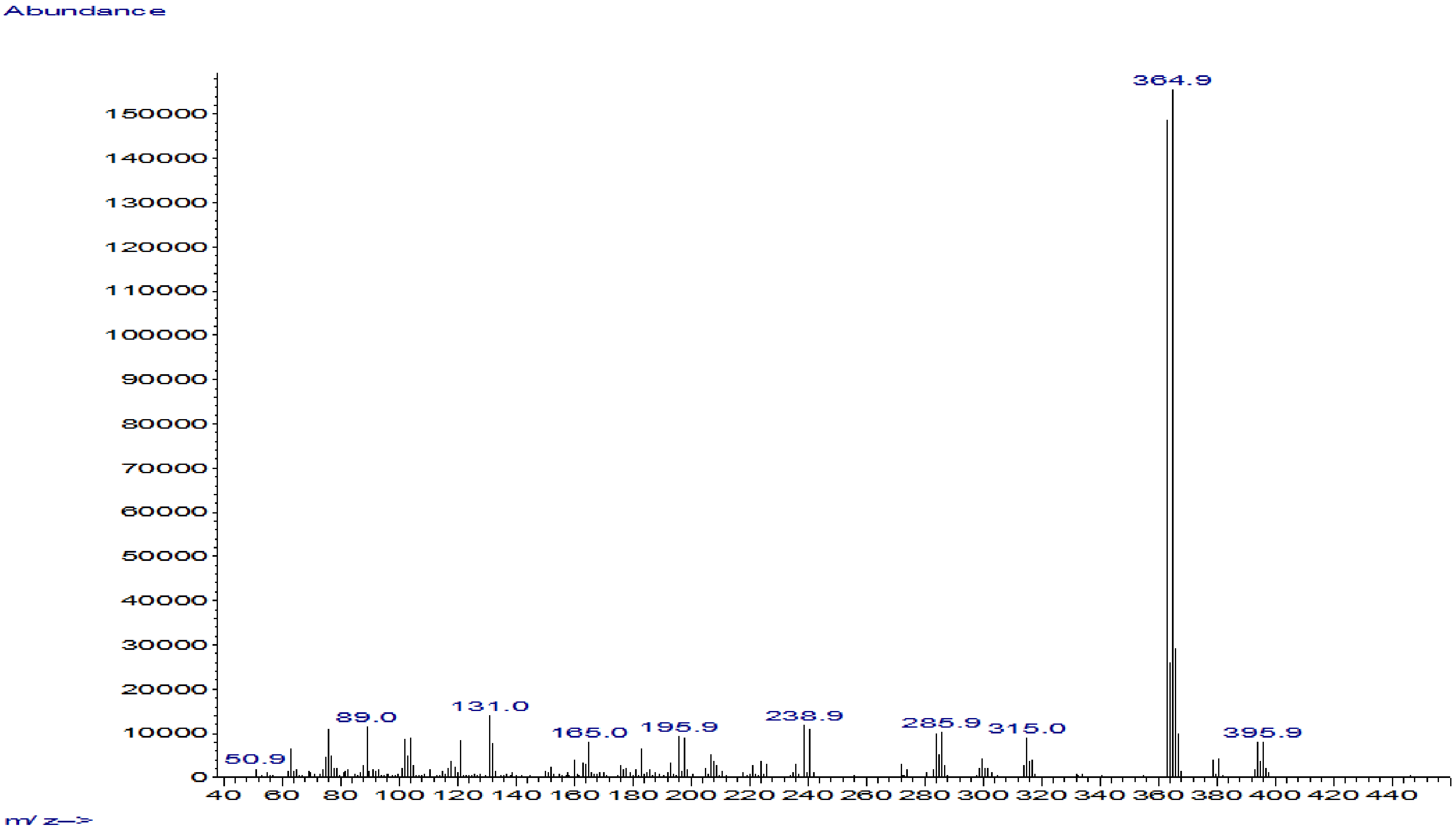

Compound 11

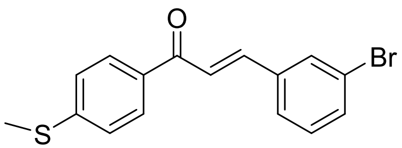

Molecular Weight: 333.24
m/z: 331.99 (100.0%), 333.99 (97.3%), 334.99 (16.8%), 332.99 (16.2%), 333.98 (4.5%), 335.98 (4.4%), 333.99 (1.4%), 335.99 (1.2%), 332.99 (1.1%)

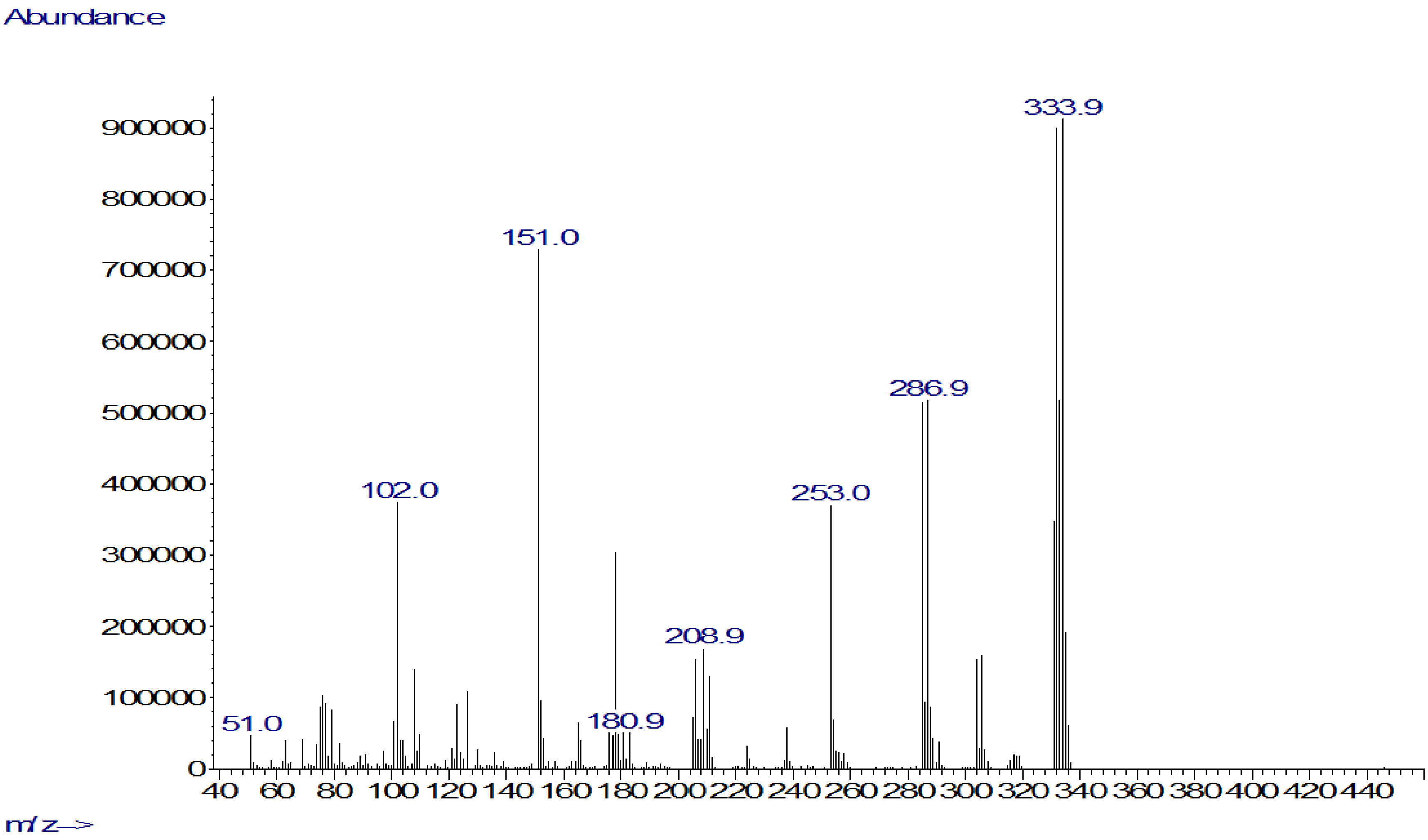

Compound 12

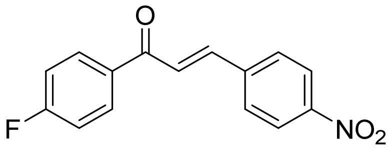

Molecular Weight: 271.25
m/z: 271.06 (100.0%), 272.07 (16.2%), 273.07 (1.2%)

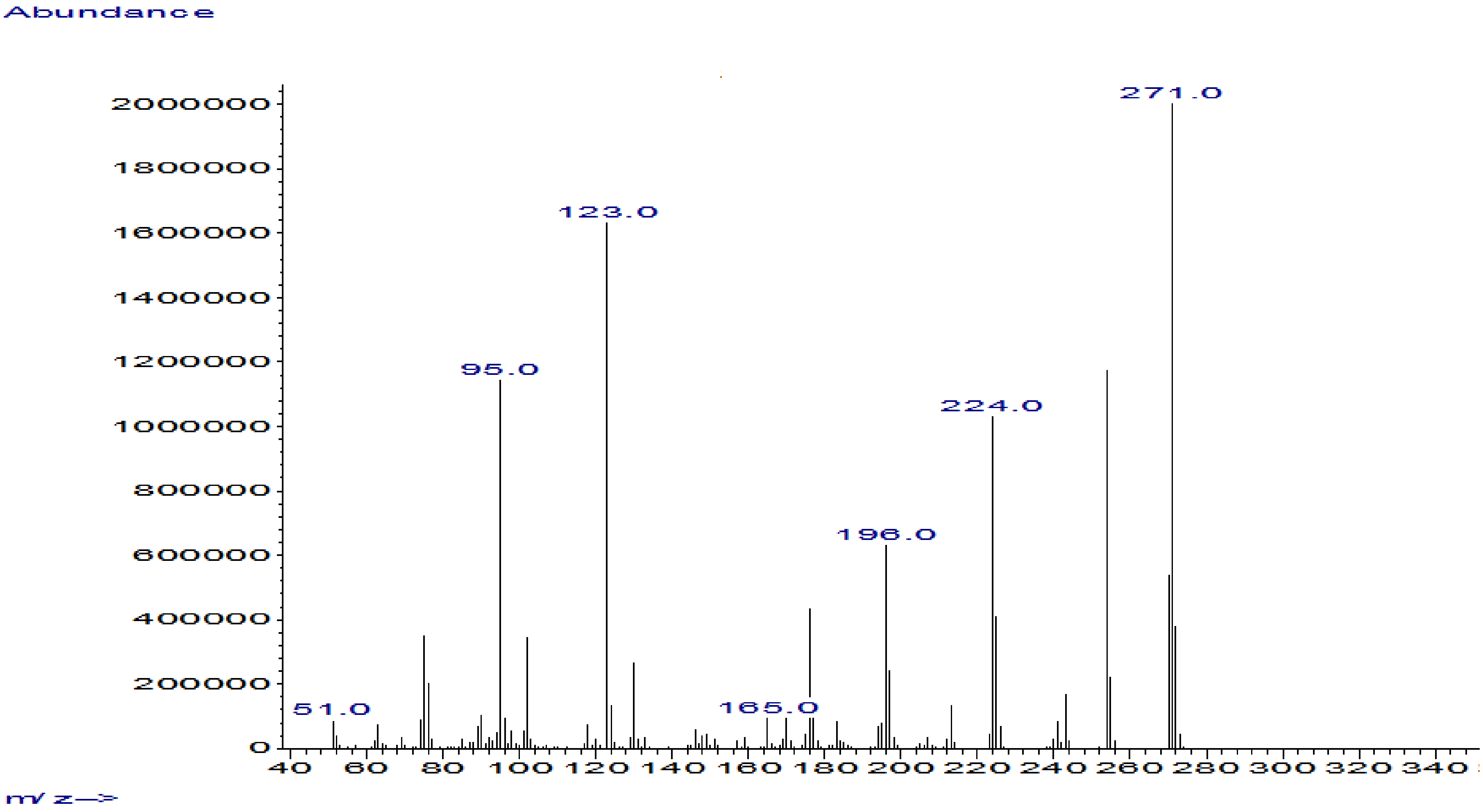

Compound 13

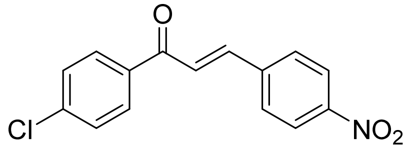

Molecular Weight: 287.70
m/z: 287.03 (100.0%), 289.03 (32.0%), 288.04 (16.2%), 290.04 (5.2%), 289.04 (1.2%)

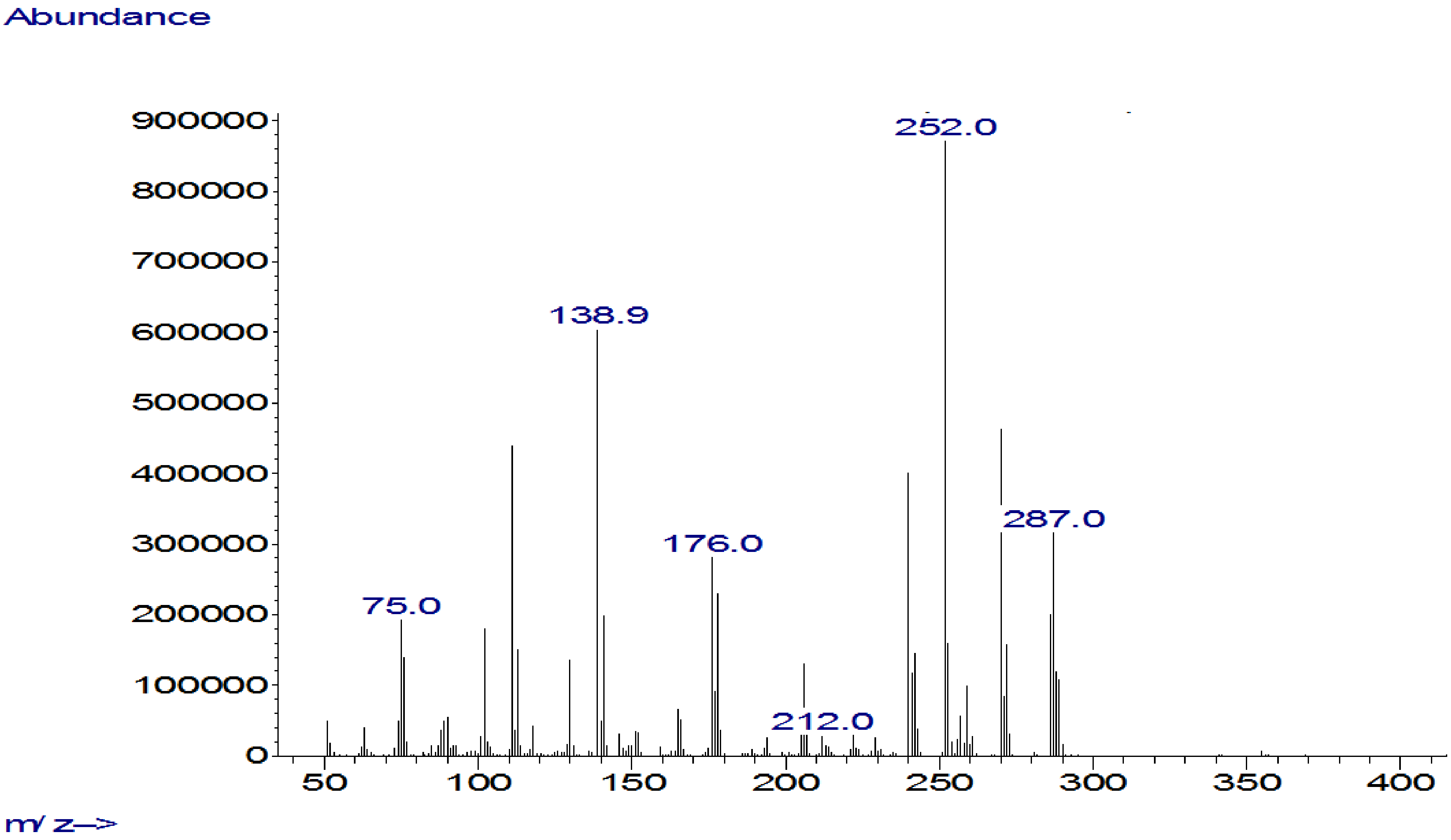

Compound 14

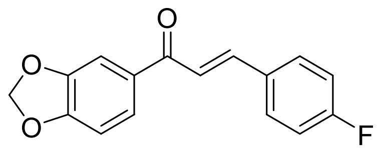

Molecular Weight: 270.26
m/z: 270.07 (100.0%), 271.07 (17.3%), 272.08 (1.4%)

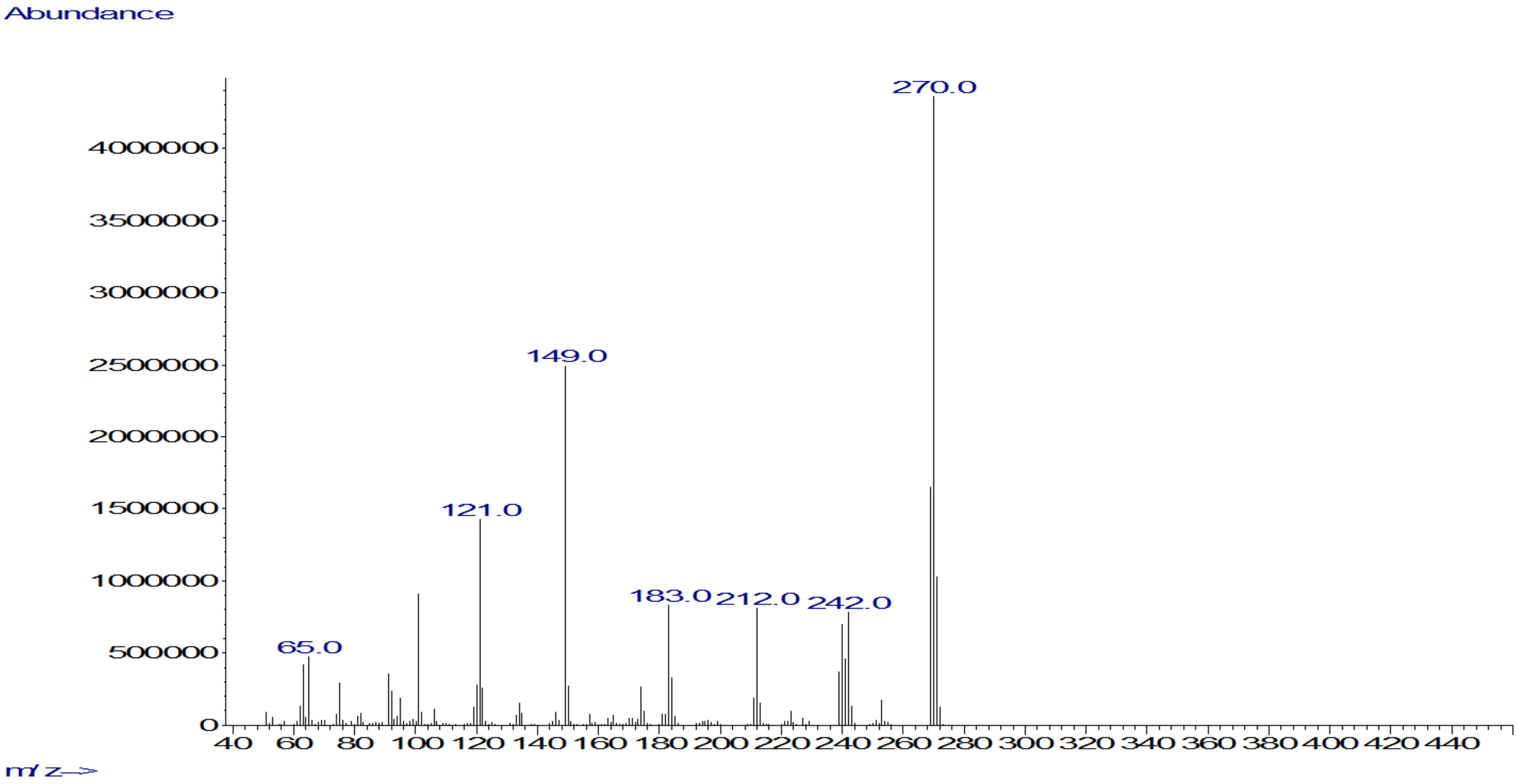

Compound 15

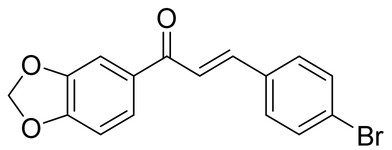

Molecular Weight: 331.17
m/z: 329.99 (100.0%), 331.99 (97.3%), 332.99 (16.8%), 330.99 (16.2%), 333.99 (1.2%), 330.99 (1.1%), 332.00 (1.1%)

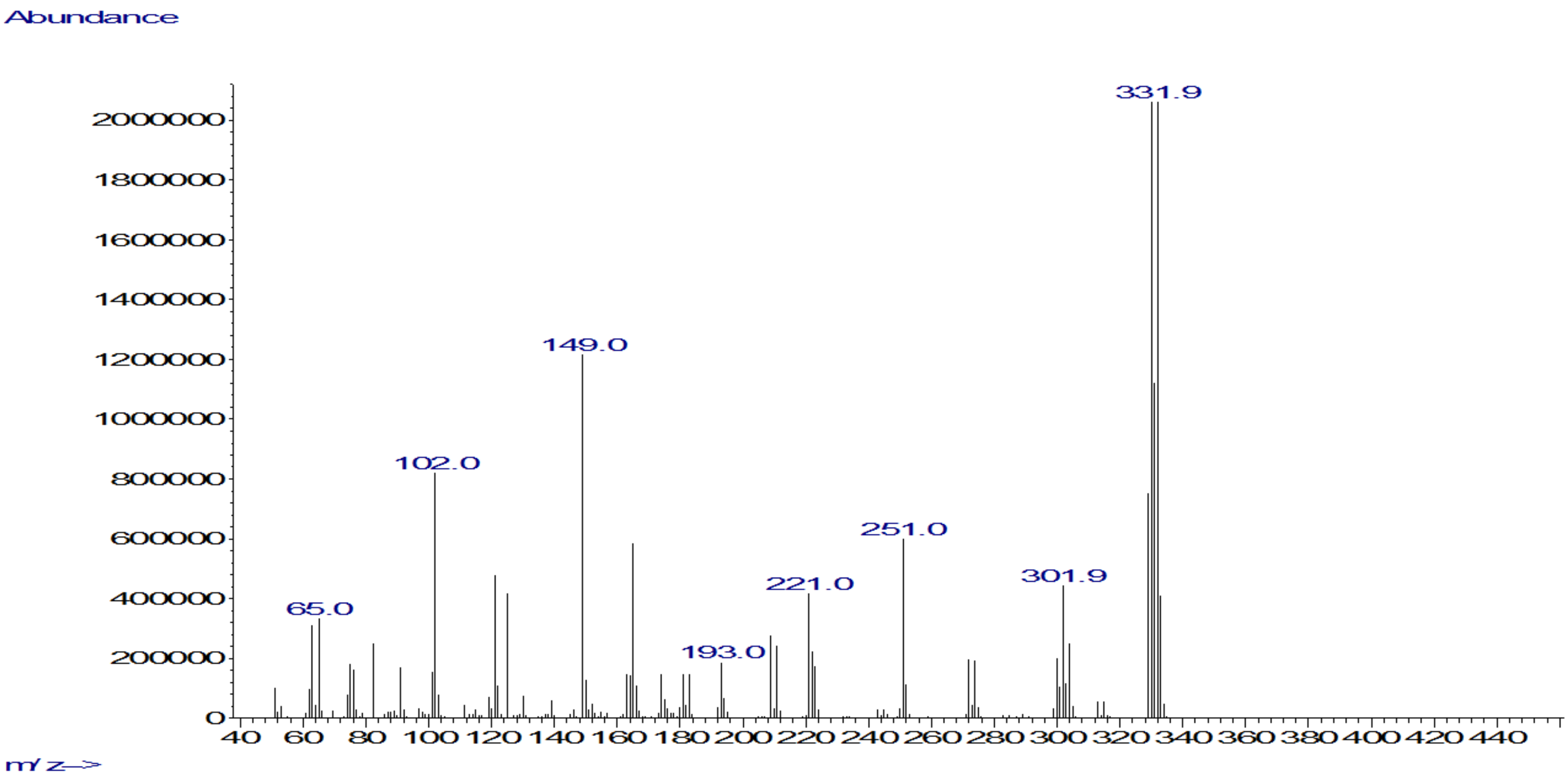

Compound 16

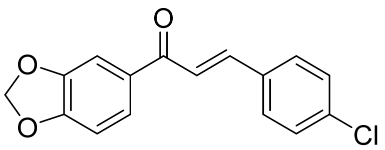

Molecular Weight: 286.71

m/z: 286.04 (100.0%), 288.04 (32.0%), 287.04 (17.3%), 289.04 (5.5%), 288.05 (1.4%)

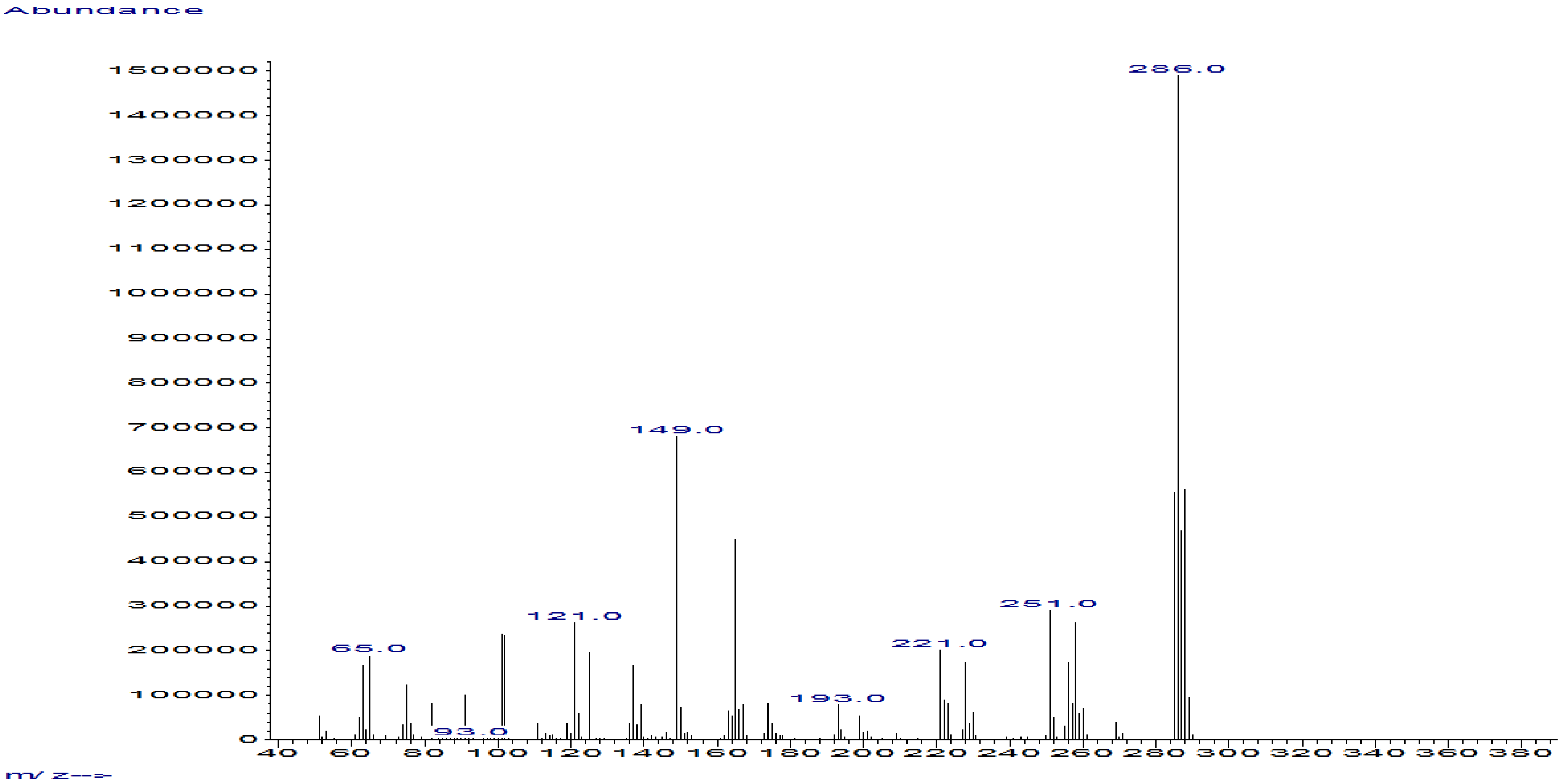

Compound 17

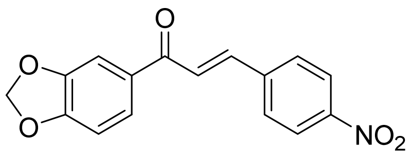

Molecular Weight: 297.27
m/z: 297.06 (100.0%), 298.07 (17.3%), 299.07 (1.4%), 299.07 (1.0%)

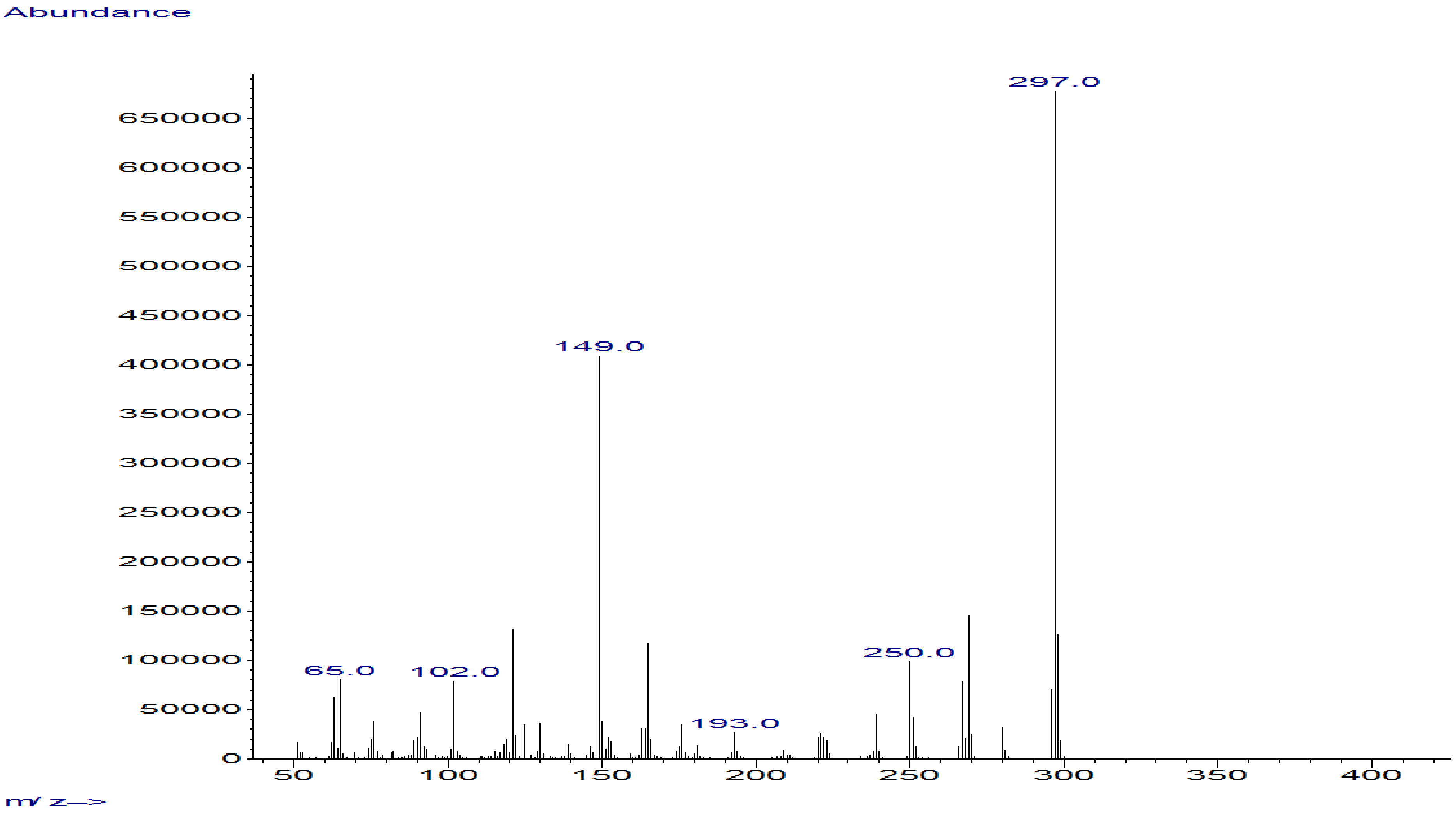

Compound 18

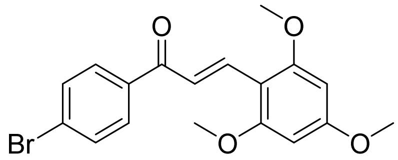

Molecular Weight: 377.23
m/z: 376.03 (100.0%), 378.03 (97.3%), 379.03 (18.9%), 377.03 (16.2%), 377.03 (3.2%), 380.04 (1.2%), 378.04 (1.1%)

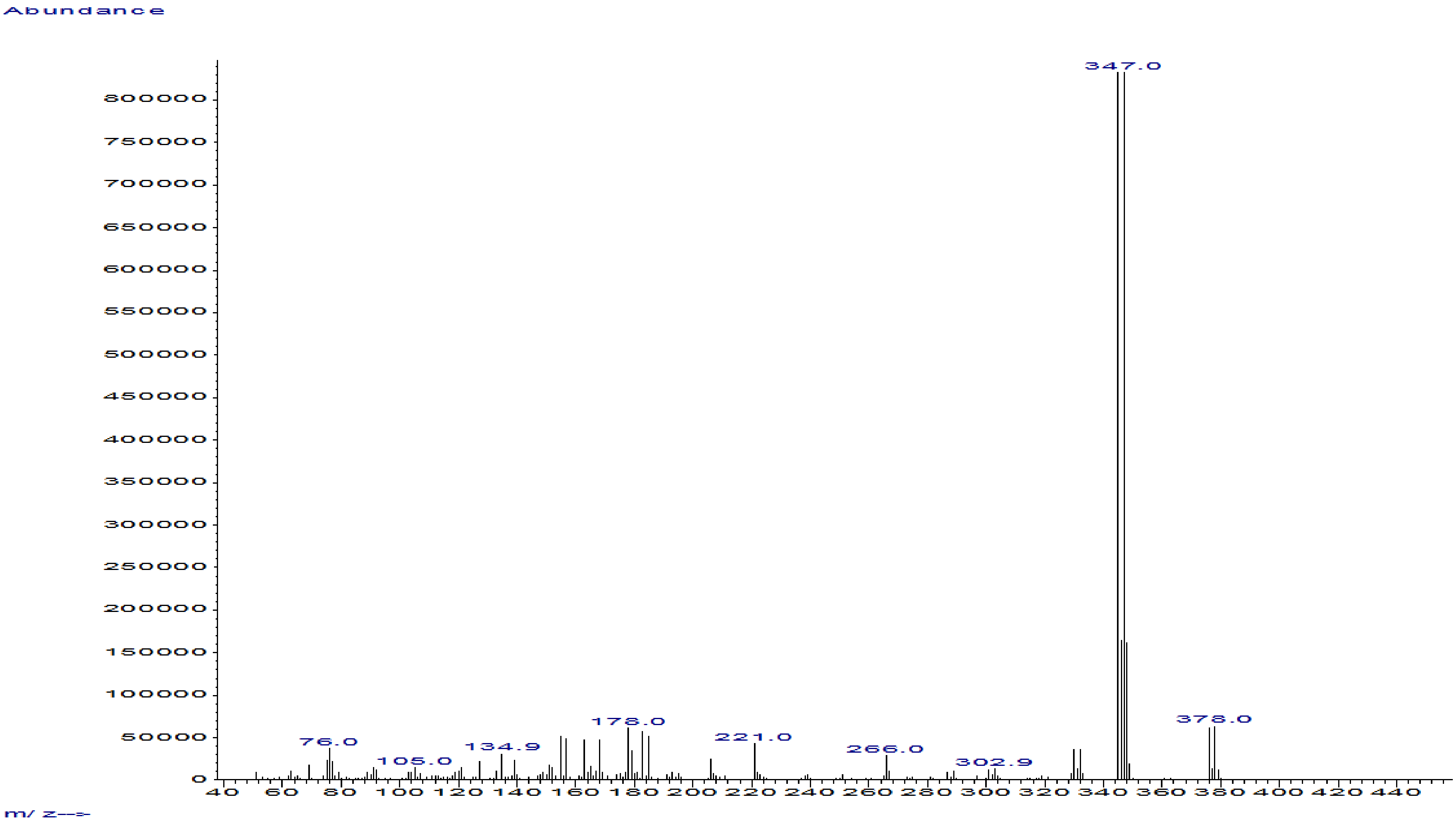

Compound 19

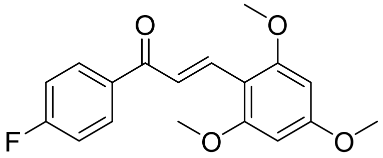

Molecular Weight: 316.33
m/z: 316.11 (100.0%), 317.11 (19.5%), 318.12 (1.8%)

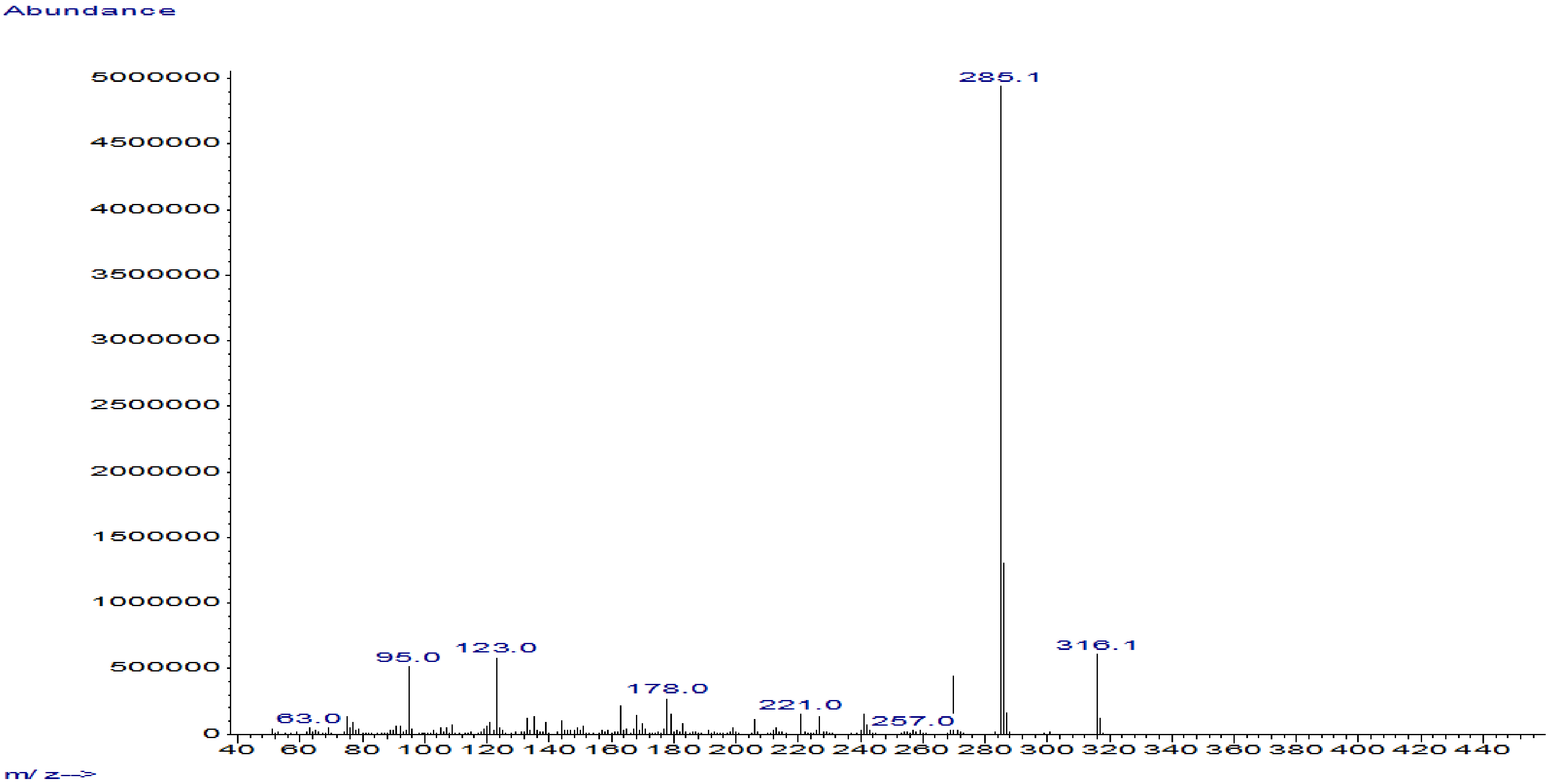

Compound 20

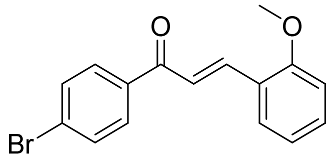

Molecular Weight: 317.18
m/z: 316.01 (100.0%), 318.01 (97.3%), 319.01 (16.8%), 317.01 (16.2%), 320.01 (1.2%), 317.01 (1.1%), 318.02 (1.1%)

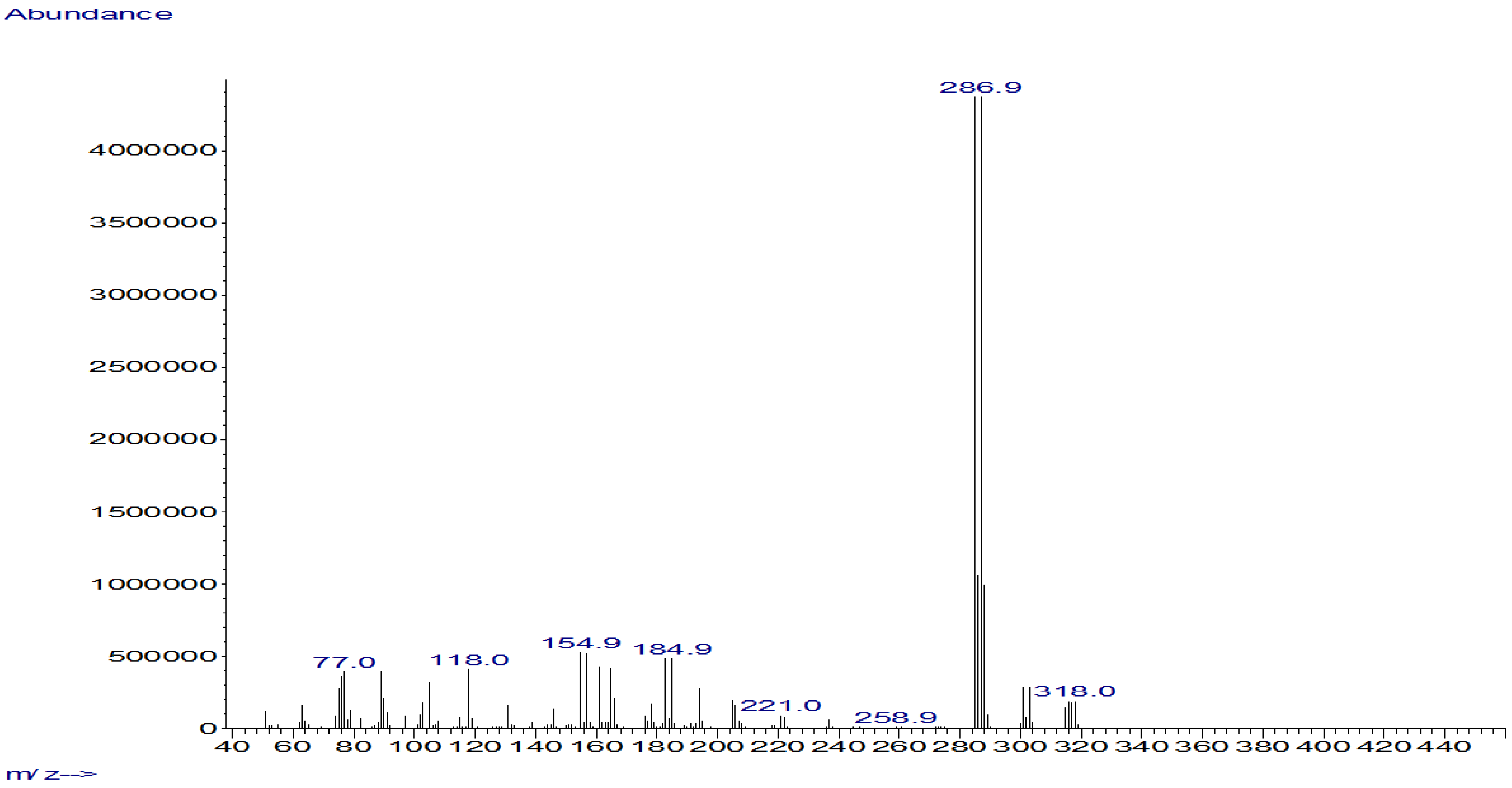

Compound 21

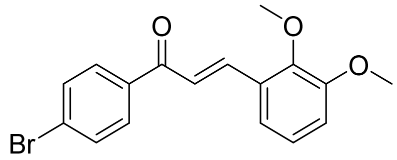

Molecular Weight: 347.21
m/z: 346.02 (100.0%), 348.02 (97.3%), 349.02 (17.9%), 347.02 (16.2%), 347.02 (2.2%), 350.03 (1.2%), 348.03 (1.1%)

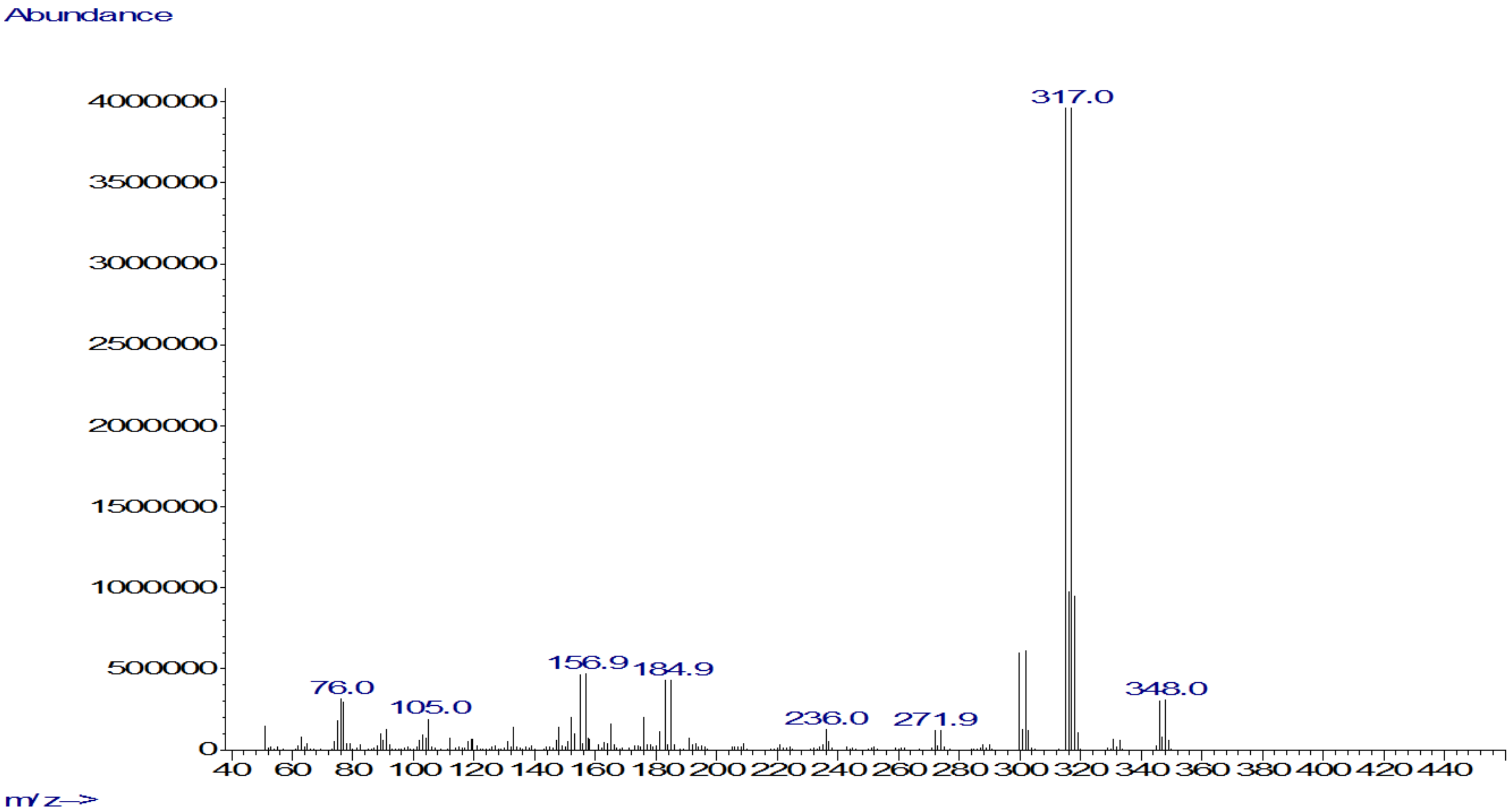

Compound 22

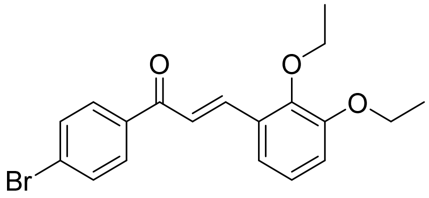

Molecular Weight: 375.26
m/z: 374.05 (100.0%), 376.05 (97.3%), 377.05 (20.0%), 375.06 (16.2%), 375.06 (4.3%), 378.06 (1.2%), 376.06 (1.1%)

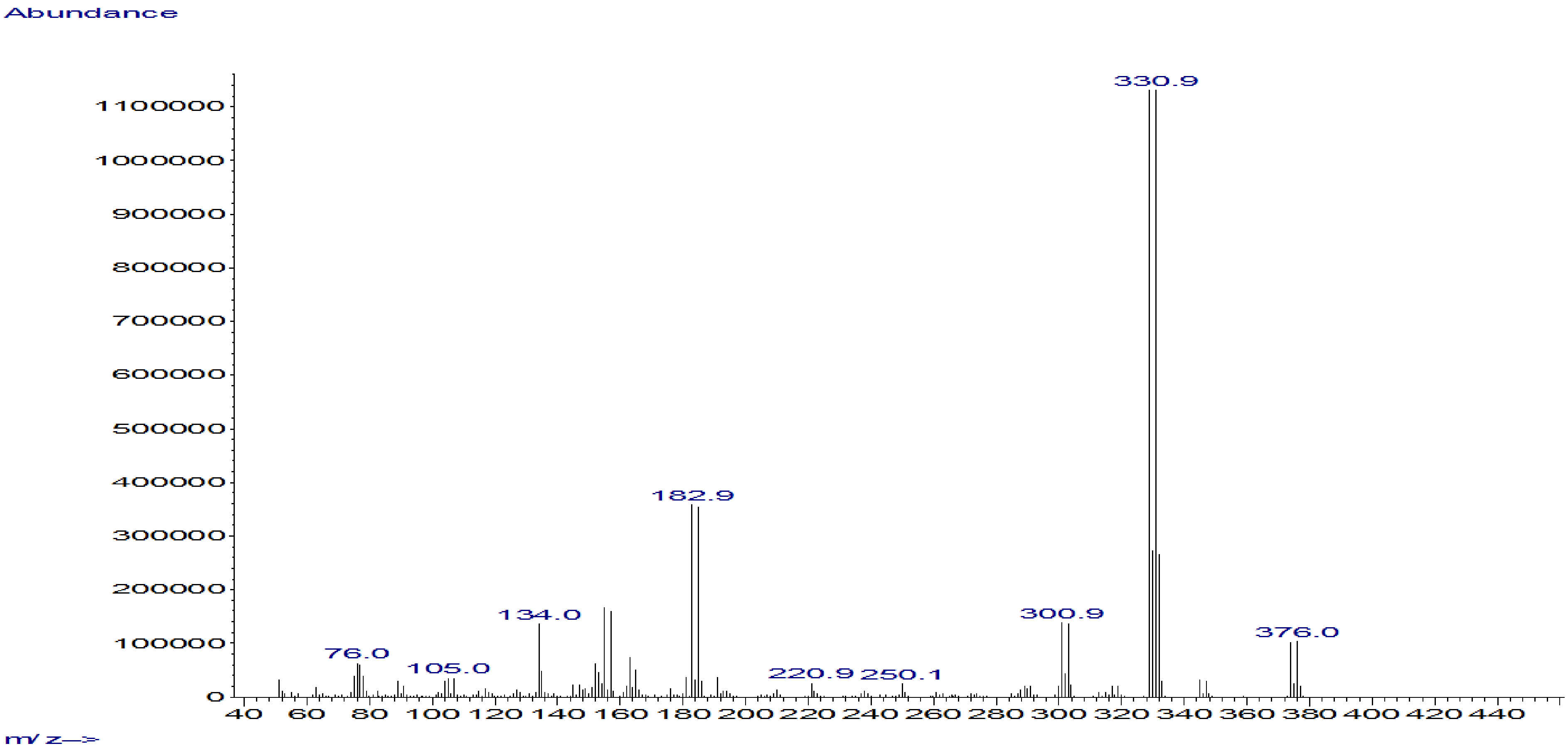

Supplement: Supplemental Material [file IENZ_A_1593158_SM9500.zip › IENZ_1593158_Supplementary Material/GC_MS_Manuscript.pdf]
